# *In silico* Selection of Amplification Targets for Rapid Polymorphism Screening in Ebola Virus Outbreaks

**DOI:** 10.3389/fmicb.2019.00857

**Published:** 2019-04-26

**Authors:** Trudy M. Wassenaar, Visanu Wanchai, Gregory S. Buzard, David W. Ussery

**Affiliations:** ^1^Molecular Microbiology and Genomics Consultants, Zotzenheim, Germany; ^2^Department of Biomedical Informatics, University of Arkansas for Medical Sciences, Little Rock, AR, United States; ^3^Retired, Middletown, MD, United States

**Keywords:** Ebola virus, transmission chain, virus evolution, mutation hotspot, PCR sequencing

## Abstract

To achieve maximum transmission chain tracking in the current Ebola outbreak, whole genome sequencing (WGS) has been proposed to provide optimal information. However, WGS remains a costly and time-intensive procedure that is poorly suited for the large numbers of samples being generated, especially under severe time and work-environment constraints as in the present DRC outbreak. To better prepare for future outbreaks, where an apparent single outbreak may actually represent overlapping outbreaks caused by independent variants, and where rapid identification of emerging new transmission chains will be essential, a more practical method would be to amplify and sequence genomic areas that reveal the highest information to differentiate EBOV variants. We have identified four highly informative polymorphism PCR sequencing targets, suitable for rapid tracing of transmission chains and identification of new sources of Ebola outbreaks, an approach which will be far more practical in the field than WGS.

## Introduction

The year 2018 saw two closely consecutive deadly Ebola virus zaire (EBOV) outbreaks in the Democratic Republic of the Congo (DRC), one beginning in May, the second in August. As a result of lessons learned during the catastrophic 2014 West Africa outbreak, local authorities and the world health organization (WHO) were better prepared to quickly quell the May outbreak, which lasted till July 25 (Muyembe Tamfum et al., 2018). However, the August outbreak is of growing concern, as the disease has been expanding inexorably within areas of a civil war zone in eastern DRC near the Uganda border, making the area inaccessible or unsafe for healthcare workers, and where monitoring and control has been extremely challenging (Nakkazi, 2018).

It is now apparent that these two DRC outbreaks, separated by only 7 days, were caused by two different variants of EBOV, with two independent initiating chains of human transmission (Muyembe Tamfum et al., 2018). Two independent outbreaks this close together geographically and temporally have not been previously described, as all previous major EBOV outbreaks were caused by sole-source, singular variants (Jun et al., 2015). There are as yet no easy methods in place to recognize and deal with potential situations where multiple distinct outbreak variants might be simultaneously circulating within the same geographical region.

Promising vaccination campaigns are ongoing (Lévy et al., 2018; Nakkazi, 2018; Saphire et al., 2018), and these may actually be effective (Ewer et al., 2018), but the currently available stockpile of EBOV vaccines is in limited supply (e.g., there are, at the time of this writing, only 300,000 doses of Merck’s rVSV-EBOV in stock) (Source^1^). Vaccine supplies could soon be exhausted, as adjoining countries such as Uganda and Sudan have now initiated prophylactic mass immunizations of their healthcare workers, and the virus has also now reached several major DRC cities, such as Butembo, a sprawling city home to over one million people, that is also close to the Ugandan border.

The determination of accurate transmission chains contributes to a higher efficacy for the ring vaccination method currently being used by the WHO in the DRC to contain EBOV; this reduces inappropriate distribution and improves the use of the limited amounts of available vaccine. The inability to achieve accurate determination of a chain-of-transmission can have consequences of potentially failing containment. A situation of emerging multiple circulating variants could easily worsen this scenario, as both the DRC and several of the surrounding countries have their own endemic reservoirs of EBOV (Caron et al., 2018) that could re-emerge at any moment.

Before their complete viral genome sequences were available, *Filoviridae* members were typically characterized by sequencing of RT-PCR amplicons of the two genes coding for glycoprotein (GP) and nucleoprotein (NP) (Wittmann et al., 2007). These PCR fragments were chosen to optimally differentiate between different Ebolavirus species and strains, but were never designed to optimally differentiate isolates within EBOV Zaire. Whole-genome sequencing (WGS) is another robust method to characterize virus variants, and this can now be achieved by high-throughput sequencing on portable devices (Quick et al., 2016).

Maximum transmission chain tracking could theoretically be achieved by WGS of all newly confirmed clinical cases. However, WGS remains a costly and time-intensive procedure, making it less suitable when applied to the large numbers of clinical isolates being generated in the DRC outbreak, especially under the severe time and work-environment constraints clinicians are facing there. WGS is not practical when expanding outbreaks are imminent to run out of control if novel chains of transmission are not identified and new containment efforts not initiated in a timely fashion. In order to better address the current DRC outbreak, and to better prepare for future outbreaks where an apparent single outbreak might actually represent overlapping outbreaks caused by multiple independent variants, and to aid in rapid identification of emerging novel transmission chains, it should, for now, be much more practical to amplify and sequence genomic areas that contain the highest information value to differentiate potential EBOV variants.

One cannot predict which combination of novel variants might cause a future outbreak; however, with an estimated substitution rate of 0.87 to 1.42 × 10^–3^ substitutions per site per year (Quick et al., 2016), one can be confident that every variant should be distinguishable, and thus likely that transmission chains can be differentiated too. Using bioinformatic analysis of polymorphic sites already described in historical isolates that belong to different EBOV outbreaks, we were able to deduce five focal regions in the EBOV genome that are the most likely to mutate, and thus be the most informative. These polymorphism hotspots should be the center of our attention for rapid variant screening protocols.

In order to identify these potential targets for variant-specific amplification, we have analyzed the polymorphic sites from the EBOV Zaire strains that have been isolated from Africa since the first outbreak, in 1976. Our analysis is based on *in silico* nucleotide sequence comparisons, in appreciation that *in silico* PCR is usually a reliable predictor for experimental performance, as we have demonstrated for other virus species (Alkam et al., 2017; Wongsurawat et al., 2018).

## Materials and Methods

### Original Dataset and Genome Atlas

**FIGURE 1:**
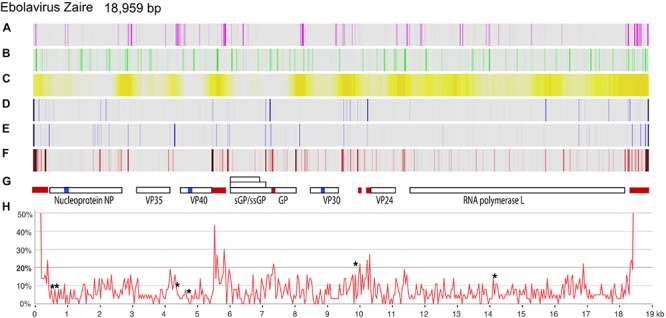
Genome atlas of the EBOV genome and variation plots of the two 2018 DRC outbreaks. As the reference genome, EBOV Zaire subtype Mayinga (AY142960.1) was used. Lanes **A–C** show local inverted repeats (capable of forming stem-loop structures), simple repeats, and percent AT, respectively; lanes **D,E** show the variation of the May 2018 (*n* = 17) and August 2018 (*n* = 15) DRC genomes, respectively, compared to the reference genome. Lane **F** represents all variations in 1,232 EBOV genomes. Lane **G** indicates the location of gene coding regions; three conserved stretches, of 99 bases or longer lacking polymorphisms, are shown as blue blocks, and five polymorphic stretches, of 79 bases or longer reaching >20% polymorphic sites, are shown as red blocks. Lane **H** shows the variation of all 1,232 EBOV genomes over a 37-bp window, expressed as a percentage. The asterisks identify the location of known microRNAs (as per 14). Both 5′- and 3′-ends of the virus reached 100% variation, which is omitted from lane **H**.

An initial dataset of 1,547 EBOV genomes was extracted from GenBank in December, 2018. These were curated to retain high-quality (no ambiguous sequences), full-length (>18,700 bp) and non-redundant genomes only, from which we retained 1,232 genomes. A genome atlas (Ussery et al., 2009) was produced based on the genome of EBOV Zaire – subtype Mayinga, GenBank accession number AY142960.1 (a 1976 isolate) as the reference genome. All lanes shown in Figure 1 represent the smoothed output over a window of 37 nucleotides (nt). This atlas was complemented with three lanes summarizing the variation (with respect to the reference genome) of the two recent 2018 DRC outbreaks (based on 17 genomes of the May outbreak and 15 genomes of the August outbreak), and displaying the variation of all 1,232 genomes.

### Multiple Alignments of Subsets

A subset of 28 highly informative EBOV genomes was based on our previously conducted phylogenetic analysis (Jun et al., 2015) comprised of the following genomes: AF499101, AY142960, AY354458, HQ613402, HQ613403, KC242785, KC242789, KC242790, KC242791, KC242792, KC242793, KC242794, KC242800, KF113528, KJ660348, KM034555, KM519951, KR105271,KT725333, KT762962, KY426696, KY471090, KY471092, MF102255, MH121164, MH470382, MH481611, and MH613311. These isolates were selected to cover maximum diversity (based on total genome phylogeny), covering all nine outbreaks prior to the August 2018 outbreak for which genomes were available at the time of our analysis. Additionally, all included genome sequences had to be in a continuous contig without ambiguities. Multiple alignments were performed using Muscle^2^, with default settings. Hotspots for polymorphism were defined as regions surrounding sites where the frequency of polymorphic sites was >20%, assessed using a 60 nt window. Conserved regions were defined as stretches of at least 99 nt devoid of polymorphisms.

**FIGURE 2:**
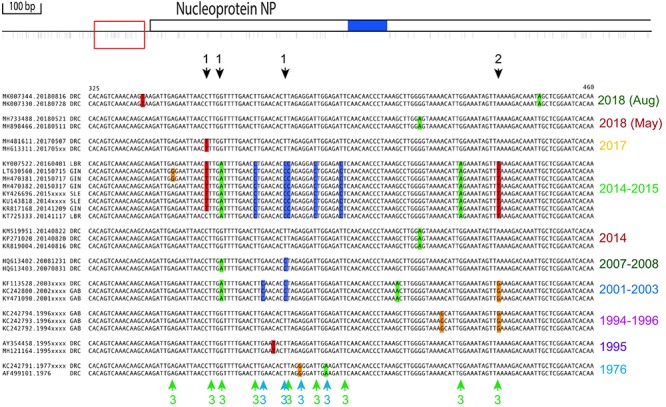
Polymorphic region upstream of NP. Top: All polymorphic sites identified in a region of 1,680 nt upstream of and within NP’s coding region (position 89 to 1770, based on genome sequence AY142960.1) shown as thin lines below the top graphic bar, as determined by alignment of 29 non-redundant historical isolates. The red square to the left represents the expanded alignment used below, based on 45 informative sequences from which redundancy was removed (see section “Materials and Methods”). Bottom: Black arrows above the alignment indicate polymorphisms (color-shaded nucleotides) occurring in multiple outbreaks and colored arrows below the alignment indicate outbreak-specific observations. Arrows are numbered for positions for which: (1) The same deviance from consensus is seen in more than one outbreak; (2) the deviance from consensus differs between outbreaks; and (3) the deviance from consensus is found within 10 bp in neighboring sites. The alignments are grouped per outbreak. Outbreaks and sequences within an outbreak are sorted chronologically, with the most recent outbreak shown at the top.

**FIGURE 3:**
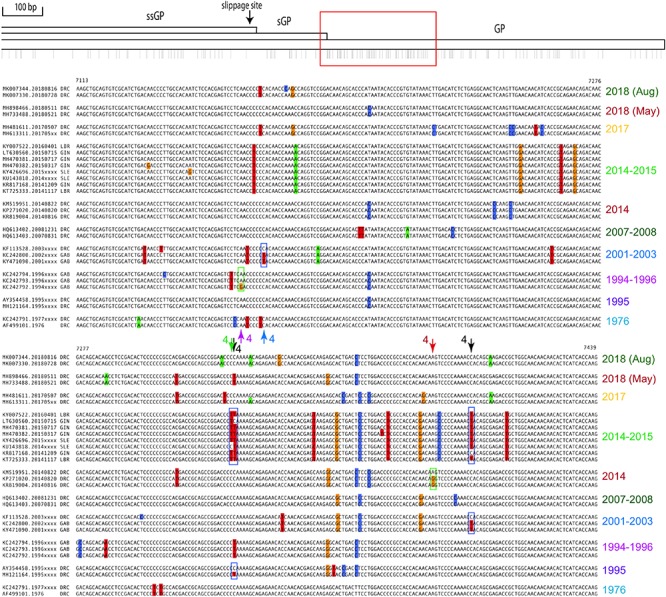
Polymorphic region within the coding sequences of GP. The top schematic represents the EVOV genome from position 6254 to 8068. Arrows in the multiple alignment marked 4 indicate examples where a back mutation toward the consensus occurred during an outbreak (boxed in the alignments). Black arrows indicate where this occurred in two outbreaks. For further explanation on the layout, see the legend of Figure 2.

For analysis of specific highly polymorphic sites (Figures 2, 3 and Supplementary Figures S1, S2), the dataset was extended to 40 genomes, to include genomes covering the maximum temporal spread within an outbreak. These also included five sequences from the DCR 2018 outbreaks available at the time of analysis. A minimum of three genomes per outbreak was included, covering polymorphisms within the defined fragments, if possible. For each shown multiple alignment, redundancy was removed, retaining at least two members per outbreak. Genome locations are numbered according to the nucleotide sequence of the Mayinga subtype of Zaire, GenBank accession number AY142960.1. The multiple alignment is presented as Supplementary Data Sheet S1.

### *In silico* PCR

For *in silico* PCR analysis, conserved regions flanking the highly polymorphic regions were selected and extended with upstream and downstream sequences to reach a continuous conserved length of at least 30 nt, and in total should be at least 50 nt, while not including more than three polymorphic sites per 60 nt, based on the 40 informative genomes. These regions were assessed for primer selection by Primer3 (Untergasser et al., 2012) and the top suggested primer pair was assessed for presence of polymorphisms by *in silico* PCR. For this, the two primers of each pair were concatenated and used as a query for Nblast searches in the non-redundant nucleotide database at NCBI, using default settings, with the output set for 10,000 retrieved hits. All retrieved hits were checked for a match with both halves of the concatenated query. Any hits to human sequences were recorded. Presence of mismatches in the queries (ignoring ambiguous sequences in the hits, when applicable) was recorded. If a primer pair covered too many polymorphic sites, the second, third, or fourth pair suggested by Primer3 was tested instead. For amplification of one region, primers were also manually selected.

### Phylogenetic Analysis

The sequences of amplification fragments identified by *in silico* PCR analysis were extracted from 40 informative FASTA files of complete EBOV genomes and after alignment these were subjected to phylogenetic analysis by Maximum Likelihood using IQ-Tree, which selected the best-fit substitution model for each analysis (Trifinopoulos et al., 2016). Each tree was rooted using the mid-point rooting method.

## Results

A genome atlas, based on a 1976 EBOV genome sequence, is shown in Figure 1, summarizing local inverted repeats, simple repeats and %AT, to which is added, in lanes D and E, the nucleotide differences of the variants causing the two 2018 DRC outbreaks. Lane F shows the overall variation of 1,232 EBOV genomes, which is plotted as percentages in panel H. As expected, intergenic regions are not only more AT-rich than coding regions, but are also more variable. These regions also more frequently contain inverted or direct repeats.

We identified three extended locations in the viral genome that seemed more resistant to mutations, covering 110 bases in NP (nucleotide positions 983 to 1092 with reference to genome AY142960.1), 108 bases in VP40 (positions 4704 to 4811), and 99 bases in VP30 (8812 to 8910) (blue blocks in Figure 1, lane G). One of these overlapped with the location of a microRNA (EBOV-pre-miVP coding for mature EBOV-miR-VP-3p, located on the minus strand) (Chen et al., 2016; Duy et al., 2018). This and other locations of miRNAs are indicated in the multiple alignment (Supplementary Data Sheet S1).

More importantly, we identified five hotspots for polymorphisms, defined as regions where the frequency of polymorphic sites was >20%. These hotspots were located in non-coding sequences upstream of the genes for NP (between positions 367 and 415), GP (5520 to 5876) and VP24 (10,157 to 10,324), within the coding region of GP (7243 to 7411), and downstream of L (18,313 to 18,375) (Figures 1F,H).

Four potential amplification products, containing “*drift-signature sites,”* were analyzed in detail, and past isolate deviations from a consensus sequence were recorded. The displayed dataset was reduced to show informative fragments only, with at least one genome per year of isolation shown for each outbreak.

The non-coding polymorphic region upstream of NP is shown in Figure 2. A number of independent outbreaks shared the same deviation from the consensus sequence, indicated by arrows numbered 1 in the figure, while in other instances the deviation at the same position differed between outbreaks (arrows numbered 2). A deviation from consensus was also frequently accompanied by another deviation in its immediate vicinity (within 10-bp) (arrows numbered 3).

Figure 3 shows the highly polymorphic region within the coding sequence of GP, for which similar observations as for Figure 2 can be made, though arrows for events 1 to 3 are no longer indicated. Arrows numbered 4 indicate positions for which a back mutation toward consensus during an outbreak can be postulated. For two positions there are even two independent examples for a back mutation, one occurring during the outbreak of 2014 (West Africa, WA, green date label) and also found in isolates from 1995, and the other shared by the outbreak of 2014 WA and that of 2001–2003 (black arrows numbered 4). The multiple alignments of the highly polymorphic intergenic region between VP40 and GP, and that upstream of VP24, are shown in Supplementary Figures S1, S2, respectively.

Next, we assessed *in silico* if the identified hypervariable regions could be used for PCR amplification, making use of their more conserved flanking regions as primer sites. The conserved flanking regions, as deduced from the 45-genome comparison, were extended to cover a conserved stretch of at least 30 nucleotides (see section “Materials and Methods”) and the consensus sequence of these potential target regions were used to predict amplification primers by means of Primer3 software. Predicted primers were then used in a blastN query of the non-redundant DNA database at NCBI. All retrieved hits were analyzed for eventual mismatches within the query sequences, correct amplicon length, and absence of hits to human DNA sequences. In one case, the locations of primer sequences were manually selected to cover the least possible number of polymorphic sites. The findings are summarized in Table 1.

**TABLE 1:** *In silico* PCR.

Predicted PCR primers and positions^1^	Total hits to EBOV sequences	EBOV hits with 100% primer match^2^	Hits with mismatch in F-primer^3^	Hits with mismatch in R-primer^3^	Total hits to human sequences
Upstream of GP, amplicon size 630 bp					
**T**GCAATAATTGAC**T**CAGATCCAGT (F, 5461 to 5484)	1,591	1,517	4	10	0
CGTGATCGAT**T**CAAGA***G***GACATC (R, 6069 to 6091)					

Upstream of GP, amplicon size 652 bp (manual)					
AGAAGTAAT***T***GCAATAATTG (F, 5452 to 5471)	1,587	1,509	0^4^	0	1 (partial)
GACATCATTCTTTCTTTGG (R, 6086 to 6104)					

Internal GP, amplicon size 351 bp					
CTGCAATGGTTCAAGTGCACA (F, 7080 to 7100)	1,572	1,528	0	1	0
GCTGGCAACAAC**A**ACACTCA (R, 7412 to 7431)					

Upstream of NP, amplicon size 318 bp					
**C**TCTGCAGGGTGATCC**A**ACA (F, 265 to 284)	1,555	1,513	3	0	0
AACAGGGGATTGTTCGGCAA (R, 564 to 583)					

Upstream of VP24, amplicon size 537 bp					
TAATGATGAAGATTAATGC**G**GAGGT (F, 9881 to 9905)	1,652	1546	1	0	0
AAAGGGGTTGTCTTAAGCGAC (R, 10,399 to 10,419)					

Downstream of L, amplicon size 355 bp					
AAGGCTGACAGGGCTTCTGA (F, 18,165 to 18,184)	1663	1450	0	4	0
AGGTCTGGGCTCATATTGT**T**AT**TG** (R, 18,497 to 18,520)					

*^1^Predictions are based on Primer3 analysis of conserved regions, except for the second primer pair for the region upstream of GP, which was selected manually. Positions found to be polymorphic when tested by *in silico* PCR are in bold. The reverse sequences are given in the positive direction and should be used as reverse-complement in case of PCR amplification. Positions are given with respect to AY142960.1. Also see their positions in the alignment (Supplementary Data Sheet S1). ^2^Only hits reported by both primers are listed; hits that were retrieved by only one primer were removed, even when the hit was found to be 100% identical. ^3^Only hits reported by both primers are considered here. ^4^The polymorphic position in the forward primer was not found in this *in silico* PCR as the four GenBank entries found with the Primer3 pair were incompletely sequenced and failed to cover the reverse primer.*

The *in silico* PCR analysis for amplification of the polymorphism hotspot fragment upstream of GP retrieved 1,591 EBOV sequences with the best primer set predicted by Primer3; of these, 1,517 produced a perfect match. A total of 60 hits were only retrieved with one of the two query primers, either due to submissions of incomplete genomes to GenBank or due to ambiguous sequences being present in the hits leading to a failing match. In four hits that were retrieved with both primers, the forward primer contained a mismatch due to a polymorphic nucleotide, with “C” replacing “T” at one of the two positions indicated in Table 1. The reverse primer also contained two positions that represented polymorphic sites, in 10 of the hits retrieved with both primers. Matches to human DNA were not identified. Manually we were able to select a reverse primer that was devoid of polymorphic positions, but the manually selected forward primer of that pair overlapped for 17 nucleotides at its 5′-end with a human sequence (Table 1). Since there was no hit with the corresponding reverse primer, production of an amplicon based on human DNA is highly unlikely.

Using the conserved sequences flanking the internal GP hotspot fragment, the top primer pair predicted by Primer3 identified 1,528 potential amplicons with perfect primer homology, while only one hit containing a mismatch in the reverse (R) primer. The predicted forward (F) primer for amplification of the upstream NP region retrieved two hits where the outermost (5′-) nucleotide was polymorphic, but also one where a mismatch was found in the more crucial 3′-terminal region; this primer pair retrieved 1,513 hits with perfect homology.

The overall conservation of the top two primer pairs predicted for amplification of VP24 upstream region was poor, but the third pair retrieved 1546 hits with 100% identity, plus one with a single mismatch in the forward primer. Similarly, the first three candidate primer pairs predicted for amplification of the region downstream of L covered too many polymorphic sites, but the fourth suggested pair was sufficient, although the reverse primer failed to match perfectly to four hits (Table 1).

**FIGURE 4:**
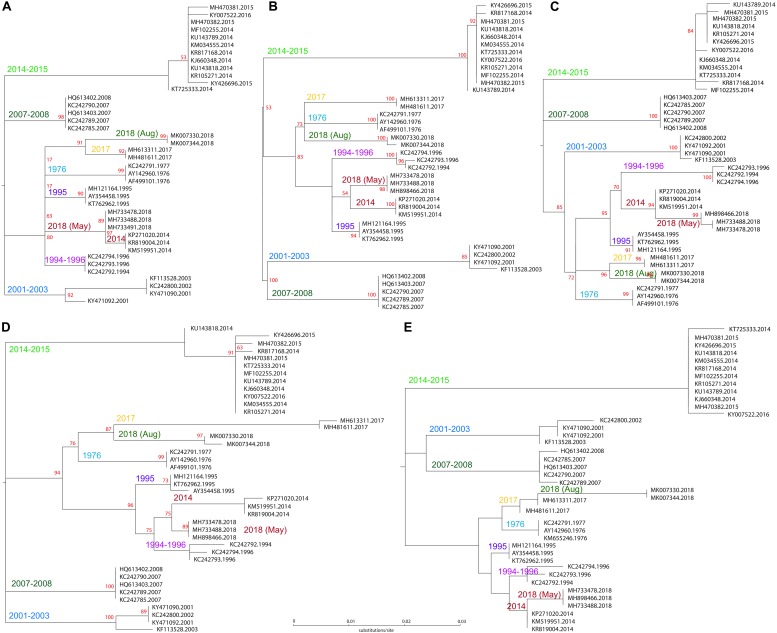
Phylogenetic analysis of *in silico* amplicons based on 40 genome sequences of past and present outbreaks. Maximum likelihood trees are based on **(A)** fragment upstream of NP, with the substitution model K3Pu+FF; **(B)** fragment intergenic of VP-GP (K3Pu+F model); **(C)** fragment upstream of VP24 (HKY+F model); **(D)** fragment internal of GP (TIM+F model); and **(E)** fragment downstream of L (K3Pu+F model).

In a last step, we digitally “extracted” the “amplicons” identified by *in silico* PCR (excluding the primer sequences) and constructed phylogenetic sequences based on 40 informative genome representatives of the analyzed outbreaks. Each of the *in silico* amplicons was sufficient to resolve phylogenetic relationships of the outbreaks (Figure 4).

## Discussion

Genome comparison of over 1,230 non-redundant, high quality EBOV full-length sequences within the Zaire lineage revealed both conserved and highly variable regions (Figure 1). The latter were concentrated in non-coding sequences, which were also more AT-rich than coding sequences, an observation that has also been made for other virus species, for instance Hepatitis B virus (González et al., 2018). The AT-content of coding sequences was most likely lower due to codon constraints, though we observe that the gene for RNA polymerase is richer in AT than the other EBOV genes. The analysis further identified the presence of local inverted repeats in intergenic regions that are likely to function in translation termination. The intergenic region between NP and VP35 was rich in AT but not enriched in polymorphic sequences, which may indicate other local constraints for conservation. One possibility is the presence of a pre-microRNA, whose processing would depend on conservation of inverted repeats (Liu et al., 2016). Indeed, these are present in the intergenic region of NP and VP35, although, to the best of our knowledge, no miRNA has yet been identified for this location.

Commercial companies producing drugs and detection technology are mostly interested in EBOV genomic regions that are highly conserved, as their products should not be affected by ongoing genetic changes. The three extended highly conserved regions identified, in genes coding for NP, VP40 and VP30, could be of interest for this purpose. One of these overlapped with the location of a microRNA (EBOV-miR-VP-3p, located on the minus strand), located in the coding region of VP40, and this microRNA was proposed to serve as a biomarker for early infection (Teng et al., 2015). Moreover, two of our predicted highly conserved primers partly overlapped with the location of miRNAs. The extensive genome comparison further revealed that all other proposed mature microRNA sequences derived from isolates of the 2014 outbreak were not completely conserved across EBOV Zaire, as mismatches were found in, for instance, EBOV-miR-1-3p [identified by Liu et al. (2016) based on the EBOV/Boende-Lokolia variant] and in EBOV-miR-T2-3p [identified by Teng et al. (2015), based on the EBOV/Makona variant].

We have further identified five sites displaying the highest frequencies of polymorphisms in the EBOV genome, which, along with conserved flanking sequences serving as stable amplification primer sites, were analyzed to assess suitability as amplification targets in future EBOV variants. We are proposing that these PCR targets will produce highly informative amplicons to feed into NGS rapid screening protocols for EBOV outbreak variant identification. These proposed PCR amplicons can be used for virus detection in clinical material, and, following amplicon sequencing, for tracking transmission chains or identifying new outbreaks. Based on the historical outbreaks of EBOV available so far, each of the proposed PCR amplicons can assign an isolate to its proper outbreak cluster, although the practical performance of the predicted primers still needs to be established experimentally. It cannot be excluded that a future outbreak with a new divergent variant would contain a substitution in crucial positions of one or multiple primers, while efficiency of amplification can also be hampered by hairpins and other secondary structures (Crary et al., 2003). The latter are particularly found toward the 5′-end of the viral genome, which not only hampers amplification but also makes sequencing more difficult. Of the roughly 50,000 Ebola entries at NCBI, to date only 1,547 represent full-length genomes. The variability in both terminal regions may be reflected by a slight potential for a failure of the 5′-NP forward primer and any future 3′-L reverse primers annealing during reverse transcription, amplification or sequencing.

There are multiple reasons why all of the intergenic and extragenic regions are not uniformly polymorphic, as there are important constraints to protect certain regions from mutations, while other regions are more easily selected for variation. For instance, the highly variable hotspot internal of the GP coding region likely reflects adaption to the human host (Quinlan et al., 2017; Ruedas et al., 2018).

Because the internal amplicon sequences were selected here for their tendency for variation, they are thus far less suitable for design of internal probes used for rapid identification and for quantification. Numerous EBOV detection methods based on amplification of conserved GP or NP sequences have been described (e.g., 23–25), whose primers are indicated in the Supplementary Data Sheet S1. These were not necessarily designed for phylogenetic assessment or transmission chain identification. As a consequence, the NP amplicon proposed by Lau et al. (2017) would only capture four polymorphic sites (plus two located in their primers). Their proposed primers for amplification of a GP1 fragment were not conserved (though interestingly, these surrounded our conserved reverse GP primer), and their degenerate primers for a GP2 fragment targeted a strongly conserved region that would not resolve individual lineages. The F-primer targeting GP sequences proposed by Ro et al. (2017) was not conserved in the critical 3′-terminal nucleotides, while their amplicon was too conserved to be used for transmission chain identification. The probe sequence proposed for rt-PCR by Dedkov et al. (2016) was well conserved, though their amplification primers were not, as indicated in the Supplementary Data Sheet S1.

The primary proposed use of the four hotspots discussed here would be for rapid field identification of EBOV polymorphisms in human clinical samples. This information could provide timely information in case of emergence of a new chain of human transmission in an on-going outbreak, or the appearance of an entirely independent variant outbreak, which, given the propensity of EBOV to reemerge at ever more frequent intervals in Central Africa, is like to occur, sooner or later.

Compared to deep sequencing of the entire genome, the *in silico* resolution of each of the hotspot amplicons described here might be faster and more cost effective for future outbreak investigations and rapid screening, eventually to be followed up by WGS on a selection of isolates to investigate phylogenetic relationships in detail. However, for EBOV transmission chain tracking, WGS is not required, as sequencing of highly polymorphic regions will most likely be sufficient.

Where reasonable, we do support the eventual generation of whole genome sequences from outbreak isolates, as that provides the most complete information on virus isolates. However, when resources are limited, the environment challenging, and time is of the essence, that approach is not usually feasible. During surging outbreaks like the current one in the DRC, we recommend concentrating real-time clinical sequencing efforts to screening of the amplification products of EBOV mutation hotspots, i.e., the polymorphism-rich “*drift-signature sites*” which we describe here, as they are likely to be the most immediately and significantly informative.

## Author Contributions

GB and TW conceived the study. TW and VW defined the datasets, and produced the alignment and figures. GB, TW, and DU produced the first draft of the manuscript. All authors edited and approved of the final manuscript.

## Conflict of Interest Statement

The authors declare that the research was conducted in the absence of any commercial or financial relationships that could be construed as a potential conflict of interest.

## Footnotes

1 www.businesswire.com

2 https://www.ebi.ac.uk/Tools/msa/muscle

## Supplementary Material

FIGURE S1 Polymorphic region separating VP40 and GP.

FIGURE S2 Polymorphic region upstream of VP24.

DATA SHEET S1 Multiple alignment with annotations of historical outbreaks.
