# Supplementary figures and images for: In silico Selection of Amplification Targets for Rapid Polymorphism Screening in Ebola Virus Outbreaks
Source: Front Microbiol. 2019 Apr 26;10:857. doi: 10.3389/fmicb.2019.00857 (PMC6497787; doi:10.3389/fmicb.2019.00857)

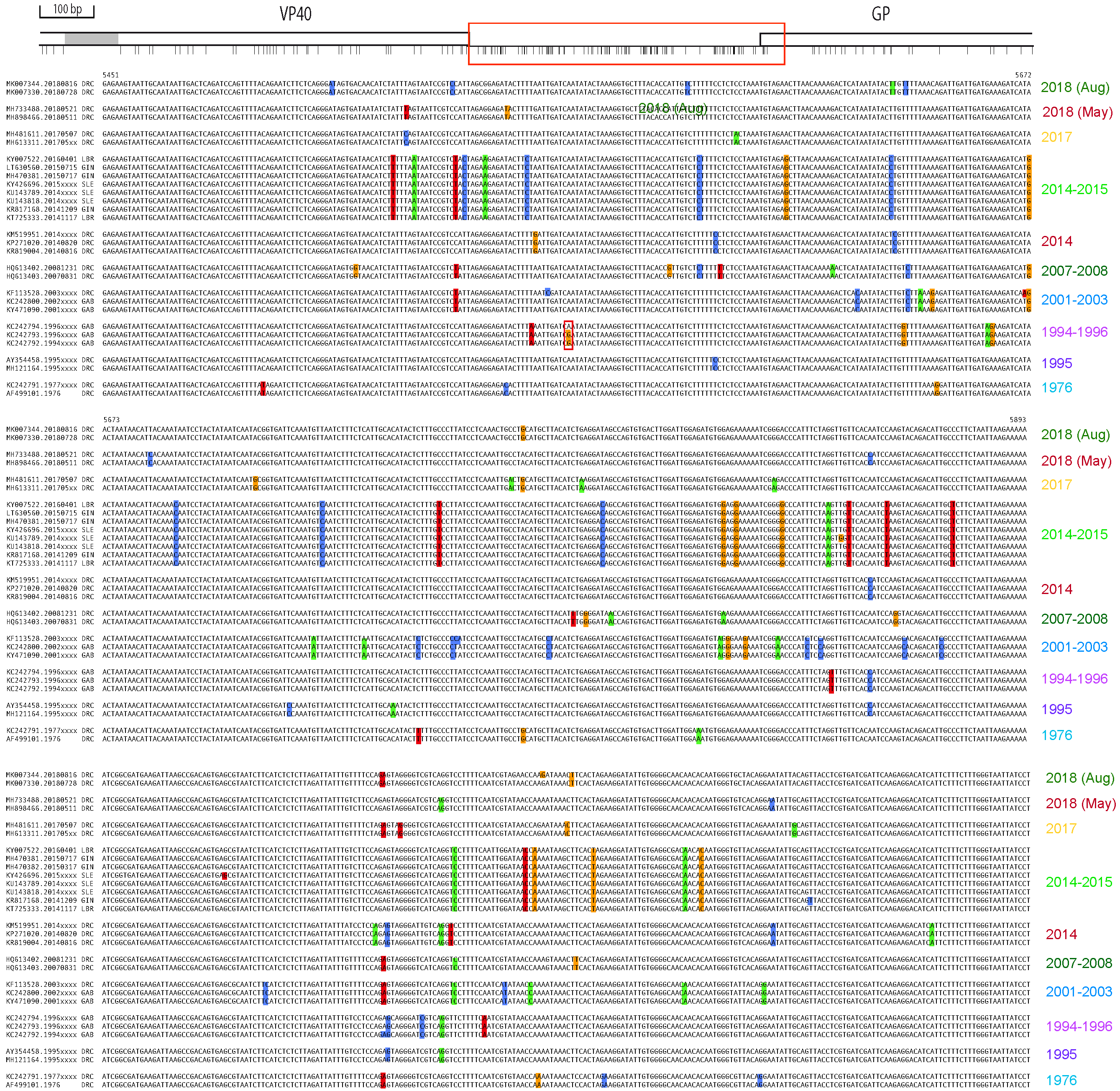

Supplement: FIGURE S1 — Polymorphic region separating VP40 and GP. [file Image_1.png]

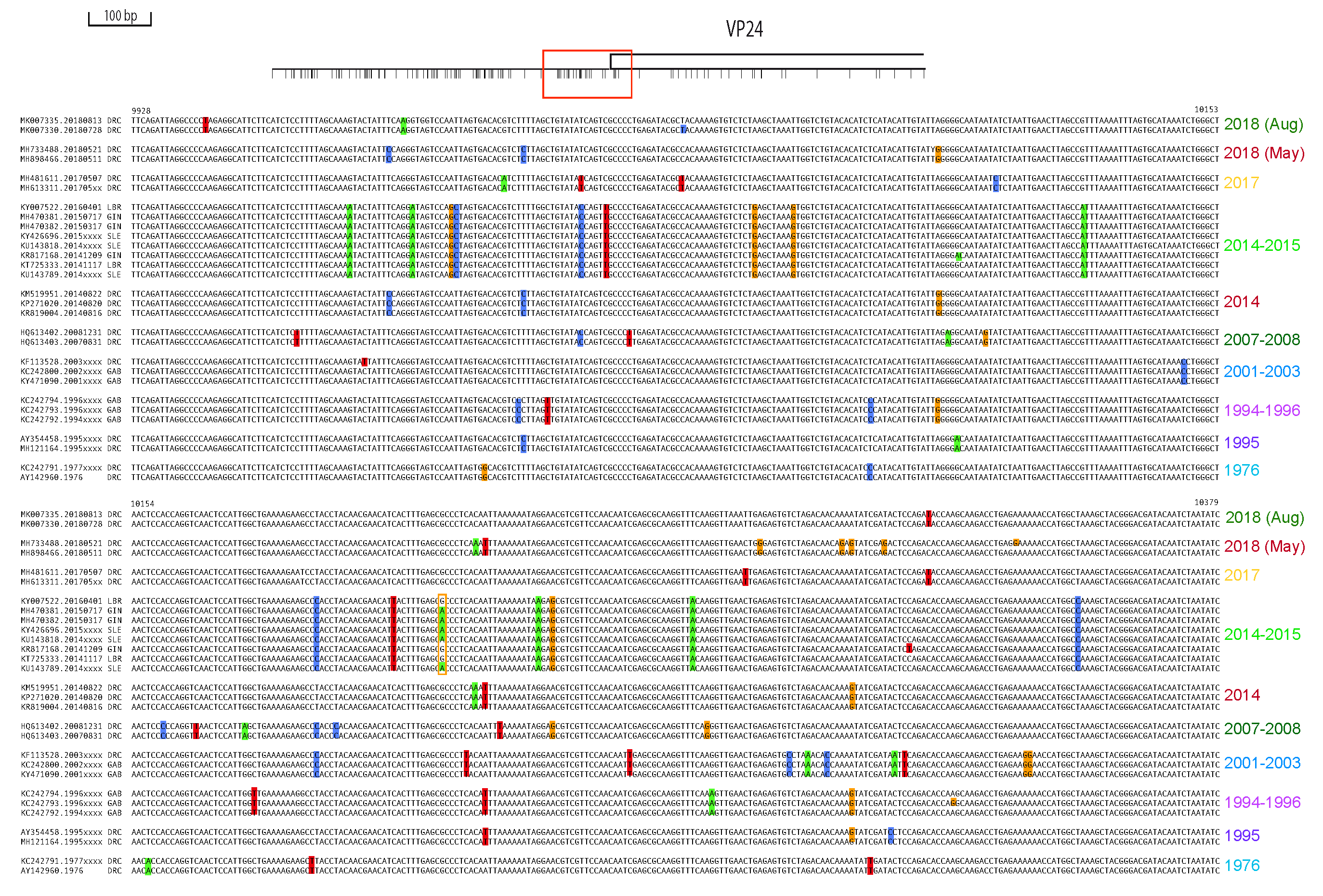

Supplement: FIGURE S2 — Polymorphic region upstream of VP24. [file Image_2.png]
