# Supplementary material for: In silico Selection of Amplification Targets for Rapid Polymorphism Screening in Ebola Virus Outbreaks
Source: Front Microbiol. 2019 Apr 26;10:857. doi: 10.3389/fmicb.2019.00857 (PMC6497787; doi:10.3389/fmicb.2019.00857)
Supplement: DATA SHEET S1 — Multiple alignment with annotations of historical outbreaks. [file Data_Sheet_1.doc]

**Supplementary file Wassenaar et al.**

# Amplification targets for rapid polymorphism screening in Ebola virus outbreaks

**Multiple alignment of 40 Ebolavirus Zaire genomes representing all historical outbreaks.**

Colors and formats are used to indicate the following features, with annotations to the right of the alignment:

ATG TGA start and stop codons of proteins with annotation to the right

************* stretch of continuously conserved positions longer than 100 bp

CTCTGCAGGGTGA Forward primer predicted by Primer3. Note, that the partly overlapping primer selected

manually for amplification of the upstream region of GP is not indicated.

AACAGGGGATTGT Reverse primer predicted by Primer3. Note, that the partly overlapping primer selected

manually for amplification of the upstream region of GP is not indicated.

ATCTGACATGGAT Underlined: conserved miRNA according to Duy et al., Sci Rep. 2018;8(1):6480

*ATCTGACATGGAT* Italics: amplification probes for detection and identification according to Lau et al., J Clin

Microbiol. 2017;55(3):783-790, to Ro et al., Microbiol Immunol. 2017;61(3-4):130-137,

and to Dedkov et al., J Virol Methods. 2016;228:26-30.

*************Conserved detection probe according to Ro et al., Microbiol Immunol. 2017;61(3-4):130-

137, and to Dedkov et al., J Virol Methods. 2016;228:26-30.

AY142960.1976 CGGACACACAAAAAGAAAGAAGAATTTTTAGGATCTTTTGTGTGCGAATAACTATGAGGA 60

KC242791.1977 CGGACACACAAAAAGAAAGAAGAATTTTTAGGATCTTTTGTGTGCGAATAACTATGAGGA 60

AF499101.1976 CGGACACACAAAAAGAAAGAAGAATTTTTAGGATCTTTTGTGTGCGAATAACTATGAGGA 60

KF113528.2003 ----CACACAAAAAGAAAGAAGAATTTTTAGGATCTTTTGTATGCGAATAACTATGAGGA 56

KC242800.2002 CGGACACACAAAAAGAAAGAAGAATTTTTAGGATCTTTTGTGTGCGAATAACTATGAGGA 60

KY471090.2001 -----------------------ATTTTTAGGATCTTTTGTGTGCGAATAACTATGAGGA 37

KY471092.2001 -----------------------ATTTTTAGGATCTTTTGTGTGCGAATAACTATGAGGA 37

MH481611.2017 ----------------------------------CTTTTGTGTGCGAATAACTATGAGGA 26

MH613311.2017 -------------------------------------------------------GAGGA 5

KC242792.1994 CGGACACACAAAAAGAAAGAAGAATTTTTAGGATCTTTTGTGTGCGAATAACTATGAGGA 60

KC242793.1996 CGGACACACAAAAAGAAAGAAGAATTTTTAGGATCTTTTGTGTGCGAATAACTATGAGGA 60

KC242794.1996 CGGACACACAAAAAGAAAGAAGAATTTTTAGGATCTTTTGTGTGCGAATAACTATGAGGA 60

MH121164.1995 CGGACACACAAAAAGAAAGAAGAATTTTTAGGATCTTTTGTGTGCGAATAACTATGAGGA 60

AY354458.1995 CGGACACACAAAAAGAAAGAAGAATTTTTAGGATCTTTTGTGTGCGAATAACTATGAGGA 60

KT762962.1995 CGGACACACAAAAAGAAAGAAGAATTTTTAGGATCTTTTGTGTGCGAATAACTATGAGGA 60

HQ613402.2008 ------------------------------------------------------------ 0

KC242789.2007 CGGACACACAAAAAGAAAGAAGAATTTTTAGGATCTTTTGTGTGCGAATAACTATGAGGA 60

HQ613403.2007 ------------------------------------------------TAACTATGAGGA 12

KC242785.2007 CGGACACACAAAAAGAAAGAAGAATTTTTAGGATCTTTTGTGTGCGAATAACTATGAGGA 60

KC242790.2007 CGGACACACAAAAAGAAAGAAGAATTTTTAGGATCTTTTGTGTGCGAATAACTATGAGGA 60

KU143789.2014 CGGACACACAAAAAGAAAGAAGAATTTTTAGGATCTTTTGTGTGCGAATAACTATGAGGA 60

KR817168.2014 --------------------------TTTAGGATCTTTTGTGTGCGAATAACTATGAGGA 34

KY426696.2015 CGGACACACAAAAAGAAAGAAGAATTTTTAGGATCTTTTGTGTGCGAATAACTATGAGGA 60

KR105271.2014 ---------------------------------TCTTTTGTGTGCGAATAACTATGAGGA 27

KY007522.2016 --------------------AGAATTTTTAGGATCTTTTGTGTGCGAATAACTATGAGGA 40

KM034555.2014 --------CAAAAAGAAAGAAGAATTTTTAGGATCTTTTGTGTGCGAATAACTATGAGGA 52

MH470381.2015 ------------------------------------TTTGTGTGCGAATAACTATGAGGA 24

MH470382.2015 ------------------------------------TTTGTGTGCGAATAACTATGAGGA 24

MF102255.2014 -----------------------ATTTTTAGGATCTTTTGTGTGCGAATAACTATGAGGA 37

KJ660348.2014 CGGACACACAAAAAGAAAGAAGAATTTTTAGGATCTTTTGTGTGCGAATAACTATGAGGA 60

KU143818.2014 CGGACACACAAAAAGAAAGAAGAATTTTTAGGATCTTTTGTGTGCGAATAACTATGAGGA 60

KT725333.2014 -----------------------------------TTTTGTGTGCGAATAACTATGAGGA 25

KR819004.2014 -----------------------------------------------------ATGAGGA 7

KP271020.2014 -----------------------------------------------------ATGAGGA 7

KM519951.2014 ---ACACACAAAAAGAAAGAAGAATTTTTAGGATCTTTTGTGTGCGAATAACTATGAGGA 57

MH733488.2018 ---------AAAAAGAAAGAAGAATTTTTAGGATCTTTTGTGTGCGAATAACTATGAGGA 51

MH733491.2018 --------------GAAAGAAGAATTTTTAGGATCTTTTGTGTGCGAATAACTATGAGGA 46

MH733478.2018 -----------AAAGAAAGAAGAATTTTTAGGATCTTTTGTGTGCGAATAACTATGAGGA 49

MK007330.2018 ------------AAGAAAGAAGAATTTTTAGGATCTTTTGTGTGCGAATAACTATGAGGA 48

MK007344.2018 ------------------GAAGAATTTTTAGGATCTTTTGTGTGCGAATAACTATGAGGA 42

AY142960.1976 AGATTAATAATTTTCCTCTCATTGAAATTTATATCGGAATTTAAATTGAAATTGTTACTG 120

KC242791.1977 AGATTAATAATTTTCCTCTCATTGAAATTTATATCGGAATTTAAATTGAAATTGTTACTG 120

AF499101.1976 AGATTAATAATTTTCCTCTCATTGAAATTTATATCGGAATTTAAATTGAAATTGTTACTG 120

KF113528.2003 AGATTAATAATCTTCCTCTCATTGAAATTTATATCGGAATTTAAATTGAAATTGTTACTG 116

KC242800.2002 AGATTAATAATTTTCCTCTCATTGAAATTTATATCGGAATTTAAATTGAAATTGTTACTG 120

KY471090.2001 AGATTAATAATTTTCCTCTCATTGAAATTTATATCGGAATTTAAATTGAAATTGTTACTG 97

KY471092.2001 AGATTAATAATTTTCCTCTCATTGAAATTTATATCGGAATTTAAATTGAAATTGTTACTG 97

MH481611.2017 AGATTAATAATTTTCCTCTCACTGAAATCTATATCGGAATTTAAATTGAAATTGTTACTG 86

MH613311.2017 AAATTATTAATCTTCCTCTCACTGAAATCTATATCGGAATTTAAATTGAAATTGTTACTG 65

KC242792.1994 AGATTAATAATTTTCCTCTCATTGAAATTTATATCGGAATTTAAATTGAAATTGTTACTG 120

KC242793.1996 AGATTAATAATTTTCCTCTCATTGAAATTTATATCGGAATTTAAATTGAAATTGTTACTG 120

KC242794.1996 AGATTAATAATTTTCCTCTCATTGAAATTTATATCGGAATTTAAATTGAAATTGTTACTG 120

MH121164.1995 AGATTAATAATTTTCCTCTCATTGAAATTTATATCGGAATTTAAATTGAAATTGTTACTG 120

AY354458.1995 AGATTAATAATTTTCCTCTCATTGAAATTTATATCGGAATTTAAATTGAAATTGTTACTG 120

KT762962.1995 AGATTAATAATTTTCCTCTCATTGAAATTTATATCGGAATTTAAATTGAAATTGTTACTG 120

HQ613402.2008 ---------------------TTGAAATTTATATCGGAATTTAAATTGAAATTGTTACTG 39

KC242789.2007 AGATTAATAATTTTCCTCTCATTGAAATTTATATCGGAATTTAAATTGAAATTGTTACTG 120

HQ613403.2007 AGATTAATAATTTTCCTCTCATTGAAATTTATATCGGAATTTAAATTGAAATTGTTACTG 72

KC242785.2007 AGATTAATAATTTTCCTCTCATTGAAATTTATATCGGAATTTAAATTGAAATTGTTACTG 120

KC242790.2007 AGATTAATAATTTTCCTCTCATTGAAATTTATATCGGAATTTAAATTGAAATTGTTACTG 120

KU143789.2014 AGATTAATAATTTTCCTCTCATTGAAATTTATATCGGAATTTAAATTGAAATTGTTACTG 120

KR817168.2014 AGATTAATAATTTTCCTCTCATTGAAATTTATATCGGAATTTAAATTGAAATTGTTACTG 94

KY426696.2015 AGATTAATAATTTTCCTCTCATTGAAATTTATATCGGAATTTAAATTGAAATTGTTACTG 120

KR105271.2014 AGATTAATAATTTTCCTCTCATTGAAATTTATATCGGAATTTAAATTGAAATTGTTACTG 87

KY007522.2016 AGATTAATAATTTTCCTCTCATTGAAATTTATATCGGAATTTAAATTGAAATTGTTACTG 100

KM034555.2014 AGATTAATAATTTTCCTCTCATTGAAATTTATATCGGAATTTAAATTGAAATTGTTACTG 112

MH470381.2015 AGATTAATAATTTTCCTCTCATTGAAATTTATATCAGAATTTAAATTGAAATTGTTACTG 84

MH470382.2015 AGATTAATAATTTTCCTCTCATTGAAATTTATATCGGAATTTAAATTGAAATTGTTACTG 84

MF102255.2014 AGATTAATAATTTTCCTCTCATTGAAATTTATATCGGAATTTAAATTGAAATTGTTACTG 97

KJ660348.2014 AGATTAATAATTTTCCTCTCATTGAAATTTATATCGGAATTTAAATTGAAATTGTTACTG 120

KU143818.2014 AGATTAATAATTTTCCTCTCATTGAAATTTATATCGGAATTTAAATTGAAATTGTTACTG 120

KT725333.2014 AGATTAATAATTTTCCTCTCATTGAAATTTATATCGGAATTTAAATTGAAATTGTTACTG 85

KR819004.2014 AGATTAATAATTTTCCTCTCATTGAAATTTATATCGGAATTTAAATTGAAATTGTTACTG 67

KP271020.2014 AGATTAATAATTTTCCTCTCATTGAAATTTATATCGGAATTTAAATTGAAATTGTTACTG 67

KM519951.2014 AGATTAATAATTTTCCTCTCATTGAAATTTATATCGGAATTTAAATTGAAATTGTTACTG 117

MH733488.2018 AGATTAATAATTTTCCTCTCATTGAAATTTATATCGGAATTTAAATTGAAATTGTTACTG 111

MH733491.2018 AGATTAATAATTTTCCTCTCATTGAAATTTATATCGGAATTTAAATTGAAATTGTTACTG 106

MH733478.2018 AGATTAATAATTTTCCTCTCATTGAAATTTATATCGGAATTTAAATTGAAATTGTTACTG 109

MK007330.2018 AGATTAATAATTTTCCTCTCATTGAAATTTATATCGGAATTTAAATTGAAATTGTTACTG 108

MK007344.2018 AGATTAATAATTTTCCTCTCATTGAAATTTATATCGGAATTTAAATTGAAATTGTTACTG 102

****** ****** ************************

AY142960.1976 TAATCACACCTGGTTTGTTTCAGAGCCACATCACAAAGATAGAGAACAACCTAGGTCTCC 180

KC242791.1977 TAATCACACCTGGTTTGTTTCAGAGCCACATCACAAAGATAGAGAACAACCTAGGTCTCC 180

AF499101.1976 TAATCACACCTGGTTTGTTTCAGAGCCACATCACAAAGATAGAGAACAACCTAGGTCTCC 180

KF113528.2003 TAATCACACCTGGTTTGTTTCAGAGCCACATCACAAAGATAGAGAACAGCCTAGGTCTCC 176

KC242800.2002 TAATCACACCTGGTTTGTTTCAGAGCCACATCACAAAGATAGAGAACAGCCTAGGTCTCC 180

KY471090.2001 TAATCACACCTGGTTTGTTTCAGAGCCACATCACAAAGATAGAGAACAGCCTAGGTCTCC 157

KY471092.2001 TAATCACACCTGGTTTGTTTCAGAGCCACATCACAAAGATAGAGAACAGCCTAGGTCTCC 157

MH481611.2017 TAATCACACCTGGTTTGTTTCAGAGCCACATCACAAAGATAGAGAACAACCTAGGTCTCC 146

MH613311.2017 TAATCACACCTGGTTTGTTTCAGAGCCACATCACAAAGATAGAGAACAACCTAGGTCTCC 125

KC242792.1994 TAATCACACCTGGTTTGTTTCAGAGCCACATCACAAAGATAGAGAACAACCTAGGTCTCT 180

KC242793.1996 TAATCACACCTGGTTTGTTTCAGAGCCACATCACAAAGATAGAGAACAACCTAGGTCTCT 180

KC242794.1996 TAATCACACCTGGTTTGTTTCAGAGCCACATCACAAAGATAGAGAACAACCTAGGTCTCT 180

MH121164.1995 TAATCACACCTGGTTTGTTTCAGAGCCACATCACAAAGATAGAGAACAACCTAGGTCTCT 180

AY354458.1995 TAATCACACCTGGTTTGTTTCAGAGCCACATCACAAAGATAGAGAACAACCTAGGTCTCT 180

KT762962.1995 TAATCACACCTGGTTTGTTTCAGAGCCACATCACAAAGATAGAGAACAACCTAGGTCTCT 180

HQ613402.2008 TAATCACACCTGTTTTGTTTCAGAGCCATGTCACAAAGATAGAGAACAACCTAGGTCTCC 99

KC242789.2007 TAATCACACCTGTTTTGTTTCAGAGCCATGTCACAAAGATAGAGAACAACCTAGGTCTCC 180

HQ613403.2007 TAATCACACCTGTTTTGTTTCAGAGCCATGTCACAAAGATAGAGAACAACCTAGGTCTCC 132

KC242785.2007 TAATCACACCTGTTTTGTTTCAGAGCCATGTCACAAAGATAGAGAACAACCTAGGTCTCC 180

KC242790.2007 TAATCACACCTGTTTTGTTTCAGAGCCATGTCACAAAGATAGAGAACAACCTAGGTCTCC 180

KU143789.2014 TAATCATACCTGGTTTGTTTCAGAGCCATATCACCAAGATAGAGAACAACCTAGGTCTCC 180

KR817168.2014 TAATCATACCTGGTTTGTTTCAGAGCCATATCACCAAGATAGAGAACAACCTAGGTCTCC 154

KY426696.2015 TAATCATACCTGGTTTGTTTCAGAGCCATATCACCAAGATAGAGAACAACCTAGGTCTCC 180

KR105271.2014 TAATCATACCTGGTTTGTTTCAGAGCCATATCACCAAGATAGAGAACAACCTAGGTCTCC 147

KY007522.2016 TAATCATACCTGGTTTGTTTCAGAGCCATATCACCAAGATAGAGAACAACCTAGGTCTCC 160

KM034555.2014 TAATCATACCTGGTTTGTTTCAGAGCCATATCACCAAGATAGAGAACAACCTAGGTCTCC 172

MH470381.2015 TAATCATACCTGGTTTGTTTCAGAGCCATATCACCAAGATAGAGAACAACCTAGGTCTCC 144

MH470382.2015 TAATCATACCTGGTTTGTTTCAGAGCCATATCACCAAGATAGAGAACAACCTAGGTCTCC 144

MF102255.2014 TAATCATACCTGGTTTGTTTCAGAGCCATATCACCAAGATAGAGAACAACCTAGGTCTCC 157

KJ660348.2014 TAATCATACCTGGTTTGTTTCAGAGCCATATCACCAAGATAGAGAACAACCTAGGTCTCC 180

KU143818.2014 TAATCATACCTGGTTTGTTTCAGAGCCATATCACCAAGATAGAGAACAACCTAGGTCTCC 180

KT725333.2014 TAATCATACCTGGTTTGTTTCAGAGCCATATCACCAAGATAGAGAACAGCCTAGGTCTCC 145

KR819004.2014 TAATCACACCTGTTTTGTTTCAGAGCCACATCACAAAGATAGAGAACAACCTAGGTCTCT 127

KP271020.2014 TAATCACACCTGTTTTGTTTCAGAGCCACATCACAAAGATAGAGAACAACCTAGGTCTCT 127

KM519951.2014 TAATCACACCTGTTTTGTTTCAGAGCCACATCACAAAGATAGAGAACAACCTAGGTCTCT 177

MH733488.2018 TAATCACACCTGGTTTGTTTCAGAGCCACATCACAAAGATAGAGAACAACCTAGGTCTCT 171

MH733491.2018 TAATCACACCTGGTTTGTTTCAGAGCCACATCACAAAGATAGAGAACAACCTAGGTCTCT 166

MH733478.2018 TAATCACACCTGGTTTGTTTCAGAGCCACATCACAAAGATAGAGAACAACCTAGGTCTCT 169

MK007330.2018 TAATCACACCTGGTTTGTTTCAGAGCCACATCACAAAGATAGAGAACAACCTAGGTCTCC 168

MK007344.2018 TAATCACACCTGGTTTGTTTCAGAGCCACATCACAAAGATAGAGAACAACCTAGGTCTCC 162

****** ***** *************** **** ************* **********

AY142960.1976 GAAGGGAGCAAGGGCATCAGTGTGCTCAGTTGAAAATCCCTTGTCAACACCTAGGTCTTA 240

KC242791.1977 GAAGGGAGCAAGGGCATCAGTGTGCTCAGTTGAAAATCCCTTGTCAACACCTAGGTCTTA 240

AF499101.1976 GAAGGGAGCAAGGGCATCAGTGTGCTCAGTTGAAAATCCCTTGTCAACACCTAGGTCTTA 240

KF113528.2003 GAAGGGAACAAGGGCACCAGTGTGCTCAGTTGAAAATCCCTTGTCAACATCTAGGTCTTA 236

KC242800.2002 GAAGGGAACAAGGGCACCAGTGTGCTCAGTTGAAAATCCCTTGTCAACATCTAGGTCTTA 240

KY471090.2001 GAAGGGAACAAGGGCACCAGTGTGCTCAGTTGAAAATCCCTTGTCAACATCTAGGTCTTA 217

KY471092.2001 GAAGGGAACAAGGGCACCAGTGTGCTCAGTTGAAAATCCCTTGTCAACATCTAGGTCTTA 217

MH481611.2017 GAAGGGAGCAAGGGCATCAGTGTGCCCAGTTGAAAATCCCTTGTCAACATCTAGGTCTTA 206

MH613311.2017 GAAGGGAGCAAGGGCATCAGTGTGCCCAGTTGAAAATCCCTTGTCAACATCTAGGTCTTA 185

KC242792.1994 GAAGGGAGCAAGGGCATCAGTGTGCTCAGTTGAAAATCCCTTGTCAACATCTAGGTCTTA 240

KC242793.1996 GAAGGGAGCAAGGGCATCAGTGTGCTCAGTTGAAGATCCCTTGTCAACATCTAGGTCTTA 240

KC242794.1996 GAAGGGAGCAAGGGCATCAGTGTGCTCAGTTGAAAATCCCTTGTCAACATCTAGGTCTTA 240

MH121164.1995 GAAGGGAGCAAGGGCATCAGTGTGCTCAGTTGAAAATCCCTTGTCAACATCTAGGTCTTA 240

AY354458.1995 GAAGGGAGCAAGGGCATCAGTGTGCTCAGTTGAAAATCCCTTGTCAACATCTAGGTCTTA 240

KT762962.1995 GAAGGGAGCAAGGGCATCAGTGTGCTCAGTTGAAAATCCCTTGTCAACATCTAGGTCTTA 240

HQ613402.2008 GAAGGGAGCAAGGGCATCAGTGTGCTCAGTTGAAAATCCCTTGTCAACATCTAGGTCTTA 159

KC242789.2007 GAAGGGAGCAAGGGCATCAGTGTGCTCAGTTGAAAATCCCTTGTCAACATCTAGGTCTTA 240

HQ613403.2007 GAAGGGAGCAAGGGCATCAGTGTGCTCAGTTGAAAATCCCTTGTCAACATCTAGGTCTTA 192

KC242785.2007 GAAGGGAGCGAGGGCATCAGTGTGCTCAGTTGAAAATCCCTTGTCAACATCTAGGTCTTA 240

KC242790.2007 GAAGGGAGCGAGGGCATCAGTGTGCTCAGTTGAAAATCCCTTGTCAACATCTAGGTCTTA 240

KU143789.2014 GGAGGGGGCAAGGGCATCAGTGTGCTCAGTTGAAAATCCCTTGTCAACATCTAGGCCTTA 240

KR817168.2014 GGAGGGGGCAAGGGCATCAGTGTGCTCAGTTGAAAATCCCTTGTCAACATCTAGGCCTTA 214

KY426696.2015 GGAGGGGGCAAGGGCATCAGTGTGCTCAGTTGAAAATCCCTTGTCAACATCTAGGCCTTA 240

KR105271.2014 GGAGGGGGCAAGGGCATCAGTGTGCTCAGTTGAAAATCCCTTGTCAACATCTAGGCCTTA 207

KY007522.2016 GGAGGGGGCAAGGGCATCAGTGTGCTCAGTTGAAAATCCCTTGTCAACATCTAGGCCTTA 220

KM034555.2014 GGAGGGGGCAAGGGCATCAGTGTGCTCAGTTGAAAATCCCTTGTCAACATCTAGGCCTTA 232

MH470381.2015 GGAGGGGGCAAGGGCATCAGTGTGCTCAGTTGAAAATCCCTTGTCAACATCTAGGCCTTA 204

MH470382.2015 GGAGGGGGCAAGGGCATCAGTGTGCTCAGTTGAAAATCCCTTGTCAACATCTAGGCCTTA 204

MF102255.2014 GGAGGGGGCAAGGGCATCAGTGTGCTCAGTTGAAAATCCCTTGTCAACATCTAGGCCTTA 217

KJ660348.2014 GGAGGGGGCAAGGGCATCAGTGTGCTCAGTTGAAAATCCCTTGTCAACATCTAGGCCTTA 240

KU143818.2014 GGAGGGGGCAAGGGCATCAGTGTGCTCAGTTGAAAATCCCTTGTCAACATCTAGGCCTTA 240

KT725333.2014 GGAGGGGGCAAGGGCATCAGTGTGCTCAGTTGAAAATCCCTTGTCAACATCTAGGCCTTA 205

KR819004.2014 AAAGGGGGCAAGGGCATCAGTGTGCTCAGTTGAAAATCCCTTGTCAACATCTAGGTCTTA 187

KP271020.2014 AAAGGGGGCAAGGGCATCAGTGTGCTCAGTTGAAAATCCCTTGTCAACATCTAGGTCTTA 187

KM519951.2014 AAAGGGGGCAAGGGCATCAGTGTGCTCAGTTGAAAATCCCTTGTCAACATCTAGGTCTTA 237

MH733488.2018 AAAGGGGGCAAGGGCATCAGTGTGCTCAGTTGAAAATCCCTTGTCAACATCTAGGTCTTA 231

MH733491.2018 AAAGGGGGCAAGGGCATCAGTGTGCTCAGTTGAAAATCCCTTGTCAACATCTAGGTCTTA 226

MH733478.2018 AAAGGGGGCAAGGGCATCAGTGTGCTCAGTTGAAAATCCCTTGTCAACATCTAGGTCTTA 229

MK007330.2018 GAAGGGAGCAAGGGCATCAGTGTGCTCAGTTGAGAATCCCTTGTCAACATCTAGGTCTTA 228

MK007344.2018 GAAGGGAGCAAGGGCATCAGTGTGCTCAGTTGAGAATCCCTTGTCAACATCTAGGTCTTA 222

**** * ****** ******** ******* ************** ***** ****

AY142960.1976 TCACATCACAAGTTCCACCTCAGACTCTGCAGGGTGATCCAACAACCTTAATAGAAACAT 300 -> Fprimer urNP

KC242791.1977 TCACATCACAAGTTCCACCTCAGACTCTGCAGGGTGATCCAACAACCTTAATAGAAACAT 300

AF499101.1976 TCACATCACAAGTTCCACCTCAGACTCTGCAGGGTGATCCAACAACCTTAATAGAAACAT 300

KF113528.2003 TCACATCACAAGTTCCACCTCAGACTCTGCAGGGTGATCCAACAACCCTAATAGAAAAAT 296

KC242800.2002 TCACATCACAAGTTCCACCTCAGACTCTGCAGGGTGATCCAACAACCCTAATAGAAAAAT 300

KY471090.2001 TCACATCACAAGTTCCACCTCAGACTCTGCAGGGTGATCCAACAACCCTAATAGAAAAAT 277

KY471092.2001 TCACATCACAAGTTCCACCTCAGACTCTGCAGGGTGATCCAACAACCCTAATAGAAAAAT 277

MH481611.2017 TCACATCACAAGTTCCACCTCAGACTCTGCAGGGTGATCCAACAACCTTAATAGAAACAT 266

MH613311.2017 TCACATCACAAGTTCCACCTCAGACTCTGCAGGGTGATCCAACAACCTTAATAGAAACAT 245

KC242792.1994 TCACATCACAAGTTCCACCTCAGACTCTGCAGGGTGATCCAACAACCTTAATAGAAACAT 300

KC242793.1996 TCACATCACAAGTTCCACCTCAGACTCTGCAGGGTGATCCAACAACCTTAATAGAAACAT 300

KC242794.1996 TCACATCACAAGTTCCACCTCAGACTCTGCAGGGTGATCCAACAACCTTAATAGAAACAT 300

MH121164.1995 TCACATCACAAGTCCCACCTCAGACTCTGCAGGGTGATCCAACAACCTTAATAGAAACAT 300

AY354458.1995 TCACATCACAAGTCCCACCTCAGACTCTGCAGGGTGATCCAACAACCTTAATAGAAACAT 300

KT762962.1995 TCACATCACAAGTCCCACCTCAGACTCTGCAGGGTGATCCAACAACCTTAATAGAAACAT 300

HQ613402.2008 TCACATCACAGGTTCCACCTCAGGTTCTGCAGGGTGATCCAACAACCTTAATAGAAACAT 219

KC242789.2007 TCACATCACAGGTTCCACCTCAGGCTCTGCAGGGTGATCCAACAACCTTAATAGAAACAT 300

HQ613403.2007 TCACATCACAGGTTCCACCTCAGGCTCTGCAGGGTGATCCAACAACCTTAATAGAAACAT 252

KC242785.2007 TCACATCACAGGTTCCACCTCAGGCTCTGCAGGGTGATCCAACAACCTTAATAGAAACAT 300

KC242790.2007 TCACATCACAGGTTCCACCTCAGGCTCTGCAGGGTGATCCAACAACCTTAATAGAAACAT 300

KU143789.2014 TCACATCACAAGTTCCGCCTTAAACTCTGCAGGGTGATCCAACAACCTTAATAGCAACAT 300

KR817168.2014 TCACATCACAAGTTCCGCCTTAAACTCTGCAGGGTGATCCAACAACCTTAATAGCAACAT 274

KY426696.2015 TCACATCACAAGTTCCGCCTTAAACTCTGCAGGGTGATCCAACAACCTTAATAGCAACAT 300

KR105271.2014 TCACATCACAAGTTCCGCCTTAAACTCTGCAGGGTGATCCAACAACCTTAATAGCAACAT 267

KY007522.2016 TCACATCACAAGTTCCGCCTTAAACTCTGCAGGGTGATCCGACAACCTTAATAGCAACAT 280

KM034555.2014 TCACATCACAAGTTCCGCCTTAAACTCTGCAGGGTGATCCAACAACCTTAATAGCAACAT 292

MH470381.2015 TCACATCACAAGTTCCGCCTTAAACTCTGCAGGGTGATCCAACAACCTTAATAGCAACAT 264

MH470382.2015 TCACATCACAAGTTCCGCCTTAAACTCTGCAGGGTGATCCAACAACCTTAATAGCAACAT 264

MF102255.2014 TCACATCACAAGTTCCGCCTTAAACTCTGCAGGGTGATCCAACAACCTTAATAGCAACAT 277

KJ660348.2014 TCACATCACAAGTTCCGCCTTAAACTCTGCAGGGTGATCCAACAACCTTAATAGCAACAT 300

KU143818.2014 TCACATCACAAGTTCCGCCTTAAACTCTGCAGGGTGATCCAACAACCTTAATAGCAACAT 300

KT725333.2014 TCACATCACAAGTTCCGCCTTAAACTCTGCAGGGTGATCCAACAACCTTAATAGCAACAT 265

KR819004.2014 TCACATCACAAGTTCCACCTCAGACTCTGCAGGGTGATCCAACAACCTTAATAGAAACAT 247

KP271020.2014 TCACATCACAAGTTCCACCTCAGACTCTGCAGGGTGATCCAACAACCTTAATAGAAACAT 247

KM519951.2014 TCACATCACAAGTTCCACCTCAGACTCTGCAGGGTGATCCAACAACCTTAATAGAAACAT 297

MH733488.2018 TCACATCACAAGTTCCACCTCAGACTCTGCAGGGTGATCCAACAACCTTAATAGAAACAT 291

MH733491.2018 TCACATCACAAGTTCCACCTCAGACTCTGCAGGGTGATCCAACAACCTTAATAGAAACAT 286

MH733478.2018 TCACATCACAAGTTCCACCTCAGACTCTGCAGGGTGATCCAACAACCTTAATAGAAACAT 289

MK007330.2018 TCACACCACAAGTTCCACCTCAGACTCTGCAGGGTGATCCAACAACCTTAATAGAAACAT 288

MK007344.2018 TCACACCACAAGTTCCACCTCAGACTCTGCAGGGTGATCCAACAACCTTAATAGAAACAT 282

***** **** ** ** *** * *************** ****** ****** ** **

AY142960.1976 TATTGTTAAAGGACAGCATTAGTTCACAGTCAAACAAGCAAGATTGAGAATTAACCTTGG 360

KC242791.1977 TATTGTTAAAGGACAGCATTAGTTCACAGTCAAACAAGCAAGATTGAGAATTAACCTTGG 360

AF499101.1976 TATTGTTAAAGGACAGCATTAGTTCACAGTCAAACAAGCAAGATTGAGAATTAACCTTGG 360

KF113528.2003 TATTGTTAACGGACAGCATTAGTTCACAGTCAAACAAGCAAGATTGAGAATTAACCTTGA 356

KC242800.2002 TATTGTTAACGGACAGCATTAGTTCACAGTCAAACAAGCAAGATTGAGAATTAACCTTGA 360

KY471090.2001 TATTGTTAACGGACAGCATTAGTTCACAGTCAAACAAGCAAGATTGAGAATTAACCTTGA 337

KY471092.2001 TATTGTTAACGGACAGCATTAGTTCACAGTCAAACAAGCAAGATTGAGAATTAACCTTGA 337

MH481611.2017 TATTGTTAAAGGACAGCATTAGTTCACAGTCAAACAAGCAAGATTGAGAATTAACTTTGG 326

MH613311.2017 TATTGTTAAAGGACAGCATTAGTTCACAGTCAAACAAGCAAGATTGAGAATTAACTTTGG 305

KC242792.1994 TATTGTTAAAGGACAGCATTAGTTCACAGTCAAACAAGCAAGATTGAGAATTAACCTTGG 360

KC242793.1996 TATTGTTAAAGGACAGCATTAGTTCACAGTCAAACAAGCAAGATTGAGAATTAACCTTGG 360

KC242794.1996 TATTGTTAAAGGACAGCATTAGTTCACAGTCAAACAAGCAAGATTGAGAATTAACCTTGG 360

MH121164.1995 TATTGTTAAAGGACAGCATTAGTTCACAGTCAAACAAGCAAGATTGAGAATTAACCTTGG 360

AY354458.1995 TATTGTTAAAGGACAGCATTAGTTCACAGTCAAACAAGCAAGATTGAGAATTAACCTTGG 360

KT762962.1995 TATTGTTAAAGGACAGCATTAGTTCACAGTCAAACAAGCAAGATTGAGAATTAACCTTGG 360

HQ613402.2008 TATTGTTAAAGGACAACATTAGGTCACAGTCAAACAAGCAAGATTGAGAATTAACCTTGA 279

KC242789.2007 TATTGTTAAAGGACAACATTAGGTCACAGTCAAACAAGCAAGATTGAGAATTAACCTTGA 360

HQ613403.2007 TATTGTTAAAGGACAACATTAGGTCACAGTCAAACAAGCAAGATTGAGAATTAACCTTGA 312

KC242785.2007 TATTGTTAAAGGACAACATTAGGTCACAGTCAAACAAGCAAGATTGAGAATTAACCTTGA 360

KC242790.2007 TATTGTTAAAGGACAACATTAGGTCACAGTCAAACAAGCAAGATTGAGAATTAACCTTGA 360

KU143789.2014 TATTGTTAAAGGACAGCATTAGTTCACAGTCAAACAAGCAAGATTGAGAATTAACTTTGA 360

KR817168.2014 TATTGTTAAAGGACAGCATTAGTTCACAGTCAAACAAGCAAGATTGAGAATTAACTTTGA 334

KY426696.2015 TATTGTTAAAGGACAGCATTAGTTCACAGTCAAACAAGCAAGATTGAGAATTAACTTTGA 360

KR105271.2014 TATTGTTAAAGGACAGCATTAGTTCACAGTCAAACAAGCAAGATTGAGAATTAACTTTGA 327

KY007522.2016 TATTGTTAAAGGACAGCATTAGTTCACAGTCAAACAAGCAAGATTGAGAATTAACTTTGA 340

KM034555.2014 TATTGTTAAAGGACAGCATTAGTTCACAGTCAAACAAGCAAGATTGAGAATTAACTTTGA 352

MH470381.2015 TATTGTTAAAGGACAGCATTAGTTCACAGTCAAACAAGCAAGATTGGGAATTAACTTTGA 324

MH470382.2015 TATTGTTAAAGGACAGCATTAGTTCACAGTCAAACAAGCAAGATTGAGAATTAACTTTGA 324

MF102255.2014 TATTGTTAAAGGACAGCATTAGTTCACAGTCAAACAAGCAAGATTGAGAATTAACTTTGA 337

KJ660348.2014 TATTGTTAAAGGACAGCATTAGTTCACAGTCAAACAAGCAAGATTGAGAATTAACTTTGA 360

KU143818.2014 TATTGTTAAAGGACAGCATTAGTTCACAGTCAAACAAGCAAGATTGAGAATTAACTTTGA 360

KT725333.2014 TATTGTTAAAGGACAGCATTAGTTCACAGTCAAACAAGCAAGATTGAGAATTAACCTTGA 325

KR819004.2014 TATTGTTAAAGGACAGCATTAGTTCACAGTCAAACAAGCAAGATTGAGAATTAACCTTGG 307

KP271020.2014 TATTGTTAAAGGACAGCATTAGTTCACAGTCAAACAAGCAAGATTGAGAATTAACCTTGG 307

KM519951.2014 TATTGTTAAAGGACAGCATTAGTTCACAGTCAAACAAGCAAGATTGAGAATTAACCTTGG 357

MH733488.2018 TATTGTTAAAGGACAGCATTAGTTCACAGTCAAACAAGCAAGATTGAGAATTAACCTTGG 351

MH733491.2018 TATTGTTAAAGGACAGCATTAGTTCACAGTCAAACAAGCAAGATTGAGAATTAACCTTGG 346

MH733478.2018 TATTGTTAAAGGACAGCATTAGTTCACAGTCAAACAAGCAAGATTGAGAATTAACCTTGG 349

MK007330.2018 TATTGTTAAAGGACAGCATTAGTTCACAGTCAAACAAGTAAGATTGAGAATTAACCTTGG 348

MK007344.2018 TATTGTTAAAGGACAGCATTAGTTCACAGTCAAACAAGTAAGATTGAGAATTAACCTTGG 342

********* ***** ****** *************** ******* ******** ***

AY142960.1976 TTTTGAACTTGAACACTTAGGGGATTGAAGATTCAACAACCCTAAAGCTTGGGGTAAAAC 420

KC242791.1977 TTTTGAACTTGAACACTTAGGGGATTGAAGATTCAACAACCCTAAAGCTTGGGGTAAAAC 420

AF499101.1976 TTTTGAACTTGAACACTTAGGGGATTGAAGATTCAACAACCCTAAAGCTTGGGGTAAAAC 420

KF113528.2003 TTTTGAACTTCAACACCTAGAGGATTGGAGATTCAACAACCCTAAAACTTGGGGTAAAAC 416

KC242800.2002 TTTTGAACTTCAACACCTAGAGGATTGGAGATTCAACAACCCTAAAACTTGGGGTAAAAC 420

KY471090.2001 TTTTGAACTTCAACACCTAGAGGATTGGAGATTCAACAACCCTAAAACTTGGGGTAAAAC 397

KY471092.2001 TTTTGAACTTCAACACCTAGAGGATTGGAGATTCAACAACCCTAAAACTTGGGGTAAAAC 397

MH481611.2017 TTTTGAACTTGAACACTTAGAGGATTGGAGATTCAACAACCCTAAAGCTTGGGGTAAAAC 386

MH613311.2017 TTTTGAACTTGAACACTTAGAGGATTGGAGATTCAACAACCCTAAAGCTTGGGGTAAAAC 365

KC242792.1994 TTTTGAACTTGAACACTTAGAGGATTGGAGATTCAACAACCCTAAAGCTTGGGGTAAAGC 420

KC242793.1996 TTTTGAACTTGAACACTTAGAGGATTGGAGATTCAACAACCCTAAAGCTTGGGGTAAAGC 420

KC242794.1996 TTTTGAACTTGAACACTTAGAGGATTGGAGATTCAACAACCCTAAAGCTTGGGGTAAAGC 420

MH121164.1995 TTTTGAACTTGAATACTTAGAGGATTGGAGATTCAACAACCCTAAAGCTTGGGGTAAAAC 420

AY354458.1995 TTTTGAACTTGAATACTTAGAGGATTGGAGATTCAACAACCCTAAAGCTTGGGGTAAAAC 420

KT762962.1995 TTTTGAACTTGAATACTTAGAGGATTGGAGATTCAACAACCCTAAAGCTTGGGGTAAAAC 420

HQ613402.2008 TTTTGAACTTGAACACCTAGAGGATTGGAGATTCAACAACCCTAAAGCTTGGGGTAAAAC 339

KC242789.2007 TTTTGAACTTGAACACCTAGAGGATTGGAGATTCAACAACCCTAAAGCTTGGGGTAAAAC 420

HQ613403.2007 TTTTGAACTTGAACACCTAGAGGATTGGAGATTCAACAACCCTAAAGCTTGGGGTAAAAC 372

KC242785.2007 TTTTGAACTTGAACACCTAGAGGATTGGAGATTCAACAACCCTAAAGCTTGGGGTAAAAC 420

KC242790.2007 TTTTGAACTTGAACACCTAGAGGATTGGAGATTCAACAACCCTAAAGCTTGGGGTAAAAC 420

KU143789.2014 TTTTGAACCTGAACACCCAGAGGACTGGAGACTCAACAACCCTAAAGCCTGGGGTAAAAC 420

KR817168.2014 TTTTGAACCTGAACACCCAGAGGACTGGAGACTCAACAACCCTAAAGCCTGGGGTAAAAC 394

KY426696.2015 TTTTGAACCTGAACACCCAGAGGACTGGAGACTCAACAACCCTAAAGCCTGGGGTAAAAC 420

KR105271.2014 TTTTGAACCTGAACACCCAGAGGACTGGAGACTCAACAACCCTAAAGCCTGGGGTAAAAC 387

KY007522.2016 TTTTGAACCTGAACACCCAGAGGACTGGAGACTCAACAACCCTAAAGCCTGGGGTAAAAC 400

KM034555.2014 TTTTGAACCTGAACACCCAGAGGACTGGAGACTCAACAACCCTAAAGCCTGGGGTAAAAC 412

MH470381.2015 TTTTGAACCTGAACACCCAGAGGACTGGAGACTCAACAACCCTAAAGCCTGGGGTAAAAC 384

MH470382.2015 TTTTGAACCTGAACACCCAGAGGACTGGAGACTCAACAACCCTAAAGCCTGGGGTAAAAC 384

MF102255.2014 TTTTGAACCTGAACACCCAGAGGACTGGAGACTCAACAACCCTAAAGCCTGGGGTAAAAC 397

KJ660348.2014 TTTTGAACCTGAACACCCAGAGGACTGGAGACTCAACAACCCTAAAGCCTGGGGTAAAAC 420

KU143818.2014 TTTTGAACCTGAACACCCAGAGGACTGGAGACTCAACAACCCTAAAGCCTGGGGTAAAAC 420

KT725333.2014 TTTTGAACCTGAACACCCAGAGGACTGGAGACTCAACAACCCTAAAGCCTGGGGTAAAAC 385

KR819004.2014 TTTTGAACTTGAACACTTAGAGGATTGGAGATTCAACAACCCTAAAGCTTGGAGTAAAAC 367

KP271020.2014 TTTTGAACTTGAACACTTAGAGGATTGGAGATTCAACAACCCTAAAGCTTGGAGTAAAAC 367

KM519951.2014 TTTTGAACTTGAACACTTAGAGGATTGGAGATTCAACAACCCTAAAGCTTGGAGTAAAAC 417

MH733488.2018 TTTTGAACTTGAACACTTAGAGGATTGGAGATTCAACAACCCTAAAGCTTGGAGTAAAAC 411

MH733491.2018 TTTTGAACTTGAACACTTAGAGGATTGGAGATTCAACAACCCTAAAGCTTGGAGTAAAAC 406

MH733478.2018 TTTTGAACTTGAACACTTAGAGGATTGGAGATTCAACAACCCTAAAGCTTGGAGTAAAAC 409

MK007330.2018 TTTTGAACTTGAACACTTAGAGGATTGGAGATTCAACAACCCTAAAGCTTGGGGTAAAAC 408

MK007344.2018 TTTTGAACTTGAACACTTAGAGGATTGGAGATTCAACAACCCTAAAGCTTGGGGTAAAAC 402

******** * ** ** ** *** ** *** ************** * *** ***** *

AY142960.1976 ATTGGAAATAGTTAAAAGACAAATTGCTCGGAATCACAAAATTCCGAGTATGGATTCTCG 480 start NP

KC242791.1977 ATTGGAAATAGTTAAAAGACAAATTGCTCGGAATCACAAAATTCCGAGTATGGATTCTCG 480

AF499101.1976 ATTGGAAATAGTTAAAAGACAAATTGCTCGGAATCACAAAATTCCGAGTATGGATTCTCG 480

KF113528.2003 ATTGGAAATAGTTGAAAGACAAATTGCTCGGAATCACAAAATTCCGAGTATGGATTCTCG 476

KC242800.2002 ATTGGAAATAGTTGAAAGACAAATTGCTCGGAATCACAAAATTCCGAGTATGGATTCTCG 480

KY471090.2001 ATTGGAAATAGTTGAAAGACAAATTGCTCGGAATCACAAAATTCCGAGTATGGATTCTCG 457

KY471092.2001 ATTGGAAATAGTTGAAAGACAAATTGCTCGGAATCACAAAATTCCGAGTATGGATTCTCG 457

MH481611.2017 ATTGGAAATAGTTAAAAGACAAATTGCTCGGAATCACAAAATTCCGAGTATGGATTCTCG 446

MH613311.2017 ATTGGAAATAGTTAAAAGACAAATTGCTCGGAATCACAAAATTCCGAGTATGGATTCTCG 425

KC242792.1994 ATTGGAAATAGTTGAAAGACAAATTGCTCGGAATCACAAAATTCCGAGTATGGATTCTCG 480

KC242793.1996 ATTGGAAATAGTTGAAAGACAAATTGCTCGGAATCACAAAATTCCGAGTATGGATTCTCG 480

KC242794.1996 ATTGGAAATAGTTGAAAGACAAATTGCTCGGAATCACAAAATTCCGAGTATGGATTCTCG 480

MH121164.1995 ATTGGAAATAGTTAAAAGACAAATTGCTCGGAATCACAACATTCCGAGTATGGATTCTCG 480

AY354458.1995 ATTGGAAATAGTTAAAAGACAAATTGCTCGGAATCACAACATTCCGAGTATGGATTCTCG 480

KT762962.1995 ATTGGAAATAGTTAAAAGACAAATTGCTCGGAATCACAACATTCCGAGTATGGATTCTCG 480

HQ613402.2008 ATTGGAAATAGTTAAAAGACAAATTGCTCGGAATCACAAAATTCCGAGTATGGATTCTCG 399

KC242789.2007 ATTGGAAATAGTTAAAAGACAAATTGCTCGGAATCACAAAATTCCGAGTATGGATTCTCG 480

HQ613403.2007 ATTGGAAATAGTTAAAAGACAAATTGCTCGGAATCACAAAATTCCGAGTATGGATTCTCG 432

KC242785.2007 ATTGGAAATAGTTAAAAGACAAATTGCTCGGAATCACAAAATTCCGAGTATGGATTCTCG 480

KC242790.2007 ATTGGAAATAGTTAAAAGACAAATTGCTCGGAATCACAAAATTCCGAGTATGGATTCTCG 480

KU143789.2014 ATTAGAAATAGTTTAAAGACAAATTGCTCGGAATCACAAAATTCCGAGTATGGATTCTCG 480

KR817168.2014 ATTAGAAATAGTTTAAAGACAAATTGCTCGGAATCACAAAATTCCGAGTATGGATTCTCG 454

KY426696.2015 ATTAGAAATAGTTTAAAGACAAATTGCTCGGAATCACAAAATCCCGAGTATGGATTCTCG 480

KR105271.2014 ATTAGAAATAGTTTAAAGACAAATTGCTCGGAATCACAAAATTCCGAGTATGGATTCTCG 447

KY007522.2016 ATTAGAAATAGTTTAAAGACAAATTGCTCGGAATCACAAAATTCCGAGTATGGATTCTCG 460

KM034555.2014 ATTAGAAATAGTTTAAAGACAAATTGCTCGGAATCACAAAATTCCGAGTATGGATTCTCG 472

MH470381.2015 ATTAGAAATAGTTTAAAGACAAATTGCTCGGAATCACAAAATTCCGAGTATGGATTCTCG 444

MH470382.2015 ATTAGAAATAGTTTAAAGACAAATTGCTCGGAATCACAAAATTCCGAGTATGGATTCTCG 444

MF102255.2014 ATTAGAAATAGTTTAAAGACAAATTGCTCGGAATCACAAAATTCCGAGTATGGATTCTCG 457

KJ660348.2014 ATTAGAAATAGTTTAAAGACAAATTGCTCGGAATCACAAAATTCCGAGTATGGATTCTCG 480

KU143818.2014 ATTAGAAATAGTTTAAAGACAAATTGCTCGGAATCACAAAATTCCGAGTATGGATTCTCG 480

KT725333.2014 ATTAGAAATAGTTTAAAGACAAATTGCTCGGAATCACAAAATTCCGAGTATGGATTCTCG 445

KR819004.2014 ATTGGAAATAGTTAAAAGACAAATTGCTCGGAATCACAAAATCCCGAGTATGAATTCTCG 427

KP271020.2014 ATTGGAAATAGTTAAAAGACAAATTGCTCGGAATCACAAAATCCCGAGTATGAATTCTCG 427

KM519951.2014 ATTGGAAATAGTTAAAAGACAAATTGCTCGGAATCACAAAATCCCGAGTATGAATTCTCG 477

MH733488.2018 ATTGGAAATAGTTAAAAGACAAATTGCTCGGAATCACAAAATCCCGAGTATGGATTCTCG 471

MH733491.2018 ATTGGAAATAGTTAAAAGACAAATTGCTCGGAATCACAAAATCCCGAGTATGGATTCTCG 466

MH733478.2018 ATTGGAAATAGTTAAAAGACAAATTGCTCGGAATCACAAAATCCCGAGTATGGATTCTCG 469

MK007330.2018 ATTGGAAATAGTTAAAAGACAAATAGCTCGGAATCACAAAATTCCGAGTATGGATTCTCG 468

MK007344.2018 ATTGGAAATAGTTAAAAGACAAATAGCTCGGAATCACAAAATTCCGAGTATGGATTCTCG 462

*** ********* ********** ************** ** ********* *******

AY142960.1976 TCCTCAGAAAATCTGGATGGCGCCGAGTCTCACTGAATCTGACATGGATTACCACAAGAT 540 EBOV-miR-T3-5p/T4-5p

KC242791.1977 TCCTCAGAAAATCTGGATGGCGCCGAGTCTCACTGAATCTGACATGGATTACCACAAGAT 540

AF499101.1976 TCCTCAGAAAATCTGGATGGCGCCGAGTCTCACTGAATCTGACATGGATTACCACAAGAT 540

KF113528.2003 TCCTCAGAAAGTCTGGATGACGCCGAGTCTTACTGAATCTGACATGGATTACCACAAGAT 536

KC242800.2002 TCCTCAGAAAGTCTGGATGACGCCGAGTCTTACTGAATCTGACATGGATTACCACAAGAT 540

KY471090.2001 TCCTCAGAAAGTCTGGATGACGCCGAGTCTTACTGAATCTGACATGGATTACCACAAGAT 517

KY471092.2001 TCCTCAGAAAGTCTGGATGACGCCGAGTCTTACTGAATCTGACATGGATTACCACAAGAT 517

MH481611.2017 TCCTCAGAAAGTCTGGATGACACCGAGTCTCACTGAATCTGACATGGATTACCACAAGAT 506

MH613311.2017 TCCTCAGAAAGTCTGGATGACACCGAGTCTCACTGAATCTGACATGGATTACCACAAGAT 485

KC242792.1994 TCCTCAGAAAGTCTGGATGACGCCGAGTCTCACTGAATCTGACATGGATTACCACAAGAT 540

KC242793.1996 TCCTCAGAAAGTCTGGATGACGCCGAGTCTCACTGAATCTGACATGGATTACCACAAGAT 540

KC242794.1996 TCCTCAGAAAGTCTGGATGACGCCGAGTCTCACTGAATCTGACATGGATTACCACAAGAT 540

MH121164.1995 TCCTCAGAAAGTCTGGATGACGCCGAGTCTCACTGAATCTGACATGGATTACCACAAGAT 540

AY354458.1995 TCCTCAGAAAGTCTGGATGACGCCGAGTCTCACTGAATCTGACATGGATTACCACAAGAT 540

KT762962.1995 TCCTCAGAAAGTCTGGATGACGCCGAGTCTCACTGAATCTGACATGGATTACCACAAGAT 540

HQ613402.2008 TCCTCAGAAAGTCTGGATGACGCCGAGTCTCACTGAATCTGACATGGATTACCACAAGAT 459

KC242789.2007 TCCTCAGAAAGTCTGGATGACGCCGAGTCTCACTGAATCTGACATGGATTACCACAAGAT 540

HQ613403.2007 TCCTCAGAAAGTCTGGATGACGCCGAGTCTCACTGAATCTGACATGGATTACCACAAGAT 492

KC242785.2007 TCCTCAGAAAGTCTGGATGACGCCGAGTCTCACTGAATCTGACATGGATTACCACAAGAT 540

KC242790.2007 TCCTCAGAAAGTCTGGATGACGCCGAGTCTCACTGAATCTGACATGGATTACCACAAGAT 540

KU143789.2014 TCCTCAGAAAGTCTGGATGACGCCGAGTCTCACTGAATCTGACATGGATTACCACAAGAT 540

KR817168.2014 TCCTCAGAAAGTCTGGATGACGCCGAGTCTCACTGAATCTGACATGGATTACCACAAGAT 514

KY426696.2015 TCCTCAGAAAGTCTGGATGACGCCGAGTCTCACTGAATCTGACATGGATTACCACAAGAT 540

KR105271.2014 TCCTCAGAAAGTCTGGATGACGCCGAGTCTCACTGAATCTGACATGGATTACCACAAGAT 507

KY007522.2016 TCCTCAGAGAGTCTGGATGACGCCGAGTCTCACTGAATCTGACATGGATTACCACAAGAT 520

KM034555.2014 TCCTCAGAAAGTCTGGATGACGCCGAGTCTCACTGAATCTGACATGGATTACCACAAGAT 532

MH470381.2015 TCCTCAGAAAGTCTGGATGACGCCGAGTCTCACTGAATCTGACATGGATTACCACAAGAT 504

MH470382.2015 TCCTCAGAAAGTCTGGATGACGCCGAGTCTCACTGAATCTGACATGGATTACCACAAGAT 504

MF102255.2014 TCCTCAGAAAGTCTGGATGACGCCGAGTCTCACTGAATCTGACATGGATTACCACAAGAT 517

KJ660348.2014 TCCTCAGAAAGTCTGGATGACGCCGAGTCTCACTGAATCTGACATGGATTACCACAAGAT 540

KU143818.2014 TCCTCAGAAAGTCTGGATGACGCCGAGTCTCACTGAATCTGACATGGATTACCACAAGAT 540

KT725333.2014 TCCTCAGAAAGTCTGGATGACGCCGAGTCTCACTGAATCTGACATGGATTACCACAAGAT 505

KR819004.2014 TCCTCAGAAAGTCTGGATGACGCCGAGTCTCACTGAATCTGACATGGATTACCACAAGAT 487

KP271020.2014 TCCTCAGAAAGTCTGGATGACGCCGAGTCTCACTGAATCTGACATGGATTACCACAAGAT 487

KM519951.2014 TCCTCAGAAAGTCTGGATGACGCCGAGTCTCACTGAATCTGACATGGATTACCACAAGAT 537

MH733488.2018 TCCTCAGAAAGTCTGGATGACGCCGAGTCTCACTGAATCTGACATGGATTACCACAAGAT 531

MH733491.2018 TCCTCAGAAAGTCTGGATGACGCCGAGTCTCACTGAATCTGACATGGATTACCACAAGAT 526

MH733478.2018 TCCTCAGAAAGTCTGGATGACGCCGAGTCTCACTGAATCTGACATGGATTACCACAAGAT 529

MK007330.2018 TCCTCAGAAAGTTTGGATGACGCCGAATCTCACTGAATCTGACATGGATTACCACAAGAT 528

MK007344.2018 TCCTCAGAAAGTTTGGATGACGCCGAATCTCACTGAATCTGACATGGATTACCACAAGAT 522

******** * * ****** * **** *** *****************************

AY142960.1976 CTTGACAGCAGGTCTGTCCGTTCAACAGGGGATTGTTCGGCAAAGAGTCATCCCAGTGTA 600 EBOV-miR-T3-3p

KC242791.1977 CTTGACAGCAGGTCTGTCCGTTCAACAGGGGATTGTTCGGCAAAGAGTCATCCCAGTGTA 600 <- Rprimer urNP

AF499101.1976 CTTGACAGCAGGTCTGTCCGTTCAACAGGGGATTGTTCGGCAAAGAGTCATCCCAGTGTA 600

KF113528.2003 CTTGACAGCAGGTCTGTCCGTTCAACAGGGGATTGTTCGGCAAAGAGTCATCCCAGTGTA 596

KC242800.2002 CTTGACAGCAGGTCTGTCCGTTCAACAGGGGATTGTTCGGCAAAGAGTCATCCCAGTGTA 600

KY471090.2001 CTTGACAGCAGGTCTGTCCGTTCAACAGGGGATTGTTCGGCAAAGAGTCATCCCAGTGTA 577

KY471092.2001 CTTGACAGCAGGTCTGTCCGTTCAACAGGGGATTGTTCGGCAAAGAGTCATCCCAGTGTA 577

MH481611.2017 CTTGACGGCAGGTCTGTCTGTTCAACAGGGGATTGTTCGGCAAAGAGTCATCCCAGTGTA 566

MH613311.2017 CTTGACGGCAGGTCTGTCTGTTCAACAGGGGATTGTTCGGCAAAGAGTCATCCCAGTGTA 545

KC242792.1994 CTTGACAGCAGGTCTGTCCGTTCAACAGGGGATTGTTCGGCAAAGAGTCATCCCAGTGTA 600

KC242793.1996 CTTGACAGCAGGTCTGTCCGTTCAACAGGGGATTGTTCGGCAAAGAGTCATCCCAGTGTA 600

KC242794.1996 CTTGACAGCAGGTCTGTCCGTTCAACAGGGGATTGTTCGGCAAAGAGTCATCCCAGTGTA 600

MH121164.1995 CTTGACAGCAGGTCTGTCCGTTCAACAGGGGATTGTTCGGCAAAGAGTCATCCCAGTGTA 600

AY354458.1995 CTTGACAGCAGGTCTGTCCGTTCAACAGGGGATTGTTCGGCAAAGAGTCATCCCAGTGTA 600

KT762962.1995 CTTGACAGCAGGTCTGTCCGTTCAACAGGGGATTGTTCGGCAAAGAGTCATCCCAGTGTA 600

HQ613402.2008 CTTAACAGCAGGTCTGTCCGTTCAACAGGGGATTGTTCGGCAAAGAGTCATCCAAGTGTA 519

KC242789.2007 CTTAACAGCAGGTCTGTCCGTTCAACAGGGGATTGTTCGGCAAAGAGTCATCCAAGTGTA 600

HQ613403.2007 CTTAACAGCAGGTCTGTCCGTTCAACAGGGGATTGTTCGGCAAAGAGTCATCCAAGTGTA 552

KC242785.2007 CTTAACAGCAGGTCTGTCCGTTCAACAGGGGATTGTTCGGCAAAGAGTCATCCAAGTGTA 600

KC242790.2007 CTTAACAGCAGGTCTGTCCGTTCAACAGGGGATTGTTCGGCAAAGAGTCATCCAAGTGTA 600

KU143789.2014 CTTGACAGCAGGTCTGTCCGTTCAACAGGGGATTGTTCGGCAAAGAGTCATCCCAGTGTA 600

KR817168.2014 CTTGACAGCAGGTCTGTCCGTTCAACAGGGGATTGTTCGGCAAAGAGTCATCCCAGTGTA 574

KY426696.2015 CTTGACAGCAGGTCTGTCCGTTCAACAGGGGATTGTTCGGCAAAGAGTCATCCCAGTGTA 600

KR105271.2014 CTTGACAGCAGGTCTGTCCGTTCAACAGGGGATTGTTCGGCAAAGAGTCATCCCAGTGTA 567

KY007522.2016 CTTGACAGCAGGTCTGTCCGTTCAACAGGGGATTGTTCGGCAAAGAGTCATCCCAGTGTA 580

KM034555.2014 CTTGACAGCAGGTCTGTCCGTTCAACAGGGGATTGTTCGGCAAAGAGTCATCCCAGTGTA 592

MH470381.2015 CTTGACAGCAGGTCTGTCCGTTCAACAGGGGATTGTTCGGCAAAGAGTCATCCCAGTGTA 564

MH470382.2015 CTTGACAGCAGGTCTGTCCGTTCAACAGGGGATTGTTCGGCAAAGAGTCATCCCAGTGTA 564

MF102255.2014 CTTGACAGCAGGTCTGTCCGTTCAACAGGGGATTGTTCGGCAAAGAGTCATCCCAGTGTA 577

KJ660348.2014 CTTGACAGCAGGTCTGTCCGTTCAACAGGGGATTGTTCGGCAAAGAGTCATCCCAGTGTA 600

KU143818.2014 CTTGACAGCAGGTCTGTCCGTTCAACAGGGGATTGTTCGGCAAAGAGTCATCCCAGTGTA 600

KT725333.2014 CTTGACAGCAGGTCTGTCCGTTCAACAGGGGATTGTTCGGCAAAGAGTCATCCCAGTGTA 565

KR819004.2014 CCTGACAGCAGGTCTGTCCGTTCAACAGGGGATTGTTCGGCAAAGAGTCATCCCAGTGTA 547

KP271020.2014 CCTGACAGCAGGTCTGTCCGTTCAACAGGGGATTGTTCGGCAAAGAGTCATCCCAGTGTA 547

KM519951.2014 CCTGACAGCAGGTCTGTCCGTTCAACAGGGGATTGTTCGGCAAAGAGTCATCCCAGTGTA 597

MH733488.2018 CCTGACAGCAGGTCTGTCCGTTCAACAGGGGATTGTTCGGCAAAGAGTCATCCCAGTGTA 591

MH733491.2018 CCTGACAGCAGGTCTGTCCGTTCAACAGGGGATTGTTCGGCAAAGAGTCATCCCAGTGTA 586

MH733478.2018 CCTGACAGCAGGTCTGTCCGTTCAACAGGGGATTGTTCGGCAAAGAGTCATCCCAGTGTA 589

MK007330.2018 CTTGACGGCAGGTCTGTCTGTTCAACAGGGGATTGTTCGGCAAAGAGTCATCCCAGTGTA 588

MK007344.2018 CTTGACGGCAGGTCTGTCTGTTCAACAGGGGATTGTTCGGCAAAGAGTCATCCCAGTGTA 582

* * ** *********** ********************************** ******

AY142960.1976 TCAAGTAAACAATCTTGAAGAAATTTGCCAACTTATCATACAGGCCTTTGAAGCAGGTGT 660

KC242791.1977 TCAAGTAAACAATCTTGAAGAAATTTGCCAACTTATCATACAGGCCTTTGAAGCAGGTGT 660

AF499101.1976 TCAAGTAAACAATCTTGAAGAAATTTGCCAACTTATCATACAGGCCTTTGAAGCAGGTGT 660

KF113528.2003 TCAAGTAAACAATCTTGAGGAAATTTGCCAACTTATCATACAGGCCTTTGAAGCAGGTGT 656

KC242800.2002 TCAAGTAAACAATCTTGAGGAAATTTGCCAACTTATCATACAGGCCTTTGAAGCAGGTGT 660

KY471090.2001 TCAAGTAAACAATCTTGAGGAAATTTGCCAACTTATCATACAGGCCTTTGAAGCAGGTGT 637

KY471092.2001 TCAAGTAAACAATCTTGAGGAAATTTGCCAACTTATCATACAGGCCTTTGAAGCAGGTGT 637

MH481611.2017 TCAAGTAAACAATCTTGAGGAAATTTGCCAACTTATCATACAGGCCTTTGAAGCAGGTGT 626

MH613311.2017 TCAAGTAAACAATCTTGAGGAAATTTGCCAACTTATCATACAGGCCTTTGAAGCAGGTGT 605

KC242792.1994 TCAAGTAAACAATCTTGAGGAGATTTGCCAACTTATCATACAGGCCTTTGAAGCAGGTGT 660

KC242793.1996 TCAAGTAAACAATCTTGAGGAGATTTGCCAACTTATCATACAGGCCTTTGAAGCAGGTGT 660

KC242794.1996 TCAAGTAAACAATCTTGAGGAGATTTGCCAACTTATCATACAGGCCTTTGAAGCAGGTGT 660

MH121164.1995 TCAAGTAAACAATCTTGAGGAGATTTGCCAACTTATCATACAGGCCTTTGAAGCAGGTGT 660

AY354458.1995 TCAAGTAAACAATCTTGAGGAGATTTGCCAACTTATCATACAGGCCTTTGAAGCAGGTGT 660

KT762962.1995 TCAAGTAAACAATCTTGAGGAGATTTGCCAACTTATCATACAGGCCTTTGAAGCAGGTGT 660

HQ613402.2008 TCAAGTAAACAATCTTGAGGAAATTTGCCAACTTATCATACAGGCCTTTGAAGCAGGTGT 579

KC242789.2007 TCAAGTAAACAATCTTGAGGAAATTTGCCAACTTATCATACAGGCCTTTGAAGCAGGTGT 660

HQ613403.2007 TCAAGTAAACAATCTTGAGGAAATTTGCCAACTTATCATACAGGCCTTTGAAGCAGGTGT 612

KC242785.2007 TCAAGTAAACAATCTTGAGGAAATTTGCCAACTTATCATACAGGCCTTTGAAGCAGGTGT 660

KC242790.2007 TCAAGTAAACAATCTTGAGGAAATTTGCCAACTTATCATACAGGCCTTTGAAGCAGGTGT 660

KU143789.2014 TCAAGTAAACAATCTTGAGGAAATTTGCCAACTTATCATACAGGCCTTTGAAGCTGGTGT 660

KR817168.2014 TCAAGTAAACAATCTTGAGGAAATTTGCCAACTTATCATACAGGCCTTTGAAGCTGGTGT 634

KY426696.2015 TCAAGTAAACAATCTTGAGGAAATTTGCCAACTTATCATACAGGCCTTTGAAGCTGGTGT 660

KR105271.2014 TCAAGTAAACAATCTTGAGGAAATTTGCCAACTTATCATACAGGCCTTTGAAGCTGGTGT 627

KY007522.2016 TCAAGTAAACAATCTTGAGGAAATTTGCCAACTTATCATACAGGCCTTTGAAGCTGGTGT 640

KM034555.2014 TCAAGTAAACAATCTTGAGGAAATTTGCCAACTTATCATACAGGCCTTTGAAGCTGGTGT 652

MH470381.2015 TCAAGTAAACAATCTTGAGGAAATTTGCCAACTTATCATACAGGCCTTTGAAGCTGGTGT 624

MH470382.2015 TCAAGTAAACAATCTTGAGGAAATTTGCCAACTTATCATACAGGCCTTTGAAGCTGGTGT 624

MF102255.2014 TCAAGTAAACAATCTTGAGGAAATTTGCCAACTTATCATACAGGCCTTTGAAGCTGGTGT 637

KJ660348.2014 TCAAGTAAACAATCTTGAGGAAATTTGCCAACTTATCATACAGGCCTTTGAAGCTGGTGT 660

KU143818.2014 TCAAGTAAACAATCTTGAGGAAATTTGCCAACTTATCATACAGGCCTTTGAAGCTGGTGT 660

KT725333.2014 TCAAGTAAACAATCTTGAGGAAATTTGCCAACTTATCATACAGGCCTTTGAAGCTGGTGT 625

KR819004.2014 TCAAGTAAACAATCTTGAGGAGATTTGCCAACTTATCATACAGGCCTTTGAAGCAGGTGT 607

KP271020.2014 TCAAGTAAACAATCTTGAGGAGATTTGCCAACTTATCATACAGGCCTTTGAAGCAGGTGT 607

KM519951.2014 TCAAGTAAACAATCTTGAGGAGATTTGCCAACTTATCATACAGGCCTTTGAAGCAGGTGT 657

MH733488.2018 TCAAGTAAACAATCTTGAGGAGATTTGCCAACTTATCATACAGGCTTTTGAAGCAGGTGT 651

MH733491.2018 TCAAGTAAACAATCTTGAGGAGATTTGCCAACTTATCATACAGGCTTTTGAAGCAGGTGT 646

MH733478.2018 TCAAGTAAACAATCTTGAGGAGATTTGCCAACTTATCATACAGGCTTTTGAAGCAGGTGT 649

MK007330.2018 TCAAGTCAACAATCTTGAGGAAATTTGCCAACTTATCATACAGGCCTTTGAAGCAGGTGT 648

MK007344.2018 TCAAGTCAACAATCTTGAGGAAATTTGCCAACTTATCATACAGGCCTTTGAAGCAGGTGT 642

****** *********** ** *********************** ******** *****

AY142960.1976 TGATTTTCAAGAGAGTGCGGACAGTTTCCTTCTCATGCTTTGTCTTCATCATGCGTACCA 720 EBOV-miR-T1-5p

KC242791.1977 TGATTTTCAAGAGAGTGCGGACAGTTTCCTTCTCATGCTTTGTCTTCATCATGCGTACCA 720

AF499101.1976 TGATTTTCAAGAGAGTGCGGACGGTTTCCTTCTCATGCTTTGTCTTCATCATGCGTACCA 720

KF113528.2003 TGATTTTCAAGAGAGTGCGGACAGTTTCCTTCTCATGCTTTGTCTTCATCATGCGTACCA 716

KC242800.2002 TGATTTTCAAGAGAGTGCGGACAGTTTCCTTCTCATGCTTTGTCTTCATCATGCGTACCA 720

KY471090.2001 TGATTTTCAAGAGAGTGCGGACAGTTTCCTTCTCATGCTTTGTCTTCATCATGCGTACCA 697

KY471092.2001 TGATTTTCAAGAGAGTGCGGACAGTTTCCTTCTCATGCTTTGTCTTCATCATGCGTACCA 697

MH481611.2017 TGATTTTCAAGAGAGTGCGGACAGTTTCCTTCTCATGCTTTGTCTTCATCATGCGTACCA 686

MH613311.2017 TGATTTTCAAGAGAGTGCGGACAGTTTCCTTCTCATGCTTTGTCTTCATCATGCGTACCA 665

KC242792.1994 TGATTTTCAAGAGAGTGCGGACAGTTTCCTTCTCATGCTTTGTCTTCATCATGCGTACCA 720

KC242793.1996 TGATTTTCAAGAGAGTGCGGACAGTTTCCTTCTCATGCTTTGTCTTCATCATGCGTACCA 720

KC242794.1996 TGATTTTCAAGAGAGTGCGGACAGTTTCCTTCTCATGCTTTGTCTTCATCATGCGTACCA 720

MH121164.1995 TGATTTTCAAGAGAGTGCGGACAGTTTCCTTCTCATGCTTTGTCTTCATCATGCGTACCA 720

AY354458.1995 TGATTTTCAAGAGAGTGCGGACAGTTTCCTTCTCATGCTTTGTCTTCATCATGCGTACCA 720

KT762962.1995 TGATTTTCAAGAGAGTGCGGACAGTTTCCTTCTCATGCTTTGTCTTCATCATGCGTACCA 720

HQ613402.2008 TGATTTTCAAGAGAGTGCGGACAGTTTCCTTCTCATGCTTTGTCTTCATCATGCGTACCA 639

KC242789.2007 TGATTTTCAAGAGAGTGCGGACAGTTTCCTTCTCATGCTTTGTCTTCATCATGCGTACCA 720

HQ613403.2007 TGATTTTCAAGAGAGTGCGGACAGTTTCCTTCTCATGCTTTGTCTTCATCATGCGTACCA 672

KC242785.2007 TGATTTTCAAGAGAGTGCGGACAGTTTCCTTCTCATGCTTTGTCTTCATCATGCGTACCA 720

KC242790.2007 TGATTTTCAAGAGAGTGCGGACAGTTTCCTTCTCATGCTTTGTCTTCATCATGCGTACCA 720

KU143789.2014 TGATTTTCAAGAGAGTGCGGACAGTTTCCTTCTCATGCTTTACCTTCATCATCCTTACCA 720

KR817168.2014 TGATTTTCAAGAGAGTGCGGACAGTTTCCTTCTCATGCTTTGTCTTCATCATGCGTACCA 694

KY426696.2015 TGATTTTCAAGAGAGTGCGGACAGTTTCCTTCTCATGCTTTGTCTTCATCATGCGTACCA 720

KR105271.2014 TGATTTTCAAGAGAGTGCGGACAGTTTCCTTCTCATGCTTTGTCTTCATCATGCGTACCA 687

KY007522.2016 TGATTTTCAAGAGAGTGCGGACAGTTTCCTTCTCATGCTTTGTCTTCATCATGCGTACCA 700

KM034555.2014 TGATTTTCAAGAGAGTGCGGACAGTTTCCTTCTCATGCTTTGTCTTCATCATGCGTACCA 712

MH470381.2015 TGATTTTCAAGAGAGTGCGGACAGTTTCCTTCTCATGCTTTGTCTTCATCATGCGTACCA 684

MH470382.2015 TGATTTTCAAGAGAGTGCGGACAGTTTCCTTCTCATGCTTTGTCTTCATCATGCGTACCA 684

MF102255.2014 TGATTTTCAAGAGAGTGCGGACAGTTTCCTTCTCATGCTTTGTCTTCATCATGCGTACCA 697

KJ660348.2014 TGATTTTCAAGAGAGTGCGGACAGTTTCCTTCTCATGCTTTGTCTTCATCATGCGTACCA 720

KU143818.2014 TGATTTTCAAGAGAGTGCGGACAGTTTCCTTCTCATGCTTTGTCTTCATCATGCGTACCA 720

KT725333.2014 TGATTTTCAAGAGAGTGCGGACAGTTTCCTTCTCATGCTTTGTCTTCATCATGCGTACCA 685

KR819004.2014 TGATTTTCAAGAGAGTGCGGACAGTTTCCTTCTCATGCTTTGTCTTCATCATGCGTACCA 667

KP271020.2014 TGATTTTCAAGAGAGTGCGGACAGTTTCCTTCTCATGCTTTGTCTTCATCATGCGTACCA 667

KM519951.2014 TGATTTTCAAGAGAGTGCGGACAGTTTCCTTCTCATGCTTTGTCTTCATCATGCGTACCA 717

MH733488.2018 TGATTTTCAAGAGAGTGCGGACAGTTTCCTTCTCATGCTTTGTCTTCATCATGCGTACCA 711

MH733491.2018 TGATTTTCAAGAGAGTGCGGACAGTTTCCTTCTCATGCTTTGTCTTCATCATGCGTACCA 706

MH733478.2018 TGATTTTCAAGAGAGTGCGGACAGTTTCCTTCTCATGCTTTGTCTTCATCATGCGTACCA 709

MK007330.2018 TGATTTTCAAGAGAGTGCGGACAGTTTCCTTCTCATGCTTTGTCTTCATCATGCGTACCA 708

MK007344.2018 TGATTTTCAAGAGAGTGCGGACAGTTTCCTTCTCATGCTTTGTCTTCATCATGCGTACCA 702

********************** ****************** ********* * *****

AY142960.1976 GGGAGATTACAAACTTTTCTTGGAAAGTGGCGCAGTCAAGTATTTGGAAGGGCACGGGTT 780 EBOV-miR-T1-3p

KC242791.1977 GGGAGATTACAAACTTTTCTTGGAAAGTGGCGCAGTCAAGTATTTGGAAGGGCACGGGTT 780

AF499101.1976 GGGAGATTACAAACTTTTCTTGGAAAGTGGCGCAGTCAAGTATTTGGAAGGGCACGGGTT 780

KF113528.2003 AGGAGATCACAAACTTTTCTTGGAAAGTGGTGCAGTCAAGTATTTGGAAGGGCACGGGTT 776

KC242800.2002 AGGAGATCACAAACTTTTCTTGGAAAGTGGTGCAGTCAAGTATTTGGAAGGGCACGGGTT 780

KY471090.2001 AGGAGATCACAAACTTTTCTTGGAAAGTGGTGCAGTCAAGTATTTGGAAGGGCACGGGTT 757

KY471092.2001 AGGAGATCACAAACTTTTCTTGGAAAGTGGTGCAGTCAAGTATTTGGAAGGGCACGGGTT 757

MH481611.2017 GGGAGATTACAAACTTTTCTTGGAAAGTGGTGCAGTCAAGTATTTGGAAGGGCACGGGTT 746

MH613311.2017 GGGAGATTACAAACTTTTCTTGGAAAGTGGTGCAGTCAAGTATTTGGAAGGGCACGGGTT 725

KC242792.1994 GGGAGATTGCAAACTTTTCTTGGAAAGTGGCGCAGTCAAGTATTTGGAAGGGCACGGGTT 780

KC242793.1996 GGGAGATTGCAAACTTTTCTTGGAAAGTGGCGCAGTCAAGTATTTGGAAGGGCACGGGTT 780

KC242794.1996 GGGAGATTGCAAACTTTTCTTGGAAAGTGGCGCAGTCAAGTATTTGGAAGGGCACGGGTT 780

MH121164.1995 GGGAGATTACAAACTTTTCTTGGAAAGTGGCGCAGTCAAGTATTTGGAAGGGCACGGGTT 780

AY354458.1995 GGGAGATTACAAACTTTTCTTGGAAAGTGGCGCAGTCAAGTATTTGGAAGGGCACGGGTT 780

KT762962.1995 GGGAGATTACAAACTTTTCTTGGAAAGTGGCGCAGTCAAGTATTTGGAAGGGCACGGGTT 780

HQ613402.2008 AGGAGATTACAAACTTTTCTTGGAAAGTGGCGCAGTCAAGTATTTGGAAGGGCACGGGTT 699

KC242789.2007 AGGAGATTACAAACTTTTCTTGGAAAGTGGCGCAGTCAAGTATTTGGAAGGGCACGGGTT 780

HQ613403.2007 AGGAGATTACAAACTTTTCTTGGAAAGTGGCGCAGTCAAGTATTTGGAAGGGCACGGGTT 732

KC242785.2007 AGGAGATTACAAACTTTTCTTGGAAAGTGGCGCAGTCAAGTATTTGGAAGGGCACGGGTT 780

KC242790.2007 AGGAGATTACAAACTTTTCTTGGAAAGTGGCGCAGTCAAGTATTTGGAAGGGCACGGGTT 780

KU143789.2014 AGGAGATTACAAACTTTTCTTGGAAAGTGGCGCAGTCAAGTATTTGGAAGGGCACGGGTT 780

KR817168.2014 AGGAGATTACAAACTTTTCTTGGAAAGTGGCGCAGTCAAGTATTTGGAAGGGCACGGGTT 754

KY426696.2015 AGGAGATTACAAACTTTTCTTGGAAAGTGGCGCAGTCAAGTATTTGGAAGGGCACGGGTT 780

KR105271.2014 AGGAGATTACAAACTTTTCTTGGAAAGTGGCGCAGTCAAGTATTTGGAAGGGCACGGGTT 747

KY007522.2016 AGGAGATTACAAACTTTTCTTGGAAAGTGGCGCAGTCAAGTATTTGGAAGGGCACGGGTT 760

KM034555.2014 AGGAGATTACAAACTTTTCTTGGAAAGTGGCGCAGTCAAGTATTTGGAAGGGCACGGGTT 772

MH470381.2015 AGGAGATTACAAACTTTTCTTGGAAAGTGGCGCAGTCAAGTATTTGGAAGGGCACGGGTT 744

MH470382.2015 AGGAGATTACAAACTTTTCTTGGAAAGTGGCGCAGTCAAGTATTTGGAAGGGCACGGGTT 744

MF102255.2014 AGGAGATTACAAACTTTTCTTGGAAAGTGGCGCAGTCAAGTATTTGGAAGGGCACGGGTT 757

KJ660348.2014 AGGAGATTACAAACTTTTCTTGGAAAGTGGCGCAGTCAAGTATTTGGAAGGGCACGGGTT 780

KU143818.2014 AGGAGATTACAAACTTTTCTTGGAAAGTGGCGCAGTCAAGTATTTGGAAGGGCACGGGTT 780

KT725333.2014 AGGAGATTACAAACTTTTCTTGGAAAGTGGCGCAGTCAAGTATTTGGAAGGGCACGGGTT 745

KR819004.2014 GGGAGATTACAAACTTTTCCTGGAAAGTGGCGCAGTCAAGTACTTGGAAGGGCACGGGTT 727

KP271020.2014 GGGAGATTACAAACTTTTCCTGGAAAGTGGCGCAGTCAAGTACTTGGAAGGGCACGGGTT 727

KM519951.2014 GGGAGATTACAAACTTTTCCTGGAAAGTGGCGCAGTCAAGTACTTGGAAGGGCACGGGTT 777

MH733488.2018 GGGAGATTACAAACTTTTCTTGGAAAGTGGCGCAGTCAAGTACTTGGAAGGGCACGGGTT 771

MH733491.2018 GGGAGATTACAAACTTTTCTTGGAAAGTGGCGCAGTCAAGTACTTGGAAGGGCACGGGTT 766

MH733478.2018 GGGAGATTACAAACTTTTCTTGGAAAGTGGCGCAGTCAAGTACTTGGAAGGGCACGGGTT 769

MK007330.2018 GGGAGATTACAAACTTTTCTTGGAAAGTGGTGCAGTCAAGTATTTGGAAGGGCATGGGTT 768

MK007344.2018 GGGAGATTACAAACTTTTCTTGGAAAGTGGTGCAGTCAAGTATTTGGAAGGGCATGGGTT 762

****** ********** ********** *********** *********** *****

AY142960.1976 CCGTTTTGAAGTCAAGAAGCGTGATGGAGTGAAGCGCCTTGAGGAATTGCTGCCAGCAGT 840

KC242791.1977 CCGTTTTGAAGTCAAGAAGCGTGATGGAGTGAAGCGCCTTGAGGAATTGCTGCCAGCAGT 840

AF499101.1976 CCGTTTTGAAGTCAAGAAGCGTGATGGAGTGAAGCGCCTTGAGGAATTGCTGCCAGCAGT 840

KF113528.2003 CCGTTTTGAAGTCAAGAAACGTGATGGGGTGAAGCGCCTTGAGGAATTGCTGCCAGCAGT 836

KC242800.2002 CCGTTTTGAAGTCAAGAAACGTGATGGGGTGAAGCGCCTTGAGGAATTGCTGCCAGCAGT 840

KY471090.2001 CCGTTTTGAAGTCAAGAAACGTGATGGGGTGAAGCGCCTTGAGGAATTGCTGCCAGCAGT 817

KY471092.2001 CCGTTTTGAAGTCAAGAAACGTGATGGGGTGAAGCGCCTTGAGGAATTGCTGCCAGCAGT 817

MH481611.2017 CCGTTTTGAAGTCAAGAAGCGTGATGGAGTGAAGCGCCTTGAGGAATTGCTGCCAGCAGT 806

MH613311.2017 CCGTTTTGAAGTCAAGAAGCGTGATGGAGTGAAGCGCCTTGAGGAATTGCTGCCAGCAGT 785

KC242792.1994 CCGTTTTGAAGTCAAGAAGCGTGATGGAGTGAAGCGCCTTGAGGAATTGCTGCCAGCAGT 840

KC242793.1996 CCGTTTTGAAGTCAAGAAGCGTGATGGAGTGAAGCGCCTTGAGGAATTGCTGCCAGCAGT 840

KC242794.1996 CCGTTTTGAAGTCAAGAAGCGTGATGGAGTGAAGCGCCTTGAGGAATTGCTGCCAGCAGT 840

MH121164.1995 CCGTTTTGAAGTCAAGAAGCGTGATGGAGTGAAGCGCCTTGAGGAATTGCTGCCAGCAGT 840

AY354458.1995 CCGTTTTGAAGTCAAGAAGCGTGATGGAGTGAAGCGCCTTGAGGAATTGCTGCCAGCAGT 840

KT762962.1995 CCGTTTTGAAGTCAAGAAGCGTGATGGAGTGAAGCGCCTTGAGGAATTGCTGCCAGCAGT 840

HQ613402.2008 CCGTTTTGAAGTCAAGAAGCGTGATGGAGTGAAGCGCCTTGAGGAATTGCTGCCAGCAGT 759

KC242789.2007 CCGTTTTGAAGTCAAGAAGCGTGATGGAGTGAAGCGCCTTGAGGAATTGCTGCCAGCAGT 840

HQ613403.2007 CCGTTTTGAAGTCAAGAAGCGTGATGGAGTGAAGCGCCTTGAGGAATTGCTGCCAGCAGT 792

KC242785.2007 CCGTTTTGAAGTCAAGAAGCGTGATGGAGTGAAGCGCCTTGAGGAATTGCTGCCAGCAGT 840

KC242790.2007 CCGTTTTGAAGTCAAGAAGCGTGATGGAGTGAAGCGCCTTGAGGAATTGCTGCCAGCAGT 840

KU143789.2014 CCGTTTTGAAGTCAAGAAGTGTGATGGAGTGAAGCGCCTTGAGGAATTGCTGCCAGCAGT 840

KR817168.2014 CCGTTTTGAAGTCAAGAAGTGTGATGGAGTGAAGCGCCTTGAGGAATTGCTGCCAGCAGT 814

KY426696.2015 CCGTTTTGAAGTCAAGAAGTGTGATGGAGTGAAGCGCCTTGAGGAATTGCTGCCAGCAGT 840

KR105271.2014 CCGTTTTGAAGTCAAGAAGTGTGATGGAGTGAAGCGCCTTGAGGAATTGCTGCCAGCAGT 807

KY007522.2016 CCGTTTTGAAGTCAAGAAGTGTGATGGAGTGAAGCGCCTTGAGGAATTGCTGCCAGCAGT 820

KM034555.2014 CCGTTTTGAAGTCAAGAAGCGTGATGGAGTGAAGCGCCTTGAGGAATTGCTGCCAGCAGT 832

MH470381.2015 CCGTTTTGAAGTCAAGAAGTGTGATGGAGTGAAGCGCCTTGAGGAATTGCTGCCAGCAGT 804

MH470382.2015 CCGTTTTGAAGTCAAGAAGTGTGATGGAGTGAAGCGCCTTGAGGAATTGCTGCCAGCAGT 804

MF102255.2014 CCGTTTTGAAGTCAAGAAGCGTGATGGAGTGAAGCGCCTTGAGGAATTGCTGCCAGCAGT 817

KJ660348.2014 CCGTTTTGAAGTCAAGAAGCGTGATGGAGTGAAGCGCCTTGAGGAATTGCTGCCAGCAGT 840

KU143818.2014 CCGTTTTGAAGTCAAGAAGTGTGATGGAGTGAAGCGCCTTGAGGAATTGCTGCCAGCAGT 840

KT725333.2014 CCGTTTTGAAGTCAAGAAGTGTGATGGAGTGAAGCGCCTTGAGGAATTGCTGCCAGCAGT 805

KR819004.2014 CCGTTTTGAAGTCAAGAAGCGTGATGGAGTGAAGCGCCTTGAGGAATTGCTGCCAGCAGT 787

KP271020.2014 CCGTTTTGAAGTCAAGAAGCGTGATGGAGTGAAGCGCCTTGAGGAATTGCTGCCAGCAGT 787

KM519951.2014 CCGTTTTGAAGTCAAGAAGCGTGATGGAGTGAAGCGCCTTGAGGAATTGCTGCCAGCAGT 837

MH733488.2018 CCGTTTTGAAGTCAAGAAGCGTGATGGAGTGAAGCGCCTTGAGGAATTGCTGCCAGCAGT 831

MH733491.2018 CCGTTTTGAAGTCAAGAAGCGTGATGGAGTGAAGCGCCTTGAGGAATTGCTGCCAGCAGT 826

MH733478.2018 CCGTTTTGAAGTCAAGAAGCGTGATGGAGTGAAGCGCCTTGAGGAATTGCTGCCAGCAGT 829

MK007330.2018 CCGTTTTGAAGTCAAGAAGCGTGATGGAGTGAAGCGCCTTGAGGAATTGCTGCCAGCAGT 828

MK007344.2018 CCGTTTTGAAGTCAAGAAGCGTGATGGAGTGAAGCGCCTTGAGGAATTGCTGCCAGCAGT 822

****************** ******* ********************************

AY142960.1976 ATCTAGTGGAAAAAACATTAAGAGAACACTTGCTGCCATGCCGGAAGAGGAGACAACTGA 900

KC242791.1977 ATCTAGTGGAAAAAACATTAAGAGAACACTTGCTGCCATGCCGGAAGAGGAGACAACTGA 900

AF499101.1976 ATCTAGTGGAAAAAACATTAAGAGAACACTTGCTGCCATGCCGGAAGAGGAGACAACTGA 900

KF113528.2003 ATCTAGTGGAAAAAACATTAAGAGAACACTTGCTGCCATGCCGGAAGAGGAGACGACTGA 896

KC242800.2002 ATCTAGTGGAAAAAACATTAAGAGAACACTTGCTGCCATGCCGGAAGAGGAGACGACTGA 900

KY471090.2001 ATCTAGTGGAAAAAACATTAAGAGAACACTTGCTGCCATGCCGGAAGAGGAGACGACTGA 877

KY471092.2001 ATCTAGTGGAAAAAACATTAAGAGAACACTTGCTGCCATGCCGGAAGAGGAGACGACTGA 877

MH481611.2017 ATCTAGTGGAAAAAACATTAAGAGAACACTTGCTGCCATGCCGGAAGAGGAGACAACTGA 866

MH613311.2017 ATCTAGTGGAAAAAACATTAAGAGAACACTTGCTGCCATGCCGGAAGAGGAGACAACTGA 845

KC242792.1994 ATCTAGTGGAAAAAACATTAAGAGAACACTTGCTGCCATGCCGGAAGAGGAGACAACTGA 900

KC242793.1996 ATCTAGTGGAAAAAACATTAAGAGAACACTTGCTGCCATGCCGGAAGAGGAGACAACTGA 900

KC242794.1996 ATCTAGTGGAAAAAACATTAAGAGAACACTTGCTGCCATGCCGGAAGAGGAGACAACTGA 900

MH121164.1995 ATCTAGTGGAAAAAACATTAAGAGAACACTTGCTGCCATGCCGGAAGAGGAGACAACTGA 900

AY354458.1995 ATCTAGTGGAAAAAACATTAAGAGAACACTTGCTGCCATGCCGGAAGAGGAGACAACTGA 900

KT762962.1995 ATCTAGTGGAAAAAACATTAAGAGAACACTTGCTGCCATGCCGGAAGAGGAGACAACTGA 900

HQ613402.2008 ATCTAGTGGAAAAAACATTAAGAGAACACTTGCTGCCATGCCGGAAGAGGAGACGACTGA 819

KC242789.2007 ATCTAGTGGAAAAAACATTAAGAGAACACTTGCTGCCATGCCGGAAGAGGAGACGACTGA 900

HQ613403.2007 ATCTAGTGGAAAAAACATTAAGAGAACACTTGCTGCCATGCCGGAAGAGGAGACGACTGA 852

KC242785.2007 ATCTAGTGGAAAAAACATTAAGAGAACACTTGCTGCCATGCCGGAAGAGGAGACGACTGA 900

KC242790.2007 ATCTAGTGGAAAAAACATTAAGAGAACACTTGCTGCCATGCCGGAAGAGGAGACGACTGA 900

KU143789.2014 ATCTAGTGGGAGAAACATTAAGAGAACACTTGCTGCCATGCCGGAAGAGGAGACGACTGA 900

KR817168.2014 ATCTAGTGGGAGAAACATTAAGAGAACACTTGCTGCCATGCCGGAAGAGGAGACGACTGA 874

KY426696.2015 ATCTAGTGGGAGAAACATTAAGAGAACACTTGCTGCCATGCCGGAAGAGGAGACGACTGA 900

KR105271.2014 ATCTAGTGGGAGAAACATTAAGAGAACACTTGCTGCCATGCCGGAAGAGGAGACGACTGA 867

KY007522.2016 ATCTAGTGGGAGAAACATTAAGAGAACACTTGCTGCCATGCCGGAAGAGGAGACGACTGA 880

KM034555.2014 ATCTAGTGGGAGAAACATTAAGAGAACACTTGCTGCCATGCCGGAAGAGGAGACGACTGA 892

MH470381.2015 ATCTAGTGGGAGAAACATTAAGAGAACACTTGCTGCCATGCCGGAAGAGGAGACGACTGA 864

MH470382.2015 ATCTAGTGGGAGAAACATTAAGAGAACACTTGCTGCCATGCCGGAAGAGGAGACGACTGA 864

MF102255.2014 ATCTAGTGGGAGAAACATTAAGAGAACACTTGCTGCCATGCCGGAAGAGGAGACGACTGA 877

KJ660348.2014 ATCTAGTGGGAGAAACATTAAGAGAACACTTGCTGCCATGCCGGAAGAGGAGACGACTGA 900

KU143818.2014 ATCTAGTGGGAGAAACATTAAGAGAACACTTGCTGCCATGCCGGAAGAGGAGACGACTGA 900

KT725333.2014 ATCTAGTGGGAGAAACATTAAGAGAACACTTGCTGCCATGCCGGAAGAGGAGACGACTGA 865

KR819004.2014 ATCTAGTGGAAAAAACATTAAGAGAACACTTGCTGCCATGCCGGAAGAGGAGACAACTGA 847

KP271020.2014 ATCTAGTGGAAAAAACATTAAGAGAACACTTGCTGCCATGCCGGAAGAGGAGACAACTGA 847

KM519951.2014 ATCTAGTGGAAAAAACATTAAGAGAACACTTGCTGCCATGCCGGAAGAGGAGACAACTGA 897

MH733488.2018 ATCTAGTGGAAAAAACATTAAGAGAACACTTGCTGCCATGCCGGAAGAGGAGACAACTGA 891

MH733491.2018 ATCTAGTGGAAAAAACATTAAGAGAACACTTGCTGCCATGCCGGAAGAGGAGACAACTGA 886

MH733478.2018 ATCTAGTGGAAAAAACATTAAGAGAACACTTGCTGCCATGCCGGAAGAGGAGACAACTGA 889

MK007330.2018 ATCTAGTGGAAAAAACATTAAGAGAACACTTGCTGCCATGCCGGAAGAGGAGACAACTGA 888

MK007344.2018 ATCTAGTGGAAAAAACATTAAGAGAACACTTGCTGCCATGCCGGAAGAGGAGACAACTGA 882

********* * ****************************************** *****

AY142960.1976 AGCTAATGCCGGTCAGTTTCTCTCCTTTGCAAGTCTATTCCTTCCGAAATTGGTAGTAGG 960

KC242791.1977 AGCTAATGCCGGTCAGTTTCTCTCCTTTGCAAGTCTATTCCTTCCGAAATTGGTAGTAGG 960

AF499101.1976 AGCTAATGCCGGTCAGTTTCTCTCCTTTGCAAGTCTATTCCTTCCGAAATTGGTAGTAGG 960

KF113528.2003 AGCTAATGCCGGCCAGTTTCTCTCTTTTGCAAGTCTATTCCTTCCGAAATTGGTAGTAGG 956

KC242800.2002 AGCTAATGCCGGTCAGTTTCTCTCTTTTGCAAGTCTATTCCTTCCGAAATTGGTAGTAGG 960

KY471090.2001 AGCTAATGCCGGTCAGTTTCTCTCTTTTGCAAGTCTATTCCTTCCGAAATTGGTAGTAGG 937

KY471092.2001 AGCTAATGCCGGTCAGTTTCTCTCTTTTGCAAGTCTATTCCTTCCGAAATTGGTAGTAGG 937

MH481611.2017 AGCTAATGCCGGTCAGTTTCTCTCCTTTGCAAGTCTATTCCTTCCGAAATTGGTAGTAGG 926

MH613311.2017 AGCTAATGCCGGTCAGTTTCTCTCCTTTGCAAGTCTATTCCTTCCGAAATTGGTAGTAGG 905

KC242792.1994 AGCCAATGCCGGTCAGTTTCTCTCCTTTGCAAGTCTATTCCTTCCGAAATTGGTAGTAGG 960

KC242793.1996 AGCCAATGCCGGTCAGTTTCTCTCCTTTGCAAGTCTATTCCTTCCGAAATTGGTAGTAGG 960

KC242794.1996 AGCTAATGCCGGTCAGTTTCTCTCCTTTGCAAGTCTATTCCTTCCGAAATTGGTAGTAGG 960

MH121164.1995 AGCTAATGCCGGTCAGTTTCTCTCCTTTGCAAGTCTATTCCTTCCGAAATTGGTAGTAGG 960

AY354458.1995 AGCTAATGCCGGTCAGTTTCTCTCCTTTGCAAGTCTATTCCTTCCGAAATTGGTAGTAGG 960

KT762962.1995 AGCTAATGCCGGTCAGTTTCTCTCCTTTGCAAGTCTATTCCTTCCGAAATTGGTAGTAGG 960

HQ613402.2008 AGCTAATGCCGGCCAGTTTCTCTCCTTTGCAAGTCTATTCCTTCCGAAATTGGTAGTAGG 879

KC242789.2007 AGCTAATGCCGGCCAGTTTCTCTCCTTTGCAAGTCTATTCCTTCCGAAATTGGTAGTAGG 960

HQ613403.2007 AGCTAATGCCGGCCAGTTTCTCTCCTTTGCAAGTCTATTCCTTCCGAAATTGGTAGTAGG 912

KC242785.2007 AGCTAATGCCGGCCAGTTTCTCTCCTTTGCAAGTCTATTCCTTCCGAAATTGGTAGTAGG 960

KC242790.2007 AGCTAATGCCGGCCAGTTTCTCTCCTTTGCAAGTCTATTCCTTCCGAAATTGGTAGTAGG 960

KU143789.2014 AGCTAATGCCGGTCAGTTCCTCTCCTTTGCAAGTCTATTCCTTCCGAAATTGGTAGTAGG 960

KR817168.2014 AGCTAATGCCGGTCAGTTCCTCTCCTTTGCAAGTCTATTCCTTCCGAAATTGGTAGTAGG 934

KY426696.2015 AGCTAATGCCGGTCAGTTCCTCTCCTTTGCAAGTCTATTCCTTCCGAAATTGGTAGTAGG 960

KR105271.2014 AGCTAATGCCGGTCAGTTCCTCTCCTTTGCAAGTCTATTCCTTCCGAAATTGGTAGTAGG 927

KY007522.2016 AGCTAATGCCGGTCAGTTCCTCTCCTTTGCAAGTCTATTCCTTCCGAAATTGGTAGTAGG 940

KM034555.2014 AGCTAATGCCGGTCAGTTCCTCTCCTTTGCAAGTCTATTCCTTCCGAAATTGGTAGTAGG 952

MH470381.2015 AGCTAATGCCGGTCAGTTCCTCTCCTTTGCAAGTCTATTCCTTCCGAAATTGGTAGTAGG 924

MH470382.2015 AGCTAATGCCGGTCAGTTCCTCTCCTTTGCAAGTCTATTCCTTCCGAAATTGGTAGTAGG 924

MF102255.2014 AGCTAATGCCGGTCAGTTCCTCTCCTTTGCAAGTCTATTCCTTCCGAAATTGGTAGTAGG 937

KJ660348.2014 AGCTAATGCCGGTCAGTTCCTCTCCTTTGCAAGTCTATTCCTTCCGAAATTGGTAGTAGG 960

KU143818.2014 AGCTAATGCCGGTCAGTTCCTCTCCTTTGCAAGTCTATTCCTTCCGAAATTGGTAGTAGG 960

KT725333.2014 AGCTAATGCCGGTCAGTTCCTCTCCTTTGCAAGTCTATTCCTTCCGAAATTGGTAGTAGG 925

KR819004.2014 AGCTAATGCCGGTCAGTTTCTCTCCTTTGCAAGTCTATTCCTTCCGAAATTGGTAGTAGG 907

KP271020.2014 AGCTAATGCCGGTCAGTTTCTCTCCTTTGCAAGTCTATTCCTTCCGAAATTGGTAGTAGG 907

KM519951.2014 AGCTAATGCCGGTCAGTTTCTCTCCTTTGCAAGTCTATTCCTTCCGAAATTGGTAGTAGG 957

MH733488.2018 AGCTAATGCCGGTCAGTTTCTCTCCTTTGCAAGTCTATTCCTTCCGAAATTGGTAGTAGG 951

MH733491.2018 AGCTAATGCCGGTCAGTTTCTCTCCTTTGCAAGTCTATTCCTTCCGAAATTGGTAGTAGG 946

MH733478.2018 AGCTAATGCCGGTCAGTTTCTCTCCTTTGCAAGTCTATTCCTTCCGAAATTGGTAGTAGG 949

MK007330.2018 AGCTAATGCCGGTCAGTTTCTCTCCTTTGCCAGTCTATTCCTTCCGAAATTGGTAGTAGG 948

MK007344.2018 AGCTAATGCCGGTCAGTTTCTCTCCTTTGCCAGTCTATTCCTTCCGAAATTGGTAGTAGG 942

*** ******** ***** ***** ***** *****************************

AY142960.1976 AGAAAAGGCTTGCCTTGAGAAGGTTCAAAGGCAAATTCAAGTACATGCAGAGCAAGGACT 1020

KC242791.1977 AGAAAAGGCTTGCCTTGAGAAGGTTCAAAGGCAAATTCAAGTACATGCAGAGCAAGGACT 1020

AF499101.1976 AGAAAAGGCTTGCCTTGAGAAGGTTCAAAGGCAAATTCAAGTACATGCAGAGCAAGGACT 1020

KF113528.2003 AGAAAAGGCTTGCCTTGAGAAAGTTCAAAGGCAAATTCAAGTACATGCAGAGCAAGGACT 1016

KC242800.2002 AGAAAAGGCTTGCCTTGAGAAAGTTCAAAGGCAAATTCAAGTACATGCAGAGCAAGGACT 1020

KY471090.2001 AGAAAAGGCTTGCCTTGAGAAAGTTCAAAGGCAAATTCAAGTACATGCAGAGCAAGGACT 997

KY471092.2001 AGAAAAGGCTTGCCTTGAGAAAGTTCAAAGGCAAATTCAAGTACATGCAGAGCAAGGACT 997

MH481611.2017 AGAAAAGGCTTGCCTTGAGAAGGTTCAAAGGCAAATTCAAGTACATGCAGAGCAAGGACT 986

MH613311.2017 AGAAAAGGCTTGCCTTGAGAAGGTTCAAAGGCAAATTCAAGTACATGCAGAGCAAGGACT 965

KC242792.1994 AGAAAAGGCTTGCCTTGAGAAGGTTCAAAGGCAAATTCAAGTACATGCAGAGCAAGGACT 1020

KC242793.1996 AGAAAAGGCTTGCCTTGAGAAGGTTCAAAGGCAAATTCAAGTACATGCAGAGCAAGGACT 1020

KC242794.1996 AGAAAAGGCTTGCCTTGAGAAGGTTCAAAGGCAAATTCAAGTACATGCAGAGCAAGGACT 1020

MH121164.1995 AGAAAAGGCTTGTCTTGAGAAGGTTCAAAGGCAAATTCAAGTACATGCAGAGCAAGGACT 1020

AY354458.1995 AGAAAAGGCTTGTCTTGAGAAGGTTCAAAGGCAAATTCAAGTACATGCAGAGCAAGGACT 1020

KT762962.1995 AGAAAAGGCTTGTCTTGAGAAGGTTCAAAGGCAAATTCAAGTACATGCAGAGCAAGGACT 1020

HQ613402.2008 AGAAAAGGCTTGCCTTGAGAAGGTTCAAAGGCAAATTCAAGTACATGCAGAGCAAGGACT 939

KC242789.2007 AGAAAAGGCTTGCCTTGAGAAGGTTCAAAGGCAAATTCAAGTACATGCAGAGCAAGGACT 1020

HQ613403.2007 AGAAAAGGCTTGCCTTGAGAAGGTTCAAAGGCAAATTCAAGTACATGCAGAGCAAGGACT 972

KC242785.2007 AGAAAAGGCTTGCCTTGAGAAGGTTCAAAGGCAAATTCAAGTACATGCAGAGCAAGGACT 1020

KC242790.2007 AGAAAAGGCTTGCCTTGAGAAGGTTCAAAGGCAAATTCAAGTACATGCAGAGCAAGGACT 1020

KU143789.2014 AGAAAAGGCTTGCCTTGAGAAGGTTCAAAGGCAAATTCAAGTACATGCAGAGCAAGGACT 1020

KR817168.2014 AGAAAAGGCTTGCCTTGAGAAGGTTCAAAGGCAAATTCAAGTACATGCAGAGCAAGGACT 994

KY426696.2015 AGAAAAGGCTTGCCTTGAGAAGGTTCAAAGGCAAATTCAAGTACATGCAGAGCAAGGACT 1020

KR105271.2014 AGAAAAGGCTTGCCTTGAGAAGGTTCAAAGGCAAATTCAAGTACATGCAGAGCAAGGACT 987

KY007522.2016 AGAAAAGGCTTGCCTTGAGAAGGTTCAAAGGCAAATTCAAGTACATGCAGAGCAAGGACT 1000

KM034555.2014 AGAAAAGGCTTGCCTTGAGAAGGTTCAAAGGCAAATTCAAGTACATGCAGAGCAAGGACT 1012

MH470381.2015 AGAAAAGGCTTGCCTTGAGAAGGTTCAAAGGCAAATTCAAGTACATGCAGAGCAAGGACT 984

MH470382.2015 AGAAAAGGCTTGCCTTGAGAAGGTTCAAAGGCAAATTCAAGTACATGCAGAGCAAGGACT 984

MF102255.2014 AGAAAAGGCTTGCCTTGAGAAGGTTCAAAGGCAAATTCAAGTACATGCAGAGCAAGGACT 997

KJ660348.2014 AGAAAAGGCTTGCCTTGAGAAGGTTCAAAGGCAAATTCAAGTACATGCAGAGCAAGGACT 1020

KU143818.2014 AGAAAAGGCTTGCCTTGAGAAGGTTCAAAGGCAAATTCAAGTACATGCAGAGCAAGGACT 1020

KT725333.2014 AGAAAAGGCTTGCCTTGAGAAGGTTCAAAGGCAAATTCAAGTACATGCAGAGCAAGGACT 985

KR819004.2014 AGAAAAGGCTTGCCTTGAGAAGGTTCAAAGGCAAATTCAAGTACATGCAGAGCAAGGACT 967

KP271020.2014 AGAAAAGGCTTGCCTTGAGAAGGTTCAAAGGCAAATTCAAGTACATGCAGAGCAAGGACT 967

KM519951.2014 AGAAAAGGCTTGCCTTGAGAAGGTTCAAAGGCAAATTCAAGTACATGCAGAGCAAGGACT 1017

MH733488.2018 AGAAAAGGCTTGCCTTGAGAAGGTTCAAAGGCAAATTCAAGTACATGCAGAGCAAGGACT 1011

MH733491.2018 AGAAAAGGCTTGCCTTGAGAAGGTTCAAAGGCAAATTCAAGTACATGCAGAGCAAGGACT 1006

MH733478.2018 AGAAAAGGCTTGCCTTGAGAAGGTTCAAAGGCAAATTCAAGTACATGCAGAGCAAGGACT 1009

MK007330.2018 AGAAAAGGCTTGTCTTGAGAAGGTTCAAAGGCAAATTCAAGTACATGCAGAGCAAGGACT 1008

MK007344.2018 AGAAAAGGCTTGTCTTGAGAAGGTTCAAAGGCAAATTCAAGTACATGCAGAGCAAGGACT 1002

************ ******** ************************************** >100 pos conserved

AY142960.1976 GATACAATATCCAACAGCTTGGCAATCAGTAGGACACATGATGGTGATTTTCCGTTTGAT 1080

KC242791.1977 GATACAATATCCAACAGCTTGGCAATCAGTAGGACACATGATGGTGATTTTCCGTTTGAT 1080

AF499101.1976 GATACAATATCCAACAGCTTGGCAATCAGTAGGACACATGATGGTGATTTTCCGTTTGAT 1080

KF113528.2003 GATACAATATCCAACAGCTTGGCAATCAGTAGGACACATGATGGTGATTTTCCGTTTGAT 1076

KC242800.2002 GATACAATATCCAACAGCTTGGCAATCAGTAGGACACATGATGGTGATTTTCCGTTTGAT 1080

KY471090.2001 GATACAATATCCAACAGCTTGGCAATCAGTAGGACACATGATGGTGATTTTCCGTTTGAT 1057

KY471092.2001 GATACAATATCCAACAGCTTGGCAATCAGTAGGACACATGATGGTGATTTTCCGTTTGAT 1057

MH481611.2017 GATACAATATCCAACAGCTTGGCAATCAGTAGGACACATGATGGTGATTTTCCGTTTGAT 1046

MH613311.2017 GATACAATATCCAACAGCTTGGCAATCAGTAGGACACATGATGGTGATTTTCCGTTTGAT 1025

KC242792.1994 GATACAATATCCAACAGCTTGGCAATCAGTAGGACACATGATGGTGATTTTCCGTTTGAT 1080

KC242793.1996 GATACAATATCCAACAGCTTGGCAATCAGTAGGACACATGATGGTGATTTTCCGTTTGAT 1080

KC242794.1996 GATACAATATCCAACAGCTTGGCAATCAGTAGGACACATGATGGTGATTTTCCGTTTGAT 1080

MH121164.1995 GATACAATATCCAACAGCTTGGCAATCAGTAGGACACATGATGGTGATTTTCCGTTTGAT 1080

AY354458.1995 GATACAATATCCAACAGCTTGGCAATCAGTAGGACACATGATGGTGATTTTCCGTTTGAT 1080

KT762962.1995 GATACAATATCCAACAGCTTGGCAATCAGTAGGACACATGATGGTGATTTTCCGTTTGAT 1080

HQ613402.2008 GATACAATATCCAACAGCTTGGCAATCAGTAGGACACATGATGGTGATTTTCCGTTTGAT 999

KC242789.2007 GATACAATATCCAACAGCTTGGCAATCAGTAGGACACATGATGGTGATTTTCCGTTTGAT 1080

HQ613403.2007 GATACAATATCCAACAGCTTGGCAATCAGTAGGACACATGATGGTGATTTTCCGTTTGAT 1032

KC242785.2007 GATACAATATCCAACAGCTTGGCAATCAGTAGGACACATGATGGTGATTTTCCGTTTGAT 1080

KC242790.2007 GATACAATATCCAACAGCTTGGCAATCAGTAGGACACATGATGGTGATTTTCCGTTTGAT 1080

KU143789.2014 GATACAATATCCAACAGCTTGGCAATCAGTAGGACACATGATGGTGATTTTCCGTTTGAT 1080

KR817168.2014 GATACAATATCCAACAGCTTGGCAATCAGTAGGACACATGATGGTGATTTTCCGTTTGAT 1054

KY426696.2015 GATACAATATCCAACAGCTTGGCAATCAGTAGGACACATGATGGTGATTTTCCGTTTGAT 1080

KR105271.2014 GATACAATATCCAACAGCTTGGCAATCAGTAGGACACATGATGGTGATTTTCCGTTTGAT 1047

KY007522.2016 GATACAATATCCAACAGCTTGGCAATCAGTAGGACACATGATGGTGATTTTCCGTTTGAT 1060

KM034555.2014 GATACAATATCCAACAGCTTGGCAATCAGTAGGACACATGATGGTGATTTTCCGTTTGAT 1072

MH470381.2015 GATACAATATCCAACAGCTTGGCAATCAGTAGGACACATGATGGTGATTTTCCGTTTGAT 1044

MH470382.2015 GATACAATATCCAACAGCTTGGCAATCAGTAGGACACATGATGGTGATTTTCCGTTTGAT 1044

MF102255.2014 GATACAATATCCAACAGCTTGGCAATCAGTAGGACACATGATGGTGATTTTCCGTTTGAT 1057

KJ660348.2014 GATACAATATCCAACAGCTTGGCAATCAGTAGGACACATGATGGTGATTTTCCGTTTGAT 1080

KU143818.2014 GATACAATATCCAACAGCTTGGCAATCAGTAGGACACATGATGGTGATTTTCCGTTTGAT 1080

KT725333.2014 GATACAATATCCAACAGCTTGGCAATCAGTAGGACACATGATGGTGATTTTCCGTTTGAT 1045

KR819004.2014 GATACAATATCCAACAGCTTGGCAATCAGTAGGACACATGATGGTGATTTTCCGTTTGAT 1027

KP271020.2014 GATACAATATCCAACAGCTTGGCAATCAGTAGGACACATGATGGTGATTTTCCGTTTGAT 1027

KM519951.2014 GATACAATATCCAACAGCTTGGCAATCAGTAGGACACATGATGGTGATTTTCCGTTTGAT 1077

MH733488.2018 GATACAATATCCAACAGCTTGGCAATCAGTAGGACACATGATGGTGATTTTCCGTTTGAT 1071

MH733491.2018 GATACAATATCCAACAGCTTGGCAATCAGTAGGACACATGATGGTGATTTTCCGTTTGAT 1066

MH733478.2018 GATACAATATCCAACAGCTTGGCAATCAGTAGGACACATGATGGTGATTTTCCGTTTGAT 1069

MK007330.2018 GATACAATATCCAACAGCTTGGCAATCAGTAGGACACATGATGGTGATTTTTCGTTTGAT 1068

MK007344.2018 GATACAATATCCAACAGCTTGGCAATCAGTAGGACACATGATGGTGATTTTTCGTTTGAT 1062

*************************************************** ********

AY142960.1976 GCGAACAAATTTTCTGATCAAATTTCTCCTAATACACCAAGGGATGCACATGGTTGCCGG 1140

KC242791.1977 GCGAACAAATTTTCTGATCAAATTTCTCCTAATACACCAAGGGATGCACATGGTTGCCGG 1140

AF499101.1976 GCGAACAAATTTTCTGATCAAATTTCTCCTAATACACCAAGGGATGCACATGGTTGCCGG 1140

KF113528.2003 GCGAACAAATTTTTTGATCAAATTTCTCCTAATACACCAAGGGATGCACATGGTTGCCGG 1136

KC242800.2002 GCGAACAAATTTTTTGATCAAATTTCTCCTAATACACCAAGGGATGCACATGGTTGCCGG 1140

KY471090.2001 GCGAACAAATTTTTTGATCAAATTTCTCCTAATACACCAAGGGATGCACATGGTTGCCGG 1117

KY471092.2001 GCGAACAAATTTTTTGATCAAATTTCTCCTAATACACCAAGGGATGCACATGGTTGCCGG 1117

MH481611.2017 GCGAACAAATTTTCTGATCAAATTTCTCCTAATACACCAAGGGATGCACATGGTTGCCGG 1106

MH613311.2017 GCGAACAAATTTTCTGATCAAATTTCTCCTAATACACCAAGGGATGCACATGGTTGCCGG 1085

KC242792.1994 GCGAACAAATTTTTTGATCAAATTTCTCCTAATACACCAAGGGATGCACATGGTTGCCGG 1140

KC242793.1996 GCGAACAAATTTTTTGATCAAATTTCTCCTAATACACCAAGGGATGCACATGGTTGCCGG 1140

KC242794.1996 GCGAACAAATTTTTTGATCAAATTTCTCCTAATACACCAAGGGATGCACATGGTTGCCGG 1140

MH121164.1995 GCGAACAAATTTTTTGATCAAATTTCTCCTAATACACCAAGGGATGCACATGGTTGCCGG 1140

AY354458.1995 GCGAACAAATTTTTTGATCAAATTTCTCCTAATACACCAAGGGATGCACATGGTTGCCGG 1140

KT762962.1995 GCGAACAAATTTTTTGATCAAATTTCTCCTAATACACCAAGGGATGCACATGGTTGCCGG 1140

HQ613402.2008 GCGAACAAATTTCTTGATCAAATTTCTCCTAATACACCAAGGGATGCACATGGTTGCCGG 1059

KC242789.2007 GCGAACAAATTTCTTGATCAAATTTCTCCTAATACACCAAGGGATGCACATGGTTGCCGG 1140

HQ613403.2007 GCGAACAAATTTCTTGATCAAATTTCTCCTAATACACCAAGGGATGCACATGGTTGCCGG 1092

KC242785.2007 GCGAACAAATTTCTTGATCAAATTTCTCCTAATACACCAAGGGATGCACATGGTTGCCGG 1140

KC242790.2007 GCGAACAAATTTCTTGATCAAATTTCTCCTAATACACCAAGGGATGCACATGGTTGCCGG 1140

KU143789.2014 GCGAACAAATTTTTTGATCAAATTTCTTCTAATACACCAAGGGATGCACATGGTTGCCGG 1140

KR817168.2014 GCGAACAAATTTTTTGATCAAATTTCTTCTAATACACCAAGGGATGCACATGGTTGCCGG 1114

KY426696.2015 GCGAACAAATTTTTTGATCAAATTTCTTCTAATACACCAAGGGATGCACATGGTTGCCGG 1140

KR105271.2014 GCGAACAAATTTTTTGATCAAATTTCTTCTAATACACCAAGGGATGCACATGGTTGCCGG 1107

KY007522.2016 GCGAACAAATTTTTTGATCAAATTTCTTCTAATACACCAAGGGATGCACATGGTTGCCGG 1120

KM034555.2014 GCGAACAAATTTTTTGATCAAATTTCTTCTAATACACCAAGGGATGCACATGGTTGCCGG 1132

MH470381.2015 GCGAACAAATTTTTTGATCAAATTTCTTCTAATACACCAAGGGATGCACATGGTTGCCGG 1104

MH470382.2015 GCGAACAAATTTTTTGATCAAATTTCTTCTAATACACCAAGGGATGCACATGGTTGCCGG 1104

MF102255.2014 GCGAACAAATTTTTTGATCAAATTTCTTCTAATACACCAAGGGATGCACATGGTTGCCGG 1117

KJ660348.2014 GCGAACAAATTTTTTGATCAAATTTCTTCTAATACACCAAGGGATGCACATGGTTGCCGG 1140

KU143818.2014 GCGAACAAATTTTTTGATCAAATTTCTTCTAATACACCAAGGGATGCACATGGTTGCCGG 1140

KT725333.2014 GCGAACAAATTTTTTGATCAAATTTCTTCTAATACACCAAGGGATGCACATGGTTGCCGG 1105

KR819004.2014 GCGAACAAATTTTTTGATCAAATTTCTCCTAATACACCAAGGGATGCACATGGTTGCCGG 1087

KP271020.2014 GCGAACAAATTTTTTGATCAAATTTCTCCTAATACACCAAGGGATGCACATGGTTGCCGG 1087

KM519951.2014 GCGAACAAATTTTTTGATCAAATTTCTCCTAATACACCAAGGGATGCACATGGTTGCCGG 1137

MH733488.2018 GCGAACAAATTTTTTGATCAAATTTCTCCTAATACACCAAGGGATGCACATGGTTGCCGG 1131

MH733491.2018 GCGAACAAATTTTTTGATCAAATTTCTCCTAATACACCAAGGGATGCACATGGTTGCCGG 1126

MH733478.2018 GCGAACAAATTTTTTGATCAAATTTCTCCTAATACACCAAGGGATGCACATGGTTGCCGG 1129

MK007330.2018 GCGAACAAATTTTCTGATCAAATTTCTCCTAATACACCAAGGGATGCACATGGTTGCCGG 1128

MK007344.2018 GCGAACAAATTTTCTGATCAAATTTCTCCTAATACACCAAGGGATGCACATGGTTGCCGG 1122

************ ************* ********************************

AY142960.1976 GCATGAT*GCCAACGATGCTGTGATTTC*AAATTCAGTGGCTCAAGCTCGTTTTTCAGGCTT 1200 *EBOV-NP-Fwd Lau (22)*

KC242791.1977 GCATGAT*GCCAACGATGCTGTGATTTC*AAATTCAGTGGCTCAAGCTCGTTTTTCAGGCTT 1200

AF499101.1976 GCATGAT*GCCAACGATGCTGTGATTTC*AAATTCAGTGGCTCAAGCTCGTTTTTCAGGCTT 1200

KF113528.2003 GCATGAT*GCCAACGATGCTGTGATTTC*AAATTCAGTGGCTCAAGCTCGTTTTTCAGGTTT 1196

KC242800.2002 GCATGAT*GCCAACGATGCTGTGATTTC*AAATTCAGTGGCTCAAGCTCGTTTTTCAGGTTT 1200

KY471090.2001 GCATGAT*GCCAACGATGCTGTGATTTC*AAATTCAGTGGCTCAAGCTCGTTTTTCAGGTTT 1177

KY471092.2001 GCATGAT*GCCAACGATGCTGTGATTTC*AAATTCAGTGGCTCAAGCTCGTTTTTCAGGTTT 1177

MH481611.2017 GCATGAT*GCCAACGATGCTGTGATTTC*AAATTCAGTGGCTCAAGCTCGTTTTTCAGGTTT 1166

MH613311.2017 GCATGAT*GCCAACGATGCTGTGATTTC*AAATTCAGTGGCTCAAGCTCGTTTTTCAGGTTT 1145

KC242792.1994 GCATGATGCCAACGACGCTGTGATTTCAAATTCAGTGGCTCAAGCTCGTTTTTCAGGTTT 1200

KC242793.1996 GCATGATGCCAACGACGCTGTGATTTCAAATTCAGTGGCTCAAGCTCGTTTTTCAGGTTT 1200

KC242794.1996 GCATGATGCCAACGACGCTGTGATTTCAAATTCAGTGGCTCAAGCTCGTTTTTCAGGTTT 1200

MH121164.1995 GCATGATGCCAACGACGCTGTGATTTCAAATTCAGTGGCTCAAGCTCGTTTTTCAGGTTT 1200

AY354458.1995 GCATGATGCCAACGACGCTGTGATTTCAAATTCAGTGGCTCAAGCTCGTTTTTCAGGTTT 1200

KT762962.1995 GCATGATGCCAACGACGCTGTGATTTCAAATTCAGTGGCTCAAGCTCGTTTTTCAGGTTT 1200

HQ613402.2008 GCATGAT*GCCAACGATGCTGTGATTTC*AAATTCAGTGGCTCAAGCTCGTTTTTCAGGTTT 1119

KC242789.2007 GCATGAT*GCCAACGATGCTGTGATTTC*AAATTCAGTGGCTCAAGCTCGTTTTTCAGGTTT 1200

HQ613403.2007 GCATGAT*GCCAACGATGCTGTGATTTC*AAATTCAGTGGCTCAAGCTCGTTTTTCAGGTTT 1152

KC242785.2007 GCATGAT*GCCAACGATGCTGTGATTTC*AAATTCAGTGGCTCAAGCTCGTTTTTCAGGTTT 1200

KC242790.2007 GCATGAT*GCCAACGATGCTGTGATTTC*AAATTCAGTGGCTCAAGCTCGTTTTTCAGGTTT 1200

KU143789.2014 ACATGAT*GCCAACGATGCTGTGATTTC*AAATTCAGTGGCTCAAGCTCGTTTTTCAGGTCT 1200

KR817168.2014 ACATGAT*GCCAACGATGCTGTGATTTC*AAATTCAGTGGCTCAAGCTCGTTTTTCAGGTCT 1174

KY426696.2015 ACATGAT*GCCAACGATGCTGTGATTTC*AAATTCAGTGGCTCAAGCTCGTTTTTCAGGTCT 1200

KR105271.2014 ACATGAT*GCCAACGATGCTGTGATTTC*AAATTCAGTGGCTCAAGCTCGTTTTTCAGGTCT 1167

KY007522.2016 ACATGAT*GCCAACGATGCTGTGATTTC*AAATTCAGTGGCTCAAGCTCGTTTTTCAGGTCT 1180

KM034555.2014 ACATGAT*GCCAACGATGCTGTGATTTC*AAATTCAGTGGCTCAAGCTCGTTTTTCAGGTCT 1192

MH470381.2015 ACATGAT*GCCAACGATGCTGTGATTTC*AAATTCAGTGGCTCAAGCTCGTTTTTCAGGTCT 1164

MH470382.2015 ACATGAT*GCCAACGATGCTGTGATTTC*AAATTCAGTGGCTCAAGCTCGTTTTTCAGGTCT 1164

MF102255.2014 ACATGAT*GCCAACGATGCTGTGATTTC*AAATTCAGTGGCTCAAGCTCGTTTTTCAGGTCT 1177

KJ660348.2014 ACATGAT*GCCAACGATGCTGTGATTTC*AAATTCAGTGGCTCAAGCTCGTTTTTCAGGTCT 1200

KU143818.2014 ACATGAT*GCCAACGATGCTGTGATTTC*AAATTCAGTGGCTCAAGCTCGTTTTTCAGGTCT 1200

KT725333.2014 ACATGAT*GCCAACGATGCTGTGATTTC*AAATTCAGTGGCTCAAGCTCGTTTTTCAGGTCT 1165

KR819004.2014 GCATGATGCCAACGACGCTGTGATTTCAAATTCAGTGGCTCAAGCTCGTTTTTCAGGTTT 1147

KP271020.2014 GCATGATGCCAACGACGCTGTGATTTCAAATTCAGTGGCTCAAGCTCGTTTTTCAGGTTT 1147

KM519951.2014 GCATGATGCCAACGACGCTGTGATTTCAAATTCAGTGGCTCAAGCTCGTTTTTCAGGTTT 1197

MH733488.2018 GCATGATGCCAACGACGCTGTGATTTCAAATTCAGTGGCTCAAGCTCGTTTTTCAGGTTT 1191

MH733491.2018 GCATGATGCCAACGACGCTGTGATTTCAAATTCAGTGGCTCAAGCTCGTTTTTCAGGTTT 1186

MH733478.2018 GCATGATGCCAACGACGCTGTGATTTCAAATTCAGTGGCTCAAGCTCGTTTTTCAGGTTT 1189

MK007330.2018 GCATGAT*GCCAACGATGCTGTGATTTC*AAATTCAGTGGCTCAAGCTCGTTTTTCAGGTTT 1188

MK007344.2018 GCATGAT*GCCAACGATGCTGTGATTTC*AAATTCAGTGGCTCAAGCTCGTTTTTCAGGTTT 1182

************** ***************************************** *

AY142960.1976 ATTGATTGTCAAAACAGTACTTGATCATATCCTACAAAAGA*CAGAACGAGGAGTTCGTCT* 1260 *EBOV-NP-Rev Lau (22)*

KC242791.1977 ATTGATTGTCAAAACAGTACTTGATCATATCCTACAAAAGA*CAGAACGAGGAGTTCGTCT* 1260

AF499101.1976 ATTGATTGTCAAAACAGTACTTGATCATATCCTACAAAAGA*CAGAACGAGGAGTTCGTCT* 1260

KF113528.2003 ATTGATTGTCAAAACAGTCCTTGATCATATCCTACAAAAGA*CAGAACGAGGAGTTCGTCT* 1256

KC242800.2002 ATTGATTGTCAAAACAGTCCTTGATCATATCCTACAAAAGA*CAGAACGAGGAGTTCGTCT* 1260

KY471090.2001 ATTGATTGTCAAAACAGTCCTTGATCATATCCTACAAAAGA*CAGAACGAGGAGTTCGTCT* 1237

KY471092.2001 ATTGATTGTCAAAACAGTCCTTGATCATATCCTACAAAAGA*CAGAACGAGGAGTTCGTCT* 1237

MH481611.2017 ATTGATTGTCAAAACAGTACTTGATCATATCCTACAAAAGA*CAGAACGAGGAGTTCGTCT* 1226

MH613311.2017 ATTGATTGTCAAAACAGTACTTGATCATATCCTACAAAAGA*CAGAACGAGGAGTTCGTCT* 1205

KC242792.1994 ATTGATTGTCAAAACAGTACTTGATCATATCCTACAAAAGA*CAGAACGAGGAGTTCGTCT* 1260

KC242793.1996 ATTGATTGTCAAAACAGTACTTGATCATATCCTACAAAAGA*CAGAACGAGGAGTTCGTCT* 1260

KC242794.1996 ATTGATTGTCAAAACAGTACTTGATCATATCCTACAGAAGA*CAGAACGAGGAGTTCGTCT* 1260

MH121164.1995 ATTGATTGTCAAAACAGTACTTGATCATATCCTACAAAAGACAGAACGCGGAGTTCGTCT 1260

AY354458.1995 ATTGATTGTCAAAACAGTACTTGATCATATCCTACAAAAGACAGAACGCGGAGTTCGTCT 1260

KT762962.1995 ATTGATTGTCAAAACAGTACTTGATCATATCCTACAAAAGACAGAACGCGGAGTTCGTCT 1260

HQ613402.2008 ATTGATTGTCAAAACAGTACTTGATCATATCCTACAAAAGAC*AGAACGAGGAGTTCGTCT* 1179

KC242789.2007 ATTGATTGTCAAAACAGTACTTGATCATATCCTACAAAAGAC*AGAACGAGGAGTTCGTCT* 1260

HQ613403.2007 ATTGATTGTCAAAACAGTACTTGATCATATCCTACAAAAGAC*AGAACGAGGAGTTCGTCT* 1212

KC242785.2007 ATTGATTGTCAAAACAGTACTTGATCATATCCTACAAAAGAC*AGAACGAGGAGTTCGTCT* 1260

KC242790.2007 ATTGATTGTCAAAACAGTACTTGATCATATCCTACAAAAGAC*AGAACGAGGAGTTCGTCT* 1260

KU143789.2014 ATTGATTGTCAAAACAGTACTTGATCATATCCTACAAAAGAC*AGAACGAGGAGTTCGTCT* 1260

KR817168.2014 ATTGATTGTCAAAACAGTACTTGATCATATCCTACAAAAGAC*AGAACGAGGAGTTCGTCT* 1234

KY426696.2015 ATTGATTGTCAAAACAGTACTTGATCATATCCTACAAAAGAC*AGAACGAGGAGTTCGTCT* 1260

KR105271.2014 ATTGATTGTCAAAACAGTACTTGATCATATCCTACAAAAGAC*AGAACGAGGAGTTCGTCT* 1227

KY007522.2016 ATTGATTGTCAAAACAGTACTTGATCATATCCTACAAAAGAC*AGAACGAGGAGTTCGTCT* 1240

KM034555.2014 ATTGATTGTCAAAACAGTACTTGATCATATCCTACAAAAGAC*AGAACGAGGAGTTCGTCT* 1252

MH470381.2015 ATTGATTGTCAAAACAGTACTTGATCATATCCTACAAAAGAC*AGAACGAGGAGTTCGTCT* 1224

MH470382.2015 ATTGATTGTCAAAACAGTACTTGATCATATCCTACAAAAGAC*AGAACGAGGAGTTCGTCT* 1224

MF102255.2014 ATTGATTGTCAAAACAGTACTTGATCATATCCTACAAAAGAC*AGAACGAGGAGTTCGTCT* 1237

KJ660348.2014 ATTGATTGTCAAAACAGTACTTGATCATATCCTACAAAAGAC*AGAACGAGGAGTTCGTCT* 1260

KU143818.2014 ATTGATTGTCAAAACAGTACTTGATCATATCCTACAAAAGAC*AGAACGAGGAGTTCGTCT* 1260

KT725333.2014 ATTGATTGTCAAAACAGTACTTGATCATATCCTACAAAAGAC*AGAACGAGGAGTTCGTCT* 1225

KR819004.2014 ATTGATTGTCAAAACAGTACTTGATCATATCCTACAAAAGAC*AGAACGAGGAGTTCGTCT* 1207

KP271020.2014 ATTGATTGTCAAAACAGTACTTGATCATATCCTACAAAAGAC*AGAACGAGGAGTTCGTCT* 1207

KM519951.2014 ATTGATTGTCAAAACAGTACTTGATCATATCCTACAAAAGAC*AGAACGAGGAGTTCGTCT* 1257

MH733488.2018 ATTGATTGTCAAAACAGTACTTGATCATATCCTACAAAAGAC*AGAACGAGGAGTTCGTCT* 1251

MH733491.2018 ATTGATTGTCAAAACAGTACTTGATCATATCCTACAAAAGAC*AGAACGAGGAGTTCGTCT* 1246

MH733478.2018 ATTGATTGTCAAAACAGTACTTGATCATATCCTACAAAAGAC*AGAACGAGGAGTTCGTCT* 1249

MK007330.2018 ATTGATTGTCAAAACAGTACTTGATCATATCCTACAAAAGAC*AGAACGAGGAGTTCGTCT* 1248

MK007344.2018 ATTGATTGTCAAAACAGTACTTGATCATATCCTACAAAAGAC*AGAACGAGGAGTTCGTCT* 1242

****************** ***************** *********** ***********

AY142960.1976 *CC*ATCCTCTTGCAAGGACCGCCAAGGTAAAAAATGAGGTGAACTCCTTTAAGGCTGCACT 1320

KC242791.1977 *CC*ATCCTCTTGCAAGGACCGCCAAGGTAAAAAATGAGGTGAACTCCTTTAAGGCTGCACT 1320

AF499101.1976 *CC*ATCCTCTTGCAAGGACCGCCAAGGTAAAAAATGAGGTGAACTCCTTTAAGGCTGCACT 1320

KF113528.2003 *CC*ATCCTCTTGCAAGGACTGCCAAGGTAAAAAATGAGGTGAACTCCTTTAAGGCTGCACT 1316

KC242800.2002 *CC*ATCCTCTTGCAAGGACTGCCAAGGTAAAAAATGAGGTGAACTCCTTTAAGGCTGCACT 1320

KY471090.2001 *CC*ATCCTCTTGCAAGGACTGCCAAGGTAAAAAATGAGGTGAACTCCTTTAAGGCTGCACT 1297

KY471092.2001 *CC*ATCCTCTTGCAAGGACTGCCAAGGTAAAAAATGAGGTGAACTCCTTTAAGGCTGCACT 1297

MH481611.2017 *CC*ATCCTCTTGCAAGGACCGCCAAGGTTAAAAATGAGGTGAACTCCTTTAAGGCTGCACT 1286

MH613311.2017 *CC*ATCCTCTTGCAAGGACCGCCAAGGTTAAAAATGAGGTGAACTCCTTTAAGGCTGCACT 1265

KC242792.1994 *CC*ATCCTCTTGCAAGGACCGCTAAGGTAAAAAATGAGGTGAACTCCTTTAAGGCTGCACT 1320

KC242793.1996 *CC*ATCCTCTTGCAAGGACCGCCAAGGTAAAAAATGAGGTGAACTCCTTTAAGGCTGCACT 1320

KC242794.1996 *CC*ATCCTCTTGCAAGGACCGCCAAGGTAAAAAATGAGGTGAACTCCTTTAAGGCTGCACT 1320

MH121164.1995 CCATCCTCTTGCAAGGACCGCCAAGGTAAAAAATGAGGTGAACTCCTTTAAGGCTGCACT 1320

AY354458.1995 CCATCCTCTTGCAAGGACCGCCAAGGTAAAAAATGAGGTGAACTCCTTTAAGGCTGCACT 1320

KT762962.1995 *CC*ATCCTCTTGCAAGGACCGCCAAGGTAAAAAATGAGGTGAACTCCTTTAAGGCTGCACT 1320

HQ613402.2008 *CC*ATCCTCTTGCAAGGACCGCCAAGGTAAAAAATGAGGTGAACTCCTTTAAGGCTGCACT 1239

KC242789.2007 *CC*ATCCTCTTGCAAGGACCGCCAAGGTAAAAAATGAGGTGAACTCCTTTAAGGCTGCACT 1320

HQ613403.2007 *CC*ATCCTCTTGCAAGGACCGCCAAGGTAAAAAATGAGGTGAACTCCTTTAAGGCTGCACT 1272

KC242785.2007 *CC*ATCCTCTTGCAAGGACCGCCAAGGTAAAAAATGAGGTGAACTCCTTTAAGGCTGCACT 1320

KC242790.2007 *CC*ATCCTCTTGCAAGGACCGCCAAGGTAAAAAATGAGGTGAACTCCTTTAAGGCTGCACT 1320

KU143789.2014 *CC*ATCCTCTTGCAAGGACCGCCAAGGTAAAAAATGAGGTGAACTCCTTCAAGGCTGCACT 1320

KR817168.2014 *CC*ATCCTCTTGCAAGGACCGCCAAGGTAAAAAATGAGGTGAACTCCTTCAAGGCTGCACT 1294

KY426696.2015 *CC*ATCCTCTTGCAAGGACCGCCAAGGTAAAAAATGAGGTGAACTCCTTCAAGGCTGCACT 1320

KR105271.2014 *CC*ATCCTCTTGCAAGGACCGCCAAGGTAAAAAATGAGGTGAACTCCTTCAAGGCTGCACT 1287

KY007522.2016 *CC*ATCCTCTTGCAAGGACCGCCAAGGTAAAAAATGAGGTGAACTCCTTCAAGGCTGCACT 1300

KM034555.2014 *CC*ATCCTCTTGCAAGGACCGCCAAGGTAAAAAATGAGGTGAACTCCTTCAAGGCTGCACT 1312

MH470381.2015 *CC*ATCCTCTTGCAAGGACCGCCAAGGTAAAAAATGAGGTGAACTCCTTCAAGGCTGCACT 1284

MH470382.2015 *CC*ATCCTCTTGCAAGGACCGCCAAGGTAAAAAATGAGGTGAACTCCTTCAAGGCTGCACT 1284

MF102255.2014 *CC*ATCCTCTTGCAAGGACCGCCAAGGTAAAAAATGAGGTGAACTCCTTCAAGGCTGCACT 1297

KJ660348.2014 *CC*ATCCTCTTGCAAGGACCGCCAAGGTAAAAAATGAGGTGAACTCCTTCAAGGCTGCACT 1320

KU143818.2014 *CC*ATCCTCTTGCAAGGACCGCCAAGGTAAAAAATGAGGTGAACTCCTTCAAGGCTGCACT 1320

KT725333.2014 *CC*ATCCTCTTGCAAGGACCGCCAAGGTAAAAAATGAGGTGAACTCCTTCAAGGCTGCACT 1285

KR819004.2014 *CC*ATCCTCTTGCAAGGACCGCCAAGGTAAAAAATGAGGTGAACTCCTTTAAGGCTGCACT 1267

KP271020.2014 *CC*ATCCTCTTGCAAGGACCGCCAAGGTAAAAAATGAGGTGAACTCCTTTAAGGCTGCACT 1267

KM519951.2014 *CC*ATCCTCTTGCAAGGACCGCCAAGGTAAAAAATGAGGTGAACTCCTTTAAGGCTGCACT 1317

MH733488.2018 *CC*ATCCTCTTGCAAGGACCGCCAAGGTAAAAAATGAGGTGAACTCCTTTAAGGCTGCACT 1311

MH733491.2018 *CC*ATCCTCTTGCAAGGACCGCCAAGGTAAAAAATGAGGTGAACTCCTTTAAGGCTGCACT 1306

MH733478.2018 *CC*ATCCTCTTGCAAGGACCGCCAAGGTAAAAAATGAGGTGAACTCCTTTAAGGCTGCACT 1309

MK007330.2018 *CC*ATCCTCTTGCAAGGACCGCCAAGGTAAAAAATGAGGTGAACTCCTTTAAGGCTGCACT 1308

MK007344.2018 *CC*ATCCTCTTGCAAGGACCGCCAAGGTAAAAAATGAGGTGAACTCCTTTAAGGCTGCACT 1302

****************** ** ***** ******************** ***********

AY142960.1976 CAGCTCCCTGGCCAAGCATGGAGAGTATGCTCCTTTCGCCCGACTTTTGAACCTTTCTGG 1380

KC242791.1977 CAGCTCCCTGGCCAAGCATGGAGAGTATGCTCCTTTCGCCCGACTTTTGAACCTTTCTGG 1380

AF499101.1976 CAGCTCCCTGGCCAAGCATGGAGAGTATGCTCCTTTCGCCCGACTTTTGAACCTTTCTGG 1380

KF113528.2003 CAGCTCCCTGGCCAAGCATGGAGAGTATGCTCCTTTCGCCCGACTTTTGAACCTTTCTGG 1376

KC242800.2002 CAGCTCCCTGGCCAAGCATGGAGAGTATGCTCCTTTCGCCCGACTTTTGAACCTTTCTGG 1380

KY471090.2001 CAGCTCCCTGGCCAAGCATGGAGAGTATGCTCCTTTCGCCCGACTTTTGAACCTTTCTGG 1357

KY471092.2001 CAGCTCCCTGGCCAAGCATGGAGAGTATGCTCCTTTCGCCCGACTTTTGAACCTTTCTGG 1357

MH481611.2017 CAGCTCCCTGGCCAAGCATGGAGAGTATGCTCCTTTCGCCCGACTTTTGAACCTTTCTGG 1346

MH613311.2017 CAGCTCCCTGGCCAAGCATGGAGAGTATGCTCCTTTCGCCCGACTTTTGAACCTTTCTGG 1325

KC242792.1994 CAGCTCCCTGGCCAAGCATGGAGAGTATGCTCCTTTCGCCCGACTTTTGAACCTTTCTGG 1380

KC242793.1996 CAGCTCCCTGGCCAAGCATGGAGAGTATGCTCCTTTCGCCCGACTTTTGAACCTTTCTGG 1380

KC242794.1996 CAGCTCCCTGGCCAAGCATGGAGAGTATGCTCCTTTCGCCCGACTTTTGAACCTTTCTGG 1380

MH121164.1995 CAGCTCCCTGGCCAAGCATGGAGAGTATGCTCCTTTCGCCCGACTTTTGAACCTTTCTGG 1380

AY354458.1995 CAGCTCCCTGGCCAAGCATGGAGAGTATGCTCCTTTCGCCCGACTTTTGAACCTTTCTGG 1380

KT762962.1995 CAGCTCCCTGGCCAAGCATGGAGAGTATGCTCCTTTCGCCCGACTTTTGAACCTTTCTGG 1380

HQ613402.2008 CAGCTCCCTGGCCAAGCATGGAGAGTATGCTCCTTTCGCCCGACTTTTGAACCTCTCTGG 1299

KC242789.2007 CAGCTCCCTGGCCAAGCATGGAGAGTATGCTCCTTTCGCCCGACTTTTGAACCTCTCTGG 1380

HQ613403.2007 CAGCTCCCTGGCCAAGCATGGAGAGTATGCTCCTTTCGCCCGACTTTTGAACCTCTCTGG 1332

KC242785.2007 CAGCTCCCTGGCCAAGCATGGAGAGTATGCTCCTTTCGCCCGACTTTTGAACCTCTCTGG 1380

KC242790.2007 CAGCTCCCTGGCCAAGCATGGAGAGTATGCTCCTTTCGCCCGACTTTTGAACCTCTCTGG 1380

KU143789.2014 CAGCTCCCTGGCCAAGCATGGAGAGTATGCTCCTTTCGCCCGACTTTTGAACCTTTCTGG 1380

KR817168.2014 CAGCTCCCTGGCCAAGCATGGAGAGTATGCTCCTTTCGCCCGACTTTTGAACCTTTCTGG 1354

KY426696.2015 CAGCTCCCTGGCCAAGCATGGAGAGTATGCTCCTTTCGCCCGACTTTTGAACCTTTCTGG 1380

KR105271.2014 CAGCTCCCTGGCCAAGCATGGAGAGTATGCTCCTTTCGCCCGACTTTTGAACCTTTCTGG 1347

KY007522.2016 CAGCTCCCTGGCCAAGCATGGAGAGTATGCTCCTTTCGCCCGACTTCTGAACCTTTCTGG 1360

KM034555.2014 CAGCTCCCTGGCCAAGCATGGAGAGTATGCTCCTTTCGCCCGACTTTTGAACCTTTCTGG 1372

MH470381.2015 CAGCTCCCTGGCCAAGCATGGAGAGTATGCTCCTTTCGCCCGACTTTTGAACCTTTCTGG 1344

MH470382.2015 CAGCTCCCTGGCCAAGCATGGAGAGTATGCTCCTTTCGCCCGACTTTTGAACCTTTCTGG 1344

MF102255.2014 CAGCTCCCTGGCCAAGCATGGAGAGTATGCTCCTTTCGCCCGACTTTTGAACCTTTCTGG 1357

KJ660348.2014 CAGCTCCCTGGCCAAGCATGGAGAGTATGCTCCTTTCGCCCGACTTTTGAACCTTTCTGG 1380

KU143818.2014 CAGCTCCCTGGCCAAGCATGGAGAGTATGCTCCTTTCGCCCGACTTTTGAACCTTTCTGG 1380

KT725333.2014 CAGCTCCCTGGCCAAGCATGGAGAGTATGCTCCTTTCGCCCGACTTTTGAACCTTTCTGG 1345

KR819004.2014 CAGCTCCCTGGCCAAGCATGGAGAGTATGCTCCTTTCGCCCGACTTTTGAACCTTTCTGG 1327

KP271020.2014 CAGCTCCCTGGCCAAGCATGGAGAGTATGCTCCTTTCGCCCGACTTTTGAACCTTTCTGG 1327

KM519951.2014 CAGCTCCCTGGCCAAGCATGGAGAGTATGCTCCTTTCGCCCGACTTTTGAACCTTTCTGG 1377

MH733488.2018 CAGCTCCCTGGCCAAGCATGGAGAGTATGCTCCTTTCGCCCGACTTTTGAACCTTTCTGG 1371

MH733491.2018 CAGCTCCCTGGCCAAGCATGGAGAGTATGCTCCTTTCGCCCGACTTTTGAACCTTTCTGG 1366

MH733478.2018 CAGCTCCCTGGCCAAGCATGGAGAGTATGCTCCTTTCGCCCGACTTTTGAACCTTTCTGG 1369

MK007330.2018 CAGCTCCCTGGCCAAGCATGGAGAGTATGCTCCTTTTGCCCGACTTTTGAACCTTTCTGG 1368

MK007344.2018 CAGCTCCCTGGCCAAGCATGGAGAGTATGCTCCTTTTGCCCGACTTTTGAACCTTTCTGG 1362

************************************ ********* ******* *****

AY142960.1976 AGTAAATAATCTTGAGCATGGTCTTTTCCCTCAACTATCGGCAATTGCACTCGGAGTCGC 1440

KC242791.1977 AGTAAATAATCTTGAGCATGGTCTTTTCCCTCAACTATCGGCAATTGCACTCGGAGTCGC 1440

AF499101.1976 AGTAAATAATCTTGAGCATGGTCTTTTCCCTCAACTATCGGCAATTGCACTCGGAGTCGC 1440

KF113528.2003 AGTAAATAATCTTGAGCATGGTCTTTTCCCTCAACTATCGGCAATTGCACTCGGAGTCGC 1436

KC242800.2002 AGTAAATAATCTTGAGCATGGTCTTTTCCCTCAACTATCGGCAATTGCACTCGGAGTCGC 1440

KY471090.2001 AGTAAATAATCTTGAGCATGGTCTTTTCCCTCAACTATCGGCAATTGCACTCGGAGTCGC 1417

KY471092.2001 AGTAAATAATCTTGAGCATGGTCTTTTCCCTCAACTATCGGCAATTGCACTCGGAGTCGC 1417

MH481611.2017 AGTAAATAATCTTGAGCATGGTCTTTTCCCTCAACTATCGGCAATTGCACTCGGAGTCGC 1406

MH613311.2017 AGTAAATAATCTTGAGCATGGTCTTTTCCCTCAACTATCGGCAATTGCACTCGGAGTCGC 1385

KC242792.1994 AGTAAATAATCTTGAGCATGGTCTTTTCCCTCAACTATCAGCAATTGCACTCGGAGTCGC 1440

KC242793.1996 AGTAAATAATCTTGAGCATGGTCTTTTCCCTCAACTATCAGCAATTGCACTCGGAGTCGC 1440

KC242794.1996 AGTAAATAATCTTGAGCATGGTCTTTTCCCTCAACTATCAGCAATTGCACTCGGAGTCGC 1440

MH121164.1995 AGTAAATAATCTTGAGCATGGTCTTTTCCCTCAACTATCAGCAATTGCACTCGGAGTCGC 1440

AY354458.1995 AGTAAATAATCTTGAGCATGGTCTTTTCCCTCAACTATCAGCAATTGCACTCGGAGTCGC 1440

KT762962.1995 AGTAAATAATCTTGAGCATGGTCTTTTCCCTCAACTATCAGCAATTGCACTCGGAGTCGC 1440

HQ613402.2008 AGTAAATAATCTTGAGCATGGTCTTTTCCCTCAACTATCGGCAATTGCACTCGGAGTCGC 1359

KC242789.2007 AGTAAATAATCTTGAGCATGGTCTTTTCCCTCAACTATCGGCAATTGCACTCGGAGTCGC 1440

HQ613403.2007 AGTAAATAATCTTGAGCATGGTCTTTTCCCTCAACTATCGGCAATTGCACTCGGAGTCGC 1392

KC242785.2007 AGTAAATAATCTTGAGCATGGTCTTTTCCCTCAACTATCGGCAATTGCACTCGGAGTCGC 1440

KC242790.2007 AGTAAATAATCTTGAGCATGGTCTTTTCCCTCAACTATCGGCAATTGCACTCGGAGTCGC 1440

KU143789.2014 AGTAAATAATCTTGAGCATGGTCTTTTCCCTCAACTGTCGGCAATTGCACTCGGAGTCGC 1440

KR817168.2014 AGTAAATAATCTTGAGCATGGTCTTTTCCCTCAACTGTCGGCAATTGCACTCGGAGTCGC 1414

KY426696.2015 AGTAAATAATCTTGAGCATGGTCTTTTCCCTCAACTGTCGGCAATTGCACTCGGAGTCGC 1440

KR105271.2014 AGTAAATAATCTTGAGCATGGTCTTTTCCCTCAACTGTCGGCAATTGCACTCGGAGTCGC 1407

KY007522.2016 AGTAAATAATCTTGAGCATGGTCTTTTCCCTCAACTGTCGGCAATTGCACTCGGAGTCGC 1420

KM034555.2014 AGTAAATAATCTTGAGCATGGTCTTTTCCCTCAACTGTCGGCAATTGCACTCGGAGTCGC 1432

MH470381.2015 AGTAAATAATCTTGAGCATGGTCTTTTCCCTCAACTGTCGGCAATTGCACTCGGAGTCGC 1404

MH470382.2015 AGTAAATAATCTTGAGCATGGTCTTTTCCCTCAACTGTCGGCAATTGCACTCGGAGTCGC 1404

MF102255.2014 AGTAAATAATCTTGAGCATGGTCTTTTCCCTCAACTGTCGGCAATTGCACTCGGAGTCGC 1417

KJ660348.2014 AGTAAATAATCTTGAGCATGGTCTTTTCCCTCAACTGTCGGCAATTGCACTCGGAGTCGC 1440

KU143818.2014 AGTAAATAATCTTGAGCATGGTCTTTTCCCTCAACTGTCGGCAATTGCACTCGGAGTCGC 1440

KT725333.2014 AGTAAATAATCTTGAGCATGGTCTTTTCCCTCAACTGTCGGCAATTGCACTCGGAGTCGC 1405

KR819004.2014 AGTAAATAATCTTGAGCATGGTCTTTTCCCTCAACTATCAGCAATTGCACTCGGAGTCGC 1387

KP271020.2014 AGTAAATAATCTTGAGCATGGTCTTTTCCCTCAACTATCAGCAATTGCACTCGGAGTCGC 1387

KM519951.2014 AGTAAATAATCTTGAGCATGGTCTTTTCCCTCAACTATCAGCAATTGCACTCGGAGTCGC 1437

MH733488.2018 AGTAAATAATCTTGAGCATGGTCTTTTCCCTCAACTATCAGCAATTGCACTTGGAGTCGC 1431

MH733491.2018 AGTAAATAATCTTGAGCATGGTCTTTTCCCTCAACTATCAGCAATTGCACTTGGAGTCGC 1426

MH733478.2018 AGTAAATAATCTTGAGCATGGTCTTTTCCCTCAACTATCAGCAATTGCACTTGGAGTCGC 1429

MK007330.2018 AGTAAATAATCTTGAGCATGGTCTTTTCCCTCAACTATCGGCAATTGCACTCGGAGTCGC 1428

MK007344.2018 AGTAAATAATCTTGAGCATGGTCTTTTCCCTCAACTATCGGCAATTGCACTCGGAGTCGC 1422

************************************ ** *********** ********

AY142960.1976 CACAGCACACGGGAGTACCCTCGCAGGAGTAAATGTTGGAGAACAGTATCAACAACTCAG 1500

KC242791.1977 CACAGCACACGGGAGTACCCTCGCAGGAGTAAATGTTGGAGAACAGTATCAACAACTCAG 1500

AF499101.1976 CACAGCACACGGGAGTACCCTCGCAGGAGTAAATGTTGGAGAACAGTATCAACAACTCAG 1500

KF113528.2003 CACAGCACACGGGAGCACCCTCGCAGGAGTAAATGTTGGAGAACAGTATCAACAGCTCAG 1496

KC242800.2002 CACAGCACACGGGAGCACCCTCGCAGGAGTAAATGTTGGAGAACAGTATCAACAGCTCAG 1500

KY471090.2001 CACAGCACACGGGAGCACCCTCGCAGGAGTAAATGTTGGAGAACAGTATCAACAGCTCAG 1477

KY471092.2001 CACAGCACACGGGAGCACCCTCGCAGGAGTAAATGTTGGAGAACAGTATCAACAGCTCAG 1477

MH481611.2017 CACAGCACACGGGAGCACCCTCGCAGGAGTAAATGTTGGAGAACAGTATCAACAACTCAG 1466

MH613311.2017 CACAGCACACGGGAGCACCCTCGCAGGAGTAAATGTTGGAGAACAGTATCAACAACTCAG 1445

KC242792.1994 CACAGCACACGGGAGTACCCTCGCAGGAGTAAATGTTGGAGAACAGTATCAACAACTCAG 1500

KC242793.1996 CACAGCACACGGGAGTACCCTCGCAGGAGTAAATGTTGGAGAACAGTATCAACAACTCAG 1500

KC242794.1996 CACAGCACACGGGAGTACCCTCGCAGGAGTAAATGTTGGAGAACAGTATCAACAACTCAG 1500

MH121164.1995 CACAGCACACGGGAGTACCCTCGCAGGAGTAAATGTTGGAGAACAGTATCAACAACTCAG 1500

AY354458.1995 CACAGCACACGGGAGTACCCTCGCAGGAGTAAATGTTGGAGAACAGTATCAACAACTCAG 1500

KT762962.1995 CACAGCACACGGGAGTACCCTCGCAGGAGTAAATGTTGGAGAACAGTATCAACAACTCAG 1500

HQ613402.2008 CACAGCACACGGGAGCACCCTCGCAGGAGTAAATGTGGGAGAACAGTATCAACAGCTCAG 1419

KC242789.2007 CACAGCACACGGGAGCACCCTCGCAGGAGTAAATGTGGGAGAACAGTATCAACAGCTCAG 1500

HQ613403.2007 CACAGCACACGGGAGCACCCTCGCAGGAGTAAATGTGGGAGAACAGTATCAACAGCTCAG 1452

KC242785.2007 CACAGCACACGGGAGCACCCTCGCAGGAGTAAATGTGGGAGAACAGTATCAACAGCTCAG 1500

KC242790.2007 CACAGCACACGGGAGCACCCTCGCAGGAGTAAATGTGGGAGAACAGTATCAACAGCTCAG 1500

KU143789.2014 CACAGCCCACGGGAGCACCCTCGCAGGAGTAAATGTTGGAGAACAGTATCAACAGCTCAG 1500

KR817168.2014 CACAGCCCACGGGAGCACCCTCGCAGGAGTAAATGTTGGAGAACAGTATCAACAGCTCAG 1474

KY426696.2015 CACAGCCCACGGGAGCACCCTCGCAGGAGTAAATGTTGGAGAACAGTATCAACAGCTCAG 1500

KR105271.2014 CACAGCCCACGGGAGCACCCTCGCAGGAGTAAATGTTGGAGAACAGTATCAACAGCTCAG 1467

KY007522.2016 CACAGCCCACGGGAGCACCCTCGCAGGAGTAAATGTTGGAGAACAGTATCAACAGCTCAG 1480

KM034555.2014 CACAGCCCACGGGAGCACCCTCGCAGGAGTAAATGTTGGAGAACAGTATCAACAGCTCAG 1492

MH470381.2015 CACAGCCCACGGGAGCACCCTCGCAGGAGTAAATGTTGGAGAACAGTATCAACAGCTCAG 1464

MH470382.2015 CACAGCCCACGGGAGCACCCTCGCAGGAGTAAATGTTGGAGAACAGTATCAACAGCTCAG 1464

MF102255.2014 CACAGCCCACGGGAGCACCCTCGCAGGAGTAAATGTTGGAGAACAGTATCAACAGCTCAG 1477

KJ660348.2014 CACAGCCCACGGGAGCACCCTCGCAGGAGTAAATGTTGGAGAACAGTATCAACAGCTCAG 1500

KU143818.2014 CACAGCCCACGGGAGCACCCTCGCAGGAGTAAATGTTGGAGAACAGTATCAACAGCTCAG 1500

KT725333.2014 CACAGCCCACGGGAGCACCCTCGCAGGAGTAAATGTTGGAGAACAGTATCAACAGCTCAG 1465

KR819004.2014 CACAGCACACGGGAGTACCCTCGCAGGAGTAAATGTTGGAGAACAGTATCAACAACTCAG 1447

KP271020.2014 CACAGCACACGGGAGTACCCTCGCAGGAGTAAATGTTGGAGAACAGTATCAACAACTCAG 1447

KM519951.2014 CACAGCACACGGGAGTACCCTCGCAGGAGTAAATGTTGGAGAACAGTATCAACAACTCAG 1497

MH733488.2018 CACAGCACACGGGAGTACCCTTGCAGGAGTAAATGTTGGAGAACAGTATCAACAACTCAG 1491

MH733491.2018 CACAGCACACGGGAGTACCCTTGCAGGAGTAAATGTTGGAGAACAGTATCAACAACTCAG 1486

MH733478.2018 CACAGCACACGGGAGTACCCTTGCAGGAGTAAATGTTGGAGAACAGTATCAACAACTCAG 1489

MK007330.2018 CACAGCACACGGGAGTACCCTCGCAGGAGTAAATGTTGGAGAACAGTATCAACAACTCAG 1488

MK007344.2018 CACAGCACACGGGAGTACCCTCGCAGGAGTAAATGTTGGAGAACAGTATCAACAACTCAG 1482

****** ******** ***** ************** ***************** *****

AY142960.1976 AGAGGCTGCCACTGAGGCTGAGAAGCAACTCCAACAATATGCAGAGTCTCGCGAACTTGA 1560

KC242791.1977 AGAGGCTGCCACTGAGGCTGAGAAGCAACTCCAACAATATGCAGAGTCTCGCGAACTTGA 1560

AF499101.1976 AGAGGCTGCCACTGAGGCTGAGAAGCAACTCCAACAATATGCAGAGTCTCGCGAACTTGA 1560

KF113528.2003 AGAGGCTGCCACTGAAGCTGAGAAGCAACTCCAACAATATGCAGAATCTCGCGAACTTGA 1556

KC242800.2002 AGAGGCTGCCACTGAAGCTGAGAAGCAACTCCAACAATATGCAGAATCTCGCGAACTTGA 1560

KY471090.2001 AGAGGCTGCCACTGAAGCTGAGAAGCAACTCCAACAATATGCAGAATCTCGCGAACTTGA 1537

KY471092.2001 AGAGGCTGCCACTGAAGCTGAGAAGCAACTCCAACAATATGCAGAATCTCGCGAACTTGA 1537

MH481611.2017 AGAGGCTGCCACTGAGGCTGAGAAGCAACTCCAACAATATGCAGAGTCTCGCGAACTTGA 1526

MH613311.2017 AGAGGCTGCCACTGAGGCTGAGAAGCAACTCCAACAATATGCAGAGTCTCGCGAACTTGA 1505

KC242792.1994 AGAGGCTGCCACTGAGGCTGAGAAGCAACTCCAACAATACGCAGAGTCTCGCGAACTTGA 1560

KC242793.1996 AGAGGCTGCCACTGAGGCTGAGAAGCAACTCCAACAATACGCAGAGTCTCGCGAACTTGA 1560

KC242794.1996 AGAGGCTGCCACTGAGGCTGAGAAGCAACTCCAACAATACGCAGAGTCTCGCGAACTTGA 1560

MH121164.1995 AGAGGCTGCCACTGAGGCTGAGAAGCAACTCCAACAATACGCAGAGTCTCGCGAACTTGA 1560

AY354458.1995 AGAGGCTGCCACTGAGGCTGAGAAGCAACTCCAACAATACGCAGAGTCTCGCGAACTTGA 1560

KT762962.1995 AGAGGCTGCCACTGAGGCTGAGAAGCAACTCCAACAATACGCAGAGTCTCGCGAACTTGA 1560

HQ613402.2008 AGAGGCTGCCACTGAAGCTGAGAAGCAACTCCAACAATATGCAGAGTCTCGCGAACTTGA 1479

KC242789.2007 AGAGGCTGCCACTGAAGCTGAGAAGCAACTCCAACAATATGCAGAGTCTCGCGAACTTGA 1560

HQ613403.2007 AGAGGCTGCCACTGAAGCTGAGAAGCAACTCCAACAATATGCAGAGTCTCGCGAACTTGA 1512

KC242785.2007 AGAGGCTGCCACTGAAGCTGAGAAGCAACTCCAACAATATGCAGAGTCTCGCGAACTTGA 1560

KC242790.2007 AGAGGCTGCCACTGAAGCTGAGAAGCAACTCCAACAATATGCAGAGTCTCGCGAACTTGA 1560

KU143789.2014 AGAGGCAGCCACTGAGGCTGAGAAGCAACTCCAACAATATGCGGAGTCTCGTGAACTTGA 1560

KR817168.2014 AGAGGCAGCCACTGAGGCTGAGAAGCAACTCCAACAATATGCGGAGTCTCGTGAACTTGA 1534

KY426696.2015 AGAGGCAGCCACTGAGGCTGAGAAGCAACTCCAACAATATGCGGAGTCTCGTGAACTTGA 1560

KR105271.2014 AGAGGCAGCCACTGAGGCTGAGAAGCAACTCCAACAATATGCGGAGTCTCGTGAACTTGA 1527

KY007522.2016 AGAGGCAGCCACTGAGGCTGAGAAGCAACTCCAACAATATGCGGAGTCTCGTGAACTTGA 1540

KM034555.2014 AGAGGCAGCCACTGAGGCTGAGAAGCAACTCCAACAATATGCGGAGTCTCGTGAACTTGA 1552

MH470381.2015 AGAGGCAGCCACTGAGGCTGAGAAGCAACTCCAACAATATGCGGAGTCTCGTGAACTTGA 1524

MH470382.2015 AGAGGCAGCCACTGAGGCTGAGAAGCAACTCCAACAATATGCGGAGTCTCGTGAACTTGA 1524

MF102255.2014 AGAGGCAGCCACTGAGGCTGAGAAGCAACTCCAACAATATGCGGAGTCTCGTGAACTTGA 1537

KJ660348.2014 AGAGGCAGCCACTGAGGCTGAGAAGCAACTCCAACAATATGCGGAGTCTCGTGAACTTGA 1560

KU143818.2014 AGAGGCAGCCACTGAGGCTGAGAAGCAACTCCAACAATATGCGGAGTCTCGTGAACTTGA 1560

KT725333.2014 AGAGGCAGCCACTGAGGCTGAGAAGCAACTCCAACAATATGCGGAGTCTCGTGAACTTGA 1525

KR819004.2014 AGAGGCTGCCACTGAGGCTGAGAAGCAACTCCAACAATACGCAGAGTCTCGCGAACTTGA 1507

KP271020.2014 AGAGGCTGCCACTGAGGCTGAGAAGCAACTCCAACAATACGCAGAGTCTCGCGAACTTGA 1507

KM519951.2014 AGAGGCTGCCACTGAGGCTGAGAAGCAACTCCAACAATACGCAGAGTCTCGCGAACTTGA 1557

MH733488.2018 AGAGGCTGCCACTGAGGCTGAGAAGCAACTCCAACAATACGCAGAGTCTCGCGAACTTGA 1551

MH733491.2018 AGAGGCTGCCACTGAGGCTGAGAAGCAACTCCAACAATACGCAGAGTCTCGCGAACTTGA 1546

MH733478.2018 AGAGGCTGCCACTGAGGCTGAGAAGCAACTCCAACAATACGCAGAGTCTCGCGAACTTGA 1549

MK007330.2018 AGAGGCTGCCACTGAGGCTGAGAAACAACTCCAACAATATGCAGAGTCTCGCGAACTTGA 1548

MK007344.2018 AGAGGCTGCCACTGAGGCTGAGAAACAACTCCAACAATATGCAGAGTCTCGCGAACTTGA 1542

****** ******** ******** ************** ** ** ***** ********

AY142960.1976 CCATCTTGGACTTGATGATCAGGAAAAGAAAATTCTTATGAACTTCCATCAGAAAAAGAA 1620

KC242791.1977 CCATCTTGGACTTGATGATCAGGAAAAGAAAATTCTTATGAACTTCCATCAGAAAAAGAA 1620

AF499101.1976 CCATCTTGGACTTGATGATCAGGAAAAGAAAATTCTTATGAACTTCCATCAGAAAAAGAA 1620

KF113528.2003 CCATCTTGGACTTGATGATCAGGAAAAGAAAATTCTTATGAACTTCCATCAGAAAAAGAA 1616

KC242800.2002 CCATCTTGGACTTGATGATCAGGAAAAGAAAATTCTTATGAACTTCCATCAGAAAAAGAA 1620

KY471090.2001 CCATCTTGGACTTGATGATCAGGAAAAGAAAATTCTTATGAACTTCCATCAGAAAAAGAA 1597

KY471092.2001 CCATCTTGGACTTGATGATCAGGAAAAGAAAATTCTTATGAACTTCCATCAGAAAAAGAA 1597

MH481611.2017 CCATCTTGGACTTGATGATCAGGAAAAGAAGATTCTTATGAACTTCCATCAGAAAAAGAA 1586

MH613311.2017 CCATCTTGGACTTGATGATCAGGAAAAGAAGATTCTTATGAACTTCCATCAGAAAAAGAA 1565

KC242792.1994 CCATCTTGGACTTGATGATCAGGAAAAGAAAATTCTTATGAATTTCCATCAGAAAAAGAA 1620

KC242793.1996 CCATCTTGGACTTGATGATCAGGAAAAGAAAATTCTTATGAATTTCCATCAGAAAAAGAA 1620

KC242794.1996 CCATCTTGGACTTGATGATCAGGAAAAGAAAATTCTTATGAATTTCCATCAGAAAAAGAA 1620

MH121164.1995 CCATCTTGGACTTGATGATCAGGAAAAGAAAATTCTTATGAACTTCCATCAGAAAAAGAA 1620

AY354458.1995 CCATCTTGGACTTGATGATCAGGAAAAGAAAATTCTTATGAACTTCCATCAGAAAAAGAA 1620

KT762962.1995 CCATCTTGGACTTGATGATCAGGAAAAGAAAATTCTTATGAACTTCCATCAGAAAAAGAA 1620

HQ613402.2008 CCATCTTGGACTTGATGATCAGGAAAAGAAAATCCTTATGAACTTCCATCAGAAGAAGAA 1539

KC242789.2007 CCATCTTGGACTTGATGATCAGGAAAAGAAAATCCTTATGAACTTCCATCAGAAGAAGAA 1620

HQ613403.2007 CCATCTTGGACTTGATGATCAGGAAAAGAAAATCCTTATGAACTTCCATCAGAAGAAGAA 1572

KC242785.2007 CCATCTTGGACTTGATGATCAGGAAAAGAAAATCCTTATGAACTTCCATCAGAAGAAGAA 1620

KC242790.2007 CCATCTTGGACTTGATGATCAGGAAAAGAAAATCCTTATGAACTTCCATCAGAAGAAGAA 1620

KU143789.2014 CCATCTTGGACTTGATGATCAGGAAAAGAAAATTCTTATGAACTTCCATCAGAAAAAGAA 1620

KR817168.2014 CCATCTTGGACTTGATGATCAGGAAAAGAAAATTCTTATGAACTTCCATCAGAAAAAGAA 1594

KY426696.2015 CCATCTTGGACTTGATGATCAGGAAAAGAAAATTCTTATGAACTTCCATCAGAAAAAGAA 1620

KR105271.2014 CCATCTTGGACTTGATGATCAGGAAAAGAAAATTCTTATGAACTTCCATCAGAAAAAGAA 1587

KY007522.2016 CCATCTTGGACTTGATGATCAGGAAAAGAAAATTCTTATGAACTTCCATCAGAAAAAGAA 1600

KM034555.2014 CCATCTTGGACTTGATGATCAGGAAAAGAAAATTCTTATGAACTTCCATCAGAAAAAGAA 1612

MH470381.2015 CCATCTTGGACTTGATGATCAGGAAAAGAAAATTCTTATGAGCTTCCATCAGAAAAAGAA 1584

MH470382.2015 CCATCTTGGACTTGATGATCAGGAAAAGAAAATTCTTATGAGCTTCCATCAGAAAAAGAA 1584

MF102255.2014 CCATCTTGGACTTGATGATCAGGAAAAGAAAATTCTTATGAACTTCCATCAGAAAAAGAA 1597

KJ660348.2014 CCATCTTGGACTTGATGATCAGGAAAAGAAAATTCTTATGAACTTCCATCAGAAAAAGAA 1620

KU143818.2014 CCATCTTGGACTTGATGATCAGGAAAAGAAAATTCTTATGAACTTCCATCAGAAAAAGAA 1620

KT725333.2014 CCATCTTGGACTTGATGATCAGGAAAAGAAAATTCTTATGAACTTCCATCAGAAAAAGAA 1585

KR819004.2014 CCATCTTGGACTTGATGATCAGGAAAAGAAAATTCTCATGAACTTCCATCAGAAAAAGAA 1567

KP271020.2014 CCATCTTGGACTTGATGATCAGGAAAAGAAAATTCTCATGAACTTCCATCAGAAAAAGAA 1567

KM519951.2014 CCATCTTGGACTTGATGATCAGGAAAAGAAAATTCTCATGAACTTCCATCAGAAAAAGAA 1617

MH733488.2018 CCATCTTGGACTTGATGATCAGGAAAAGAAAATTCTTATGAACTTCCATCAGAAAAAGAA 1611

MH733491.2018 CCATCTTGGACTTGATGATCAGGAAAAGAAAATTCTTATGAACTTCCATCAGAAAAAGAA 1606

MH733478.2018 CCATCTTGGACTTGATGATCAGGAAAAGAAAATTCTTATGAACTTCCATCAGAAAAAGAA 1609

MK007330.2018 CCATCTTGGACTTGATGATCAGGAAAAGAAAATTCTTATGAACTTCCATCAGAAAAAGAA 1608

MK007344.2018 CCATCTTGGACTTGATGATCAGGAAAAGAAAATTCTTATGAACTTCCATCAGAAAAAGAA 1602

****************************** ** ** **** *********** *****

AY142960.1976 CGAAATCAGCTTCCAGCAAACAAACGCTATGGTAACTCTAAGAAAAGAGCGCCTGGCCAA 1680

KC242791.1977 CGAAATCAGCTTCCAGCAAACAAACGCTATGGTAACTCTAAGAAAAGAGCGCCTGGCCAA 1680

AF499101.1976 CGAAATCAGCTTCCAGCAAACAAACGCTATGGTAACTCTAAGAAAAGAGCGCCTGGCCAA 1680

KF113528.2003 CGAAATCAGCTTCCAGCAAACAAACGCTATGGTAACTCTAAGAAAAGAGCGCCTGGCCAA 1676

KC242800.2002 CGAAATCAGCTTCCAGCAAACAAACGCTATGGTAACTCTAAGAAAAGAGCGCCTGGCCAA 1680

KY471090.2001 CGAAATCAGCTTCCAGCAAACAAACGCTATGGTAACTCTAAGAAAAGAGCGCCTGGCCAA 1657

KY471092.2001 CGAAATCAGCTTCCAGCAAACAAACGCTATGGTAACTCTAAGAAAAGAGCGCCTGGCCAA 1657

MH481611.2017 CGAAATCAGCTTCCAGCAAACAAACGCTATGGTAACTCTAAGAAAAGAGCGCCTGGCCAA 1646

MH613311.2017 CGAAATCAGCTTCCAGCAAACAAACGCTATGGTAACTCTAAGAAAAGAGCGCCTGGCCAA 1625

KC242792.1994 CGAAATCAGCTTCCAGCAAACAAACGCTATGGTAACTCTAAAGAAAGAGCGCCTGGCCAA 1680

KC242793.1996 CGAAATCAGCTTCCAGCAAACAAACGCTATGGTAACTCTAAAGAAAGAGCGCCTGGCCAA 1680

KC242794.1996 CGAAATCAGCTTCCAGCAAACAAACGCTATGGTAACTCTAAGAAAAGAGCGCCTGGCCAA 1680

MH121164.1995 CGAAATCAGCTTCCAGCAAACAAACGCTATGGTAACTCTAAGAAAAGAGCGCCTGGCCAA 1680

AY354458.1995 CGAAATCAGCTTCCAGCAAACAAACGCTATGGTAACTCTAAGAAAAGAGCGCCTGGCCAA 1680

KT762962.1995 CGAAATCAGCTTCCAGCAAACAAACGCTATGGTAACTCTAAGAAAAGAGCGCCTGGCCAA 1680

HQ613402.2008 CGAAATCAGCTTCCAGCAAACAAACGCTATGGTAACTCTAAGAAAAGAGCGCCTGGCCAA 1599

KC242789.2007 CGAAATCAGCTTCCAGCAAACAAACGCTATGGTAACTCTAAGAAAAGAGCGCCTGGCCAA 1680

HQ613403.2007 CGAAATCAGCTTCCAGCAAACAAACGCTATGGTAACTCTAAGAAAAGAGCGCCTGGCCAA 1632

KC242785.2007 CGAAATCAGCTTCCAGCAAACAAACGCTATGGTAACTCTAAGAAAAGAGCGCCTGGCCAA 1680

KC242790.2007 CGAAATCAGCTTCCAGCAAACAAACGCTATGGTAACTCTAAGAAAAGAGCGCCTGGCCAA 1680

KU143789.2014 CGAAATCAGCTTCCAGCAAACAAACGCGATGGTAACTCTAAGAAAAGAGCGCCTGGCCAA 1680

KR817168.2014 CGAAATCAGCTTCCAGCAAACAAACGCGATGGTAACTCTAAGAAAAGAGCGCCTGGCCAA 1654

KY426696.2015 CGAAATCAGCTTCCAGCAAACAAACGCGATGGTAACTCTAAGAAAAGAGCGCCTGGCCAA 1680

KR105271.2014 CGAAATCAGCTTCCAGCAAACAAACGCGATGGTAACTCTAAGAAAAGAGCGCCTGGCCAA 1647

KY007522.2016 CGAAATCAGCTTCCAGCAAACAAACGCGATGGTAACTCTAAGAAAAGAGCGCCTGGCCAA 1660

KM034555.2014 CGAAATCAGCTTCCAGCAAACAAACGCGATGGTAACTCTAAGAAAAGAGCGCCTGGCCAA 1672

MH470381.2015 CGAAATCAGCTTCCAGCAAACAAACGCGATGGTAACTCTAAGAAAAGAGCGCCTGGCCAA 1644

MH470382.2015 CGAAATCAGCTTCCAGCAAACAAACGCGATGGTAACTCTAAGAAAAGAGCGCCTGGCCAA 1644

MF102255.2014 CGAAATCAGCTTCCAGCAAACAAACGCGATGGTAACTCTAAGAAAAGAGCGCCTGGCCAA 1657

KJ660348.2014 CGAAATCAGCTTCCAGCAAACAAACGCGATGGTAACTCTAAGAAAAGAGCGCCTGGCCAA 1680

KU143818.2014 CGAAATCAGCTTCCAGCAAACAAACGCGATGGTAACTCTAAGAAAAGAGCGCCTGGCCAA 1680

KT725333.2014 CGAAATCAGCTTCCAGCAAACAAACGCGATGGTAACTCTAAGAAAAGAGCGCCTGGCCAA 1645

KR819004.2014 CGAAATCAGCTTCCAGCAAACAAACGCTATGGTAACTCTAAGAAAAGAGCGCCTGGCCAA 1627

KP271020.2014 CGAAATCAGCTTCCAGCAAACAAACGCTATGGTAACTCTAAGAAAAGAGCGCCTGGCCAA 1627

KM519951.2014 CGAAATCAGCTTCCAGCAAACAAACGCTATGGTAACTCTAAGAAAAGAGCGCCTGGCCAA 1677

MH733488.2018 CGAAATCAGCTTCCAGCAAACAAACGCTATGGTAACTCTAAGAAAAGAGCGCCTGGCCAA 1671

MH733491.2018 CGAAATCAGCTTCCAGCAAACAAACGCTATGGTAACTCTAAGAAAAGAGCGCCTGGCCAA 1666

MH733478.2018 CGAAATCAGCTTCCAGCAAACAAACGCTATGGTAACTCTAAGAAAAGAGCGCCTGGCCAA 1669

MK007330.2018 CGAAATCAGCTTCCAGCAAACAAACGCTATGGTAACTCTAAGAAAAGAGCGCCTGGCCAA 1668

MK007344.2018 CGAAATCAGCTTCCAGCAAACAAACGCTATGGTAACTCTAAGAAAAGAGCGCCTGGCCAA 1662

*************************** ************* *****************

AY142960.1976 GCTGACAGAAGCTATCACTGCTGCGTCACTGCCCAAAACAAGTGGACATTACGATGATGA 1740

KC242791.1977 GCTGACAGAAGCTATCACTGCTGCGTCACTGCCCAAAACAAGTGGACATTACGATGATGA 1740

AF499101.1976 GCTGACAGAAGCTATCACTGCTGCGTCACTGCCCAAAACAAGTGGACATTACGATGATGA 1740

KF113528.2003 GCTGACAGAAGCTATCACTGCTGCATCACTGCCCAAAACAAGTGGACATTACGATGATGA 1736

KC242800.2002 GCTGACAGAAGCTATCACTGCTGCATCACTGCCCAAAACAAGTGGACCTTACGATGATGA 1740

KY471090.2001 GCTGACAGAAGCTATCACTGCTGCATCACTGCCCAAAACAAGTGGACCTTACGATGATGA 1717

KY471092.2001 GCTGACAGAAGCTATCACTGCTGCATCACTGCCCAAAACAAGTGGACCTTACGATGATGA 1717

MH481611.2017 GCTGACAGAAGCTATCACTGCTGCGTCACTGCCCAAAACAAGTGGACATTACGATGATGA 1706

MH613311.2017 GCTGACAGAAGCTATCACTGCTGCGTCACTGCCCAAAACAAGTGGACATTACGATGATGA 1685

KC242792.1994 GCTGACGGAAGCTATCACTGCTGCGTCACTGCCCAAAACAAGTGGACATTACGATGATGA 1740

KC242793.1996 GCTGACGGAAGCTATCACTGCTGCGTCACTGCCCAAAACAAGTGGACATTACGATGATGA 1740

KC242794.1996 GCTGACGGAAGCCATCACTGCTGCGTCACTGCCCAAAACAAGTGGACATTACGATGATGA 1740

MH121164.1995 GCTGACGGAAGCTATCACTGCTGCGTCACTGCCCAAAACAAGTGGACATTACGATGATGA 1740

AY354458.1995 GCTGACGGAAGCTATCACTGCTGCGTCACTGCCCAAAACAAGTGGACATTACGATGATGA 1740

KT762962.1995 GCTGACGGAAGCTATCACTGCTGCGTCACTGCCCAAAACAAGTGGACATTACGATGATGA 1740

HQ613402.2008 GCTGACAGAAGCTATCACCGCTGCGTCACTGCCCAAAACAAGTGGACATTACGATGATGA 1659

KC242789.2007 GCTGACAGAAGCTATCACCGCTGCGTCACTGCCCAAAACAAGTGGACATTACGATGATGA 1740

HQ613403.2007 GCTGACAGAAGCTATCACCGCTGCGTCACTGCCCAAAACAAGTGGACATTACGATGATGA 1692

KC242785.2007 GCTGACAGAAGCTATCACCGCTGCGTCACTGCCCAAAACAAGTGGACATTACGATGATGA 1740

KC242790.2007 GCTGACAGAAGCTATCACCGCTGCGTCACTGCCCAAAACAAGTGGACATTACGATGATGA 1740

KU143789.2014 GCTGACAGAAGCTATCACTGCTGCATCACTGCCCAAAACAAGTGGACATTACGATGATGA 1740

KR817168.2014 GCTGACAGAAGCTATCACTGCTGCATCACTGCCCAAAACAAGTGGACATTACGATGATGA 1714

KY426696.2015 GCTGACAGAAGCTATCACTGCTGCATCACTGCCCAAAACAAGTGGACATTACGATGATGA 1740

KR105271.2014 GCTGACAGAAGCTATCACTGCTGCATCACTGCCCAAAACAAGTGGACATTACGATGATGA 1707

KY007522.2016 GCTGACAGAAGCTATCACTGCTGCATCACTGCCCAAAACAAGTGGACATTACGATGATGA 1720

KM034555.2014 GCTGACAGAAGCTATCACTGCTGCATCACTGCCCAAAACAAGTGGACATTACGATGATGA 1732

MH470381.2015 GCTGACAGAAGCTATCACTGCTGCATCACTGCCCAAAACAAGTGGACATTACGATGATGA 1704

MH470382.2015 GCTGACAGAAGCTATCACTGCTGCATCACTGCCCAAAACAAGTGGACATTACGATGATGA 1704

MF102255.2014 GCTGACAGAAGCTATCACTGCTGCATCACTGCCCAAAACAAGTGGACATTACGATGATGA 1717

KJ660348.2014 GCTGACAGAAGCTATCACTGCTGCATCACTGCCCAAAACAAGTGGACATTACGATGATGA 1740

KU143818.2014 GCTGACAGAAGCTATCACTGCTGCATCACTGCCCAAAACAAGTGGACATTACGATGATGA 1740

KT725333.2014 GCTGACAGAAGCTATCACTGCTGCATCACTGCCCAAAACAAGTGGACATTACGATGATGA 1705

KR819004.2014 GCTGACGGAAGCTATCACTGCTGCGTCACTGCCCAAAACAAGTGGACATTACGATGATGA 1687

KP271020.2014 GCTGACGGAAGCTATCACTGCTGCGTCACTGCCCAAAACAAGTGGACATTACGATGATGA 1687

KM519951.2014 GCTGACGGAAGCTATCACTGCTGCGTCACTGCCCAAAACAAGTGGACATTACGATGATGA 1737

MH733488.2018 GCTGACGGAAGCTATCACTGCTGCGTCACTGCCCAAAACAAGTGGACATTACGATGATGA 1731

MH733491.2018 GCTGACGGAAGCTATCACTGCTGCGTCACTGCCCAAAACAAGTGGACATTACGATGATGA 1726

MH733478.2018 GCTGACGGAAGCTATCACTGCTGCGTCACTGCCCAAAACAAGTGGACATTACGATGATGA 1729

MK007330.2018 GCTGACAGAAGCTATCACTGCTGCGTCACTGCCCAAAACAAGTGGACATTACGATGATGA 1728

MK007344.2018 GCTGACAGAAGCTATCACTGCTGCGTCACTGCCCAAAACAAGTGGACATTACGATGATGA 1722

****** ***** ***** ***** ********************** ************

AY142960.1976 TGACGACATTCCCTTTCCAGGACCCATCAATGATGACGACAATCCTGGCCATCAAGATGA 1800

KC242791.1977 TGACGACATTCCCTTTCCAGGACCCATCAATGATGACGACAATCCTGGCCATCAAGATGA 1800

AF499101.1976 TGACGACATTCCCTTTCCAGGACCCATCAATGATGACGACAATCCTGGCCATCAAGATGA 1800

KF113528.2003 TGACGACATTCCCTTTCCAGGACCCATCAATGATGACGACAATCCTGGCCATCAAGATGA 1796

KC242800.2002 TGACGACATTCCCTTTCCAGGACCCATCAATGATGACGACAATCCTGGCCATCAAGATGA 1800

KY471090.2001 TGACGACATTCCCTTTCCAGGACCCATCAATGATGACGACAATCCTGGCCATCAAGATGA 1777

KY471092.2001 TGACGACATTCCCTTTCCAGGACCCATCAATGATGACGACAATCCTGGCCATCAAGATGA 1777

MH481611.2017 TGACGACATTCCCTTTCCAGGACCCATCAATGATGACGACAATCCTGGCCATCAAGATGA 1766

MH613311.2017 TGACGACATTCCCTTTCCAGGACCCATCAATGATGACGACAATCCTGGCCATCAAGATGA 1745

KC242792.1994 TGACGACATTCCCTTTCCAGGACCCATCAATGATGACGACAATCCTGGCCATCAAGATGA 1800

KC242793.1996 TGACGACATTCCCTTTCCAGGACCCATCAATGATGACGACAATCCTGGCCATCAAGATGA 1800

KC242794.1996 TGACGACATTCCCTTTCCAGGACCCATCAATGATGACGACAATCCTGGCCATCAAGATGA 1800

MH121164.1995 TGACGACATTCCCTTTCCAGGACCCATCAATGATGACGACAATCCTGGCCATCAAGATGA 1800

AY354458.1995 TGACGACATTCCCTTTCCAGGACCCATCAATGATGACGACAATCCTGGCCATCAAGATGA 1800

KT762962.1995 TGACGACATTCCCTTTCCAGGACCCATCAATGATGACGACAATCCTGGCCATCAAGATGA 1800

HQ613402.2008 TGACGACATTCCCTTTCCAGGACCTATCAATGATGACGACAATCCTGGCCATCAAGATGA 1719

KC242789.2007 TGACGACATTCCCTTTCCAGGACCTATCAATGATGACGACAATCCTGGCCATCAAGATGA 1800

HQ613403.2007 TGACGACATTCCCTTTCCAGGACCTATCAATGATGACGACAATCCTGGCCATCAAGATGA 1752

KC242785.2007 TGACGACATTCCCTTTCCAGGACCTATCAATGATGACGACAATCCTGGCCATCAAGATGA 1800

KC242790.2007 TGACGACATTCCCTTTCCAGGACCTATCAATGATGACGACAATCCTGGCCATCAAGATGA 1800

KU143789.2014 TGACGACATTCCCTTTCCAGGACCCATCAATGATGACGACAATCCTGGCCATCAAGATGA 1800

KR817168.2014 TGACGACATTCCCTTTCCAGGACCCATCAATGATGACGACAATCCTGGCCATCAAGATGA 1774

KY426696.2015 TGACGACATTCCCTTTCCAGGACCCATCAATGATGACGACAATCCTGGCCATCAAGATGA 1800

KR105271.2014 TGACGACATTCCCTTTCCAGGACCCATCAATGATGACGACAATCCTGGCCATCAAGATGA 1767

KY007522.2016 TGACGACATTCCCTTTCCAGGACCCATCAATGATGACGACAATCCTGGCCATCAAGATGA 1780

KM034555.2014 TGACGACATTCCCTTTCCAGGACCCATCAATGATGACGACAATCCTGGCCATCAAGATGA 1792

MH470381.2015 TGACGACATTCCCTTTCCAGGACCCATCAATGATGACGACAATCCTGGCCATCAAGATGA 1764

MH470382.2015 TGACGACATTCCCTTTCCAGGACCCATCAATGATGACGACAATCCTGGCCATCAAGATGA 1764

MF102255.2014 TGACGACATTCCCTTTCCAGGACCCATCAATGATGACGACAATCCTGGCCATCAAGATGA 1777

KJ660348.2014 TGACGACATTCCCTTTCCAGGACCCATCAATGATGACGACAATCCTGGCCATCAAGATGA 1800

KU143818.2014 TGACGACATTCCCTTTCCAGGACCCATCAATGATGACGACAATCCTGGCCATCAAGATGA 1800

KT725333.2014 TGACGACATTCCCTTTCCAGGACCCATCAATGATGACGACAATCCTGGCCATCAAGATGA 1765

KR819004.2014 TGACGACATTCCCTTTCCAGGGCCCATCAATGATGACGACAATCCTGGCCATCAAGATGA 1747

KP271020.2014 TGACGACATTCCCTTTCCAGGGCCCATCAATGATGACGACAATCCTGGCCATCAAGATGA 1747

KM519951.2014 TGACGACATTCCCTTTCCAGGGCCCATCAATGATGACGACAATCCTGGCCATCAAGATGA 1797

MH733488.2018 TGACGACATTCCCTTTCCAGGGCCCATCAATGATGACGACAATCCTGGCCATCAAGATGA 1791

MH733491.2018 TGACGACATTCCCTTTCCAGGGCCCATCAATGATGACGACAATCCTGGCCATCAAGATGA 1786

MH733478.2018 TGACGACATTCCCTTTCCAGGGCCCATCAATGATGACGACAATCCTGGCCATCAAGATGA 1789

MK007330.2018 TGACGACATTCCTTTTCCAGGACCCATCAATGATGACGACAATCCTGGCCATCAAGATGA 1788

MK007344.2018 TGACGACATTCCTTTTCCAGGACCCATCAATGATGACGACAATCCTGGCCATCAAGATGA 1782

************ ******** ** ***********************************

AY142960.1976 TGATCCGACTGACTCACAGGATACGACCATTCCCGATGTGGTGGTTGATCCCGATGATGG 1860

KC242791.1977 TGATCCGACTGACTCACAGGATACGACCATTCCCGATGTGGTGGTTGATCCCGATGATGG 1860

AF499101.1976 TGATCCGACTGACTCACAGGATACGACCATTCCCGATGTGGTGGTTGATCCCGATGATGG 1860

KF113528.2003 TGATCCGACTGACTCACAGGATACGACCATTCCCGATGTGGTGGTTGATCCCGATGATGG 1856

KC242800.2002 TGATCCGACTGACTCACAGGATACGACCATTCCCGATGTGGTGGTTGATCCCGATGATGG 1860

KY471090.2001 TGATCCGACTGACTCACAGGATACGACCATTCCCGATGTGGTGGTTGATCCCGATGATGG 1837

KY471092.2001 TGATCCGACTGACTCACAGGATACGACCATTCCCGATGTGGTGGTTGATCCCGATGATGG 1837

MH481611.2017 TGATCCGACTGACTCACAGGATACGACCATTCCCGATGTAGTGGTTGATCCCGATGATGG 1826

MH613311.2017 TGATCCGACTGACTCACAGGATACGACCATTCCCGATGTAGTGGTTGATCCCGATGATGG 1805

KC242792.1994 TGATCCGACTGACTCACAGGATACGACCATTCCCGATGTGGTGGTTGATCCCGATGATGG 1860

KC242793.1996 TGATCCGACTGACTCACAGGATACGACCATTCCCGATGTGGTGGTTGATCCCGATGATGG 1860

KC242794.1996 TGATCCGACTGACTCACAGGATACGACCATTCCCGATGTGGTGGTTGATCCCGATGATGG 1860

MH121164.1995 TGATCCAACTGACTCACAAGATACGACCATTCCTGATGTGGTGGTTGATCCCGATGATGG 1860

AY354458.1995 TGATCCAACTGACTCACAAGATACGACCATTCCTGATGTGGTGGTTGATCCCGATGATGG 1860

KT762962.1995 TGATCCAACTGACTCACAAGATACGACCATTCCTGATGTGGTGGTTGATCCCGATGATGG 1860

HQ613402.2008 TGATCCGACTGACTCACAGGATACGACCATTCCCGATGTGGTAGTTGATCCCGATGATGG 1779

KC242789.2007 TGATCCGACTGACTCACAGGATACGACCATTCCCGATGTGGTAGTTGATCCCGATGATGG 1860

HQ613403.2007 TGATCCGACTGACTCACAGGATACGACCATTCCCGATGTGGTAGTTGATCCCGATGATGG 1812

KC242785.2007 TGATCCGACTGACTCACAGGATACGACCATTCCCGATGTGGTAGTTGATCCCGATGATGG 1860

KC242790.2007 TGATCCGACTGACTCACAGGATACGACCATTCCCGATGTGGTAGTTGATCCCGATGATGG 1860

KU143789.2014 TGATCCGACTGACTCACAGGATACGACCATTCCCGATGTGGTAGTTGACCCCGATGATGG 1860

KR817168.2014 TGATCCGACTGACTCACAGGATACGACCATTCCCGATGTGGTAGTTGACCCCGATGATGG 1834

KY426696.2015 TGATCCGACTGACTCACAGGATACGACCATTCCCGATGTGGTAGTTGACCCCGATGATGG 1860

KR105271.2014 TGATCCGACTGACTCACAGGATACGACCATTCCCGATGTGGTAGTTGACCCCGATGATGG 1827

KY007522.2016 TGATCCGACTGACTCACAGGATACGACCATTCCCGATGTGGTAGTTGACCCCGATGATGG 1840

KM034555.2014 TGATCCGACTGACTCACAGGATACGACCATTCCCGATGTGGTAGTTGACCCCGATGATGG 1852

MH470381.2015 TGATCCGACTGACTCACAGGATACGACCATTCCCGATGTGGTAGTTGACCCCGATGATGG 1824

MH470382.2015 TGATCCGACTGACTCACAGGATACGACCATTCCCGATGTGGTAGTTGACCCCGATGATGG 1824

MF102255.2014 TGATCCGACTGACTCACAGGATACGACCATTCCCGATGTGGTAGTTGATCCCGATGATGG 1837

KJ660348.2014 TGATCCGACTGACTCACAGGATACGACCATTCCCGATGTGGTAGTTGATCCCGATGATGG 1860

KU143818.2014 TGATCCGACTGACTCACAGGATACGACCATTCCCGATGTGGTAGTTGACCCCGATGATGG 1860

KT725333.2014 TGATCCGACTGACTCACAGGATACGACCATTCCCGATGTGGTAGTTGACCCCGATGATGG 1825

KR819004.2014 TGATCCGACTGACTCACAGGATACGACCATTCCCGATGTGGTGGTTGATCCCGATGATGG 1807

KP271020.2014 TGATCCGACTGACTCACAGGATACGACCATTCCCGATGTGGTGGTTGATCCCGATGATGG 1807

KM519951.2014 TGATCCGACTGACTCACAGGATACGACCATTCCCGATGTGGTGGTTGATCCCGATGATGG 1857

MH733488.2018 TGATCCGACTGACTCACAGGATACGACCATTCCCGATGTGGTGGTTGATCCCGATGATGG 1851

MH733491.2018 TGATCCGACTGACTCACAGGATACGACCATTCCCGATGTGGTGGTTGATCCCGATGATGG 1846

MH733478.2018 TGATCCGACTGACTCACAGGATACGACCATTCCCGATGTGGTGGTTGATCCCGATGATGG 1849

MK007330.2018 TGATCCGACTGACTCACAGGATACGACCATTCCCGATGTAGTGATTGATCCCGATGATGG 1848

MK007344.2018 TGATCCGACTGACTCACAGGATACGACCATTCCCGATGTAGTGATTGATCCCGATGATGG 1842

****** *********** ************** ***** ** **** ***********

AY142960.1976 AAGCTACGGCGAATACCAGAGTTACTCGGAAAACGGCATGAATGCACCAGATGACTTGGT 1920

KC242791.1977 AAGCTACGGCGAATACCAGAGTTACTCGGAAAACGGCATGAATGCACCAGATGACTTGGT 1920

AF499101.1976 AAGCTACGGCGAATACCAGAGTTACTCGGAAAACGGCATGAATGCACCAGATGACTTGGT 1920

KF113528.2003 AAGCTACGGCGAATACCAGAGTTACTCGGAAAACGGCATGAATGCACCAGATGACTTGGT 1916

KC242800.2002 AAGCTACGGCGAATACCAGAGTTACTCGGAAAACGGCATGAATGCACCAGATGACTTGGT 1920

KY471090.2001 AAGCTACGGCGAATACCAGAGTTACTCGGAAAACGGCATGAATGCACCAGATGACTTGGT 1897

KY471092.2001 AAGCTACGGCGAATACCAGAGTTACTCGGAAAACGGCATGAATGCACCAGATGACTTGGT 1897

MH481611.2017 AAGCTACGGCGAATACCAGAGTTACTCGGAAAACGGCATGAATGCACCAGATGACTTGGT 1886

MH613311.2017 AAGCTACGGCGAATACCAGAGTTACTCGGAAAACGGCATGAATGCACCAGATGACTTGGT 1865

KC242792.1994 AAGCTACGGCGAATACCAGAGTTACTCGGAAAACGGCATGAATGCACCAGATGACTTGAT 1920

KC242793.1996 AAGCTACGGCGAATACCAGAGTTACTCGGAAAACGGCATGAATGCACCAGATGATTTGAT 1920

KC242794.1996 AAGCTACGGCGAATACCAGAGTTACTCGGAAAACGGCATGAATGCACCAGATGACTTGGT 1920

MH121164.1995 AAGCTACGGCGAATACCAGAGTTACTCGGAAAACGGCATGAATGCACCAGATGACTTGGT 1920

AY354458.1995 AAGCTACGGCGAATACCAGAGTTACTCGGAAAACGGCATGAATGCACCAGATGACTTGGT 1920

KT762962.1995 AAGCTACGGCGAATACCAGAGTTACTCGGAAAACGGCATGAATGCACCAGATGACTTGGT 1920

HQ613402.2008 AAGCTACGGCGAATACCAGAGTTACTCGGAAAACGGTATGAATGCACCAGATGACTTGGT 1839

KC242789.2007 AAGCTACGGCGAATACCAGAGTTACTCGGAAAACGGTATGAATGCACCAGATGACTTGGT 1920

HQ613403.2007 AAGCTACGGCGAATACCAGAGTTACTCGGAAAACGGTATGAATGCACCAGATGACTTGGT 1872

KC242785.2007 AAGCTACGGCGAATACCAGAGTTACTCGGAAAACGGTATGAATGCACCAGATGACTTGGT 1920

KC242790.2007 AAGCTACGGCGAATACCAGAGTTACTCGGAAAACGGTATGAATGCACCAGATGACTTGGT 1920

KU143789.2014 AGGCTACGGCGAATACCAAAGTTACTCGGAAAACGGCATGAGTGCACCAGATGACTTGGT 1920

KR817168.2014 AGGCTACGGCGAATACCAAAGTTACTCGGAAAACGGCATGAGTGCACCAGATGACTTGGT 1894

KY426696.2015 AGGCTACGGCGAATACCAAAGTTACTCGGAAAACGGCATGAGTGCACCAGATGACTTGGT 1920

KR105271.2014 AGGCTACGGCGAATACCAAAGTTACTCGGAAAACGGCATGAGTGCACCAGATGACTTGGT 1887

KY007522.2016 AGGCTACGGCGAATACCAAAGTTACTCGGAAAACGGCATGAGTGCACCAGATGACTTGGT 1900

KM034555.2014 AGGCTACGGCGAATACCAAAGTTACTCGGAAAACGGCATGAGTGCACCAGATGACTTGGT 1912

MH470381.2015 AGGCTACGGCGAATACCAAAGTTACTCGGAAAACGGCATGAGTGCACCAGATGACTTGGT 1884

MH470382.2015 AGGCTACGGCGAATACCAAAGTTACTCGGAAAACGGCATGAGTGCACCAGATGACTTGGT 1884

MF102255.2014 AGGCTACGGCGAATACCAAAGTTACTCGGAAAACGGCATGAGTGCACCAGATGACTTGGT 1897

KJ660348.2014 AGGCTACGGCGAATACCAAAGTTACTCGGAAAACGGCATGAGTGCACCAGATGACTTGGT 1920

KU143818.2014 AGGCTACGGCGAATACCAAAGTTACTCGGAAAACGGCATGAGTGCACCAGATGACTTGGT 1920

KT725333.2014 AGGCTACGGCGAATACCAAAGTTACTCGGAAAACGGCATGAGTGCACCAGATGACTTGGT 1885

KR819004.2014 AAGCTACGGCGAATACCAGAGTTACTCGGAAAACGGCATGAATGCACCAGATGACTTGGT 1867

KP271020.2014 AAGCTACGGCGAATACCAGAGTTACTCGGAAAACGGCATGAATGCACCAGATGACTTGGT 1867

KM519951.2014 AAGCTACGGCGAATACCAGAGTTACTCGGAAAACGGCATGAATGCACCAGATGACTTGGT 1917

MH733488.2018 AAGCTACGGCGAATACCAGAGTTACTCGGAAAACGGCATGAATGCACCAGATGACTTGGT 1911

MH733491.2018 AAGCTACGGCGAATACCAGAGTTACTCGGAAAACGGCATGAATGCACCAGATGACTTGGT 1906

MH733478.2018 AAGCTACGGCGAATACCAGAGTTACTCGGAAAACGGCATGAATGCACCAGATGACTTGGT 1909

MK007330.2018 AAGTTACGGCGAATACCAGAGTTATTCGGAAAACGGCATGAGTGCACCAGATGATTTGGT 1908

MK007344.2018 AAGTTACGGCGAATACCAGAGTTATTCGGAAAACGGCATGAGTGCACCAGATGATTTGGT 1902

* * ************** ***** *********** **** ************ *** *

AY142960.1976 CCTATTCGATCTAGACGAGGACGACGAGGACACTAAGCCAGTGCCTAATAGATCGACCAA 1980

KC242791.1977 CCTATTCGATCTAGACGAGGACGACGAGGACACTAAGCCAGTGCCTAATAGATCGACCAA 1980

AF499101.1976 CCTATTCGATCTAGACGAGGACGACGAGGACACTAAGCCAGTGCCTAATAGATCGACCAA 1980

KF113528.2003 CCTATTCGATCTAGACGAGGACGACGAGGACACTAAGCCAGTGCCTAACAGATTGACCAA 1976

KC242800.2002 CCTATTCGATCTAGACGAGGACGACGAGGACACTAAGCCAGTGCCTAACAGATTGACCAA 1980

KY471090.2001 CCTATTCGATCTAGACGAGGACGACGAGGACACTAAGCCAGTGCCTAACAGATTGACCAA 1957

KY471092.2001 CCTATTCGATCTAGACGAGGACGACGAGGACACTAAGCCAGTGCCTAACAGATTGACCAA 1957

MH481611.2017 CCTATTTGATTTAGACGAGGACGACGAGGACACTAAGCCAGTGCCCAATAGATCGACCAA 1946

MH613311.2017 CCTATTTGATTTAGACGAGGACGACGAGGACACTAAGCCAGTGCCCAATAGATCGACCAA 1925

KC242792.1994 CCTATTCGATCTAGACGAGGACGATGAGGACACTAAGCCAGTGCCTAATAGATCGACCAA 1980

KC242793.1996 CCTATTCGATCTAGACGAGGACGATGAGGACACTAAGCCAGTGCCTAATAGATCGACCAA 1980

KC242794.1996 CCTATTCGATCTAGACGAGGACGATGAGGACACTAAGCCAGTGCCTAATAGATCGACCAA 1980

MH121164.1995 CCTATTCGATCTAGACGAGGACGATGAGGACACTAAGCCAGTGCCTAATAGATCAACCAA 1980

AY354458.1995 CCTATTCGATCTAGACGAGGACGATGAGGACACTAAGCCAGTGCCTAATAGATCAACCAA 1980

KT762962.1995 CCTATTCGATCTAGACGAGGACGATGAGGACACTAAGCCAGTGCCTAATAGATCAACCAA 1980

HQ613402.2008 CCTATTCGATCTAGACGAGGACGACGAGGACACTAAGCCAGTGCCTAACAGATCGACCAA 1899

KC242789.2007 CCTATTCGATCTAGACGAGGACGACGAGGACACTAAGCCAGTGCCTAACAGATCGACCAA 1980

HQ613403.2007 CCTATTCGATCTAGACGAGGACGACGAGGACACTAAGCCAGTGCCTAACAGATCGACCAA 1932

KC242785.2007 CCTATTCGATCTAGACGAGGACGACGAGGACACTAAGCCAGTGCCTAACAGATCGACCAA 1980

KC242790.2007 CCTATTCGATCTAGACGAGGACGACGAGGACACTAAGCCAGTGCCTAACAGATCGACCAA 1980

KU143789.2014 CCTATTCGATCTAGACGAGGACGACGAGGACACCAAGCCAGTGCCTAACAGATCGACCAA 1980

KR817168.2014 CCTATTCGATCTAGACGAGGACGACGAGGACACCAAGCCAGTGCCTAACAGATCGACCAA 1954

KY426696.2015 CCTATTCGATCTAGACGAGGACGACGAGGACACCAAGCCAGTGCCTAACAGATCGACCAA 1980

KR105271.2014 CCTATTCGATCTAGACGAGGACGACGAGGACACCAAGCCAGTGCCTAACAGATCGACCAA 1947

KY007522.2016 CCTATTTGATCTAGACGAGGACGACGAGGACACCAAGCCAGTGCCTAACAGATCGACCAA 1960

KM034555.2014 CCTATTCGATCTAGACGAGGACGACGAGGACACCAAGCCAGTGCCTAACAGATCGACCAA 1972

MH470381.2015 CCTATTCGATCTAGACGAGGACGACGAGGACACCAAGCCAGTGCCTAACAGATCGACCAA 1944

MH470382.2015 CCTATTCGATCTAGACGAGGACGACGAGGACACCAAGCCAGTGCCTAACAGATCGACCAA 1944

MF102255.2014 CCTATTCGATCTAGACGAGGACGACGAGGACACCAAGCCAGTGCCTAACAGATCGACCAA 1957

KJ660348.2014 CCTATTCGATCTAGACGAGGACGACGAGGACACCAAGCCAGTGCCTAACAGATCGACCAA 1980

KU143818.2014 CCTATTCGATCTAGACGAGGACGACGAGGACACCAAGCCAGTGCCTAACAGATCGACCAA 1980

KT725333.2014 CCTATTCGATCTAGACGAGGACGACGAGGACACCAAGCCAGTGCCTAACAGATCGACCAA 1945

KR819004.2014 CCTATTCGATCTAGACGAGGACGATGAGGACACTAAGCCAGTGCCTAATAGATCGACCAA 1927

KP271020.2014 CCTATTCGATCTAGACGAGGACGATGAGGACACTAAGCCAGTGCCTAATAGATCGACCAA 1927

KM519951.2014 CCTATTCGATCTAGACGAGGACGATGAGGACACTAAGCCAGTGCCTAATAGATCGACCAA 1977

MH733488.2018 CCTATTCGATCTAGACGAGGACGATGAGGACACTAAGCCAGTGCCTAATAGATCGACCAA 1971

MH733491.2018 CCTATTCGATCTAGACGAGGACGATGAGGACACTAAGCCAGTGCCTAATAGATCGACCAA 1966

MH733478.2018 CCTATTCGATCTAGACGAGGACGATGAGGACACTAAGCCAGTGCCTAATAGATCGACCAA 1969

MK007330.2018 CCTATTTGATCTAGACGAGGACGACGAGGACACTAAGCCAGTGCCTAATAGATCAATCAA 1968

MK007344.2018 CCTATTTGATCTAGACGAGGACGACGAGGACACTAAGCCAGTGCCTAATAGATCAATCAA 1962

****** *** ************* ******** *********** ** **** * ***

AY142960.1976 GGGTGGACAACAGAAGAACAGTCAAAAGGGCCAGCATATAGAGGGCAGACAGACACAATC 2040

KC242791.1977 GGGTGGACAACAGAAGAACAGTCAAAAGGGCCAGCATATAGAGGGCAGACAGACACAATC 2040

AF499101.1976 GGGTGGACAACAGAAGAACAGTCAAAAGGGCCAGCATATAGAGGGCAGACAGACACAATC 2040

KF113528.2003 GGGTGGACAACAGAAAAACAGTCAAAAGGGCCAGCATACAGAGGGCAGACAGACACAATC 2036

KC242800.2002 GGGTGGACAACAGAAAAACAGTCAAAAGGGCCAGCATACAGAGGGCAGACAGACACAATC 2040

KY471090.2001 GGGTGGACAACAGAAAAACAGTCAAAAGGGCCAGCATACAGAGGGCAGACAGACACAATC 2017

KY471092.2001 GGGTGGACAACAGAAAAACAGTCAAAAGGGCCAGCATACAGAGGGCAGACAGACACAATC 2017

MH481611.2017 GGGTGGACAACAGAAAAACAGTCAAAAGGGCCAGCATACAGAGGGCAGACAGACACAATC 2006

MH613311.2017 GGGTGGACAACAGAAAAACAGTCAAAAGGGCCAGCATACAGAGGGCAGACAGACACAATC 1985

KC242792.1994 GGGTGGACAACAGAAAAACAGTCAAAAGGGCCAGCATACAGAGGGCAGACAGACACAATC 2040

KC242793.1996 GGGTGGACAACAGAAAAACAGTCAAAAGGGCCAGCATACAGAGGGCAGACAGACACAATC 2040

KC242794.1996 GGGTGGACAACAGAAAAACAGTCAAAAGGGCCAGCATACAGAGGGCAGACAGACACAATC 2040

MH121164.1995 GGGTGGACAACAGAAAAACAGTCAAAAGGGCCAGCATACAGAGGGCAGACAGACACAATC 2040

AY354458.1995 GGGTGGACAACAGAAAAACAGTCAAAAGGGCCAGCATACAGAGGGCAGACAGACACAATC 2040

KT762962.1995 GGGTGGACAACAGAAAAACAGTCAAAAGGGCCAGCATACAGAGGGCAGACAGACACAATC 2040

HQ613402.2008 GGGTGAACAACAGAAAAACAGTCAAAAGGGCCAGCATACAGAGGGCAGACAGACACAATC 1959

KC242789.2007 GGGTGAACAACAGAAAAACAGTCAAAAGGGCCAGCATACAGAGGGCAGACAGACACAATC 2040

HQ613403.2007 GGGTGAACAACAGAAAAACAGTCAAAAGGGCCAGCATACAGAGGGCAGACAGACACAATC 1992

KC242785.2007 GGGTGAACAACAGAAAAACAGTCAAAAGGGCCAGCATACAGAGGGCAGACAGACACAATC 2040

KC242790.2007 GGGTGAACAACAGAAAAACAGTCAAAAGGGCCAGCATACAGAGGGCAGACAGACACAATC 2040

KU143789.2014 GGGTGGACAACAGAAAAACAGTCAAAAGGGCCAGCATACAGAGGGCAGACAGACACAATC 2040

KR817168.2014 GGGTGGACAACAGAAAAACAGTCAAAAGGGCCAGCATACAGAGGGCAGACAGACACAATC 2014

KY426696.2015 GGGTGGACAACAGAAAAACAGTCAAAAGGGCCAGCATACAGAGGGCAGACAGACACAATC 2040

KR105271.2014 GGGTGGACAACAGAAAAACAGTCAAAAGGGCCAGCATACAGAGGGCAGACAGACACAATC 2007

KY007522.2016 GGGTGGACAACAGAAAAACAGTCAAAAGGGCCAGCATACAGAGGGCAGACAGACACAATC 2020

KM034555.2014 GGGTGGACAACAGAAAAACAGTCAAAAGGGCCAGCATACAGAGGGCAGACAGACACAATC 2032

MH470381.2015 GGGTGGACAACAGAAAAACAGTCAAAAGGGCCAGCATACAGAGGGCAGACAGACACAATC 2004

MH470382.2015 GGGTGGACAACAGAAAAACAGTCAAAAGGGCCAGCATACAGAGGGCAGACAGACACAATC 2004

MF102255.2014 GGGTGGACAACAGAAAAACAGTCAAAAGGGCCAGCATACAGAGGGCAGACAGACACAATC 2017

KJ660348.2014 GGGTGGACAACAGAAAAACAGTCAAAAGGGCCAGCATACAGAGGGCAGACAGACACAATC 2040

KU143818.2014 GGGTGGACAACAGAAAAACAGTCAAAAGGGCCAGCATACAGAGGGCAGACAGACACAATC 2040

KT725333.2014 GGGTGGACAACAGAAAAACAGTCAAAAGGGCCAGCATACAGAGGGCAGACAGACACAATC 2005

KR819004.2014 GGGTGGACAACAGAAAAACAGTCAAAAAGGCCAGCATACAGAGGGCAGACAGACACAATC 1987

KP271020.2014 GGGTGGACAACAGAAAAACAGTCAAAAAGGCCAGCATACAGAGGGCAGACAGACACAATC 1987

KM519951.2014 GGGTGGACAACAGAAAAACAGTCAAAAAGGCCAGCATACAGAGGGCAGACAGACACAATC 2037

MH733488.2018 GGGTGGACAACAGAAAAACAGTCAAAAGGGCCAGCATACAGAGGGCAGACAGACACAATC 2031

MH733491.2018 GGGTGGACAACAGAAAAACAGTCAAAAGGGCCAGCATACAGAGGGCAGACAGACACAATC 2026

MH733478.2018 GGGTGGACAACAGAAAAACAGTCAAAAGGGCCAGCATACAGAGGGCAGACAGACACAATC 2029

MK007330.2018 GGGTGGACAACAGAAAAACAGTCAAAAGGGCCAGCATACAGAGGGCAGACAGGCACAATC 2028

MK007344.2018 GGGTGGACAACAGAAAAACAGTCAAAAGGGCCAGCATACAGAGGGCAGACAGGCACAATC 2022

***** ********* *********** ********** ************* *******

AY142960.1976 CAGGCCAATTCAAAATGTCCCAGGCCCTCACAGAACAATCCACCACGCCAGTGCGCCACT 2100

KC242791.1977 CAGGCCAATTCAAAATGTCCCAGGCCCTCACAGAACAATCCACCACGCCAGTGCGCCACT 2100

AF499101.1976 CAGGCCAATTCAAAATGTCCCAGGCCCTCACAGAACAATCCACCACGCCAGTGCGCCACT 2100

KF113528.2003 CAGGCCAACTCAAAATGTCCCAGGCCCTCGCAGAACAATCCACCACGCCAGTGCTCCACT 2096

KC242800.2002 CAGGCCAACTCAAAATGTCCCAGGCCCTCGCAGAACAATCCACCACGCCAGTGCTCCACT 2100

KY471090.2001 CAGGCCAACTCAAAATGTCCCAGGCCCTCGCAGAACAATCCACCACGCCAGTGCTCCACT 2077

KY471092.2001 CAGGCCAACTCAAAATGTCCCAGGCCCTCGCAGAACAATCCACCACGCCAGTGCTCCACT 2077

MH481611.2017 CAGGCCAACTCAAAATGTCCCAGGCCCTCACAGAACAATCCACCACGCCAGTGCTCCATT 2066

MH613311.2017 CAGGCCAACTCAAAATGTCCCAGGCCCTCACAGAACAATCCACCACGCCAGTGCTCCATT 2045

KC242792.1994 CAGGCCAACTCAAAATATCCCAGGCCCTCACAGAACAATCCACCACGCAAGTGCTCCACT 2100

KC242793.1996 CAGGCCAACTCAAAATATCCCAGGCCCTCACAGAACAATCCACCACGCAAGTGCTCCACT 2100

KC242794.1996 CAGGCCAACTCAAAATGTCCCAGGCCCTCACAGAACAATCCACCACGCAAGTGCTCCACT 2100

MH121164.1995 CAGGCCAACTCAAAATGTCCCAGGCCCTCACAGAACAATCCACCACGCCAGTGCTCCACT 2100

AY354458.1995 CAGGCCAACTCAAAATGTCCCAGGCCCTCACAGAACAATCCACCACGCCAGTGCTCCACT 2100

KT762962.1995 CAGGCCAACTCAAAATGTCCCAGGCCCTCACAGAACAATCCACCACGCCAGTGCTCCACT 2100

HQ613402.2008 CAGGCCAACTCAAAATGTCCCAGGCCCTCGCAGAACAATCCACCATGCCAGCGCTCCACT 2019

KC242789.2007 CAGGCCAACTCAAAATGTCCCAGGCCCTCGCAGAACAATCCACCATGCCAGCGCTCCACT 2100

HQ613403.2007 CAGGCCAACTCAAAATGTCCCAGGCCCTCGCAGAACAATCCACCATGCCAGCGCTCCACT 2052

KC242785.2007 CAGGCCAACTCAAAATGTCCCAGGCCCTCGCAGAACAATCCACCATGCCAGCGCTCCACT 2100

KC242790.2007 CAGGCCAACTCAAAATGTCCCAGGCCCTCGCAGAACAATCCACCATGCCAGCGCTCCACT 2100

KU143789.2014 CACGCCAACTCAAAACGTCACAGGCCCTCGCAGAACAATCCACCATGCCAGTGCTCCACT 2100

KR817168.2014 CACGCCAACTCAAAACGTCACAGGCCCTCGCAGAACAATCCACCATGCCAGTGCTCCACT 2074

KY426696.2015 CACGCCAACTCAAAACGTCACAGGCCCTCGCAGAACAATCCACCATGCCAGTGCTCCACT 2100

KR105271.2014 CACGCCAACTCAAAACGTCACAGGCCCTCGCAGAACAATCCACCATGCCAGTGCTCCACT 2067

KY007522.2016 CACGCCAACTCAAAACGTCACAGGCCCTCGCAGAACAATCCACCATGCCAGTGCTCCACT 2080

KM034555.2014 CACGCCAACTCAAAACGTCACAGGCCCTCGCAGAACAATCCACCATGCCAGTGCTCCACT 2092

MH470381.2015 CACGCCAACTCAAAACGTCACAGGCCCTCGCAGAACAATCCACCATGCCAGTGCTCCACT 2064

MH470382.2015 CACGCCAACTCAAAACGTCACAGGCCCTCGCAGAACAATCCACCATGCCAGTGCTCCACT 2064

MF102255.2014 CACGCCAACTCAAAACGTCACAGGCCCTCGCAGAACAATCCACCATGCCAGTGCTCCACT 2077

KJ660348.2014 CACGCCAACTCAAAACGTCACAGGCCCTCGCAGAACAATCCACCATGCCAGTGCTCCACT 2100

KU143818.2014 CACGCCAACTCAAAACGTCACAGGCCCTCGCAGAACAATCCACCATGCCAGTGCTCCACT 2100

KT725333.2014 CACGCCAACTCAAAACGTCACAGGCCCTCGCAGAACAATCCACCATGCCAGTGCTCCACT 2065

KR819004.2014 CAGGCCAACTCAAAATGTCCCAGGCCCTCACAGAGCAATCCACCACACCAGTGCTCCACT 2047

KP271020.2014 CAGGCCAACTCAAAATGTCCCAGGCCCTCACAGAGCAATCCACCACACCAGTGCTCCACT 2047

KM519951.2014 CAGGCCAACTCAAAATGTCCCAGGCCCTCACAGAGCAATCCACCACACCAGTGCTCCACT 2097

MH733488.2018 CAGGCCAACTCAAAATGTCCCAGGCCCTCACAGAGCAATCCACCACACCAGTGCTCCACT 2091

MH733491.2018 CAGGCCAACTCAAAATGTCCCAGGCCCTCACAGAGCAATCCACCACACCAGTGCTCCACT 2086

MH733478.2018 CAGGCCAACTCAAAATGTCCCAGGCCCTCACAGAGCAATCCACCACACCAGTGCTCCACT 2089

MK007330.2018 CAGGCCAACTCAAAATGTCCCAGGCCCTCACAGAACAATCCACCACGACAGTGCTCCACT 2088

MK007344.2018 CAGGCCAACTCAAAATGTCCCAGGCCCTCACAGAACAATCCACCACGACAGTGCTCCACT 2082

** ***** ****** ** ********* **** ********** ** ** *** *

AY142960.1976 CACGGACAATGACAGAAGAAATGAACCCTCCGGCTCAACCAGCCCTCGCATGCTGACACC 2160

KC242791.1977 CACGGACAATGACAGAAGAAATGAACCCTCCGGCTCAACCAGCCCTCGCATGCTGACACC 2160

AF499101.1976 CACGGACAATGACAGAAGAAATGAACCCTCCGGCTCAACCAGCCCTCGCATGCTGACACC 2160

KF113528.2003 CACGGACAACGACAGAGGAAATGAACCCTCCGGCTCAACCAGCCCTCGCATGCTGACACC 2156

KC242800.2002 CACGGACAACGACAGAGGAAATGAACCCTCCGGCTCAACCAGCCCTCGCATGCTGACACC 2160

KY471090.2001 CACGGACAACGACAGAGGAAATGAACCCTCCGGCTCAACCAGCCCTCGCATGCTGACACC 2137

KY471092.2001 CACGGACAACGACAGAGGAAATGAACCCTCCGGCTCAACCAGCCCTCGCATGCTGACACC 2137

MH481611.2017 CACGGACAATGACAGAAGAAATGAACCCTCCGGCTCAACCAGCCCTCGCATGCTGACACC 2126

MH613311.2017 CACGGACAATGACAGAAGAAATGAACCCTCCGGCTCAACCAGCCCTCGCATGCTGACACC 2105

KC242792.1994 CACGGACAATGACAGAAGAAATGAACCCTCCGGCTCAACCAGCCCTCGCATGCTGACACC 2160

KC242793.1996 CACGGACAATGACAGAAGAAATGAACCCTCCGGCTCAACCAGCCCTCGCATGCTGACACC 2160

KC242794.1996 CACGGACAATGACAGAAGAAATGAACCCTCCGGCTCAACCAGCCCTCGCATGCTGACACC 2160

MH121164.1995 CACGGATAATGACAGAAGAAATGAACCCTCCGGCTCAACCAGCCCTCGCATGCTGACACC 2160

AY354458.1995 CACGGATAATGACAGAAGAAATGAACCCTCCGGCTCAACCAGCCCTCGCATGCTGACACC 2160

KT762962.1995 CACGGATAATGACAGAAGAAATGAACCCTCCGGCTCAACCAGCCCTCGCATGCTGACACC 2160

HQ613402.2008 CACGGACAATGACAGAAGAAATGAACCCTCCGGCTCAACCAGCCCTCGCATGCTGACACC 2079

KC242789.2007 CACGGACAATGACAGAAGAAATGAACCCTCCGGCTCAACCAGCCCTCGCATGCTGACACC 2160

HQ613403.2007 CACGGACAATGACAGAAGAAATGAACCCTCCGGCTCAACCAGCCCTCGCATGCTGACACC 2112

KC242785.2007 CACGGACAATGACAGAAGAAATGAACCCTCCGGCTCAACCAGCCCTCGCATGCTGACACC 2160

KC242790.2007 CACGGACAATGACAGAAGAAATGAACCCTCCGGCTCAACCAGCCCTCGCATGCTGACACC 2160

KU143789.2014 CACGGACAATGACAGAAGAAACGAACCCTCCGGCTCAACCAGCCCTCGCATGCTGACCCC 2160

KR817168.2014 CACGGACAATGACAGAAGAAACGAACCCTCCGGCTCAACCAGCCCTCGCATGCTGACCCC 2134

KY426696.2015 CACGGACAATGACAGAAGAAACGAACCCTCCGGCTCAACCAGCCCTCGCATGCTGACCCC 2160

KR105271.2014 CACGGACAATGACAGAAGAAACGAACCCTCCGGCTCAACCAGCCCTCGCATGCTGACCCC 2127

KY007522.2016 CACGGACAATGACAGAAGAAACGAACCCTCCGGCTCAACCAGCCCTCGCATGCTGACCCC 2140

KM034555.2014 CACGGACAATGACAGAAGAAACGAACCCTCCGGCTCAACCAGCCCTCGCATGCTGACCCC 2152

MH470381.2015 CACGGACAATGACAGAAGAAACGAACCCTCCGGCTCAACCAGCCCTCGCATGCTGACCCC 2124

MH470382.2015 CACGGACAATGACAGAAGAAACGAACCCTCCGGCTCAACCAGCCCTCGCATGCTGACCCC 2124

MF102255.2014 CACGGACAATGACAGAAGAAACGAACCCTCCGGCTCAACCAGCCCTCGCATGCTGACCCC 2137

KJ660348.2014 CACGGACAATGACAGAAGAAACGGACCCTCCGGCTCAACCAGCCCTCGCATGCTGACCCC 2160

KU143818.2014 CACGGACAATGACAGAAGAAACGAACCCTCCGGCTCAACCAGCCCTCGCATGCTGACCCC 2160

KT725333.2014 CACGGACAATGACAGAAGAAACGAACCCTCCGGCTCAACCAGCCCTCGCATGCTGACCCC 2125

KR819004.2014 CACGGACAATGACAGAAGAAATGAACCCTCCAGCTCAACCAGCCCTCGCATGCTGACACC 2107

KP271020.2014 CACGGACAATGACAGAAGAAATGAACCCTCCAGCTCAACCAGCCCTCGCATGCTGACACC 2107

KM519951.2014 CACGGACAATGACAGAAGAAATGAACCCTCCAGCTCAACCAGCCCTCGCATGCTGACACC 2157

MH733488.2018 CACGGACAATGACAGAAGAAATGAACCCTCCAGCTCAACCAGCCCTCGCATGCTGACACC 2151

MH733491.2018 CACGGACAATGACAGAAGAAATGAACCCTCCAGCTCAACCAGCCCTCGCATGCTGACACC 2146

MH733478.2018 CACGGACAATGACAGAAGAAATGAACCCTCCAGCTCAACCAGCCCTCGCATGCTGACACC 2149

MK007330.2018 CACGGACAATAACAGAAGAAATGAACCCTCCGGCTCAACCAGCCCTCGCATGCTGACACC 2148

MK007344.2018 CACGGACAATAACAGAAGAAATGAACCCTCCGGCTCAACCAGCCCTCGCATGCTGACACC 2142

****** ** ***** **** * ******* ************************* **

AY142960.1976 AATTAACGAAGAGGCAGACCCACTGGACGATGCCGACGACGAGACGTCTAGCCTTCCGCC 2220

KC242791.1977 AATTAACGAAGAGGCAGACCCACTGGACGATGCCGACGACGAGACGTCTAGCCTTCCGCC 2220

AF499101.1976 AATTAACGAAGAGGCAGACCCACTGGACGATGCCGACGACGAGACGTCTAGCCTTCCGCC 2220

KF113528.2003 AATTAACGAAGAGGCAGACCCACTGGACGATGCCGACGACGAGACGTCTAGTCTTCCGCC 2216

KC242800.2002 AATTAACGAAGAGGCAGACCCACTGGACGATGCCGACGACGAGACGTCTAGTCTTCCGCC 2220

KY471090.2001 AATTAACGAAGAGGCAGACCCACTGGACGATGCCGACGACGAGACGTCTAGTCTTCCGCC 2197

KY471092.2001 AATTAACGAAGAGGCAGACCCACTGGACGATGCCGACGACGAGACGTCTAGTCTTCCGCC 2197

MH481611.2017 AATTAACGAAGAGGCGGACCCACTGGACGATGCCGACGACGAGACGTCTAGCCTTCCGCC 2186

MH613311.2017 AATTAACGAAGAGGCGGACCCACTGGACGATGCCGACGACGAGACGTCTAGCCTTCCGCC 2165

KC242792.1994 AATCAACGAAGAGGCAGACCCACTGGACGATGCCGACGACGAAACGTCTAGCCTTCCGCC 2220

KC242793.1996 AATCAACGAAGAGGCAGACCCACTGGACGATGCCGACGACGAGACGTCTAGCCTTCCGCC 2220

KC242794.1996 AATCAACGAAGAGGCAGACCCACTGGACGATGCCGACGACGAGACGTCTAGCCTTCCGCC 2220

MH121164.1995 AATCAACGAAGAGGCAGACCCACTGGACGATGCCGACGACGAGACGTCTAGCCTTCCGCC 2220

AY354458.1995 AATCAACGAAGAGGCAGACCCACTGGACGATGCCGACGACGAGACGTCTAGCCTTCCGCC 2220

KT762962.1995 AATCAACGAAGAGGCAGACCCACTGGACGATGCCGACGACGAGACGTCTAGCCTTCCGCC 2220

HQ613402.2008 AATTAACGAGGAGGCAGACCCACTGGACGATGCCGACGACGAGACGTCCAGCCTTCCGCC 2139

KC242789.2007 AATTAACGAGGAGGCAGACCCACTGGACGATGCCGACGACGAGACGTCCAGCCTTCCGCC 2220

HQ613403.2007 AATTAACGAGGAGGCAGACCCACTGGACGATGCCGACGACGAGACGTCCAGCCTTCCGCC 2172

KC242785.2007 AATTAACGAGGAGGCAGACCCACTGGACGATGCCGACGACGAGACGTCCAGCCTTCCGCC 2220

KC242790.2007 AATTAACGAGGAGGCAGACCCACTGGACGATGCCGACGACGAGACGTCCAGCCTTCCGCC 2220

KU143789.2014 AATCAACGAAGAGGCAGACCCACTGGACGATGCCGACGACGAGACGTCTAGCCTTCCGCC 2220

KR817168.2014 AATCAACGAAGAGGCAGACCCACTGGACGATGCCGACGACGAGACGTCTAGCCTTCCGCC 2194

KY426696.2015 AATCAACGAAGAGGCAGACCCACTGGACGATGCCGACGACGAGACGTCTAGCCTTCCGCC 2220

KR105271.2014 AATCAACGAAGAGGCAGACCCACTGGACGATGCCGACGACGAGACGTCTAGCCTTCCGCC 2187

KY007522.2016 AATCAACGAAGAGGCAGACCCACTGGACGATGCCGACGACGAGACGTCTAGCCTTCCGCC 2200

KM034555.2014 AATCAACGAAGAGGCAGACCCACTGGACGATGCCGACGACGAGACGTCTAGCCTTCCGCC 2212

MH470381.2015 AATCAACGAAGAGGCAGACCCACTGGACGATGCCGACGACGAGACGTCTAGCCTTCCGCC 2184

MH470382.2015 AATCAACGAAGAGGCAGACCCACTGGACGATGCCGACGACGAGACGTCTAGCCTTCCGCC 2184

MF102255.2014 AATCAACGAAGAGGCAGACCCACTGGACGATGCCGACGACGAGACGTCTAGCCTTCCGCC 2197

KJ660348.2014 AATCAACGAAGAGGCAGACCCACTAGACGATGCCGACGACGAGACGTCTAGCCTTCCGCC 2220

KU143818.2014 AATCAACGAAGAGGCAGACCCACTGGACGATGCCGACGACGAGACGTCTAGCCTTCCGCC 2220

KT725333.2014 AATCAACGAAGAGGCAGACCCACTGGACGATGCCGACGACGAGACGTCTAGCCTTCCGCC 2185

KR819004.2014 AATCAACGAAGAGGCAGACCCACTGGACGATGCCGACGACGAGACGTCTAGCCTTCCGCC 2167

KP271020.2014 AATCAACGAAGAGGCAGACCCACTGGACGATGCCGACGACGAGACGTCTAGCCTTCCGCC 2167

KM519951.2014 AATCAACGAAGAGGCAGACCCACTGGACGATGCCGACGACGAGACGTCTAGCCTTCCGCC 2217

MH733488.2018 AATCAACGAAGAGGCAGACCCACTGGACGATGCCGACGACGAGACGTCCAGCCTTCCGCC 2211

MH733491.2018 AATCAACGAAGAGGCAGACCCACTGGACGATGCCGACGACGAGACGTCCAGCCTTCCGCC 2206

MH733478.2018 AATCAACGAAGAGGCAGACCCACTGGACGATGCCGACGACGAGACGTCCAGCCTTCCGCC 2209

MK007330.2018 AATTAACGAAGAGGCGGACCCACTGGACGATGCCGACGACGAGACGTCTAGCCTTCCGCC 2208

MK007344.2018 AATTAACGAAGAGGCGGACCCACTGGACGATGCCGACGACGAGACGTCTAGCCTTCCGCC 2202

*** ***** ***** ******** ***************** ***** ** ********

AY142960.1976 CTTGGAGTCAGATGATGAAGAGCAGGACAGGGACGGAACTTCCAACCGCACACCCACTGT 2280

KC242791.1977 CTTGGAGTCAGATGATGAAGAGCAGGACAGGGACGGAACTTCCAACCGCACACCCACTGT 2280

AF499101.1976 CTTGGAGTCAGATGATGAAGAGCAGGACAGGGACGGAACTTCCAACCGCACACCCACTGT 2280

KF113528.2003 CTTGGAGTCAGACGATGAAGAACAGGACAGGGACGAAACCTCCAACCGCACACCCACTGT 2276

KC242800.2002 CTTGGAGTCAGACGATGAAGAACAGGACAGGGACGAAACTTCCAACCGCACACCCACTGT 2280

KY471090.2001 CTTGGAGTCAGACGATGAAGAACAGGACAGGGACGAAACTTCCAACCGCACACCCACTGT 2257

KY471092.2001 CTTGGAGTCAGACGATGAAGAACAGGACAGGGACGAAACTTCCAACCGCACACCCACTGT 2257

MH481611.2017 CTTGGAGTCAGATGATGAAGAACAGGACAGGGACGGAATTTCCAACCGCACACCCACTGT 2246

MH613311.2017 CTTGGAGTCAGATGATGAAGAACAGGACAGGGACGGAATTTCCAACCGCACACCCACTGT 2225

KC242792.1994 CTTGGAGTCAGATGATGAAGAACAGGACAGGGACGGAACCTCCAATCGCACACCCACTGT 2280

KC242793.1996 CTTGGAGTCAGATGAAGAAGAACAGGACAGGGACGGAACCTCCAATCGCACACCCACTGT 2280

KC242794.1996 CTTGGAGTCAGATGATGAAGAACAGGACAGGGGCGGAACCTCCAATCGCACACCCACTGT 2280

MH121164.1995 CCTGGAGTCAGACGATGAAGAACAGGACAGGGACGGAACTTCCAACCGCACACCCACTGT 2280

AY354458.1995 CCTGGAGTCAGACGATGAAGAACAGGACAGGGACGGAACTTCCAACCGCACACCCACTGT 2280

KT762962.1995 CCTGGAGTCAGACGATGAAGAACAGGACAGGGACGGAACTTCCAACCGCACACCCACTGT 2280

HQ613402.2008 CTTGGAGTCAGATGATGAAGAACAGGACAGGGACGGAACTTCCAACCGCACACCCACTGT 2199

KC242789.2007 CTTGGAGTCAGATGATGAAGAACAGGACAGGGACGGAACTTCCAACCGCACACCCACTGT 2280

HQ613403.2007 CTTGGAGTCAGATGATGAAGAACAGGACAGGGACGGAACTTCCAACCGCACACCCACTGT 2232

KC242785.2007 CTTGGAGTCAGATGATGAAGAACAGGACAGGGACGGAACTTCCAACCGCACACCCACTGT 2280

KC242790.2007 CTTGGAGTCAGATGATGAAGAACAGGACAGGGACGGAACTTCCAACCGCACACCCACTGT 2280

KU143789.2014 CTTAGAGTCAGATGATGAAGAACAGGACAGGGACGGAACTTCTAACCGCACACCCACTGT 2280

KR817168.2014 CTTAGAGTCAGATGATGAAGAACAGGACAGGGACGGAACTTCTAACCGCACACCCACTGT 2254

KY426696.2015 CTTAGAGTCAGATGATGAAGAACAGGACAGGGACGGAACTTCTAACCGCACACCCACTGT 2280

KR105271.2014 CTTAGAGTCAGATGATGAAGAACAGGACAGGGACGGAACTTCTAACCGCACACCCACTGT 2247

KY007522.2016 CTTAGAGTCAGATGATGAAGAACAGGACAGGGACGGAACTTCTAACCGCACACCCACTGT 2260

KM034555.2014 CTTAGAGTCAGATGATGAAGAACAGGACAGGGACGGAACTTCTAACCGCACACCCACTGT 2272

MH470381.2015 CTTAGAGTCAGATGATGAAGAACAGGACAGGGACGGAACTTCTAACCGCACACCCACTGT 2244

MH470382.2015 CTTAGAGTCAGATGATGAAGAACAGGACAGGGACGGAACTTCTAACCGCACACCCACTGT 2244

MF102255.2014 CTTAGAGTCAGATGATGAAGAACAGGACAGGGACGGAACTTCTAACCGCACACCCACTGT 2257

KJ660348.2014 CTTAGAGTCAGATGATGAAGAACAGGACAGGGACGGAACTTCTAACCGCACACCCACTGT 2280

KU143818.2014 CTTAGAGTCAGATGATGAAGAACAGGACAGGGACGGAACTTCTAACCGCACACCCACTGT 2280

KT725333.2014 CTTAGAGTCAGATGATGAAGAACAGGACAGGGACGGAACTTCTAACCGCACACCCACTGT 2245

KR819004.2014 CTTGGAGTCAGATGATGAAGAACAGGACAGGGACGGAACCTCCAACCGCACACCCACTGT 2227

KP271020.2014 CTTGGAGTCAGATGATGAAGAACAGGACAGGGACGGAACCTCCAACCGCACACCCACTGT 2227

KM519951.2014 CTTGGAGTCAGATGATGAAGAACAGGACAGGGACGGAACCTCCAACCGCACACCCACTGT 2277

MH733488.2018 CTTGGAGTCAGACGATGAAGAACAGGACAGGGACGGAACCCCCAACCGCACACCCACTGT 2271

MH733491.2018 CTTGGAGTCAGACGATGAAGAACAGGACAGGGACGGAACCCCCAACCGCACACCCACTGT 2266

MH733478.2018 CTTGGAGTCAGACGATGAAGAACAGGACAGGGACGGAACCCCCAACCGCACACCCACTGT 2269

MK007330.2018 CTTGGAGTCAGATGATGAAGAACAGGACAGGGACGGAACTTCCAACCGCACACCCACTGT 2268

MK007344.2018 CTTGGAGTCAGATGATGAAGAACAGGACAGGGACGGAACTTCCAACCGCACACCCACTGT 2262

* * ******** ** ***** ********** ** ** * ** **************

AY142960.1976 CGCCCCACCGGCTCCCGTATACAGAGATCACTCTGAAAAGAAAGAACTCCCGCAAGACGA 2340

KC242791.1977 CGCCCCACCGGCTCCCGTATACAGAGATCACTCTGAAAAGAAAGAACTCCCGCAAGACGA 2340

AF499101.1976 CGCCCCACCGGCTCCCGTATACAGAGATCACTCTGAAAAGAAAGAACTCCCGCAAGACGA 2340

KF113528.2003 CGCCCCACCGGCTCCCGTATACAGAGATCACTCTGAAAAGAAAGAACTCCCGCAAGATGA 2336

KC242800.2002 CGCCCCACCGGCTCCCGTATACAGAGATCACTCTGAAAAGAAAGAACTCCCGCAAGATGA 2340

KY471090.2001 CGCCCCACCGGCTCCCGTATACAGAGATCACTCTGAAAAGAAAGAACTCCCGCAAGATGA 2317

KY471092.2001 CGCCCCACCGGCTCCCGTATACAGAGATCACTCTGAAAAGAAAGAACTCCCGCAAGATGA 2317

MH481611.2017 CGCCCCACCGGCTCCCGTATACAGAGATCACCCTGAAAAGAAAGAACTCCCGCAAGATGA 2306

MH613311.2017 CGCCCCACCGGCTCCCGTATACAGAGATCACCCTGAAAAGAAAGAACTCCCGCAAGATGA 2285

KC242792.1994 CGCCCCACCGGCTCCCGTATACAGAGATCACTCTGAAAAGAAAGAACTCCCGCAAGATGA 2340

KC242793.1996 CGCCCCACCGGCTCCCGTATACAGAGATCACTCTGAAAAGAAAGAACTCCCGCAAGATGA 2340

KC242794.1996 CGCCCCACCGGCTCCCGTATACAGAGATCACTCTGAAAAGAAAGAACTCCCGCAAGATGA 2340

MH121164.1995 CGCCCCACCGGCTCCCGTATACAGAGATCACTCTGAAAAGAGAGAACTCCCGCAAGATGA 2340

AY354458.1995 CGCCCCACCGGCTCCCGTATACAGAGATCACTCTGAAAAGAGAGAACTCCCGCAAGATGA 2340

KT762962.1995 CGCCCCACCGGCTCCCGTATACAGAGATCACTCTGAAAAGAGAGAACTCCCGCAAGATGA 2340

HQ613402.2008 CGCCCCACCGGCTCCCGTATACAGAGATCACTCTGAAAAGAAAGAACTCCCGCAAGATGA 2259

KC242789.2007 CGCCCCACCGGCTCCCGTATACAGAGATCACTCTGAAAAGAAAGAACTCCCGCAAGATGA 2340

HQ613403.2007 CGCCCCACCGGCTCCCGTATACAGAGATCACTCTGAAAAGAAAGAACTCCCGCAAGATGA 2292

KC242785.2007 CGCCCCACCGGCTCCCGTATACAGAGATCACTCTGAAAAGAAAGAACTCCCGCAAGATGA 2340

KC242790.2007 CGCCCCACCGGCTCCCGTATACAGAGATCACTCTGAAAAGAAAGAACTCCCGCAAGATGA 2340

KU143789.2014 CGCCCCACCGGCTCCCGTATACAGAGATCACTCCGAAAAGAAAGAACTCCCGCAAGATGA 2340

KR817168.2014 CGCCCCACCGGCTCCCGTATACAGAGATCACTCCGAAAAGAAAGAACTCCCGCAAGATGA 2314

KY426696.2015 CGCCCCACCGGCTCCCGTATACAGAGATCACTCCGAAAAGAAAGAACTCCCGCAAGATGA 2340

KR105271.2014 CGCCCCACCGGCTCCCGTATACAGAGATCACTCCGAAAAGAAAGAACTCCCGCAAGATGA 2307

KY007522.2016 CGCCCCACCGGCTCCCGTATACAGAGATCACTCCGAAAAGAAAGAACTCCCGCAAGATGA 2320

KM034555.2014 CGCCCCACCGGCTCCCGTATACAGAGATCACTCCGAAAAGAAAGAACTCCCGCAAGATGA 2332

MH470381.2015 CGCCCCACCGGCTCCCGTATACAGAGATCACTCCGAAAAGAAAGAACTCCCGCAAGATGA 2304

MH470382.2015 CGCCCCACCGGCTCCCGTATACAGAGATCACTCCGAAAAGAAAGAACTCCCGCAAGATGA 2304

MF102255.2014 CGCCCCACCGGCTCCCGTATACAGAGATCACTCCGAAAAGAAAGAACTCCCGCAAGATGA 2317

KJ660348.2014 CGCCCCACCGGCTCCCGTATACAGAGATCACTCCGAAAAGAAAGAACTCCCGCAAGATGA 2340

KU143818.2014 CGCCCCACCGGCTCCCGTATACAGAGATCACTCCGAAAAGAAAGAACTCCCGCAAGATGA 2340

KT725333.2014 CGCCCCACCGGCTCCCGTATACAGAGATCACTCCGAAAAGAAAGAACTCCCGCAAGATGA 2305

KR819004.2014 CGCCCCACCGGCTCCCGTATACAGAGATCACTCTGAAAAGAAAGAACTCCCGCAAGATGA 2287

KP271020.2014 CGCCCCACCGGCTCCCGTATACAGAGATCACTCTGAAAAGAAAGAACTCCCGCAAGATGA 2287

KM519951.2014 CGCCCCACCGGCTCCCGTATACAGAGATCACTCTGAAAAGAAAGAACTCCCGCAAGATGA 2337

MH733488.2018 CGCCCCACCGGCTCCCGTATACAGAGATCACTCTGAAAAGAAAGAACTCCCGCAAGATGA 2331

MH733491.2018 CGCCCCACCGGCTCCCGTATACAGAGATCACTCTGAAAAGAAAGAACTCCCGCAAGATGA 2326

MH733478.2018 CGCCCCACCGGCTCCCGTATACAGAGATCACTCTGAAAAGAAAGAACTCCCGCAAGATGA 2329

MK007330.2018 CGCCCCACCGGCCCCCGTATACAGAGATCACTCTGAAAAGAAAGAACTCCCGCAGGATGA 2328

MK007344.2018 CGCCCCACCGGCCCCCGTATACAGAGATCACTCTGAAAAGAAAGAACTCCCGCAGGATGA 2322

************ ****************** * ******* ************ ** **

AY142960.1976 GCAACAAGATCAGGACCACACTCAAGAGGCCAGGAACCAGGACAGTGACAACACCCAGTC 2400

KC242791.1977 GCAACAAGATCAGGACCACACTCAAGAGGCCAGGAACCAGGACAGTGACAACACCCAGTC 2400

AF499101.1976 GCAACAAGATCAGGACCACACTCAAGAGGCCAGGAACCAGGACAGTGACAACACCCAGTC 2400

KF113528.2003 GCAGCAAGATCAGGACCACACTCAAGAGGCCAGGAACCAGGACAGTGACAACACCCAGCC 2396

KC242800.2002 GCAGCAAGATCAGGACCACACTCAAGAGGCCAGGAACCAGGACAGTGACAACACCCAGCC 2400

KY471090.2001 GCAGCAAGATCAGGACCACACTCAAGAGGCCAGGAACCAGGACAGTGACAACACCCAGCC 2377

KY471092.2001 GCAGCAAGATCAGGACCACACTCAAGAGGCCAGGAACCAGGACAGTGACAACACCCAGCC 2377

MH481611.2017 GCAACAAGATCAGGACCACACTCAAGAGGCCAGGAACCAGGACAGTGACAACACCCAGCC 2366

MH613311.2017 GCAACAAGATCAGGACCACACTCAAGAGGCCAGGAACCAGGACAGTGACAACACCCAGCC 2345

KC242792.1994 GCGACAAGATCAGGACCACACTCAAGAGGCCAGGAACCAGGACAGTGACAACACCCAGCC 2400

KC242793.1996 GCGACAAGATCAGGACCACACTCAAGAGGCCAGGAACCAGGACAGTGACAACACCCAGCC 2400

KC242794.1996 GCGACAAGATCAGGACCACACTCAAGAGGCCAGGAACCAGGACAGTGACAACACCCAGCC 2400

MH121164.1995 GCAACAAGATCAGGACCACACTCAAGAGGCCAGGAACCAGGACAGTGACAACACCCAGCC 2400

AY354458.1995 GCAACAAGATCAGGACCACACTCAAGAGGCCAGGAACCAGGACAGTGACAACACCCAGCC 2400

KT762962.1995 GCAACAAGATCAGGACCACACTCAAGAGGCCAGGAACCAGGACAGTGACAACACCCAGCC 2400

HQ613402.2008 GCAACAAGACCAGGACCACACTCAAGAGGCCAAGAACCAGGACAGTGACAACACCCAGCC 2319

KC242789.2007 GCAACAAGACCAGGACCACACTCAAGAGGCCAAGAACCAGGACAGTGACAACACCCAGCC 2400

HQ613403.2007 GCAACAAGACCAGGACCACACTCAAGAGGCCAAGAACCAGGACAGTGACAACACCCAGCC 2352

KC242785.2007 GCAACAAGACCAGGACCACACTCAAGAGGCCAAGAACCAGGACAGTGACAACACCCAGCC 2400

KC242790.2007 GCAACAAGACCAGGACCACACTCAAGAGGCCAAGAACCAGGACAGTGACAACACCCAGCC 2400

KU143789.2014 ACAACAAGATCAGGACCACATTCAAGAGGCCAGGAACCAAGACAGTGACAACACCCAGCC 2400

KR817168.2014 ACAACAAGATCAGGACCACATTCAAGAGGCCAGGAACCAAGACAGTGACAACACCCAGCC 2374

KY426696.2015 ACAACAAGATCAGGACCACATTCAAGAGGCCAGGAACCAAGACAGTGACAACACCCAGCC 2400

KR105271.2014 ACAACAAGATCAGGACCACATTCAAGAGGCCAGGAACCAAGACAGTGACAACACCCAGCC 2367

KY007522.2016 ACAACAAGATCAGGACCACATTCAAGAGGCCAGGAACCAAGACAGTGACAACACCCAGCC 2380

KM034555.2014 ACAACAAGATCAGGACCACATTCAAGAGGCCAGGAACCAAGACAGTGACAACACCCAGCC 2392

MH470381.2015 ACAACAAGATCAGGACCACATTCAAGAGGCCAAGAACCAAGACAGTGACAACACCCAGCC 2364

MH470382.2015 ACAACAAGATCAGGACCACATTCAAGAGGCCAGGAACCAAGACAGTGACAACACCCAGCC 2364

MF102255.2014 ACAACAAGATCAGGACCACATTCAAGAGGCCAGGAACCAAGACAGTGACAACACCCAGCC 2377

KJ660348.2014 ACAACAAGATCAGGACCACATTCAAGAGGCCAGGAACCAAGACAGTGACAACACCCAGCC 2400

KU143818.2014 ACAACAAGATCAGGACCACATTCAAGAGGCCAGGAACCAAGACAGTGACAACACCCAGCC 2400

KT725333.2014 ACAACAAGATCAGGACCACATTCAAGAGGCCAGGAACCAAGACAGTGACAACACCCAGCC 2365

KR819004.2014 GCAACAAGATCAGGACCACACTCAAGAGGCCAGGAACCAGGACAGTGACAACACCCAGCC 2347

KP271020.2014 GCAACAAGATCAGGACCACACTCAAGAGGCCAGGAACCAGGACAGTGACAACACCCAGCC 2347

KM519951.2014 GCAACAAGATCAGGACCACACTCAAGAGGCCAGGAACCAGGACAGTGACAACACCCAGCC 2397

MH733488.2018 GCAACAAGATCAGGACCACACTCAAGAGGCCAGGAACCAGGACAGTGACAACACCCAGCC 2391

MH733491.2018 GCAACAAGATCAGGACCACACTCAAGAGGCCAGGAACCAGGACAGTGACAACACCCAGCC 2386

MH733478.2018 GCAACAAGATCAGGACCACACTCAAGAGGCCAGGAACCAGGACAGTGACAACACCCAGCC 2389

MK007330.2018 GCAACAAGATCAAGACCACACTCAAGAGGCCAGGAACCAGGACAGTGACAACACCCAGCC 2388

MK007344.2018 GCAACAAGATCAAGACCACACTCAAGAGGCCAGGAACCAGGACAGTGACAACACCCAGCC 2382

* ***** ** ******* *********** ****** ****************** *

AY142960.1976 AGAACACTCTTTTGAGGAGATGTATCGCCACATTCTAAGATCACAGGGGCCATTTGATGC 2460

KC242791.1977 AGAACACTCTTTTGAGGAGATGTATCGCCACATTCTAAGATCACAGGGGCCATTTGATGC 2460

AF499101.1976 AGAACACTCTTTTGAGGAGATGTACCGCCACATTCTAAGATCACAGGGGCCATTTGATGC 2460

KF113528.2003 AGAACACTCTTTTGAGGAGATGTATCGCCACATTCTAAGATCACAGGGACCATTTGATGC 2456

KC242800.2002 AGAACACTCTTTTGAGGAGATGTATCGCCACATTCTAAGATCACAGGGACCATTTGATGC 2460

KY471090.2001 AGAACACTCTTTTGAGGAGATGTATCGCCACATTCTAAGATCACAGGGACCATTTGATGC 2437

KY471092.2001 AGAACACTCTTTTGAGGAGATGTATCGCCACATTCTAAGATCACAGGGACCATTTGATGC 2437

MH481611.2017 AGAACACTCTTTTGAGGAGATGTATCGCCACATTCTAAGATCACAGGGGCCATTTGATGC 2426

MH613311.2017 AGAACACTCTTTTGAGGAGATGTATCGCCACATTCTAAGATCACAGGGGCCATTTGATGC 2405

KC242792.1994 AGAACACTCTTTTGAGGAGATGTATCGCCACATTCTAAGATCACAGGGGCCATTTGATGC 2460

KC242793.1996 AGAACACTCTTTTGAGGAGATGTATCGCCACATTCTAAGATCACAGGGGCCATTTGATGC 2460

KC242794.1996 AGAACACTCTTTTGAGGAGATGTATCGCCACATTCTAAGATCACAGGGGCCATTTGATGC 2460

MH121164.1995 AGAACACTCTTTTGAGGAGATGTATCGCCACATTCTAAGATCACAGGGGCCATTTGATGC 2460

AY354458.1995 AGAACACTCTTTTGAGGAGATGTATCGCCACATTCTAAGATCACAGGGGCCATTTGATGC 2460

KT762962.1995 AGAACACTCTTTTGAGGAGATGTATCGCCACATTCTAAGATCACAGGGGCCATTTGATGC 2460

HQ613402.2008 AGAACACTCTTTTGAGGAGATGTATCGCCACATTCTAAGATCACAAGGGCCATTTGATGC 2379

KC242789.2007 AGAACACTCTTTTGAGGAGATGTATCGCCACATTCTAAGATCACAAGGGCCATTTGATGC 2460

HQ613403.2007 AGAACACTCTTTTGAGGAGATGTATCGCCACATTCTAAGATCACAAGGGCCATTTGATGC 2412

KC242785.2007 AGAACACTCTTTTGAGGAGATGTATCGCCACATTCTAAGATCACAAGGGCCATTTGATGC 2460

KC242790.2007 AGAACACTCTTTTGAGGAGATGTATCGCCACATTCTAAGATCACAAGGGCCATTTGATGC 2460

KU143789.2014 AGAACATTCTTTTGAGGAGATGTATCGCCACATTCTAAGATCACAGGGGCCATTTGATGC 2460

KR817168.2014 AGAACATTCTTTTGAGGAGATGTATCGCCACATTCTAAGATCACAGGGGCCATTTGATGC 2434

KY426696.2015 AGAACATTCTTTTGAGGAGATGTATCGCCACATTCTAAGATCACAGGGGCCATTTGATGC 2460

KR105271.2014 AGAACATTCTTTTGAGGAGATGTATCGCCACATTCTAAGATCACAGGGGCCATTTGATGC 2427

KY007522.2016 AGAACATTCTTTTGAGGAGATGTATCGCCACATTCTAAGATCACAGGGGCCATTTGATGC 2440

KM034555.2014 AGAACATTCTTTTGAGGAGATGTATCGCCACATTCTAAGATCACAGGGGCCATTTGATGC 2452

MH470381.2015 AGAACATTCTTTTGAGGAGATGTATCGCCACATTCTAAGATCACAGGGGCCATTTGATGC 2424

MH470382.2015 AGAACATTCTTTTGAGGAGATGTATCGCCACATTCTAAGATCACAGGGGCCATTTGATGC 2424

MF102255.2014 AGAACATTCTTTTGAGGAGATGTATCGCCACATTCTAAGATCACAGGGGCCATTTGATGC 2437

KJ660348.2014 AGAACATTCTTTTGAGGAGATGTATCGCCACATTCTAAGATCACAGGGGCCATTTGATGC 2460

KU143818.2014 AGAACATTCTTTTGAGGAGATGTATCGCCACATTCTAAGATCACAGGGGCCATTTGATGC 2460

KT725333.2014 AGAACATTCTTTTGAGGAGATGTATCGCCACATTCTAAGATCACAGGGGCCATTTGATGC 2425

KR819004.2014 AGAACACTCTTTTGAGGAGATGTATCGCCACATTCTAAGATCACAAGGGCCATTTGATGC 2407

KP271020.2014 AGAACACTCTTTTGAGGAGATGTATCGCCACATTCTAAGATCACAAGGGCCATTTGATGC 2407

KM519951.2014 AGAACACTCTTTTGAGGAGATGTATCGCCACATTCTAAGATCACAAGGGCCATTTGATGC 2457

MH733488.2018 AGAACACTCTTTTGAGGAGATGTATCGCCACATTCTAAGATCACAGGGGCCATTTGATGC 2451

MH733491.2018 AGAACACTCTTTTGAGGAGATGTATCGCCACATTCTAAGATCACAGGGGCCATTTGATGC 2446

MH733478.2018 AGAACACTCTTTTGAGGAGATGTATCGCCACATTCTAAGATCACAGGGGCCATTTGATGC 2449

MK007330.2018 AGAACACTCTTTTGAGGAGATGTATCGCCACATTTTAAGATCACAGGGGCCATTTGATGC 2448

MK007344.2018 AGAACACTCTTTTGAGGAGATGTATCGCCACATTTTAAGATCACAGGGGCCATTTGATGC 2442

****** ***************** ********* ********** ** ***********

AY142960.1976 TGTTTTGTATTATCATATGATGAAGGATGAGCCTGTAGTTTTCAGTACCAGTGATGGCAA 2520

KC242791.1977 TGTTTTGTATTATCATATGATGAAGGATGAGCCTGTAGTTTTCAGTACCAGTGATGGCAA 2520

AF499101.1976 TGTTTTGTATTATCATATGATGAAGGATGAGCCTGTAGTTTTCAGTACCAGTGATGGCAA 2520

KF113528.2003 TGTTTTGTATTATCATATGATGAAGGATGAGCCTGTAGTTTTCAGTACTAGTGATGGCAA 2516

KC242800.2002 TGTTTTGTATTATCATATGATGAAGGATGAGCCTGTAGTTTTCAGTACTAGTGATGGCAA 2520

KY471090.2001 TGTTTTGTATTATCATATGATGAAGGATGAGCCTGTAGTTTTCAGTACTAGTGATGGCAA 2497

KY471092.2001 TGTTTTGTATTATCATATGATGAAGGATGAGCCTGTAGTTTTCAGTACTAGTGATGGCAA 2497

MH481611.2017 TGTTTTGTATTATCATATGATGAAGGATGAACCTGTAGTTTTCAGCACCAGTGATGGCAA 2486

MH613311.2017 TGTTTTGTATTATCATATGATGAAGGATGAACCTGTAGTTTTCAGCACCAGTGATGGCAA 2465

KC242792.1994 TGTTTTGTATTATCATATGATGAAGGATGAGCCTGTAGTTTTCAGTACCAGTGATGGCAA 2520

KC242793.1996 TGTTTTGTATTATCATATGATGAAGGATGAGCCTGTAGTTTTCAGTACCAGTGATGGCAA 2520

KC242794.1996 TGTTTTGTATTATCATATGATGAAGGATGAGCCTGTAGTTTTCAGTACCAGTGATGGCAA 2520

MH121164.1995 TGTTTTGTATTATCATATGATGAAGGATGAGCCTGTAGTTTTCAGTACCAGTGATGGCAA 2520

AY354458.1995 TGTTTTGTATTATCATATGATGAAGGATGAGCCTGTAGTTTTCAGTACCAGTGATGGCAA 2520

KT762962.1995 TGTTTTGTATTATCATATGATGAAGGATGAGCCTGTAGTTTTCAGTACCAGTGATGGCAA 2520

HQ613402.2008 TGTTTTGTATTATCATATGATGAAGGATGAGCCTGTAGTTTTCAGTACCAGTGATGGCAA 2439

KC242789.2007 TGTTTTGTATTATCATATGATGAAGGATGAGCCTGTAGTTTTCAGTACCAGTGATGGCAA 2520

HQ613403.2007 TGTTTTGTATTATCATATGATGAAGGATGAGCCTGTAGTTTTCAGTACCAGTGATGGCAA 2472

KC242785.2007 TGTTTTGTATTATCATATGATGAAGGATGAGCCTGTAGTTTTCAGTACCAGTGATGGCAA 2520

KC242790.2007 TGTTTTGTATTATCATATGATGAAGGATGAGCCTGTAGTTTTCAGTACCAGTGATGGCAA 2520

KU143789.2014 CGTTTTGTATTATCATATGATGAAGGATGAGCCTGTAGTTTTCAGTACCAGTGATGGTAA 2520

KR817168.2014 CGTTTTGTATTATCATATGATGAAGGATGAGCCTGTAGTTTTCAGTACCAGTGATGGTAA 2494

KY426696.2015 CGTTTTGTATTATCATATGATGAAGGATGAGCCTGTAGTTTTCAGTACCAGTGATGGTAA 2520

KR105271.2014 CGTTTTGTATTATCATATGATGAAGGATGAGCCTGTAGTTTTCAGTACCAGTGATGGTAA 2487

KY007522.2016 CGTTTTGTATTATCATATGATGAAGGATGAGCCTGTAGTTTTCAGTACCAGTGATGGTAA 2500

KM034555.2014 CGTTTTGTATTATCATATGATGAAGGATGAGCCTGTAGTTTTCAGTACCAGTGATGGTAA 2512

MH470381.2015 CGTTTTGTATTATCATATGATGAAGGATGAGCCTGTAGTTTTCAGTACCAGTGATGGTAA 2484

MH470382.2015 CGTTTTGTATTATCATATGATGAAGGATGAGCCTGTAGTTTTCAGTACCAGTGATGGTAA 2484

MF102255.2014 CGTTTTGTATTATCATATGATGAAGGATGAGCCTGTAGTTTTCAGTACCAGTGATGGTAA 2497

KJ660348.2014 CGTTTTGTATTATCATATGATGAAGGATGAGCCTGTAGTTTTCAGTACCAGTGATGGTAA 2520

KU143818.2014 CGTTTTGTATTATCATATGATGAAGGATGAGCCTGTAGTTTTCAGTACCAGTGATGGTAA 2520

KT725333.2014 CGTTTTGTATTATCATATGATGAAGGATGAGCCTGTAGTTTTCAGTACCAGTGATGGTAA 2485

KR819004.2014 TGTTTTGTATTATCATATGATGAAGGATGAGCCTGTAGTTTTCAGTACCAGTGATGGCAA 2467

KP271020.2014 TGTTTTGTATTATCATATGATGAAGGATGAGCCTGTAGTTTTCAGTACCAGTGATGGCAA 2467

KM519951.2014 TGTTTTGTATTATCATATGATGAAGGATGAGCCTGTAGTTTTCAGTACCAGTGATGGCAA 2517

MH733488.2018 TGTTTTGTATTATCATATGATGAAGGATGAGCCTGTAGTTTTCAGTACCAGTGATGGCAA 2511

MH733491.2018 TGTTTTGTATTATCATATGATGAAGGATGAGCCTGTAGTTTTCAGTACCAGTGATGGCAA 2506

MH733478.2018 TGTTTTGTATTATCATATGATGAAGGATGAGCCTGTAGTTTTCAGTACCAGTGATGGCAA 2509

MK007330.2018 TGTTTTGTATTATCATATGATGAAGGATGAGCCTGTAGTTTTCAGTACCAGTGATGGCAA 2508

MK007344.2018 TGTTTTGTATTATCATATGATGAAGGATGAGCCTGTAGTTTTCAGTACCAGTGATGGCAA 2502

***************************** ************** ** ******** **

AY142960.1976 AGAGTACACGTATCCAGACTCCCTTGAAGAGGAATATCCACCATGGCTCACTGAAAAAGA 2580

KC242791.1977 AGAGTACACGTATCCAGACTCCCTTGAAGAGGAATATCCACCATGGCTCACTGAAAAAGA 2580

AF499101.1976 AGAGTACACGTATCCAGACTCCCTTGAAGAGGAATATCCACCATGGCTCACTGAAAAAGA 2580

KF113528.2003 AGAGTACACGTATCCGGACTCCCTTGAAGAGGAATATCCACCATGGCTCACTGAAAAAGA 2576

KC242800.2002 AGAGTACACGTATCCGGACTCCCTTGAAGAGGAATATCCACCATGGCTCACTGAAAAAGA 2580

KY471090.2001 AGAGTACACGTATCCGGACTCCCTTGAAGAGGAATATCCACCATGGCTCACTGAAAAAGA 2557

KY471092.2001 AGAGTACACGTATCCGGACTCCCTTGAAGAGGAATATCCACCATGGCTCACTGAAAAAGA 2557

MH481611.2017 AGAGTACACGTATCCAGACTCCCTTGAAGAGGAATATCCACCATGGCTCACTGAAAAAGA 2546

MH613311.2017 AGAGTACACGTATCCAGACTCCCTTGAAGAGGAATATCCACCATGGCTCACTGAAAAAGA 2525

KC242792.1994 AGAGTACACGTATCCAGACTCCCTTGAAGAGGAATATCCACCATGGCTCACTGAAAAAGA 2580

KC242793.1996 AGAGTACACGTATCCAGACTCCCTTGAAGAGGAATATCCACCATGGCTCACTGAAAAAGA 2580

KC242794.1996 AGAGTACACGTATCCCGACTCCCTTGAAGAGGAATATCCACCATGGCTCACTGAAAAAGA 2580

MH121164.1995 AGAGTACACGTATCCAGACTCCCTTGAAGAGGAATATCCACCATGGCTCACTGAAAAAGA 2580

AY354458.1995 AGAGTACACGTATCCAGACTCCCTTGAAGAGGAATATCCACCATGGCTCACTGAAAAAGA 2580

KT762962.1995 AGAGTACACGTATCCAGACTCCCTTGAAGAGGAATATCCACCATGGCTCACTGAAAAAGA 2580

HQ613402.2008 AGAGTACACGTATCCGGACTCCCTTGAAGAGGAATATCCACCATGGCTCACTGAAAAAGA 2499

KC242789.2007 AGAGTACACGTATCCGGACTCCCTTGAAGAGGAATATCCACCATGGCTCACTGAAAAAGA 2580

HQ613403.2007 AGAGTACACGTATCCGGACTCCCTTGAAGAGGAATATCCACCATGGCTCACTGAAAAAGA 2532

KC242785.2007 AGAGTACACGTATCCGGACTCCCTTGAAGAGGAATATCCACCATGGCTCACTGAAAAAGA 2580

KC242790.2007 AGAGTACACGTATCCGGACTCCCTTGAAGAGGAATATCCACCATGGCTCACTGAAAAAGA 2580

KU143789.2014 AGAGTACACGTATCCGGACTCCCTTGAAGAGGAATATCCACCATGGCTCACTGAAAAAGA 2580

KR817168.2014 AGAGTACACGTATCCGGACTCCCTTGAAGAGGAATATCCACCATGGCTCACTGAAAAAGA 2554

KY426696.2015 AGAGTACACGTATCCGGACTCCCTTGAAGAGGAATATCCACCATGGCTCACTGAAAAAGA 2580

KR105271.2014 AGAGTACACGTATCCGGACTCCCTTGAAGAGGAATATCCACCATGGCTCACTGAAAAAGA 2547

KY007522.2016 AGAGTACACGTATCCGGACTCCCTTGAAGAGGAATATCCACCATGGCTCACTGAAAAAGA 2560

KM034555.2014 AGAGTACACGTATCCGGACTCCCTTGAAGAGGAATATCCACCATGGCTCACTGAAAAAGA 2572

MH470381.2015 AGAGTACACGTATCCGGACTCCCTTGAAGAGGAATATCCACCATGGCTCACTGAAAAAGA 2544

MH470382.2015 AGAGTACACGTATCCGGACTCCCTTGAAGAGGAATATCCACCATGGCTCACTGAAAAAGA 2544

MF102255.2014 AGAGTACACGTATCCGGACTCCCTTGAAGAGGAATATCCACCATGGCTCACTGAAAAAGA 2557

KJ660348.2014 AGAGTACACGTATCCGGACTCCCTTGAAGAGGAATATCCACCATGGCTCACTGAAAAAGA 2580

KU143818.2014 AGAGTACACGTATCCGGACTCCCTTGAAGAGGAATATCCACCATGGCTCACTGAAAAAGA 2580

KT725333.2014 AGAGTACACGTATCCGGACTCCCTTGAAGAGGAATATCCACCATGGCTCACTGAAAAAGA 2545

KR819004.2014 AGAGTACACGTATCCAGACTCCCTTGAAGAGGAATATCCACCATGGCTCACTGAAAAAGA 2527

KP271020.2014 AGAGTACACGTATCCAGACTCCCTTGAAGAGGAATATCCACCATGGCTCACTGAAAAAGA 2527

KM519951.2014 AGAGTACACGTATCCAGACTCCCTTGAAGAGGAATATCCACCATGGCTCACTGAAAAAGA 2577

MH733488.2018 AGAGTACACGTATCCAGACTCCCTTGAAGAGGAATATCCACCATGGCTCACTGAAAAAGA 2571

MH733491.2018 AGAGTACACGTATCCAGACTCCCTTGAAGAGGAATATCCACCATGGCTCACTGAAAAAGA 2566

MH733478.2018 AGAGTACACGTATCCAGACTCCCTTGAAGAGGAATATCCACCATGGCTCACTGAAAAAGA 2569

MK007330.2018 AGAGTACACGTATCCAGACTCCCTTGAAGAGGAATATCCACCATGGCTCACTGAAAAAGA 2568

MK007344.2018 AGAGTACACGTATCCAGACTCCCTTGAAGAGGAATATCCACCATGGCTCACTGAAAAAGA 2562

*************** ********************************************

AY142960.1976 GGCTATGAATGAAGAGAATAGATTTGTTACATTGGATGGTCAACAATTTTATTGGCCGGT 2640

KC242791.1977 GGCTATGAATGAAGAGAATAGATTTGTTACATTGGATGGTCAACAATTTTATTGGCCGGT 2640

AF499101.1976 GGCTATGAATGAAGAGAATAGATTTGTTACATTGGATGGTCAACAATTTTATTGGCCGGT 2640

KF113528.2003 GGCCATGAATGAAGAGAATAGATTTGTTACATTGGATGGTCAACAATTTTATTGGCCGGT 2636

KC242800.2002 GGCCATGAATGAAGAGAATAGATTTGTTACATTGGATGGTCAACAATTTTATTGGCCGGT 2640

KY471090.2001 GGCCATGAATGAAGAGAATAGATTTGTTACATTGGATGGTCAACAATTTTATTGGCCGGT 2617

KY471092.2001 GGCCATGAATGAAGAGAATAGATTTGTTACATTGGATGGTCAACAATTTTATTGGCCGGT 2617

MH481611.2017 GGCTATGAATGAAGAGAATAGATTTGTTACATTGGATGGTCAACAATTTTATTGGCCGGT 2606

MH613311.2017 GGCTATGAATGAAGAGAATAGATTTGTTACATTGGATGGTCAACAATTTTATTGGCCGGT 2585

KC242792.1994 GGCTATGAATGAAGAGAATAGATTTGTTACATTGGATGGTCAACAATTTTATTGGCCGGT 2640

KC242793.1996 GGCTATGAATGAAGAGAATAGATTTGTTACATTGGATGGTCAACAATTTTATTGGCCGGT 2640

KC242794.1996 GGCTATGAATGAAGAGAATAGATTTGTTACATTGGATGGTCAACAATTTTATTGGCCGGT 2640

MH121164.1995 GGCTATGAATGAAGAGAATAGATTTGTTACATTGGATGGTCAACAATTTTATTGGCCGGT 2640

AY354458.1995 GGCTATGAATGAAGAGAATAGATTTGTTACATTGGATGGTCAACAATTTTATTGGCCGGT 2640

KT762962.1995 GGCTATGAATGAAGAGAATAGATTTGTTACATTGGATGGTCAACAATTTTATTGGCCGGT 2640

HQ613402.2008 GGCCATGAATGAAGAGAATAGATTTGTTACATTGGATGGTCAACAATTTTATTGGCCGGT 2559

KC242789.2007 GGCCATGAATGAAGAGAATAGATTTGTTACATTGGATGGTCAACAATTTTATTGGCCGGT 2640

HQ613403.2007 GGCCATGAATGAAGAGAATAGATTTGTTACATTGGATGGTCAACAATTTTATTGGCCGGT 2592

KC242785.2007 GGCCATGAATGAAGAGAATAGATTTGTTACATTGGATGGTCAACAATTTTATTGGCCGGT 2640

KC242790.2007 GGCCATGAATGAAGAGAATAGATTTGTTACATTGGATGGTCAACAATTTTATTGGCCGGT 2640

KU143789.2014 GGCCATGAATGATGAGAATAGATTTGTTACACTGGATGGTCAACAATTTTATTGGCCAGT 2640

KR817168.2014 GGCCATGAATGATGAGAATAGATTTGTTACACTGGATGGTCAACAATTTTATTGGCCAGT 2614

KY426696.2015 GGCCATGAATGATGAGAATAGATTTGTTACACTGGATGGTCAACAATTTTATTGGCCAGT 2640

KR105271.2014 GGCCATGAATGATGAGAATAGATTTGTTACACTGGATGGTCAACAATTTTATTGGCCAGT 2607

KY007522.2016 GGCCATGAATGATGAGAATAGATTTGTTACACTGGATGGTCAACAATTTTATTGGCCAGT 2620

KM034555.2014 GGCCATGAATGATGAGAATAGATTTGTTACACTGGATGGTCAACAATTTTATTGGCCAGT 2632

MH470381.2015 GGCCATGAATGATGAGAATAGATTTGTTACACTGGATGGTCAACAATTTTATTGGCCAGT 2604

MH470382.2015 GGCCATGAATGATGAGAATAGATTTGTTACACTGGATGGTCAACAATTTTATTGGCCAGT 2604

MF102255.2014 GGCCATGAATGATGAGAATAGATTTGTTACACTGGATGGTCAACAATTTTATTGGCCAGT 2617

KJ660348.2014 GGCCATGAATGATGAGAATAGATTTGTTACACTGGATGGTCAACAATTTTATTGGCCAGT 2640

KU143818.2014 GGCCATGAATGATGAGAATAGATTTGTTACACTGGATGGTCAACAATTTTATTGGCCAGT 2640

KT725333.2014 GGCCATGAATGATGAGAATAGATTTGTTACACTGGATGGTCAACAATTTTATTGGCCAGT 2605

KR819004.2014 GGCTATGAATGAAGAGAATAGATTTGTTACATTGGATGGTCAACAATTTTATTGGCCGGT 2587

KP271020.2014 GGCTATGAATGAAGAGAATAGATTTGTTACATTGGATGGTCAACAATTTTATTGGCCGGT 2587

KM519951.2014 GGCTATGAATGAAGAGAATAGATTTGTTACATTGGATGGTCAACAATTTTATTGGCCGGT 2637

MH733488.2018 GGCTATGAATGAAGAGAATAGATTTGTTACATTGGATGGTCAACAATTTTATTGGCCGGT 2631

MH733491.2018 GGCTATGAATGAAGAGAATAGATTTGTTACATTGGATGGTCAACAATTTTATTGGCCGGT 2626

MH733478.2018 GGCTATGAATGAAGAGAATAGATTTGTTACATTGGATGGTCAACAATTTTATTGGCCGGT 2629

MK007330.2018 GGCTATGAATGAAGAGAATAGATTTGTTACATTGGATGGTCAACAATTTTATTGGCCGGT 2628

MK007344.2018 GGCTATGAATGAAGAGAATAGATTTGTTACATTGGATGGTCAACAATTTTATTGGCCGGT 2622

*** ******** ****************** ************************* **

AY142960.1976 GATGAATCACAAGAATAAATTCATGGCAATCCTGCAACATCATCAGTGAATGAGCATGGA 2700 end NP

KC242791.1977 GATGAATCACAAGAATAAATTCATGGCAATCCTGCAACATCATCAGTGAATGAGCATGGA 2700

AF499101.1976 GATGAATCACAAGAATAAATTCATGGCAATCCTGCAACATCATCAGTGAATGAGCATGGA 2700

KF113528.2003 AATGAATCACAAGAATAAATTCATGGCAATCCTGCAACATCATCAGTGAATGAGAATGGA 2696

KC242800.2002 AATGAATCACAAGAATAAATTCATGGCAATCCTGCAACATCATCAGTGAATGAGAATGGA 2700

KY471090.2001 AATGAATCACAAGAATAAATTCATGGCAATCCTGCAACATCATCAGTGAATGAGAATGGA 2677

KY471092.2001 AATGAATCACAAGAATAAATTCATGGCAATCCTGCAACATCATCAGTGAATGAGAATGGA 2677

MH481611.2017 AATGAATCACAAGAATAAATTCATGGCAATCCTGCAACATCATCAGTGAATGAGCATGGA 2666

MH613311.2017 AATGAATCACAAGAATAAATTCATGGCAATCCTGCAACATCATCAGTGAATGAGCATGGA 2645

KC242792.1994 AATGAATCACAAGGATAAATTCATGGCAATCCTGCAACATCATCAGTGAATGAGCATGGA 2700

KC242793.1996 AATGAATCACAAGAATAAATTCATGGCAATCCTGCAACATCATCAGTGAATGAGCATGGA 2700

KC242794.1996 AATGAATCACAAGAATAAATTCATGGCAATCCTGCAACATCATCAGTGAATGAGCATGGA 2700

MH121164.1995 AATGAATCACAAGAATAAATTCATGGCAATCCTGCAACATCATCAGTGAATGAGCATGGA 2700

AY354458.1995 AATGAATCACAAGAATAAATTCATGGCAATCCTGCAACATCATCAGTGAATGAGCATGGA 2700

KT762962.1995 AATGAATCACAAGAATAAATTCATGGCAATCCTGCAACATCATCAGTGAATGAGCATGGA 2700

HQ613402.2008 AATGAATCACAAGAACAAATTCATGGCAATCCTGCAACATCATCAGTGAATGAGCATGGA 2619

KC242789.2007 AATGAATCACAAGAATAAATTCATGGCAATCCTGCAACATCATCAGTGAATGAGCATGGA 2700

HQ613403.2007 AATGAATCACAAGAATAAATTCATGGCAATCCTGCAACATCATCAGTGAATGAGCATGGA 2652

KC242785.2007 AATGAATCACAAGAATAAATTCATGGCAATTCTGCAACATCATCAGTGAATGAGCATGGA 2700

KC242790.2007 AATGAATCACAAGAATAAATTCATGGCAATCCTGCAACATCATCAGTGAATGAGCATGGA 2700

KU143789.2014 AATGAATCACAGGAATAAATTCATGGCAATCCTGCAACATCATCAGTGAATGAGCATGTA 2700

KR817168.2014 AATGAATCACAGGAATAAATTCATGGCAATCCTGCAACATCATCAGTGAATGAGCATGTA 2674

KY426696.2015 AATGAATCACAGGAATAAATTCATGGCAATCCTGCAACATCATCAGTGAATGAGCATGTA 2700

KR105271.2014 AATGAATCACAGGAATAAATTCATGGCAATCCTGCAACATCATCAGTGAATGAGCATGTA 2667

KY007522.2016 AATGAATCACAGGAATAAATTCATGGCAATCCTGCAACATCATCAGTGAATGAGCATGTA 2680

KM034555.2014 AATGAATCACAGGAATAAATTCATGGCAATCCTGCAACATCATCAGTGAATGAGCATGTA 2692

MH470381.2015 AATGAATCACAGGAATAAATTCATGGCAATCCTGCAACATCATCAGTGAATGAGCATGTA 2664

MH470382.2015 AATGAATCACAGGAATAAATTCATGGCAATCCTGCAACATCATCAGTGAATGAGCATGTA 2664

MF102255.2014 AATGAATCACAGGAATAAATTCATGGCAATCCTGCAACATCATCAGTGAATGAGCATGTA 2677

KJ660348.2014 AATGAATCACAGGAATAAATTCATGGCAATCCTGCAACATCATCAGTGAATGAGCATGTA 2700

KU143818.2014 AATGAATCACAGGAATAAATTCATGGCAATCCTGCAACATCATCAGTGAATGAGCATGTA 2700

KT725333.2014 AATGAATCACAGGAATAAATTCATGGCAATCCTGCAACATCATCAGTGAATGAGCATGTA 2665

KR819004.2014 AATGAATCACAAGAATAAATTCATGGCAATCCTGCAACATCATCAGTGAATGAGCATGGA 2647

KP271020.2014 AATGAATCACAAGAATAAATTCATGGCAATCCTGCAACATCATCAGTGAATGAGCATGGA 2647

KM519951.2014 AATGAATCACAAGAATAAATTCATGGCAATCCTGCAACATCATCAGTGAATGAGCATGGA 2697

MH733488.2018 AATGAACCACAAGAATAAATTCATGGCAATCCTGCAACATCATCAGTGAATGAGCATGGA 2691

MH733491.2018 AATGAACCACAAGAATAAATTCATGGCAATCCTGCAACATCATCAGTGAATGAGCATGGA 2686

MH733478.2018 AATGAACCACAAGAATAAATTCATGGCAATCCTGCAACATCATCAGTGAATGAGCATGGA 2689

MK007330.2018 AATGAATCACAAGAATAAATTCATGGCAATCCTGCAACATCATCAGTGAATGAGCATGGA 2688

MK007344.2018 AATGAATCACAAGAATAAATTCATGGCAATCCTGCAACATCATCAGTGAATGAGCATGGA 2682

***** **** * * ************** *********************** *** *

AY142960.1976 ACAATGGGATGATTCAACCGACAAATAGCTAACATTAAGTAGTCAAGGAACGAAAACAGG 2760

KC242791.1977 ACAATGGGATGATTCAACCGACAAATAGCTAACATTAAGTAGTCAAGGAACGAAAACAGG 2760

AF499101.1976 ACAATGGGATGATTCAACCGACAAATAGCTAACATTAAGTAGTCAAGGAACGAAAACAGG 2760

KF113528.2003 ATAATGGGATGATTTAACCGACAAATAGCTAACATTAAATAGTCAAGAAACGCAAACAGG 2756

KC242800.2002 ATAATGGGATGATTTAACCGACAAATAGCTAACATTAAATAGTCAAGAAACGCAAACAGG 2760

KY471090.2001 ATAATGGGATGATTTAACCGACAAATAGCTAACATTAAATAGTCAAGAAACGCAAACAGG 2737

KY471092.2001 ATAATGGGATGATTTAACCGACAAATAGCTAACATTAAATAGTCAAGAAACGCAAACAGG 2737

MH481611.2017 ACAATGGGATGATTTAACCGACAAATAGCTAACATTATGTAGTCAAGGAACGAAAACAGG 2726

MH613311.2017 ACAATGGGATGATTTAACCGACAAATAGCTAACATTATGTAGTCAAGGAACGAAAACAGG 2705

KC242792.1994 ACAATGGGATGATTCAACCGACAAATAGCTAACATTAAGTAGTCAAGGAACGAAAACAGG 2760

KC242793.1996 ACAATGGGATGATTCAACCGACAAATAGCTAACATTAAGTAGTCAAGGAACGAAAACAGG 2760

KC242794.1996 ACAATGGGATGATTCAACCGACAAATAGCTAACATTAAGTAGTCAAGGAACGAAAACAGG 2760

MH121164.1995 ACAATGGGATGATTCAACCGACAAATAGCTAACATTAGGTAGTCAAGGAACGAAAACAGG 2760

AY354458.1995 ACAATGGGATGATTCAACCGACAAATAGCTAACATTAGGTAGTCAAGGAACGAAAACAGG 2760

KT762962.1995 ACAATGGGATGATTCAACCGACAAATAGCTAACATTAGGTAGTCAAGGAACGAAAACAGG 2760

HQ613402.2008 ATAATGGGATGATTTAACCGACAAATAGCTAACATTAAATAGTCAAGGAACGCAAACAGG 2679

KC242789.2007 ATAATGGGATGATTTAACCGACAAATAGCTAACATTAAATAGTCAAGGAACGCAAACAGG 2760

HQ613403.2007 ATAATGGGATGATTTAACCGACAAATAGCTAACATTAAATAGTCAAGGAACGCAAACAGG 2712

KC242785.2007 ATAATGGGATGATTTAACCGACAAATAGCTAACATTAAATAGTCAAGGAACGCAAACAGG 2760

KC242790.2007 ATAATGGGATGATTTAACCGACAAATAGCTAACATTAAATAGTCAAGGAACGCAAACAGG 2760

KU143789.2014 ATAATGGGATGATTTAATCGACAAATAGCTAACATTAAATAGTCAAGGAACGCAAACAGG 2760

KR817168.2014 ATAATGGGATGATTTAATCGACAAATAGCTAACATTAAATAGTCAAGGAACGCAAACAGG 2734

KY426696.2015 ATAATGGGATGATTTAATCGACAAATAGCTAACATTAAATAGTCAAGGAACGCAAACAGG 2760

KR105271.2014 ATAATGGGATGATTTAATCGACAAATAGCTAACATTAAATAGTCAAGGAACGCAAACAGG 2727

KY007522.2016 ATAATGGGATGATTTAATCGACAAATAGCTAACATTAAATAGTCAAGGAACGCAAACAGG 2740

KM034555.2014 ATAATGGGATGATTTAATCGACAAATAGCTAACATTAAATAGTCAAGGAACGCAAACAGG 2752

MH470381.2015 ATAATGGGATGATTTAATCGACAAATAGCTAACATTAAATAGTCAAGGAACGCAAACAGG 2724

MH470382.2015 ATAATGGGATGATTTAATCGACAAATAGCTAACATTAAATAGTCAAGGAACGCAAACAGG 2724

MF102255.2014 ATAATGGGATGATTTAATCGACAAATAGCTAACATTAAATAGTCAAGGAACGCAAACAGG 2737

KJ660348.2014 ATAATGGGATGATTTAATCGACAAATAGCTAACATTAAATAGTCAAGGAACGCAAACAGG 2760

KU143818.2014 ATAATGGGATGATTTAATCGACAAATAGCTAACATTAAATAGTCAAGGAACGCAAACAGG 2760

KT725333.2014 ATAATGGGATGATTTAATCGACAAATAGCTAACATTAAATAGTCAAGGAACGCAAACAGG 2725

KR819004.2014 ACAACGGGATGATTCAACCGACAAATAGCTAACATTAAGTAGTCAAGGAACGAAAACAGG 2707

KP271020.2014 ACAACGGGATGATTCAACCGACAAATAGCTAACATTAAGTAGTCAAGGAACGAAAACAGG 2707

KM519951.2014 ACAACGGGATGATTCAACCGACAAATAGCTAACATTAAGTAGTCAAGGAACGAAAACAGG 2757

MH733488.2018 ACAATGGGATGATTCAACCGACAAATAGCTAACATTAAGTAGTCAAGGAACGAAAACAGG 2751

MH733491.2018 ACAATGGGATGATTCAACCGACAAATAGCTAACATTAAGTAGTCAAGGAACGAAAACAGG 2746

MH733478.2018 ACAATGGGATGATTCAACCGACAAATAGCTAACATTAAGTAGTCAAGGAACGAAAACAGG 2749

MK007330.2018 ACAATGGGATGATTCAACCGACAAATAGCTAACATTAAGTAGTCAAGGAACGAAAACAGG 2748

MK007344.2018 ACAATGGGATGATTCAACCGACAAATAGCTAACATTAAGTAGTCAAGGAACGAAAACAGG 2742

* ** ********* ** ******************* ******** **** *******

AY142960.1976 AAGAATTTTTGATGTCTAAGGTGTGAATTATTATCACAATAAAAGTGATTCTTATTTTTG 2820

KC242791.1977 AAGAATTTTTGATGTCTAAGGTGTGAATTATTATCACAATAAAAGTGATTCTTATTTTTG 2820

AF499101.1976 AAGAATTTTTGATGTCTAAGGTGTGAATTATTATCACAATAAAAGTGATTCTTATTTTTG 2820

KF113528.2003 AAGAATTTTTGATGTCTAAGGTGTGAATTATTATCACAATAAAAGTGATTCTTATTTTTG 2816

KC242800.2002 AAGAATTTTTGATGTCTAAGGTGTGAATTATTATCACAATAAAAGTGATTCTTATTTTTG 2820

KY471090.2001 AAGAATTTTTGATGTCTAAGGTGTGAATTATTATCACAATAAAAGTGATTCTTATTTTTG 2797

KY471092.2001 AAGAATTTTTGATGTCTAAGGTGTGAATTATTATCACAATAAAAGTGATTCTTATTTTTG 2797

MH481611.2017 AGGAATTTTTGATGTCTAAGGTGTGAATTATTATCACAATAAAAGTGATTCTTATTTTTG 2786

MH613311.2017 AGGAATTTTTGATGTCTAAGGTGTGAATTATTATCACAATAAAAGTGATTCTTATTTTTG 2765

KC242792.1994 AAGAATTTTTGATGTCTAAGGTGTGAATTATTATCACAATAAAAGTGATTCTTATTTTTG 2820

KC242793.1996 AAGAATTTTTGATGTCTAAGGTGTGAATTATTATCACAATAAAAGTGATTCTTATTTTTG 2820

KC242794.1996 AAGAATTTTTGATGTCTAAGGTGTGAATTATTATCATAATAAAAGTGATTCTTATTTTTG 2820

MH121164.1995 AAGAATTTTTGATGTCTAAGGTGTGAATTATTATCACAATAAAAGTGATTCTTATTTTTG 2820

AY354458.1995 AAGAATTTTTGATGTCTAAGGTGTGAATTATTATCACAATAAAAGTGATTCTTATTTTTG 2820

KT762962.1995 AAGAATTTTTGATGTCTAAGGTGTGAATTATTATCACAATAAAAGTGATTCTTATTTTTG 2820

HQ613402.2008 AAGAATTTTTGATGTCTAAGGTGTGAATTATTATCACAATAAAAGTGATTCTTATTTTTG 2739

KC242789.2007 AAGAATTTTTGATGTCTAAGGTGTGAATTATTATCACAATAAAAGTGATTCTTATTTTTG 2820

HQ613403.2007 AAGAATTTTTGATGTCTAAGGTGTGAATTATTATCACAATAAAAGTGATTCTTATTTTTG 2772

KC242785.2007 AAGAATTTTTGATGTCTAAGGTGTGAATTATTATCACAATAAAAGTGATTCTTATTTTTG 2820

KC242790.2007 AAGAATTTTTGATGTCTAAGGTGTGAATTATTATCATAATAAAAGTGATTCTTATTTTTG 2820

KU143789.2014 AAGAATTTTTGATGTCTAAGGTGTGAATTATTATCACAATAAAAGTGATTCTTAGTTTTG 2820

KR817168.2014 AAGAATTTTTGATGTCTAAGGTGTGAATTATTATCACAATAAAAGTGATTCTTAGTTTTG 2794

KY426696.2015 AAGAATTTTTGATGTCTAAGGTGTGAATTATTATCACAATAAAAGTGATTCTTAGTTTTG 2820

KR105271.2014 AAGAATTTTTGATGTCTAAGGTGTGAATTATTATCACAATAAAAGTGATTCTTAGTTTTG 2787

KY007522.2016 AAGAATTTTTGATGTCTAAGGTGTGAATTATTATCACAATAAAAGTGATTCTTAGTTTTG 2800

KM034555.2014 AAGAATTTTTGATGTCTAAGGTGTGAATTATTATCACAATAAAAGTGATTCTTAGTTTTG 2812

MH470381.2015 AAGAATTTTTGATGTCTAAGGTGTGAATTATTATCACAATAAAAGTGATTCTTAGTTTTG 2784

MH470382.2015 AAGAATTTTTGATGTCTAAGGTGTGAATTATTATCACAATAAAAGTGATTCTTAGTTTTG 2784

MF102255.2014 AAGAATTTTTGATGTCTAAGGTGTGAATTATTATCACAATAAAAGTGATTCTTAGTTTTG 2797

KJ660348.2014 AAGAATTTTTGATGTCTAAGGTGTGAATTATTATCACAATAAAAGTGATTCTTAGTTTTG 2820

KU143818.2014 AAGAATTTTTGATGTCTAAGGTGTGAATTATTATCACAATAAAAGTGATTCTTAGTTTTG 2820

KT725333.2014 AAGAATTTTTGATGTCTAAGGTGTGAATTATTATCACAATAAAAGTGATTCTTAGTTTTG 2785

KR819004.2014 AAGAATTTTTGATGTCTAAGGTGTGAATTATTATCACAATAAAAGTGATTCTTATTTTTG 2767

KP271020.2014 AAGAATTTTTGATGTCTAAGGTGTGAATTATTATCACAATAAAAGTGATTCTTATTTTTG 2767

KM519951.2014 AAGAATTTTTGATGTCTAAGGTGTGAATTATTATCACAATAAAAGTGATTCTTATTTTTG 2817

MH733488.2018 AAGAATTTTTGATGTCTAAGGTGTGAATTATTATCACAATAAAAGTGATTTTTATTTTTG 2811

MH733491.2018 AAGAATTTTTGATGTCTAAGGTGTGAATTATTATCACAATAAAAGTGATTTTTATTTTTG 2806

MH733478.2018 AAGAATTTTTGATGTCTAAGGTGTGAATTATTATCACAATAAAAGTGATTTTTATTTTTG 2809

MK007330.2018 AAGAATTCTTGATGTCTAAGGTGTGAATTATTATCACAATAAAAGTGATTCTTATTTTTG 2808

MK007344.2018 AAGAATTCTTGATGTCTAAGGTGTGAATTATTATCACAATAAAAGTGATTCTTATTTTTG 2802

* ***** **************************** ************* *** *****

AY142960.1976 AATTTAAAGCTAGCTTATTATTACTAGCCGTTTTTCAAAGTTCAATTTGAGTCTTAATGC 2880

KC242791.1977 AATTTAAAGCTAGCTTATTATTACTAGCCGTTTTTCAAAGTTCAATTTGAGTCTTAATGC 2880

AF499101.1976 AATTTAAAGCTAGCTTATTATTACTAGCCGTTTTTCAAAGTTCAATTTGAGTCTTAATGC 2880

KF113528.2003 AATTTAAAGCTAGCTTATTATTACTAGCCGTTTTTCAAAGTTCAATTTGAGTCTTAATGC 2876

KC242800.2002 AATTTAAAGCTAGCTTATTATTACTAGCCGTTTTTCAAAGTTCAATTTGAGTCTTAATGC 2880

KY471090.2001 AATTTAAAGCTAGCTTATTATTACTAGCCGTTTTTCAAAGTTCAATTTGAGTCTTAATGC 2857

KY471092.2001 AATTTAAAGCTAGCTTATTATTACTAGCCGTTTTTCAAAGTTCAATTTGAGTCTTAATGC 2857

MH481611.2017 AATTTAAAGCTAGCTTATTATTACTAGCCGTTTTTCAAAGTTCAATTTGAGTCTTAATGC 2846

MH613311.2017 AATTTAAAGCTAGCTTATTATTACTAGCCGTTTTTCAAAGTTCAATTTGAGTCTTAATGC 2825

KC242792.1994 AATTTAAAGCTAGCTTATTATTACTAGCCGTTTTTCAAAGTTCAATTTGAGTCTTAATGC 2880

KC242793.1996 AATTTAAAGCTAGCTTATTATTACTAGCCGTTTTTCAAAGTTCAATTTGAGTCTTAATGC 2880

KC242794.1996 AATTTAAAGCTAGCTTATTATTACTAGCCGTCCTTCAAAGTTCAATTTGAGTCTTAATGC 2880

MH121164.1995 AATTTAAAGCTAGCTTATTATTACTAGCCGTTTTTCAAAGTCCAATTTGAGTCTTAATGC 2880

AY354458.1995 AATTTAAAGCTAGCTTATTATTACTAGCCGTTTTTCAAAGTCCAATTTGAGTCTTAATGC 2880

KT762962.1995 AATTTAAAGCTAGCTTATTATTACTAGCCGTTTTTCAAAGTCCAATTTGAGTCTTAATGC 2880

HQ613402.2008 AATTTAAAGCTAGCTTATTATTACTAGCCGTTTTTCAAAGTTCAATTTGAGTCTTAATGC 2799

KC242789.2007 AATTTAAAGCTAGCTTATTATTACTAGCCGTTTTTCAAAGTTCAATTTGAGTCTTAATGC 2880

HQ613403.2007 AATTTAAAGCTAGCTTATTATTACTAGCCGTTTTTCAAAGTTCAATTTGAGTCTTAATGC 2832

KC242785.2007 AATTTAAAGCTAGCTTATTATTACTAGCCGTTTTTCAAAGTTCAATTTGAGTCTTAATGC 2880

KC242790.2007 AATTTAAAGCTAGCTTATTATTACTAGCCGTTTTTCAAAGTTCAATTTGAGTCTTAATGC 2880

KU143789.2014 AATTTAAAGCTAGCTTATTATTACTAGCCGTTTTTCAAAGTTCAATTTGAGTCTTAATGC 2880

KR817168.2014 AATTTAAAGCTAGCTTATTATTACTAGCCGTTTTTCAAAGTTCAATTTGAGTCTTAATGC 2854

KY426696.2015 AATTTAAAGCTAGCTTATTATTACTAGCCGTTTTTCAAAGTTCAATTTGAGTCTTAATGC 2880

KR105271.2014 AATTTAAAGCTAGCTTATTATTACTAGCCGTTTTTCAAAGTTCAATTTGAGTCTTAATGC 2847

KY007522.2016 AATTTAAAGCTAGCTTATTATTACTAGCCGTTTTTCAAAGTTCAATTTGAGTCTTAATGC 2860

KM034555.2014 AATTTAAAGCTAGCTTATTATTACTAGCCGTTTTTCAAAGTTCAATTTGAGTCTTAATGC 2872

MH470381.2015 AATTTAAAGCTAGCTTATTATTACTAGCCGTTTTTCAAAGTTCAATTTGAGTCTTAATGC 2844

MH470382.2015 AATTTAAAGCTAGCTTATTATTACTAGCCGTTTTTCAAAGTTCAATTTGAGTCTTAATGC 2844

MF102255.2014 AATTTAAAGCTAGCTTATTATTACTAGCCGTTTTTCAAAGTTCAATTTGAGTCTTAATGC 2857

KJ660348.2014 AATTTAAAGCTAGCTTATTATTACTAGCCGTTTTTCAAAGTTCAATTTGAGTCTTAATGC 2880

KU143818.2014 AATTTAAAGCTAGCTTATTATTACTAGCCGTTTTTCAAAGTTCAATTTGAGTCTTAATGC 2880

KT725333.2014 AATTTAAAGCTAGCTTATTATTACTAGCCGTTTTTCAAAGTTCAATTTGAGTCTTAATGC 2845

KR819004.2014 AATTTAAAGCTAGCTTATTATTACTAGCCGTTTTTCAAAGTTCAATTTGAGTCTTAATGC 2827

KP271020.2014 AATTTAAAGCTAGCTTATTATTACTAGCCGTTTTTCAAAGTTCAATTTGAGTCTTAATGC 2827

KM519951.2014 AATTTAAAGCTAGCTTATTATTACTAGCCGTTTTTCAAAGTTCAATTTGAGTCTTAATGC 2877

MH733488.2018 AATTTAAAGCTAGCTTATTATTACTAGCCGTTTTTCAAAGTTCAATTTGAGTCTTAATGC 2871

MH733491.2018 AATTTAAAGCTAGCTTATTATTACTAGCCGTTTTTCAAAGTTCAATTTGAGTCTTAATGC 2866

MH733478.2018 AATTTAAAGCTAGCTTATTATTACTAGCCGTTTTTCAAAGTTCAATTTGAGTCTTAATGC 2869

MK007330.2018 AATTTAAAGCTAGCTTATTATTACTAGCTGTTTTTCAAAGTTCAATTTGAGTCTTAATGC 2868

MK007344.2018 AATTTAAAGCTAGCTTATTATTACTAGCTGTTTTTCAAAGTTCAATTTGAGTCTTAATGC 2862

**************************** ** ******** ******************

AY142960.1976 AAATAGGCGTTAAGCCACAGTTATAGCCATAATTGTAACTCAATATTCTAACTAGCGATT 2940

KC242791.1977 AAATAGGCGTTAAGCCACAGTTATAGCCATAATTGTAACTCAATATTCTAACTAGCGATT 2940

AF499101.1976 AAATAGGCGTTAAGCCACAGTTATAGCCATAATTGTAACTCAATATTCTAACTAGCGATT 2940

KF113528.2003 AAATAGGCGTTAAGCCACAGTTATAGCCATAATTGTAACTCAATATCTTAGCTAGCGATT 2936

KC242800.2002 AAATAGGCGTTAAGCCACAGTTATAGCCATAATTGTAACTCAATATCTTAGCTAGCGATT 2940

KY471090.2001 AAATAGGCGTTAAGCCACAGTTATAGCCATAATTGTAACTCAATATCTTAGCTAGCGATT 2917

KY471092.2001 AAATAGGCGTTAAGCCACAGTTATAGCCATAATTGTAACTCAATATCTTAGCTAGCGATT 2917

MH481611.2017 AAATAGGCGTTAAGCCACAGTTATAGCCATAATTGTAACTCAATATCCTAACTAGCGATT 2906

MH613311.2017 AAATAGGCGTTAAGCCACAGTTATAGCCATAATTGTAACTCAATATCCTAACTAGCGATT 2885

KC242792.1994 AAATAGGCGTTAAGCCACAGTTATAGCCATAATTGTAACTCAATATCCTAACTAGCGATT 2940

KC242793.1996 AAATAGGCGTTAAGCCACAGTTATAGCCATAATTGTAACTCAATATCCTAACTAGCGATT 2940

KC242794.1996 AAATAGGCGTTAAGCCACAGTTATAGCCATAATTGTAACTCAATATCCTAACTAGCGATT 2940

MH121164.1995 AAATAGGCGTTAAGCCACAGTTATAGCCATAATTGTAACTCAATATCCTAACTAGCGATT 2940

AY354458.1995 AAATAGGCGTTAAGCCACAGTTATAGCCATAATTGTAACTCAATATCCTAACTAGCGATT 2940

KT762962.1995 AAATAGGCGTTAAGCCACAGTTATAGCCATAATTGTAACTCAATATCCTAACTAGCGATT 2940

HQ613402.2008 AAATAGGCGTTAAGCCACAGTTATAACCATAATTGTAACTCAATATCTTAGCTAGTGATT 2859

KC242789.2007 AAATAGGCGTTAAGCCACAGTTATAACCATAATTGTAACTCAATATCTTAGCTAGTGATT 2940

HQ613403.2007 AAATAGGCGTTAAGCCACAGTTATAACCATAATTGTAACTCAATATCTTAGCTAGTGATT 2892

KC242785.2007 AAATAGGCGTTAAGCCACAGTTATAACCATAATTGTAACTCAATATCTTAGCTAGTGATT 2940

KC242790.2007 AAATAGGCGTTAAGCCACAGTTATAACCATAATTGTAACTCAATATCTTAGCTAGTGATT 2940

KU143789.2014 AAATAAGCGTTAAGCCACAGTTATAGCCATAATGGTAACTCAATATCTTAGCCAGCGATT 2940

KR817168.2014 AAATAAGCGTTAAGCCACAGTTATAGCCATAATGGTAACTCAATATCTTAGCCAGCGATT 2914

KY426696.2015 AAATAAGCGTTAAGCCACAGTTATAGCCATAATGGTAACTCAATATCTTAGCCAGCGATT 2940

KR105271.2014 AAATAAGCGTTAAGCCACAGTTATAGCCATAATGGTAACTCAATATCTTAGCCAGCGATT 2907

KY007522.2016 AAATAAGCGTTAAGCCACAGTTATAGCCATAATGGTAACTCAATATCTTAGCCAGCGATT 2920

KM034555.2014 AAATAAGCGTTAAGCCACAGTTATAGCCATAATGGTAACTCAATATCTTAGCCAGCGATT 2932

MH470381.2015 AAATAAGCGTTAAGCCACAGTTATAGCCATAATGGTAACTCAATATCTTAGCCAGCGATT 2904

MH470382.2015 AAATAAGCGTTAAGCCACAGTTATAGCCATAATGGTAACTCAATATCTTAGCCAGCGATT 2904

MF102255.2014 AAATAAGCGTTAAGCCACAGTTATAGCCATAATGGTAACTCAATATCTTAGCCAGCGATT 2917

KJ660348.2014 AAATAAGCGTTAAGCCACAGTTATAGCCATAATGGTAACTCAATATCTTAGCCAGCGATT 2940

KU143818.2014 AAATAAGCGTTAAGCCACAGTTATAGCCATAATGGTAACTCAATATCTTAGCCAGCGATT 2940

KT725333.2014 AAATAAGCGTTAAGCCACAGTTATAGCCATAATGGTAACTCAATATCTTAGCCAGCGATT 2905

KR819004.2014 AAATAGGCGTTAAGCCACAGTTATAGCCATAATTGTAACTCAATATCCTAACTAGCGATT 2887

KP271020.2014 AAATAGGCGTTAAGCCACAGTTATAGCCATAATTGTAACTCAATATCCTAACTAGCGATT 2887

KM519951.2014 AAATAGGCGTTAAGCCACAGTTATAGCCATAATTGTAACTCAATATCCTAACTAGCGATT 2937

MH733488.2018 AAATAGGCGTTAAGCCACAGTTATAGCCATAATTGTAACTCAATATCCTAACTAGCGATT 2931

MH733491.2018 AAATAGGCGTTAAGCCACAGTTATAGCCATAATTGTAACTCAATATCCTAACTAGCGATT 2926

MH733478.2018 AAATAGGCGTTAAGCCACAGTTATAGCCATAATTGTAACTCAATATCCTAACTAGCGATT 2929

MK007330.2018 AAATAGGCGTTAAGCCACAGTTGTAGCCATAATTGTAACTCAATATCTTAACTAGCGATT 2928

MK007344.2018 AAATAGGCGTTAAGCCACAGTTGTAGCCATAATTGTAACTCAATATCTTAACTAGCGATT 2922

***** **************** ** ******* ************ ** * ** ****

AY142960.1976 TATCTAAATTAAATTACATTATGCTTTTATAACTTACCTACTAGCCTGCCCAACATTTAC 3000

KC242791.1977 TATCTAAATTAAATTACATTATGCTTTTATAACTTACCTACTAGCCTGCCCAACATTTAC 3000

AF499101.1976 TATCTAAATTAAATTACATTATGCTTTTATAACTTACCTACTAGCCTGCCCAACATTTAC 3000

KF113528.2003 TATCTAAATTAAATTACATTATGCTTTTATAACTTACCTACTAGCCTGCCCAACATTTAC 2996

KC242800.2002 TATCTAAATTAAATTACATTATGCTTTTATAACTTACCTACTAGCCTGCCCAACATTTAC 3000

KY471090.2001 TATCTAAATTAAATTACATTATGCTTTTATAACTTACCTACTAGCCTGCCCAACATTTAC 2977

KY471092.2001 TATCTAAATTAAATTACATTATGCTTTTATAACTTACCTACTAGCCTGCCCAACATTTAC 2977

MH481611.2017 TATCTGAATTAAATTACATTATGCTTTTATAACTTACCTACTAGCCTGCCCAACATTTAC 2966

MH613311.2017 TATCTGAATTAAATTACATTATGCTTTTATAACTTACCTACTAGCCTGCCCAACATTTAC 2945

KC242792.1994 TATCTAAATTAAATTACATTATGCTTTTATAACTTACCTACTAGCCTACCCAACATTTAC 3000

KC242793.1996 TATCTAAATTAAATTACATTATGCTTTTATAACTTACCTACTAGCCTACCCAACATTTAC 3000

KC242794.1996 TATCTAAATTAAATTACATTATGCTTTTATAACTTACCTACTAGCCTACCCAACATTTAC 3000

MH121164.1995 TATCTAAATTAAATTACATTATGCTTTTATAACTTACCTACTAGCCTACCCAACATTTAC 3000

AY354458.1995 TATCTAAATTAAATTACATTATGCTTTTATAACTTACCTACTAGCCTACCCAACATTTAC 3000

KT762962.1995 TATCTAAATTAAATTACATTATGCTTTTATAACTTACCTACTAGCCTACCCAACATTTAC 3000

HQ613402.2008 TATCTAAATTAAATTACATTATGCTTTTATAACTTACTTATTAGCCCGCCCAACATTTAC 2919

KC242789.2007 TATCTAAATTAAATTACATTATGCTTTTATAACTTACTTATTAGCCCGCCCAACATTTAC 3000

HQ613403.2007 TATCTAAATTAAATTACATTATGCTTTTATAACTTACTTATTAGCCCGCCCAACATTTAC 2952

KC242785.2007 TATCTAAATTAAATTACATTATGCTTTTATAACTTACTTATTAGCCCGCCCAACATTTAC 3000

KC242790.2007 TATCTAAATTAAATTACATTATGCTTTTATAACTTACTTATTAGCCCGCCCAACATTTAC 3000

KU143789.2014 TATCTAAATTAAATTACATTATGCTTTTATAACTTACCTACTAGCCTGCCCAACATTTAC 3000

KR817168.2014 TATCTAAATTAAATTACATTATGCTTTTATAACTTACCTACTAGCCTGCCCAACATTTAC 2974

KY426696.2015 TATCTAAATTAAATTACATTATGCTTTTATAACTTACCTACTAGCCTGCCCAACATTTAC 3000

KR105271.2014 TATCTAAATTAAATTACATTATGCTTTTATAACTTACCTACTAGCCTGCCCAACATTTAC 2967

KY007522.2016 TATCTAAATTAAATTACATTATGCTTTTATAACTTACCTACTAGCCTGCCCAACATTTAC 2980

KM034555.2014 TATCTAAATTAAATTACATTATGCTTTTATAACTTACCTACTAGCCTGCCCAACATTTAC 2992

MH470381.2015 TATCTAAATTAAATTACATTATGCTTTTATAACTTACCTACTAGCCTGCCCAACATTTAC 2964

MH470382.2015 TATCTAAATTAAATTACATTATGCTTTTATAACTTACCTACTAGCCTGCCCAACATTTAC 2964

MF102255.2014 TATCTAAATTAAATTACATTATGCTTTTATAACTTACCTACTAGCCTGCCCAACATTTAC 2977

KJ660348.2014 TATCTAAATTAAATTACATTATGCTTTTATAACTTACCTACTAGCCTGCCCAACATTTAC 3000

KU143818.2014 TATCTAAATTAAATTACATTATGCTTTTATAACTTACCTACTAGCCTGCCCAACATTTAC 3000

KT725333.2014 TATCTAAATTAAATTACATTATGCTTTTATAACTTACCTACTAGCCTGCCCAACATTTAC 2965

KR819004.2014 TATCTAAATTAAATTACATTATGCTTTTATAACTTACCTACTAGCCTACCCAACATTTAC 2947

KP271020.2014 TATCTAAATTAAATTACATTATGCTTTTATAACTTACCTACTAGCCTACCCAACATTTAC 2947

KM519951.2014 TATCTAAATTAAATTACATTATGCTTTTATAACTTACCTACTAGCCTACCCAACATTTAC 2997

MH733488.2018 TATCTAAATTAAATTACATTATGCTTTTATAACTTACCTACTAGCCTACCCAACATTTAC 2991

MH733491.2018 TATCTAAATTAAATTACATTATGCTTTTATAACTTACCTACTAGCCTACCCAACATTTAC 2986

MH733478.2018 TATCTAAATTAAATTACATTATGCTTTTATAACTTACCTACTAGCCTACCCAACATTTAC 2989

MK007330.2018 TGTCTAAATTAAATTACATTATGCTTTTATAACTTACCTACTAGCCTGCCCAACATTTAC 2988

MK007344.2018 TGTCTAAATTAAATTACATTATGCTTTTATAACTTACCTACTAGCCTGCCCAACATTTAC 2982

* *** ******************************* ** ***** ************

AY142960.1976 ACGATCGTTTTATAATTAAGAAAAAACTAATGATGAAGATTAAAACCTTCATCATCCTTA 3060

KC242791.1977 ACGATCGTTTTATAATTAAGAAAAAACTAATGATGAAGATTAAAACCTTCATCATCCTTA 3060

AF499101.1976 ACGATCGTTTTATAATTAAGAAAAAACTAATGATGAAGATTAAAACCTTCATCATCCTTA 3060

KF113528.2003 ACGATCGTTTTATAATTAAGAAAAAACTAATGATGAAGATTAAAACCTTCATCATCCTTA 3056

KC242800.2002 ACGATCGTTTTATAATTAAGAAAAAACTAATGATGAAGATTAAAACCTTCATCATCCTTA 3060

KY471090.2001 ACGATCGTTTTATAATTAAGAAAAAACTAATGATGAAGATTAAAACCTTCATCATCCTTA 3037

KY471092.2001 ACGATCGTTTTATAATTAAGAAAAAACTAATGATGAAGATTAAAACCTTCATCATCCTTA 3037

MH481611.2017 ATGATCGTTTTATAATTAAGAAAAAACGAATGATGAAGATTAAAACCTTCATCATCCTTA 3026

MH613311.2017 ATGATCGTTTTATAATTAAGAAAAAACGAATGATGAAGATTAAAACCTTCATCATCCTTA 3005

KC242792.1994 ACGATCATTTTATAATTAAGAAAAAACTAATGATGAAGATTAAAACCTTCATCATCCTTA 3060

KC242793.1996 ACGATCATTTTATAATTAAGAAAAAACTAATGATGAAGATTAAAACCTTCATCATCCTTA 3060

KC242794.1996 ACGATCATTTTATAATTAAGAAAAAACTAATGATGAAGATTAAAACCTTTATCATCCTTA 3060

MH121164.1995 ACGATCATTTTATAATTAAGAAAAAACTAATGATGAAGATTAAAACCTTCATCATCCTTA 3060

AY354458.1995 ACGATCATTTTATAATTAAGAAAAAACTAATGATGAAGATTAAAACCTTCATCATCCTTA 3060

KT762962.1995 ACGATCATTTTATAATTAAGAAAAAACTAATGATGAAGATTAAAACCTTCATCATCCTTA 3060

HQ613402.2008 ACGATCGTTTTATAATTAAGAAAAAACTAATGATGAAGATTAAAACCTTCATCATCCTTA 2979

KC242789.2007 ACGATCGTTTTATAATTAAGAAAAAACTAATGATGAAGATTAAAACCTTCATCATCCTTA 3060

HQ613403.2007 ACGATCGTTTTATAATTAAGAAAAAACTAATGATGAAGATTAAAACCTTCATCATCCTTA 3012

KC242785.2007 ACGATCGTTTTATAATTAAGAAAAAACTAATGATGAAGATTAAAACCTTCATCATCCTTA 3060

KC242790.2007 ACGATCGTTTTATAATTAAGAAAAAACTAATGATGAAGATTAAAACCTTCATCATCCTTA 3060

KU143789.2014 ACGATCGTTTTATAATTAAGAAAAAACTAATGATGAAGATTAAAACCTTCATCATCCTTA 3060

KR817168.2014 ACGATCGTTTTATAATTAAGAAAAAACTAATGATGAAGATTAAAACCTTCATCATCCTTA 3034

KY426696.2015 ACGATCGTTTTATAATTAAGAAAAAACTAATGATGAAGATTAAAACCTTCATCATCCTTA 3060

KR105271.2014 ACGATCGTTTTATAATTAAGAAAAAACTAATGATGAAGATTAAAACCTTCATCATCCTTA 3027

KY007522.2016 ACGATCGTTTTATAATTAAGAAAAAACTAATGATGAAGATTAAAACCTTCATCATCCTTA 3040

KM034555.2014 ACGATCGTTTTATAATTAAGAAAAAACTAATGATGAAGATTAAAACCTTCATCATCCTTA 3052

MH470381.2015 ACGATCGTTTTATAATTAAGAAAAAACTAATGATGAAGATTAAAACCTTCATCATCCTTA 3024

MH470382.2015 ACGATCGCTTCATAATTAAGAAAAAACTAATGATGAAGATTAAAACCTTCATCATCCTTA 3024

MF102255.2014 ACGATCGTTTTATAATTAAGAAAAAACTAATGATGAAGATTAAAACCTTCATCATCCTTA 3037

KJ660348.2014 ACGATCGTTTTATAATTAAGAAAAAACTAATGATGAAGATTAAAACCTTCATCATCCTTA 3060

KU143818.2014 ACGATCGTTTTATAATTAAGAAAAAACTAATGATGAAGATTAAAACCTTCATCATCCTTA 3060

KT725333.2014 ACGATCATTTTATAATTAAGAAAAAACTAATGATGAAGATTAAAACCTTCATCATCCTTA 3025

KR819004.2014 ACGATCATTTTATAATTAAGAAAAAACTAATGATGAAGATTAAAACCTTCATCATCCTTA 3007

KP271020.2014 ACGATCATTTTATAATTAAGAAAAAACTAATGATGAAGATTAAAACCTTCATCATCCTTA 3007

KM519951.2014 ACGATCATTTTATAATTAAGAAAAAACTAATGATGAAGATTAAAACCTTCATCATCCTTA 3057

MH733488.2018 ACGATCATTTTATAATTAAGAAAAAACTAATGATGAAGATTAAAACCTTCATCATCCTTA 3051

MH733491.2018 ACGATCATTTTATAATTAAGAAAAAACTAATGATGAAGATTAAAACCTTCATCATCCTTA 3046

MH733478.2018 ACGATCATTTTATAATTAAGAAAAAACTAATGATGAAGATTAAAACCTTCATCATCCTTA 3049

MK007330.2018 ACGATCGTTTTATAATTAAGAAAAAACTAACGATGAAGATTAAAACCTTCATCATCCTTA 3048

MK007344.2018 ACGATCGTTTTATAATTAAGAAAAAACTAACGATGAAGATTAAAACCTTCATCATCCTTA 3042

* **** ** **************** ** ****************** **********

AY142960.1976 CGTCAATTGAATTCTCTAGCACTCGAAGCTTATTGTCTTCAATGTAAAAGAAAAGCTGGT 3120

KC242791.1977 CGTCAATTGAATTCTCTAGCACTCGAAGCTTATTGTCTTCAATGTAAAAGAAAAGCTGGT 3120

AF499101.1976 CGTCAATTGAATTCTCTAGCACTCGAAGCTTATTGTCTTCAATGTAAAAGAAAAGCTGGT 3120

KF113528.2003 CGTCAATTGAATTCTCTAGCACTCGAAGCTTATTGTCTTCAATGTAAAAGAAAAGCTGGT 3116

KC242800.2002 CGTCAATTGAATTCTCTAGCACTCGAAGCTTATTGTCTTCAATGTAAAAGAAAAGCTGGT 3120

KY471090.2001 CGTCAATTGAATTCTCTAGCACTCGAAGCTTATTGTCTTCAATGTAAAAGAAAAGCTGGT 3097

KY471092.2001 CGTCAATTGAATTCTCTAGCACTCGAAGCTTATTGTCTTCAATGTAAAAGAAAAGCTGGT 3097

MH481611.2017 CGTCAATTGAATTCTCTAGCATTCGAAGCTTATTGTCTTCAATGTAAAAGAAAAGCTGGT 3086

MH613311.2017 CGTCAATTGAATTCTCTAGCATTCGAAGCTTATTGTCTTCAATGTAAAAGAAAAGCTGGT 3065

KC242792.1994 CGTCAATTGAATTCTCTAGCACTCGAAGCTTATTGTCTTCAATGTAAAAGAAAAGCTGGT 3120

KC242793.1996 CGTCAATTGAATTCTCTAGCACTCGAAGCTTATTGTCTTCAATGTAAAAGAAAAGCTGGT 3120

KC242794.1996 CGTCAATTGAATTCTCTAGCACTCGAAGCTTATTGTCTTCAATGTAAAAGAAAAGCTGGT 3120

MH121164.1995 CGTCAATTGAATTCTCTAGCACTCGAAGCTTATTGTCCTCAATGTAAAAGAAAAGCTGGT 3120

AY354458.1995 CGTCAATTGAATTCTCTAGCACTCGAAGCTTATTGTCCTCAATGTAAAAGAAAAGCTGGT 3120

KT762962.1995 CGTCAATTGAATTCTCTAGCACTCGAAGCTTATTGTCCTCAATGTAAAAGAAAAGCTGGT 3120

HQ613402.2008 CGTCAATTGAATTCTCTAGCACTCGAAGCTTATTGTCTTCAATGTAAAAGAAAAGCTAGT 3039

KC242789.2007 CGTCAATTGAATTCTCTAGCACTCGAAGCTTATTGTCTTCAATGTAAAAGAAAAGCTAGT 3120

HQ613403.2007 CGTCAATTGAATTCTCTAGCACTCGAAGCTTATTGTCTTCAATGTAAAAGAAAAGCTAGT 3072

KC242785.2007 CGTCAATTGAATTCTCTAGCACTCGAAGCTTATTGTCTTCAATGTAAAAGAAAAGCTAGT 3120

KC242790.2007 CGTCAATTGAATTCTCTAGCACTCGAAGCTTATTGTCTTCAATGTAAAAGAAAAGCTAGT 3120

KU143789.2014 CGTCAATTGAATTCTCTACCACTAGAAGCTTATTGTCTTCAATGTAAAAGAAAAGCTGGC 3120

KR817168.2014 CGTCAATTGAATTCTCTAGCACTAGAAGCTTATTGTCTTCAATGTAAAAGAAAAGCTGGC 3094

KY426696.2015 CGTCAATTGAATTCTCTAGCACTAGAAGCTTATTGTCTTCAATGTAAAAGAAAAGCTGGC 3120

KR105271.2014 CGTCAATTGAATTCTCTAGCACTAGAAGCTTATTGTCTTCAATGTAAAAGAAAAGCTGGC 3087

KY007522.2016 CGTCAATTGAATTCTCTAGCACTAGAAGCTTATTGTCTTCAATGTAAAAGAAAAGCTGGC 3100

KM034555.2014 CGTCAATTGAATTCTCTAGCACTAGAAGCTTATTGTCTTCAATGTAAAAGAAAAGCTGGC 3112

MH470381.2015 CGTCAATTGAATTCTCTAGCACTAGAAGCTTATTGTCTTCAATGTAAAAGAAAAGCTGGC 3084

MH470382.2015 CGTCAATTGAATTCTCTAGCACTAGAAGCTTATTGTCTTCAATGTAAAAGAAAAGCTGGC 3084

MF102255.2014 CGTCAATTGAATTCTCTAGCACTAGAAGCTTATTGTCTTCAATGTAAAAGAAAAGCTGGC 3097

KJ660348.2014 CGTCAATTGAATTCTCTAGCACTAGAAGCTTATTGTCTTCAATGTAAAAGAAAAGCTGGC 3120

KU143818.2014 CGTCAATTGAATTCTCTAGCACTAGAAGCTTATTGTCTTCAATGTAAAAGAAAAGCTGGC 3120

KT725333.2014 CGTCAATTGAATTCTCTAGCACTAGAAGCTTATTGTCTTCAATGTAAAAGAAAAGCTGGC 3085

KR819004.2014 CGTCAATTGAATTCTCTAGCACTCGAAGCTTATTGTCTTCAATGTAAAAGAAAAGCTGGT 3067

KP271020.2014 CGTCAATTGAATTCTCTAGCACTCGAAGCTTATTGTCTTCAATGTAAAAGAAAAGCTGGT 3067

KM519951.2014 CGTCAATTGAATTCTCTAGCACTCGAAGCTTATTGTCTTCAATGTAAAAGAAAAGCTGGT 3117

MH733488.2018 CGTCAATTGAATTCTCTAGCACTCGAAGCTTATTGTCTTCAATGTAAAAGAAAAGCTGGT 3111

MH733491.2018 CGTCAATTGAATTCTCTAGCACTCGAAGCTTATTGTCTTCAATGTAAAAGAAAAGCTGGT 3106

MH733478.2018 CGTCAATTGAATTCTCTAGCACTCGAAGCTTATTGTCTTCAATGTAAAAGAAAAGCTGGT 3109

MK007330.2018 CGTCAATTGAATTCTCTAGCACTCGAAGCTTATTGTCTTCAATGTAAAAGAAAAGCTGGT 3108

MK007344.2018 CGTCAATTGAATTCTCTAGCACTCGAAGCTTATTGTCTTCAATGTAAAAGAAAAGCTGGT 3102

****************** ** * ************* ******************* *

AY142960.1976 CTAACAAGATGACAACTAGAACAAAGGGCAGGGGCCATACTGCGGCCACGACTCAAAACG 3180 start VP35

KC242791.1977 CTAACAAGATGACAACTAGAACAAAGGGCAGGGGCCATACTGCGGCCACGACTCAAAACG 3180

AF499101.1976 CTAACAAGATGACAACTAGAACAAAGGGCAGGGGCCATACTGTGGCCACGACTCAAAACG 3180

KF113528.2003 CCAACAAGATGACAACTAGAACAAAGGGCAGGGGCCATACTGTGGCCACGACTCAAAACG 3176

KC242800.2002 CCAACAAGATGACAACTAGAACAAAGGGCAGGGGCCATACTGTGGCCACGACTCAAAACG 3180

KY471090.2001 CCAACAAGATGACAACTAGAACAAAGGGCAGGGGCCATACTGTGGCCACGACTCAAAACG 3157

KY471092.2001 CCAACAAGATGACAACTAGAACAAAGGGCAGGGGCCATACTGTGGCCACGACTCAAAACG 3157

MH481611.2017 CTAACAAGATGACAACTAGAACAAAGGGCAGGGGCCATACTGCGGCCACGACTCAAAACG 3146

MH613311.2017 CTAACAAGATGACAACTAGAACAAAGGGCAGGGGCCATACTGCGGCCACGACTCAAAACG 3125

KC242792.1994 CAAACAAGATGACAACCAGAACAAAGAGCAGGGGCCACACTGCGGCCACGACTCAAAACG 3180

KC242793.1996 CTAACAAGATGACAACCAGAACAAAGAGCAGGGGCCACACTGCGGCCACGACTCAAAACG 3180

KC242794.1996 CTAACAAGATGACAACCAGAACAAAGAGCAGGGGCCACACTGCGGCCACGACTCAAAACG 3180

MH121164.1995 CTAACAAGATGACAACCAGAACAAAGGGCAGGGGCCACACTGCGGCCACGACTCAAAACG 3180

AY354458.1995 CTAACAAGATGACAACCAGAACAAAGGGCAGGGGCCACACTGCGGCCACGACTCAAAACG 3180

KT762962.1995 CTAACAAGATGACAACCAGAACAAAGGGCAGGGGCCACACTGCGGCCACGACTCAAAACG 3180

HQ613402.2008 CTAACAAGATGACAACTAGAACAAAGGGCAGGGGCCATACTGTGGCCACGACTCTAAACG 3099

KC242789.2007 CTAACAAGATGACAACTAGAACAAAGGGCAGGGGCCATACTGTGGCCACGACTCTAAACG 3180

HQ613403.2007 CTAACAAGATGACAACTAGAACAAAGGGCAGGGGCCATACTGTGGCCACGACTCTAAACG 3132

KC242785.2007 CTAACAAGATGACAACTAGAACAAAGGGCAGGGGCCATACTGTGGCCACGACTCTAAACG 3180

KC242790.2007 CTAACAAGATGACAACTAGAACAAAGGGCAGGGGCCATACTGTGGCCACGACTCTAAACG 3180

KU143789.2014 CTAACAAGATGACAACTAGAACAAAGGGCAGGGGCCATACTGTGGCCACGACTCAAAACG 3180

KR817168.2014 CTAACAAGATGACAACTAGAACAAAGGGCAGGGGCCATACTGTGGCCACGACTCAAAACG 3154

KY426696.2015 CTAACAAGATGACAACTAGAACAAAGGGCAGGGGCCATACTGTGGCCACGACTCAAAACG 3180

KR105271.2014 CTAACAAGATGACAACTAGAACAAAGGGCAGGGGCCATACTGTGGCCACGACTCAAAACG 3147

KY007522.2016 CTAACAAGATGACAACTAGAACAAAGGGCAGGGGCCATACTGTGGCCACGACTCAAAACG 3160

KM034555.2014 CTAACAAGATGACAACTAGAACAAAGGGCAGGGGCCATACTGTGGCCACGACTCAAAACG 3172

MH470381.2015 CTAACAAGATGACAACTAGAACAAAGGGCAGGGGCCATACTGTGGCCACGACTCAAAACG 3144

MH470382.2015 CTAACAAGATGACAACTAGAACAAAGGGCAGGGGCCATACTGTGGCCACGACTCAAAACG 3144

MF102255.2014 CTAACAAGATGACAACTAGAACAAAGGGCAGGGGCCATACTGTGGCCACGACTCAAAACG 3157

KJ660348.2014 CTAACAAGATGACAACTAGAACAAAGGGCAGGGGCCATACTGTGGCCACGACTCAAAACG 3180

KU143818.2014 CTAACAAGATGACAACTAGAACAAAGGGCAGGGGCCATACTGTGGCCACGACTCAAAACG 3180

KT725333.2014 CTAACAAGATGACAACTAGAACAAAGGGCAGGGGCCATACTGTGGCCACGACTCAAAACG 3145

KR819004.2014 CTAACAAGATGACAACCAGAACAAAGGGCAGGGGCCACACTGCGGCCACGACTCAAAACG 3127

KP271020.2014 CTAACAAGATGACAACCAGAACAAAGGGCAGGGGCCACACTGCGGCCACGACTCAAAACG 3127

KM519951.2014 CTAACAAGATGACAACCAGAACAAAGGGCAGGGGCCACACTGCGGCCACGACTCAAAACG 3177

MH733488.2018 CTAACAAGATGACAACCAGAACAAAGGGCAGGGGCCACACTGCGGCCACGACTCAAAACG 3171

MH733491.2018 CTAACAAGATGACAACCAGAACAAAGGGCAGGGGCCACACTGCGGCCACGACTCAAAACG 3166

MH733478.2018 CTAACAAGATGACAACCAGAACAAAGGGCAGGGGCCACACTGCGGCCACGACTCAAAACG 3169

MK007330.2018 CTAACAAGATGACAACTAGAACAAAGGGCAGGGGCCATACTGCGGTCACGACTCAAAACG 3168

MK007344.2018 CTAACAAGATGACAACTAGAACAAAGGGCAGGGGCCATACTGCGGTCACGACTCAAAACG 3162

* ************** ********* ********** **** ** ******** *****

AY142960.1976 ACAGAATGCCAGGCCCTGAGCTTTCGGGCTGGATCTCTGAGCAGCTAATGACCGGAAGAA 3240

KC242791.1977 ACAGAATGCCAGGCCCTGAGCTTTCGGGCTGGATCTCTGAGCAGCTAATGACCGGAAGAA 3240

AF499101.1976 ACAGAATGCCAGGCCCTGAGCTTTCGGGCTGGATCTCTGAGCAGCTAATGACCGGAAGAA 3240

KF113528.2003 ACAGAATGCCAGGCCCTGAGCTTTCGGGCTGGATCTCCGAGCAGCTAATGACCGGAAGAA 3236

KC242800.2002 ACAGAATGCCAGGCCCTGAGCTTTCGGGCTGGATCTCCGAGCAGCTAATGACCGGAAGAA 3240

KY471090.2001 ACAGAATGCCAGGCCCTGAGCTTTCGGGCTGGATCTCCGAGCAGCTAATGACCGGAAGAA 3217

KY471092.2001 ACAGAATGCCAGGCCCTGAGCTTTCGGGCTGGATCTCCGAGCAGCTAATGACCGGAAGAA 3217

MH481611.2017 ACAGAATGCCAGGCCCTGAGCTTTCGGGCTGGATCTCTGAGCAGCTAATGACCGGAAGAA 3206

MH613311.2017 ACAGAATGCCAGGCCCTGAGCTTTCGGGCTGGATCTCTGAGCAGCTAATGACCGGAAGAA 3185

KC242792.1994 ACAGAATGCCAGGCCCTGAGCTTTCGGGCTGGATCTCTGAGCAGCTAATGACCGGAAGAA 3240

KC242793.1996 ACAGAATGCCAGGCCCTGAGCTTTCGGGCTGGATCTCTGAGCAGCTAATGACCGGAAGAA 3240

KC242794.1996 ACAGAATGCCAGGCCCTGAGCTTTCGGGCTGGATCTCTGAGCAGCTAATGACCGGAAGAA 3240

MH121164.1995 ACAGAATGCCAGGCCCTGAGCTTTCGGGCTGGATCTCTGAGCAGCTAATGACCGGAAGAA 3240

AY354458.1995 ACAGAATGCCAGGCCCTGAGCTTTCGGGCTGGATCTCTGAGCAGCTAATGACCGGAAGAA 3240

KT762962.1995 ACAGAATGCCAGGCCCTGAGCTTTCGGGCTGGATCTCTGAGCAGCTAATGACCGGAAGAA 3240

HQ613402.2008 ACAGAATGCCAGGCCCTGAGCTTTCGGGCTGGATCTCTGAGCAGCTAATGACCGGAAGAA 3159

KC242789.2007 ACAGAATGCCAGGCCCTGAGCTTTCGGGCTGGATCTCTGAGCAGCTAATGACCGGAAGAA 3240

HQ613403.2007 ACAGAATGCCAGGCCCTGAGCTTTCGGGCTGGATCTCTGAGCAGCTAATGACCGGAAGAA 3192

KC242785.2007 ACAGAATGCCAGGCCCTGAGCTTTCGGGCTGGATCTCTGAGCAGCTAATGACCGGAAGAA 3240

KC242790.2007 ACAGAATGCCAGGCCCTGAGCTTTCGGGCTGGATCTCTGAGCAGCTAATGACCGGAAGAA 3240

KU143789.2014 ACAGAATGCCAGGCCCTGAGCTTTCGGGCTGGATCTCTGAGCAGCTAATGACCGGAAGGA 3240

KR817168.2014 ACAGAATGCCAGGCCCTGAGCTTTCGGGCTGGATCTCTGAGCAGCTAATGACCGGAAGGA 3214

KY426696.2015 ACAGAATGCCAGGCCCTGAGCTTTCGGGCTGGATCTCTGAGCAGCTAATGACCGGAAGGA 3240

KR105271.2014 ACAGAATGCCAGGCCCTGAGCTTTCGGGCTGGATCTCTGAGCAGCTAATGACCGGAAGGA 3207

KY007522.2016 ACAGAATGCCAGGCCCTGAGCTTTCGGGCTGGATCTCTGAGCAGCTAATGACCGGAAGGA 3220

KM034555.2014 ACAGAATGCCAGGCCCTGAGCTTTCGGGCTGGATCTCTGAGCAGCTAATGACCGGAAGGA 3232

MH470381.2015 ACAGAATGCCAGGCCCTGAGCTTTCGGGCTGGATCTCTGAGCAGCTAATGACCGGAAGGA 3204

MH470382.2015 ACAGAATGCCAGGCCCTGAGCTTTCGGGCTGGATCTCTGAGCAGCTAATGACCGGAAGGA 3204

MF102255.2014 ACAGAATGCCAGGCCCTGAGCTTTCGGGCTGGATCTCTGAGCAGCTAATGACCGGAAGGA 3217

KJ660348.2014 ACAGAATGCCAGGCCCTGAGCTTTCGGGCTGGATCTCTGAGCAGCTAATGACCGGAAGGA 3240

KU143818.2014 ACAGAATGCCAGGCCCTGAGCTTTCGGGCTGGATCTCTGAGCAGCTAATGACCGGAAGGA 3240

KT725333.2014 ACAGAATGCCAGGCCCTGAGCTTTCGGGCTGGATCTCTGAGCAGCTAATGACCGGAAGGA 3205

KR819004.2014 ACAGAATGCCAGGCCCTGAGCTTTCGGGCTGGATCTCTGAGCAGCTAATGACCGGAAGAA 3187

KP271020.2014 ACAGAATGCCAGGCCCTGAGCTTTCGGGCTGGATCTCTGAGCAGCTAATGACCGGAAGAA 3187

KM519951.2014 ACAGAATGCCAGGCCCTGAGCTTTCGGGCTGGATCTCTGAGCAGCTAATGACCGGAAGAA 3237

MH733488.2018 ACAGAATGCCAGGCCCTGAGCTTTCGGGCTGGATCTCTGAGCAGCTAATGACCGGAAGAA 3231

MH733491.2018 ACAGAATGCCAGGCCCTGAGCTTTCGGGCTGGATCTCTGAGCAGCTAATGACCGGAAGAA 3226

MH733478.2018 ACAGAATGCCAGGCCCTGAGCTTTCGGGCTGGATCTCTGAGCAGCTAATGACCGGAAGAA 3229

MK007330.2018 ACAGAATGCCAGGCCCTGAGCTTTCGGGCTGGATCTCTGAGCAGCTAATGACCGGAAGAA 3228

MK007344.2018 ACAGAATGCCAGGCCCTGAGCTTTCGGGCTGGATCTCTGAGCAGCTAATGACCGGAAGAA 3222

************************************* ******************** *

AY142960.1976 TTCCTGTAAGCGACATCTTCTGTGATATTGAGAACAATCCAGGATTATGCTACGCATCCC 3300

KC242791.1977 TTCCTGTAAGCGACATCTTCTGTGATATTGAGAACAATCCAGGATTATGCTACGCATCCC 3300

AF499101.1976 TTCCTGTAAGCGACATCTTCTGTGATATTGAGAACAATCCAGGATTATGCTACGCATCCC 3300

KF113528.2003 TTCCTGTAAGCGACATCTTCTGTGATATTGAGAACAATCCAGGATTATGTTACGCATCCC 3296

KC242800.2002 TTCCTGTAAGCGACATCTTCTGTGATATTGAGAACAATCCAGGATTATGTTACGCATCCC 3300

KY471090.2001 TTCCTGTAAGCGACATCTTCTGTGATATTGAGAACAATCCAGGATTATGTTACGCATCCC 3277

KY471092.2001 TTCCTGTAAGCGACATCTTCTGTGATATTGAGAACAATCCAGGATTATGTTACGCATCCC 3277

MH481611.2017 TTCCTGTAAGCGAAATCTTCTGTGATATTGAGAACAATCCAGGATTATGCTACGCATCCC 3266

MH613311.2017 TTCCTGTAAGCGAAATCTTCTGTGATATTGAGAACAATCCAGGATTATGCTACGTATCCC 3245

KC242792.1994 TTCCTGTAAGCGACATCTTCTGTGATATTGAGAACAATCCAGGATTATGCTACGCATCCC 3300

KC242793.1996 TTCCTGTAAGCGACATCTTCTGTGATATTGAGAACAATCCAGGATTATGCTACGCATCCC 3300

KC242794.1996 TTCCTGTAAGCGACATCTTCTGTGATATTGAGAACAATCCAGGATTATGCTACGCATCCC 3300

MH121164.1995 TTCCTGTAAGCGACATCTTCTGTGATATTGAGAACAATCCAGGATTATGCTACGCATCCC 3300

AY354458.1995 TTCCTGTAAGCGACATCTTCTGTGATATTGAGAACAATCCAGGATTATGCTACGCATCCC 3300

KT762962.1995 TTCCTGTAAGCGACATCTTCTGTGATATTGAGAACAATCCAGGATTATGCTACGCATCCC 3300

HQ613402.2008 TTCCTGTAAGCGACATCTTCTGTGATATTGAGAACAATCCAGGATTATGCTACGCATCCC 3219

KC242789.2007 TTCCTGTAAGCGACATCTTCTGTGATATTGAGAACAATCCAGGATTATGCTACGCATCCC 3300

HQ613403.2007 TTCCTGTAAGCGACATCTTCTGTGATATTGAGAACAATCCAGGATTATGCTACGCATCCC 3252

KC242785.2007 TTCCTGTAAGCGACATCTTCTGTGATATTGAGAACAATCCAGGATTATGCTACGCATCCC 3300

KC242790.2007 TTCCTGTAAGCGACATCTTCTGTGATATTGAGAACAATCCAGGATTATGCTACGCATCCC 3300

KU143789.2014 TTCCTGTAAACGACATCTTCTGTGATATTGAGAACAATCCAGGATTATGCTACGCATCCC 3300

KR817168.2014 TTCCTGTAAACGACATCTTCTGTGATATTGAGAACAATCCAGGATTATGCTACGCATCCC 3274

KY426696.2015 TTCCTGTAAACGACATCTTCTGTGATATTGAGAACAATCCAGGATTATGCTACGCATCCC 3300

KR105271.2014 TTCCTGTAAACGACATCTTCTGTGATATTGAGAACAATCCAGGATTATGCTACGCATCCC 3267

KY007522.2016 TTCCTGTAAACGACATCTTCTGTGATATTGAGAACAATCCAGGATTATGCTACGCATCCC 3280

KM034555.2014 TTCCTGTAAACGACATCTTCTGTGATATTGAGAACAATCCAGGATTATGCTACGCATCCC 3292

MH470381.2015 TTCCTGTAAACGACATCTTCTGTGATATTGAGAACAATCCAGGATTATGCTACGCATCCC 3264

MH470382.2015 TTCCTGTAAACGACATCTTCTGTGATATTGAGAACAATCCAGGATTATGCTACGCATCCC 3264

MF102255.2014 TTCCTGTAAACGACATCTTCTGTGATATTGAGAACAATCCAGGATTATGCTACGCATCCC 3277

KJ660348.2014 TTCCTGTAAACGACATCTTCTGTGATATTGAGAACAATCCAGGATTATGCTACGCATCCC 3300

KU143818.2014 TTCCTGTAAACGACATCTTCTGTGATATTGAGAACAATCCAGGATTATGCTACGCATCCC 3300

KT725333.2014 TTCCTGTAAACGACATCTTCTGTGATATTGAGAACAATCCAGGATTATGCTACGCATCCC 3265

KR819004.2014 TTCCTATAAGCGACATCTTCTGTGATATTGAGAACAATCCAGGATTATGCTACGCATCCC 3247

KP271020.2014 TTCCTATAAGCGACATCTTCTGTGATATTGAGAACAATCCAGGATTATGCTACGCATCCC 3247

KM519951.2014 TTCCTATAAGCGACATCTTCTGTGATATTGAGAACAATCCAGGATTATGCTACGCATCCC 3297

MH733488.2018 TTCCTGTAAGCGACATCTTCTGTGATATTGAGAACAATCCAGGATTATGCTACGCATCCC 3291

MH733491.2018 TTCCTGTAAGCGACATCTTCTGTGATATTGAGAACAATCCAGGATTATGCTACGCATCCC 3286

MH733478.2018 TTCCTGTAAGCGACATCTTCTGTGATATTGAGAACAATCCAGGATTATGCTACGCATCCC 3289

MK007330.2018 TTCCTGTAAGCGACATCTTCTGTGATATTGAGAACAATCCAGGATTATGCCACGTATCCC 3288

MK007344.2018 TTCCTGTAAGCGACATCTTCTGTGATATTGAGAACAATCCAGGATTATGCCACGTATCCC 3282

***** *** *** *********************************** *** *****

AY142960.1976 AAATGCAACAAACGAAGCCAAACCCGAAGACGCGCAACAGTCAAACCCAAACGGACCCAA 3360

KC242791.1977 AAATGCAACAAACGAAGCCAAACCCGAAGACGCGCAACAGTCAAACCCAAACGGACCCAA 3360

AF499101.1976 AAATGCAACAAACGAAGCCAAACCCGAAGACGCGCAACAGTCAAACCCAAACGGACCCAA 3360

KF113528.2003 AAATGCAACAAACAAAGCCAAACCCGAAGATGCGCAACAGTCAAACCCAAACGGACCCAA 3356

KC242800.2002 AAATGCAACAAACAAAGCCAAACCCGAAGATGCGCAACAGTCAAACCCAAACGGACCCAA 3360

KY471090.2001 AAATGCAACAAACAAAGCCAAACCCGAAGATGCGCAACAGTCAAACCCAAACGGACCCAA 3337

KY471092.2001 AAATGCAACAAACAAAGCCAAACCCGAAGATGCGCAACAGTCAAACCCAAACGGACCCAA 3337

MH481611.2017 AAATGCAACAAACGAAGCCAAACCCGAAGACGCGCAACAGTCAAACCCAAACGGACCCAA 3326

MH613311.2017 AAATGCAACAAACGAAGCCAAACCCGAAGACGCGCAACAGTCAAACCCAAACGGACCCAA 3305

KC242792.1994 AAATGCAACAAACGAAGCCAAACCCGAAGACGCGCAACAGTCAAACCCAAACGGATCCAA 3360

KC242793.1996 AAATGCAACAAACGAAGCCAAACCCGAAGACGCGCAACAGTCAAACCCAAACGGATCCAA 3360

KC242794.1996 AAATGCAACAGACGAAGCCAAACCCGAAGACGCGCAACAGTCAAACCCAAACGGATCCAA 3360

MH121164.1995 AAATGCAACAAACCAAGCCAAACCCGAAGACGCGCAACAGTCAAACCCAAACGGACCCAA 3360

AY354458.1995 AAATGCAACAAACCAAGCCAAACCCGAAGACGCGCAACAGTCAAACCCAAACGGACCCAA 3360

KT762962.1995 AAATGCAACAAACCAAGCCAAACCCGAAGACGCGCAACAGTCAAACCCAAACGGACCCAA 3360

HQ613402.2008 AAATGCAACAAACGAAGCCAAACCCGAAAACGCGCAACAGTCAAACCCAAACGGACCCAA 3279

KC242789.2007 AAATGCAACAAACGAAGCCAAACCCGAAAACGCGCAACAGTCAAACCCAAACGGACCCAA 3360

HQ613403.2007 AAATGCAACAAACGAAGCCAAACCCGAAAACGCGCAACAGTCAAACCCAAACGGACCCAA 3312

KC242785.2007 AAATGCAACAAACGAAGCCAAACCCGAAAACGCGCAACAGTCAAACCCAAACGGACCCAA 3360

KC242790.2007 AAATGCAACAAACGAAGCCAAACCCGAAAACGCGCAACAGTCAAACCCAAACGGACCCAA 3360

KU143789.2014 AAATGCAACAAACGAAGCCAAACCCGAAGATGCGCAACAGTCAAACCCAAACGGACCCAA 3360

KR817168.2014 AAATGCAACAAACGAAGCCAAACCCGAAGATGCGCAACAGTCAAACCCAAACGGACCCAA 3334

KY426696.2015 AAATGCAACAAACGAAGCCAAACCCGAAGATGCGCAACAGTCAAACCCAAACGGACCCAA 3360

KR105271.2014 AAATGCAACAAACGAAGCCAAACCCGAAGATGCGCAACAGTCAAACCCAAACGGACCCAA 3327

KY007522.2016 AAATGCAACAAACGAAGCCAAACCCGAAGATGCGCAACAGTCAAACCCAAACGGACCCAA 3340

KM034555.2014 AAATGCAACAAACGAAGCCAAACCCGAAGATGCGCAACAGTCAAACCCAAACGGACCCAA 3352

MH470381.2015 AAATGCAACAAACGAAGCCAAACCCGAAGATGCGCAACAGTCAAACCCAAACGGACCCAA 3324

MH470382.2015 AAATGCAACAAACGAAGCCAAACCCGAAGATGCGCAACAGTCAAACCCAAACGGACCCAA 3324

MF102255.2014 AAATGCAACAAACGAAGCCAAACCCGAAGATGCGCAACAGTCAAACCCAAACGGACCCAA 3337

KJ660348.2014 AAATGCAACAAACGAAGCCAAACCCGAAGATGCGCAACAGTCAAACCCAAACGGACCCAA 3360

KU143818.2014 AAATGCAACAAACGAAGCCAAACCCGAAGATGCGCAACAGTCAAACCCAAACGGACCCAA 3360

KT725333.2014 AAATGCAACAAACGAAGCCAAACCCGAAGATGCGCAACAGTCAAACCCAAACGGACCCAA 3325

KR819004.2014 AAATGCAACAAACGAAGCCAAACCCGAAGACGCGCAACAGTCAAACCCAAACGGACCCAA 3307

KP271020.2014 AAATGCAACAAACGAAGCCAAACCCGAAGACGCGCAACAGTCAAACCCAAACGGACCCAA 3307

KM519951.2014 AAATGCAACAAACGAAGCCAAACCCGAAGACGCGCAACAGTCAAACCCAAACGGACCCAA 3357

MH733488.2018 AAATGCAACAAACGAAGCCAAACCCGAAGACGCGCAACAGTCAAACCCAAACGGACCCAA 3351

MH733491.2018 AAATGCAACAAACGAAGCCAAACCCGAAGACGCGCAACAGTCAAACCCAAACGGACCCAA 3346

MH733478.2018 AAATGCAACAAACGAAGCCAAACCCGAAGACGCGCAACAGTCAAACCCAAACGGACCCAA 3349

MK007330.2018 AAATGCAGCAAACGAAGCCAAACCCGAAGACGCGCAACAGTCAAACCCAAACGGACCCAA 3348

MK007344.2018 AAATGCAGCAAACGAAGCCAAACCCGAAGACGCGCAACAGTCAAACCCAAACGGACCCAA 3342

******* ** ** ************** * ************************ ****

AY142960.1976 TTTGCAATCATAGTTTTGAGGAGGTAGTACAAACATTGGCTTCATTGGCTACTGTTGTGC 3420

KC242791.1977 TTTGCAATCATAGTTTTGAGGAGGTAGTACAAACATTGGCTTCATTGGCTACTGTTGTGC 3420

AF499101.1976 TTTGCAATCATAGTTTTGAGGAGGTAGTACAAACATTGGCTTCATTGGCTACTGTTGTGC 3420

KF113528.2003 TTTGCAATCATAGTTTTGAGGAGGTAGTACAAACATTGGCTTCATTGGCTACTGTTGTGC 3416

KC242800.2002 TTTGCAATCATAGTTTTGAGGAGGTAGTACAAACATTGGCTTCATTGGCTACTGTTGTGC 3420

KY471090.2001 TTTGCAATCATAGTTTTGAGGAGGTAGTACAAACATTGGCTTCATTGGCTACTGTTGTGC 3397

KY471092.2001 TTTGCAATCATAGTTTTGAGGAGGTAGTACAAACATTGGCTTCATTGGCTACTGTTGTGC 3397

MH481611.2017 TTTGCAATCATAGTTTTGAGGAGGTAGTACAAACATTGGCTTCATTGGCTACTGTTGTGC 3386

MH613311.2017 TTTGCAATCATAGTTTTGAGGAGGTAGTACAAACATTGGCTTCATTGGCTACTGTTGTGC 3365

KC242792.1994 TTTGCAATCATAGTTTTGAGGAGGTAGTACAAACATTAGCTTCATTGGCTACTGTTGTGC 3420

KC242793.1996 TTTGCAATCATAGTTTTGAGGAGGTAGTACAAACATTAGCTTCATTGGCTACTGTTGTGC 3420

KC242794.1996 TTTGCAATCATAGTTTTGAGGAGGTAGTACAGACATTAGCTTCATTGGCTACTGTTGTGC 3420

MH121164.1995 TTTGCAATCATAGTTTTGAGGAGGTAGTACAAACATTAGCTTCATTGGCTACTGTTGTGC 3420

AY354458.1995 TTTGCAATCATAGTTTTGAGGAGGTAGTACAAACATTAGCTTCATTGGCTACTGTTGTGC 3420

KT762962.1995 TTTGCAATCATAGTTTTGAGGAGGTAGTACAAACATTAGCTTCATTGGCTACTGTTGTGC 3420

HQ613402.2008 TTTGCAATCATAGTTTTGAGGAGGTAGTACAAACATTGGCTTCATTGGCTACTGTTGTGC 3339

KC242789.2007 TTTGCAATCATAGTTTTGAGGAGGTAGTACAAACATTGGCTTCATTGGCTACTGTTGTGC 3420

HQ613403.2007 TTTGCAATCATAGTTTTGAGGAGGTAGTACAAACATTGGCTTCATTGGCTACTGTTGTGC 3372

KC242785.2007 TTTGCAATCATAGTTTTGAGGAGGTAGTACAAACATTGGCTTCATTGGCTACTGTTGTGC 3420

KC242790.2007 TTTGCAATCATAGTTTTGAGGAGGTAGTACAAACATTGGCTTCATTGGCTACTGTTGTGC 3420

KU143789.2014 TTTGCAATCATAGTTTTGAGGAGGTAGTACAAACATTGGCTTCATTGGCTACTGTTGTGC 3420

KR817168.2014 TTTGCAATCATAGTTTTGAGGAGGTAGTACAAACATTGGCTTCATTGGCTACTGTTGTGC 3394

KY426696.2015 TTTGCAATCATAGTTTTGAGGAGGTAGTACAAACATTGGCTTCATTGGCTACTGTTGTGC 3420

KR105271.2014 TTTGCAATCATAGTTTTGAGGAGGTAGTACAAACATTGGCTTCATTGGCTACTGTTGTGC 3387

KY007522.2016 TTTGCAATCATAGTTTTGAGGAGGTAGTACAAACATTGGCTTCATTGGCTACTGTTGTGC 3400

KM034555.2014 TTTGCAATCATAGTTTTGAGGAGGTAGTACAAACATTGGCTTCATTGGCTACTGTTGTGC 3412

MH470381.2015 TTTGCAATCATAGTTTTGAGGAGGTAGTACAAACATTGGCTTCATTGGCTACTGTTGTGC 3384

MH470382.2015 TTTGCAATCATAGTTTTGAGGAGGTAGTACAAACATTGGCTTCATTGGCTACTGTTGTGC 3384

MF102255.2014 TTTGCAATCATAGTTTTGAGGAGGTAGTACAAACATTGGCTTCATTGGCTACTGTTGTGC 3397

KJ660348.2014 TTTGCAATCATAGTTTTGAGGAGGTAGTACAAACATTGGCTTCATTGGCTACTGTTGTGC 3420

KU143818.2014 TTTGCAATCATAGTTTTGAGGAGGTAGTACAAACATTGGCTTCATTGGCTACTGTTGTGC 3420

KT725333.2014 TTTGCAATCATAGTTTTGAGGAGGTAGTACAAACATTGGCTTCATTGGCTACTGTTGTGC 3385

KR819004.2014 TTTGCAATCATAGTTTTGAGGAGGTAGTACAAACATTAGCTTCATTGGCTACTGTTGTGC 3367

KP271020.2014 TTTGCAATCATAGTTTTGAGGAGGTAGTACAAACATTAGCTTCATTGGCTACTGTTGTGC 3367

KM519951.2014 TTTGCAATCATAGTTTTGAGGAGGTAGTACAAACATTAGCTTCATTGGCTACTGTTGTGC 3417

MH733488.2018 TTTGCAATCATAGTTTTGAGGAGGTAGTACAAACATTAGCTTCATTGGCTACTGTTGTGC 3411

MH733491.2018 TTTGCAATCATAGTTTTGAGGAGGTAGTACAAACATTAGCTTCATTGGCTACTGTTGTGC 3406

MH733478.2018 TTTGCAATCATAGTTTTGAGGAGGTAGTACAAACATTAGCTTCATTGGCTACTGTTGTGC 3409

MK007330.2018 TTTGCAATCATAGTTTTGAGGAGGTAGTACAAACATTGGCTTCATTGGCTACTGTTGTGC 3408

MK007344.2018 TTTGCAATCATAGTTTTGAGGAGGTAGTACAAACATTGGCTTCATTGGCTACTGTTGTGC 3402

******************************* ***** **********************

AY142960.1976 AACAACAAACCATCGCATCAGAATCATTAGAACAACGCATTACGAGTCTTGAGAATGGTC 3480

KC242791.1977 AACAACAAACCATCGCATCAGAATCATTAGAACAACGCATTACGAGTCTTGAGAATGGTC 3480

AF499101.1976 AACAACAAACCATCGCATCAGAATCATTAGAACAACGCATTACGAGTCTTGAGAATGGTC 3480

KF113528.2003 AACAACAAACTATCGCATCAGAATCATTAGAACAACGTATTACGAGTCTTGAGAATGGTC 3476

KC242800.2002 AACAACAAACTATCGCATCAGAATCATTAGAACAACGTATTACGAGTCTTGAGAATGGTC 3480

KY471090.2001 AACAACAAACTATCGCATCAGAATCATTAGAACAACGTATTACGAGTCTTGAGAATGGTC 3457

KY471092.2001 AACAACAAACTATCGCATCAGAATCATTAGAACAACGTATTACGAGTCTTGAGAATGGTC 3457

MH481611.2017 AACAACAAACCATCGCATCAGAATCATTAGAACAACGCATTACGAGTCTTGAGAATGGTC 3446

MH613311.2017 AACAACAAACCATCGCATCAGAATCATTAGAACAACGCATTACGAGTCTTGAGAATGGTC 3425

KC242792.1994 AACAACAAACCATCGCATCAGAATCATTAGAACAACGCATTACGAGTCTTGAGAATGGTC 3480

KC242793.1996 AACAACAAACCATCGCATCAGAATCATTAGAACAACGCATTACGAGTCTTGAGAATGGTC 3480

KC242794.1996 AACAACAAACCATCGCATCAGAATCATTAGAACAACGCATTACGAGTCTTGAGAATGGTC 3480

MH121164.1995 AACAACAAACCATTGCATCAGAATCATTAGAACAACGCATTACGAGTCTTGAGAATGGTC 3480

AY354458.1995 AACAACAAACCATTGCATCAGAATCATTAGAACAACGCATTACGAGTCTTGAGAATGGTC 3480

KT762962.1995 AACAACAAACCATTGCATCAGAATCATTAGAACAACGCATTACGAGTCTTGAGAATGGTC 3480

HQ613402.2008 AACAACAAACCATCGCATCAGAATCATTAGAACAACGCATTACGAGTCTTGAGAATGGTC 3399

KC242789.2007 AACAACAAACCATCGCATCAGAATCATTAGAACAACGCATTACGAGTCTTGAGAATGGTC 3480

HQ613403.2007 AACAACAAACCATCGCATCAGAATCATTAGAACAACGCATTACGAGTCTTGAGAATGGTC 3432

KC242785.2007 AACAACAAACCATCGCATCAGAATCATTAGAACAACGCATTACGAGTCTTGAGAATGGTC 3480

KC242790.2007 AACAACAAACCATCGCATCAGAATCATTAGAACAACGCATTACGAGTCTTGAGAATGGTC 3480

KU143789.2014 AACAACAAACCATCGCATCAGAATCATTAGAACAACGCATTACGAGTCTTGAGAATGGTC 3480

KR817168.2014 AACAACAAACCATCGCATCAGAATCATTAGAACAACGCATTACGAGTCTTGAGAATGGTC 3454

KY426696.2015 AACAACAAACCATCGCATCAGAATCATTAGAACAACGCATTACGAGTCTTGAGAATGGTC 3480

KR105271.2014 AACAACAAACCATCGCATCAGAATCATTAGAACAACGCATTACGAGTCTTGAGAATGGTC 3447

KY007522.2016 AACAACAAACCATCGCATCAGAATCATTAGAACAACGCATTACGAGTCTTGAGAATGGTC 3460

KM034555.2014 AACAACAAACCATCGCATCAGAATCATTAGAACAACGCATTACGAGTCTTGAGAATGGTC 3472

MH470381.2015 AACAACAAACCATCGCATCAGAATCATTAGAACAACGCATTACGAGTCTTGAGAATGGTC 3444

MH470382.2015 AACAACAAACCATCGCATCAGAATCATTAGAACAACGCATTACGAGTCTTGAGAATGGTC 3444

MF102255.2014 AACAACAAACCATCGCATCAGAATCATTAGAACAACGCATTACGAGTCTTGAGAATGGTC 3457

KJ660348.2014 AACAACAAACCATCGCATCAGAATCATTAGAACAACGCATTACGAGTCTTGAGAATGGTC 3480

KU143818.2014 AACAACAAACCATCGCATCAGAATCATTAGAACAACGCATTACGAGTCTTGAGAATGGTC 3480

KT725333.2014 AACAACAAACCATCGCATCAGAATCATTAGAACAACGCATTACGAGTCTTGAGAATGGTC 3445

KR819004.2014 AACAACAAACCATCGCATCAGAATCATTAGAACAACGCATTACGAGTCTTGAGAATGGTC 3427

KP271020.2014 AACAACAAACCATCGCATCAGAATCATTAGAACAACGCATTACGAGTCTTGAGAATGGTC 3427

KM519951.2014 AACAACAAACCATCGCATCAGAATCATTAGAACAACGCATTACGAGTCTTGAGAATGGTC 3477

MH733488.2018 AACAACAAACCATCGCATCAGAATCATTAGAACAACGCATTACGAGTCTTGAGAATGGTC 3471

MH733491.2018 AACAACAAACCATCGCATCAGAATCATTAGAACAACGCATTACGAGTCTTGAGAATGGTC 3466

MH733478.2018 AACAACAAACCATCGCATCAGAATCATTAGAACAACGCATTACGAGTCTTGAGAATGGTC 3469

MK007330.2018 AACAACAAACCATCGCATCAGAATCATTAGAGCAACGCATTACGAGTCTTGAGAATGGTC 3468

MK007344.2018 AACAACAAACCATCGCATCAGAATCATTAGAGCAACGCATTACGAGTCTTGAGAATGGTC 3462

********** ** ***************** ***** **********************

AY142960.1976 TAAAGCCAGTTTATGATATGGCAAAAACAATCTCCTCATTGAACAGGGTTTGTGCTGAGA 3540

KC242791.1977 TAAAGCCAGTTTATGATATGGCAAAAACAATCTCCTCATTGAACAGGGTTTGTGCTGAGA 3540

AF499101.1976 TAAAGCCAGTTTATGATATGGCAAAAACAATCTCCTCATTGAACAGGGTTTGTGCTGAGA 3540

KF113528.2003 TAAAGCCAGTTTATGATATGGCAAAAACAATCTCCTCATTGAACAGGGTTTGTGCTGAGA 3536

KC242800.2002 TAAAGCCAGTTTATGATATGGCAAAAACAATCTCCTCATTGAACAGGGTTTGTGCTGAGA 3540

KY471090.2001 TAAAGCCAGTTTATGATATGGCAAAAACAATCTCCTCATTGAACAGGGTTTGTGCTGAGA 3517

KY471092.2001 TAAAGCCAGTTTATGATATGGCAAAAACAATCTCCTCATTGAACAGGGTTTGTGCTGAGA 3517

MH481611.2017 TAAAGCCAGTTTATGATATGGCAAAAACAATCTCCTCATTGAACAGGGTTTGTGCTGAGA 3506

MH613311.2017 TAAAGCCAGTTTATGATATGGCAAAAACAATCTCCTCATTGAACAGGGTTTGTGCTGAGA 3485

KC242792.1994 TAAAGCCAGTTTATGATATGGCAAAAACAATCTCCTCATTGAACAGGGTTTGTGCTGAGA 3540

KC242793.1996 TAAAGCCAGTTTATGATATGGCAAAAACAATCTCCTCATTGAACAGGGTTTGTGCTGAGA 3540

KC242794.1996 TAAAGCCAGTTTATGATATGGCAAAAACAATCTCCTCATTGAACAGGGTTTGCGCTGAGA 3540

MH121164.1995 TAAAGCCAGTTTATGATATGGCTAAAACAATCTCCTCATTGAACAGGGTTTGTGCTGAGA 3540

AY354458.1995 TAAAGCCAGTTTATGATATGGCTAAAACAATCTCCTCATTGAACAGGGTTTGTGCTGAGA 3540

KT762962.1995 TAAAGCCAGTTTATGATATGGCTAAAACAATCTCCTCATTGAACAGGGTTTGTGCTGAGA 3540

HQ613402.2008 TAAAGCCAGTTTATGATATGGCAAAAACAATCTCCTCATTGAACAGGGTTTGTGCTGAGA 3459

KC242789.2007 TAAAGCCAGTTTATGATATGGCAAAAACAATCTCCTCATTGAACAGGGTTTGTGCTGAGA 3540

HQ613403.2007 TAAAGCCAGTTTATGATATGGCAAAAACAATCTCCTCATTGAACAGGGTTTGTGCTGAGA 3492

KC242785.2007 TAAAGCCAGTTTATGATATGGCAAAAACAATCTCCTCATTGAACAGGGTTTGTGCTGAGA 3540

KC242790.2007 TAAAGCCAGTTTATGATATGGCAAAAACAATCTCCTCATTGAACAGGGTTTGTGCTGAGA 3540

KU143789.2014 TAAAGCCAGTTTATGATATGGCAAAAACAATCTCCTCATTGAACAGGGTTTGTGCTGAGA 3540

KR817168.2014 TAAAGCCAGTTTATGATATGGCAAAAACAATCTCCTCATTGAACAGGGTTTGTGCTGAGA 3514

KY426696.2015 TAAAGCCAGTTTATGATATGGCAAAAACAATCTCCTCATTGAACAGGGTTTGTGCTGAGA 3540

KR105271.2014 TAAAGCCAGTTTATGATATGGCAAAAACAATCTCCTCATTGAACAGGGTTTGTGCTGAGA 3507

KY007522.2016 TAAAGCCAGTTTATGATATGGCAAAAACAATCTCCTCATTGAACAGGGTTTGTGCTGAGA 3520

KM034555.2014 TAAAGCCAGTTTATGATATGGCAAAAACAATCTCCTCATTGAACAGGGTTTGTGCTGAGA 3532

MH470381.2015 TAAAGCCAGTTTATGATATGGCAAAAACAATCTCCTCATTGAACAGGGTTTGTGCTGAGA 3504

MH470382.2015 TAAAGCCAGTTTATGATATGGCAAAAACAATCTCCTCATTGAACAGGGTTTGTGCTGAGA 3504

MF102255.2014 TAAAGCCAGTTTATGATATGGCAAAAACAATCTCCTCATTGAACAGGGTTTGTGCTGAGA 3517

KJ660348.2014 TAAAGCCAGTTTATGATATGGCAAAAACAATCTCCTCATTGAACAGGGTTTGTGCTGAGA 3540

KU143818.2014 TAAAGCCAGTTTATGATATGGCAAAAACAATCTCCTCATTGAACAGGGTTTGTGCTGAGA 3540

KT725333.2014 TAAAGCCAGTTTATGATATGGCAAAAACAATCTCCTCATTGAACAGGGTTTGTGCTGAGA 3505

KR819004.2014 TAAAGCCAGTTTATGATATGGCAAAAACAATCTCCTCATTGAACAGGGTTTGTGCTGAGA 3487

KP271020.2014 TAAAGCCAGTTTATGATATGGCAAAAACAATCTCCTCATTGAACAGGGTTTGTGCTGAGA 3487

KM519951.2014 TAAAGCCAGTTTATGATATGGCAAAAACAATCTCCTCATTGAACAGGGTTTGTGCTGAGA 3537

MH733488.2018 TAAAGCCAGTTTATGATATGGCAAAAACAATCTCCTCATTGAACAGGGTTTGTGCTGAGA 3531

MH733491.2018 TAAAGCCAGTTTATGATATGGCAAAAACAATCTCCTCATTGAACAGGGTTTGTGCTGAGA 3526

MH733478.2018 TAAAGCCAGTTTATGATATGGCAAAAACAATCTCCTCATTGAACAGGGTTTGTGCTGAGA 3529

MK007330.2018 TAAAGCCAGTTTATGATATGGCAAAAACAATCTCCTCATTGAACAGGGTTTGTGCTGAGA 3528

MK007344.2018 TAAAGCCAGTTTATGATATGGCAAAAACAATCTCCTCATTGAACAGGGTTTGTGCTGAGA 3522

********************** ***************************** *******

AY142960.1976 TGGTTGCAAAATATGATCTTCTGGTGATGACAACCGGTCGGGCAACAGCAACCGCTGCGG 3600

KC242791.1977 TGGTTGCAAAATATGATCTTCTGGTGATGACAACCGGTCGGGCAACAGCAACCGCTGCGG 3600

AF499101.1976 TGGTTGCAAAATATGATCTTCTGGTGATGACAACCGGTCGGGCAACAGCAACCGCTGCGG 3600

KF113528.2003 TGGTTGCAAAATATGATCTTCTGGTGATGACAACCGGTCGGGCAACAGCAACCGCTGCGG 3596

KC242800.2002 TGGTTGCAAAATATGATCTTCTGGTGATGACAACCGGTCGGGCAACAGCAACCACTGCGG 3600

KY471090.2001 TGGTTGCAAAATATGATCTTCTGGTGATGACAACCGGTCGGGCAACAGCAACCGCTGCGG 3577

KY471092.2001 TGGTTGCAAAATATGATCTTCTGGTGATGACAACCGGTCGGGCAACAGCAACCGCTGCGG 3577

MH481611.2017 TGGTTGCAAAATATGATCTTCTGGTGATGACAACCGGTCGGGCAACAGCAACCGCTGCAG 3566

MH613311.2017 TGGTTGCAAAATATGATCTTCTGGTGATGACAACCGGTCGGGCAACAGCAACCGCTGCAG 3545

KC242792.1994 TGGTTGCAAAATATGATCTTCTGGTGATGACAACCGGTCGGGCAACAGCAACCGCTGCGG 3600

KC242793.1996 TGGTTGCAAAATATGATCTTCTGGTGATGACAACCGGTCGGGCAACAGCAACCGCTGCGG 3600

KC242794.1996 TGGTTGCAAAATATGATCTTCTGGTGATGACAACCGGTCGGGCAACAGCAACCGCTGCGG 3600

MH121164.1995 TGGTTGCAAAATATGATCTTCTGGTGATGACAACCGGTCGGGCAACAGCAACCGCTGCGG 3600

AY354458.1995 TGGTTGCAAAATATGATCTTCTGGTGATGACAACCGGTCGGGCAACAGCAACCGCTGCGG 3600

KT762962.1995 TGGTTGCAAAATATGATCTTCTGGTGATGACAACCGGTCGGGCAACAGCAACCGCTGCGG 3600

HQ613402.2008 TGGTTGCAAAATATGATCTTCTGGTGATGACAACCGGACGGGCAACAGCAACCGCTGCGG 3519

KC242789.2007 TGGTTGCAAAATATGATCTTCTGGTGATGACAACCGGACGGGCAACAGCAACCGCTGCGG 3600

HQ613403.2007 TGGTTGCAAAATATGATCTTCTGGTGATGACAACCGGACGGGCAACAGCAACCGCTGCGG 3552

KC242785.2007 TGGTTGCAAAATATGATCTTCTGGTGATGACAACCGGACGGGCAACAGCAACCGCTGCGG 3600

KC242790.2007 TGGTTGCAAAATATGATCTTCTGGTGATGACAACCGGACGGGCAACAGCAACCGCTGCGG 3600

KU143789.2014 TGGTTGCAAAATATGATCTTCTGGTGATGACAACCGGTCGGGCAACAGCAACCGCTGCAG 3600

KR817168.2014 TGGTTGCAAAATATGATCTTCTGGTGATGACAACCGGTCGGGCAACAGCAACCGCTGCGG 3574

KY426696.2015 TGGTTGCAAAATATGATCTTCTGGTGATGACAACCGGTCGGGCAACAGCAACCGCTGCGG 3600

KR105271.2014 TGGTTGCAAAATATGATCTTCTGGTGATGACAACCGGTCGGGCAACAGCAACCGCTGCGG 3567

KY007522.2016 TGGTTGCAAAATATGATCTTCTGGTGATGACAACCGGTCGGGCAACAGCAACCGCTGCGG 3580

KM034555.2014 TGGTTGCAAAATATGATCTTCTGGTGATGACAACCGGTCGGGCAACAGCAACCGCTGCGG 3592

MH470381.2015 TGGTTGCAAAATATGATCTTCTGGTGATGACAACCGGTCGGGCAACAGCAACCGCTGCGG 3564

MH470382.2015 TGGTTGCAAAATATGATCTTCTGGTGATGACAACCGGTCGGGCAACAGCAACCGCTGCGG 3564

MF102255.2014 TGGTTGCAAAATATGATCTTCTGGTGATGACAACCGGTCGGGCAACAGCAACCGCTGCGG 3577

KJ660348.2014 TGGTTGCAAAATATGATCTTCTGGTGATGACAACCGGTCGGGCAACAGCAACCGCTGCGG 3600

KU143818.2014 TGGTTGCAAAATATGATCTTCTGGTGATGACAACCGGTCGGGCAACAGCAACCGCTGCGG 3600

KT725333.2014 TGGTTGCAAAATATGATCTTCTGGTGATGACAACCGGTCGGGCAACAGCAACCGCTGCGG 3565

KR819004.2014 TGGTTGCAAAATATGATCTTCTGGTGATGACAACCGGTCGGGCAACAGCAACCGCTGCGG 3547

KP271020.2014 TGGTTGCAAAATATGATCTTCTGGTGATGACAACCGGTCGGGCAACAGCAACCGCTGCGG 3547

KM519951.2014 TGGTTGCAAAATATGATCTTCTGGTGATGACAACCGGTCGGGCAACAGCAACCGCTGCGG 3597

MH733488.2018 TGGTTGCAAAATATGATCTTCTGGTGATGACAACCGGTCGGGCAACAGCAACCGCTGCGG 3591

MH733491.2018 TGGTTGCAAAATATGATCTTCTGGTGATGACAACCGGTCGGGCAACAGCAACCGCTGCGG 3586

MH733478.2018 TGGTTGCAAAATATGATCTTCTGGTGATGACAACCGGTCGGGCAACAGCAACCGCTGCGG 3589

MK007330.2018 TGGTTGCAAAATATGATCTTCTGGTGATGACAACCGGTCGGGCAACAGCAACCGCTGCAG 3588

MK007344.2018 TGGTTGCAAAATATGATCTTCTGGTGATGACAACCGGTCGGGCAACAGCAACCGCTGCAG 3582

************************************* *************** **** *

AY142960.1976 CAACTGAGGCTTATTGGGCCGAACATGGTCAACCACCACCTGGACCATCACTTTATGAAG 3660

KC242791.1977 CAACTGAGGCTTATTGGGCCGAACATGGTCAACCACCACCTGGACCATCACTTTATGAAG 3660

AF499101.1976 CAACTGAGGCTTATTGGGCCGAACATGGTCAACCACCACCTGGACCATCACTTTATGAAG 3660

KF113528.2003 CAACTGAGGCTTATTGGGCTGAACATGGTCAACCACCACCTGGACCATCACTTTATGAAG 3656

KC242800.2002 CAACTGAGGCTTATTGGGCTGAACATGGTCAACCACCACCTGGACCATCACTTTATGAAG 3660

KY471090.2001 CAACTGAGGCTTATTGGGCTGAACATGGTCAACCACCACCTGGACCATCACTTTATGAAG 3637

KY471092.2001 CAACTGAGGCTTATTGGGCTGAACATGGTCAACCACCACCTGGACCATCACTTTATGAAG 3637

MH481611.2017 CAACTGAGGCTTATTGGGCCGAACATGGTCAACCACCACCTGGACCATCACTTTATGAAG 3626

MH613311.2017 CAACTGAGGCTTATTGGGCCGAACATGGTCAACCACCACCTGGACCATCACTTTATGAAG 3605

KC242792.1994 CAACTGAGGCTTATTGGGCCGAACATGGTCAACCACCACCTGGACCATCACTTTATGAAG 3660

KC242793.1996 CAACTGAGGCTTATTGGGCCGAACATGGTCAACCACCACCTGGACCATCACTTTATGAAG 3660

KC242794.1996 CAACTGAGGCTTATTGGGCCGAACATGGTCAACCACCACCTGGACCATCACTTTATGAAG 3660

MH121164.1995 CAACTGAGGCTTATTGGGCCGAACATGGTCAACCACCACCTGGACCATCACTTTATGAAG 3660

AY354458.1995 CAACTGAGGCTTATTGGGCCGAACATGGTCAACCACCACCTGGACCATCACTTTATGAAG 3660

KT762962.1995 CAACTGAGGCTTATTGGGCCGAACATGGTCAACCACCACCTGGACCATCACTTTATGAAG 3660

HQ613402.2008 CAACTGAGGCTTATTGGGCTGAACATGGTCAACCACCACCTGGACCATCACTTTATGAAG 3579

KC242789.2007 CAACTGAGGCTTATTGGGCTGAACATGGTCAACCACCACCTGGACCATCACTTTATGAAG 3660

HQ613403.2007 CAACTGAGGCTTATTGGGCTGAACATGGTCAACCACCACCTGGACCATCACTTTATGAAG 3612

KC242785.2007 CAACTGAGGCTTATTGGGCTGAACATGGTCAACCACCACCTGGACCATCACTTTATGAAG 3660

KC242790.2007 CAACTGAGGCTTATTGGGCTGAACATGGTCAACCACCACCTGGACCATCACTTTATGAAG 3660

KU143789.2014 CAACTGAGGCTTATTGGGCTGAACATGGTCAACCACCACCTGGACCATCACTTTATGAAG 3660

KR817168.2014 CAACTGAGGCTTATTGGGCTGAACATGGTCAACCACCACCTGGACCATCACTTTATGAAG 3634

KY426696.2015 CAACTGAGGCTTATTGGGCTGAACATGGTCAACCACCACCTGGACCATCACTTTATGAAG 3660

KR105271.2014 CAACTGAGGCTTATTGGGCTGAACATGGTCAACCACCACCTGGACCATCACTTTATGAAG 3627

KY007522.2016 CAACTGAGGCTTATTGGGCTGAACATGGTCAACCACCACCTGGACCATCACTTTATGAAG 3640

KM034555.2014 CAACTGAGGCTTATTGGGCTGAACATGGTCAACCACCACCTGGACCATCACTTTATGAAG 3652

MH470381.2015 CAACTGAGGCTTATTGGGCTGAACATGGTCAACCACCACCTGGACCATCACTTTATGAAG 3624

MH470382.2015 CAACTGAGGCTTATTGGGCTGAACATGGTCAACCACCACCTGGACCATCACTTTATGAAG 3624

MF102255.2014 CAACTGAGGCTTATTGGGCTGAACATGGTCAACCACCACCTGGACCATCACTTTATGAAG 3637

KJ660348.2014 CAACTGAGGCTTATTGGGCTGAACATGGTCAACCACCACCTGGACCATCACTTTATGAAG 3660

KU143818.2014 CAACTGAGGCTTATTGGGCTGAACATGGTCAACCACCACCTGGACCATCACTTTATGAAG 3660

KT725333.2014 CAACTGAGGCTTATTGGGCTGAACATGGTCAACCACCACCTGGACCATCACTTTATGAAG 3625

KR819004.2014 CAACTGAGGCTTATTGGGCCGAACATGGTCAACCACCACCTGGACCATCACTTTATGAAG 3607

KP271020.2014 CAACTGAGGCTTATTGGGCCGAACATGGTCAACCACCACCTGGACCATCACTTTATGAAG 3607

KM519951.2014 CAACTGAGGCTTATTGGGCCGAACATGGTCAACCACCACCTGGACCATCACTTTATGAAG 3657

MH733488.2018 CAACTGAGGCTTATTGGGCCGAGCATGGTCAACCACCACCTGGACCATCACTTTATGAAG 3651

MH733491.2018 CAACTGAGGCTTATTGGGCCGAGCATGGTCAACCACCACCTGGACCATCACTTTATGAAG 3646

MH733478.2018 CAACTGAGGCTTATTGGGCCGAGCATGGTCAACCACCACCTGGACCATCACTTTATGAAG 3649

MK007330.2018 CAACTGAGGCTTATTGGGCCGAACATGGTCAACCACCACCTGGACCATCACTTTATGAAG 3648

MK007344.2018 CAACTGAGGCTTATTGGGCCGAACATGGTCAACCACCACCTGGACCATCACTTTATGAAG 3642

******************* ** *************************************

AY142960.1976 AAAGTGCGATTCGGGGTAAGATTGAATCTAGAGATGAGACCGTCCCTCAAAGTGTTAGGG 3720

KC242791.1977 AAAGTGCGATTCGGGGTAAGATTGAATCTAGAGATGAGACCGTCCCTCAAAGTGTTAGGG 3720

AF499101.1976 AAAGTGCGATTCGGGGTAAGATTGAATCTAGAGATGAGACCGTCCCTCAAAGTGTTAGGG 3720

KF113528.2003 AAAGTGCAATTCGGGGTAAGATTGAATCTAGAGATGAGACCGTCCCTCAAAGTGTTAGGG 3716

KC242800.2002 AAAGTGCAATTCGGGGTAAGATTGAATCTAGAGATGAGACCGTCCCTCAAAGTGTTAGGG 3720

KY471090.2001 AAAGTGCAATTCGGGGTAAGATTGAATCTAGAGATGAGACCGTCCCTCAAAGTGTTAGGG 3697

KY471092.2001 AAAGTGCAATTCGGGGTAAGATTGAATCTAGAGATGAGACCGTCCCTCAAAGTGTTAGGG 3697

MH481611.2017 AAAGTGCGATTCGGGGTAAGATTGAATCTAGAGATGAGACCGTCCCTCAAAGTGTTAGGG 3686

MH613311.2017 AAAGTGCGATTCGGGGTAAGATTGAATCTAGAGATGAGACCGTCCCTCAAAGTGTTAGGG 3665

KC242792.1994 AAAGTGCGATTCGGGGTAAGATTGAATCTAGAGATGAGACCGTCCCTCAAAGTGTTAGGG 3720

KC242793.1996 AAAGTGCGATTCGGGGTAAGATTGAATCTAGAGATGAGACCGTCCCTCAAAGTGTTAGGG 3720

KC242794.1996 AAAGTGCGATTCGGGGTAAGATTGAATCTAGAGATGAGACCGTCCCTCAAAGTGTTAGGG 3720

MH121164.1995 AAAGTGCAATTCGGGGTAAGATTGAATCTAGAGATGAGACCGTCCCTCAAAGTGTTAGGG 3720

AY354458.1995 AAAGTGCAATTCGGGGTAAGATTGAATCTAGAGATGAGACCGTCCCTCAAAGTGTTAGGG 3720

KT762962.1995 AAAGTGCAATTCGGGGTAAGATTGAATCTAGAGATGAGACCGTCCCTCAAAGTGTTAGGG 3720

HQ613402.2008 AAAGTGCGATTCGGGGTAAGATTGAATCTAGAGGTGAGACCGTCCCTCAAAGTGTTAGGG 3639

KC242789.2007 AAAGTGCGATTCGGGGTAAGATTGAATCTAGAGATGAGACCGTCCCTCAAAGTGTTAGGG 3720

HQ613403.2007 AAAGTGCGATTCGGGGTAAGATTGAATCTAGAGATGAGACCGTCCCTCAAAGTGTTAGGG 3672

KC242785.2007 AAAGTGCGATTCGGGGTAAGATTGAATCTAGAGATGAGACCGTCCCTCAAAGTGTTAGGG 3720

KC242790.2007 AAAGTGCGATTCGGGGTAAGATTGAATCTAGAGATGAGACCGTCCCTCAAAGTGTTAGGG 3720

KU143789.2014 AAAGTGCGATTCGGGGTAAGATTGAATCTAGAGATGAGACTGTCCCTCAAAGTGTTAGGG 3720

KR817168.2014 AAAGTGCGATTCGGGGTAAGATTGAATCTAGAGATGAGACTGTCCCTCAAAGTGTTAGGG 3694

KY426696.2015 AAAGTGCGATTCGGGGTAAGATTGAATCTAGAGATGAGACTGTCCCTCAAAGTGTTAGGG 3720

KR105271.2014 AAAGTGCGATTCGGGGTAAGATTGAATCTAGAGATGAGACTGTCCCTCAAAGTGTTAGGG 3687

KY007522.2016 AAAGTGCGATTCGGGGTAAGATTGAATCTAGAGATGAGACTGTCCCTCAAAGTGTTAGGG 3700

KM034555.2014 AAAGTGCGATTCGGGGTAAGATTGAATCTAGAGATGAGACTGTCCCTCAAAGTGTTAGGG 3712

MH470381.2015 AAAGTGCGATTCGGGGTAAGATTGAATCTAGAGATGAGACTGTCCCTCAAAGTGTTAGGG 3684

MH470382.2015 AAAGTGCGATTCGGGGTAAGATTGAATCTAGAGATGAGACTGTCCCTCAAAGTGTTAGGG 3684

MF102255.2014 AAAGTGCGATTCGGGGTAAGATTGAATCTAGAGATGAGACTGTCCCTCAAAGTGTTAGGG 3697

KJ660348.2014 AAAGTGCGATTCGGGGTAAGATTGAATCTAGAGATGAGACTGTCCCTCAAAGTGTTAGGG 3720

KU143818.2014 AAAGTGCGATTCGGGGTAAGATTGAATCTAGAGATGAGACTGTCCCTCAAAGTGTTAGGG 3720

KT725333.2014 AAAGTGCGATTCGGGGTAAGATTGAATCTAGAGATGAGACTGTCCCTCAAAGTGTTAGGG 3685

KR819004.2014 AAAGTGCGATTCGGGGTAAGATTGAATCTAGAGATGAGACCGTCCCTCAAAGTGTTAGGG 3667

KP271020.2014 AAAGTGCGATTCGGGGTAAGATTGAATCTAGAGATGAGACCGTCCCTCAAAGTGTTAGGG 3667

KM519951.2014 AAAGTGCGATTCGGGGTAAGATTGAATCTAGAGATGAGACCGTCCCTCAAAGTGTTAGGG 3717

MH733488.2018 AAAGTGCGATTCGGGGTAAGATTGAATCTAGAGATGAGACCGTCCCTCAAAGTGTTAGGG 3711

MH733491.2018 AAAGTGCGATTCGGGGTAAGATTGAATCTAGAGATGAGACCGTCCCTCAAAGTGTTAGGG 3706

MH733478.2018 AAAGTGCGATTCGGGGTAAGATTGAATCTAGAGATGAGACCGTCCCTCAAAGTGTTAGGG 3709

MK007330.2018 AAAGTACGATTCGGGGTAAGATTGAATCTAGAGATGAGACCGTCCCTCAAAGTGTTAGGG 3708

MK007344.2018 AAAGTACGATTCGGGGTAAGATTGAATCTAGAGATGAGACCGTCCCTCAAAGTGTTAGGG 3702

***** * ************************* ****** *******************

AY142960.1976 AGGCATTCAACAATCTAAACAGTACCACTTCACTAACTGAGGAAAATTTTGGGAAACCTG 3780

KC242791.1977 AGGCATTCAACAATCTAAACAGTACCACTTCACTAACTGAGGAAAATTTTGGGAAACCTG 3780

AF499101.1976 AGGCATTCAACAATCTAAACAGTACCACTTCACTAACTGAGGAAAATTTTGGGAAACCTG 3780

KF113528.2003 AGGCATTCAACAATCTAGACAGTACCACTTCACTAACTGAGGAAAATTTTGGGAAACCTG 3776

KC242800.2002 AGGCATTCAACAATCTAGACAGTACCACTTCACTAACTGAGGAAAATTTTGGGAAACCTG 3780

KY471090.2001 AGGCATTCAACAATCTAGACAGTACCACTTCACTAACTGAGGAAAATTTTGGGAAACCTG 3757

KY471092.2001 AGGCATTCAACAATCTAGACAGTACCACTTCACTAACTGAGGAAAATTTTGGGAAACCTG 3757

MH481611.2017 AGGCATTCAACAATCTAGACAGTACCACTTCACTAACTGAGGAAAATTTTGGGAAACCTG 3746

MH613311.2017 AGGCATTCAACAATCTAGACAGTACCACTTCACTAACTGAGGAAAATTTTGGGAAACCTG 3725

KC242792.1994 AGGCATTCAACAATCTAGACAGTACCACTTCACTAACTGAGGAAAATTTTGGGAAACCTG 3780

KC242793.1996 AGGCATTCAACAATCTAGACAGTACCACTTCACTAACTGAGGAAAATTTTGGGAAACCTG 3780

KC242794.1996 AGGCATTCAACAATCTAGACAGTACCACTTCACTAACTGAGGAAAATTTTGGGAAACCTG 3780

MH121164.1995 AGGCATTCAACAATCTAGACAGTACCACTTCACTAACTGAGGAAAATTTTGGGAAACCTG 3780

AY354458.1995 AGGCATTCAACAATCTAGACAGTACCACTTCACTAACTGAGGAAAATTTTGGGAAACCTG 3780

KT762962.1995 AGGCATTCAACAATCTAGACAGTACCACTTCACTAACTGAGGAAAATTTTGGGAAACCTG 3780

HQ613402.2008 AGGCATTCAGCAATCTAGACAGTACCACTTCACTAACTGAGGAAAATTTTGGGAAACCTG 3699

KC242789.2007 AGGCATTCAGCAATCTAGACAGTACCACTTCACTAACTGAGGAAAATTTTGGGAAACCTG 3780

HQ613403.2007 AGGCATTCAGCAATCTAGACAGTACCACTTCACTAACTGAGGAAAATTTTGGGAAACCTG 3732

KC242785.2007 AGGCATTCAGCAATCTAGACAGTACCACTTCACTAACTGAGGAAAATTTTGGGAAACCTG 3780

KC242790.2007 AGGCATTCAGCAATCTAGACAGTACCACTTCACTAACTGAGGAAAATTTTGGGAAACCTG 3780

KU143789.2014 AGACAGTCAACAATCTAGACAGTACCACTTCACTAACTGAGGAAAATTTTGGGAAACCTG 3780

KR817168.2014 AGGCATTCAACAATCTAGACAGTACCACTTCACTAACTGAGGAAAATTTTGGGAAACCTG 3754

KY426696.2015 AGGCATTCAACAATCTAGACAGTACCACTTCACTAACTGAGGAAAATTTTGGGAAACCTG 3780

KR105271.2014 AGGCATTCAACAATCTAGACAGTACCACTTCACTAACTGAGGAAAATTTTGGGAAACCTG 3747

KY007522.2016 AGGCATTCAACAATCTAGACAGTACCACTTCACTAACTGAGGAAAATTTTGGGAAACCTG 3760

KM034555.2014 AGGCATTCAACAATCTAGACAGTACCACTTCACTAACTGAGGAAAATTTTGGGAAACCTG 3772

MH470381.2015 AGGCATTCAACAATCTAGACAGTACCACTTCACTAACTGAGGAAAATTTTGGGAAACCTG 3744

MH470382.2015 AGGCATTCAACAATCTAGACAGTACCACTTCACTAACTGAGGAAAATTTTGGGAAACCTG 3744

MF102255.2014 AGGCATTCAACAATCTAGACAGTACCACTTCACTAACTGAGGAAAATTTTGGGAAACCTG 3757

KJ660348.2014 AGGCATTCAACAATCTAGACAGTACCACTTCACTAACTGAGGAAAATTTTGGGAAACCTG 3780

KU143818.2014 AGGCATTCAACAATCTAGACAGTACCACTTCACTAACTGAGGAAAATTTTGGGAAACCTG 3780

KT725333.2014 AGGCATTCAACAATCTAGACAGTACCACTTCACTAACTGAGGAAAATTTTGGGAAACCTG 3745

KR819004.2014 AGGCATTCAACAATCTAGACAGTACCACTTCACTAACTGAGGAAAATTTTGGGAAACCTG 3727

KP271020.2014 AGGCATTCAACAATCTAGACAGTACCACTTCACTAACTGAGGAAAATTTTGGGAAACCTG 3727

KM519951.2014 AGGCATTCAACAATCTAGACAGTACCACTTCACTAACTGAGGAAAATTTTGGGAAACCTG 3777

MH733488.2018 AGGCATTCAACAATCTAGACAGTACCACTTCACTAACTGAGGAAAATTTTGGGAAACCTG 3771

MH733491.2018 AGGCATTCAACAATCTAGACAGTACCACTTCACTAACTGAGGAAAATTTTGGGAAACCTG 3766

MH733478.2018 AGGCATTCAACAATCTAGACAGTACCACTTCACTAACTGAGGAAAATTTTGGGAAACCTG 3769

MK007330.2018 AGGCATTCAACAATCTAGACAGTACCACTTCACTAACTGAGGAAAATTTTGGGAAACCTG 3768

MK007344.2018 AGGCATTCAACAATCTAGACAGTACCACTTCACTAACTGAGGAAAATTTTGGGAAACCTG 3762

** ** *** ******* ******************************************

AY142960.1976 ACATTTCGGCAAAGGATTTGAGAAACATTATGTATGATCACTTGCCTGGTTTTGGAACTG 3840

KC242791.1977 ACATTTCGGCAAAGGATTTGAGAAACATTATGTATGATCACTTGCCTGGTTTTGGAACTG 3840

AF499101.1976 ACATTTCGGCAAAGGATTTGAGAAACATTATGTATGATCACTTGCCTGGTTTTGGAACTG 3840

KF113528.2003 ACATTTCAGCAAAGGATTTGAGAAACATTATGTATGATCACTTGCCTGGTTTTGGAACTG 3836

KC242800.2002 ACATTTCAGCAAAGGATTTGAGAAACATTATGTATGATCACTTGCCTGGTTTTGGAACTG 3840

KY471090.2001 ACATTTCAGCAAAGGATTTGAGAAACATTATGTATGATCACTTGCCTGGTTTTGGAACTG 3817

KY471092.2001 ACATTTCAGCAAAGGATTTGAGAAACATTATGTATGATCACTTGCCTGGTTTTGGAACTG 3817

MH481611.2017 ACATTTCGGCAAAGGATTTGAGAAACATTATGTATGATCACTTGCCTGGTTTTGGAACTG 3806

MH613311.2017 ACATTTCGGCAAAGGATTTGAGAAACATTATGTATGATCACTTGCCTGGTTTTGGAACTG 3785

KC242792.1994 ACATTTCGGCAAAGGATTTGAGAAACATTATGTATGATCACTTGCCTGGTTTTGGAACTG 3840

KC242793.1996 ACATTTCGGCAAAGGATTTGAGAAACATTATGTATGATCACTTGCCTGGTTTTGGAACTG 3840

KC242794.1996 ACATTTCGGCAAAGGATTTGAGAAACATTATGTATGATCACTTGCCTGGTTTTGGAACTG 3840

MH121164.1995 ACATTTCGGCAAAGGATTTGAGAAACATTATGTATGATCACTTGCCTGGTTTTGGAACTG 3840

AY354458.1995 ACATTTCGGCAAAGGATTTGAGAAACATTATGTATGATCACTTGCCTGGTTTTGGAACTG 3840

KT762962.1995 ACATTTCGGCAAAGGATTTGAGAAACATTATGTATGATCACTTGCCTGGTTTTGGAACTG 3840

HQ613402.2008 ACATTTCGGCAAAGGATTTGAGAAACATTATGTATGATCACTTGCCTGGTTTTGGAACTG 3759

KC242789.2007 ACATTTCGGCAAAGGATTTGAGAAACATTATGTATGATCACTTGCCTGGTTTTGGAACTG 3840

HQ613403.2007 ACATTTCGGCAAAGGATTTGAGAAACATTATGTATGATCACTTGCCTGGTTTTGGAACTG 3792

KC242785.2007 ACATTTCGGCAAAGGATTTGAGAAACATTATGTATGATCACTTGCCTGGTTTTGGAACTG 3840

KC242790.2007 ACATTTCGGCAAAGGATTTGAGAAACATTATGTATGATCACTTGCCTGGTTTTGGAACTG 3840

KU143789.2014 ACATTTCGGCAAAGGATTTGAGAAACATTATGTATGATCACTTGCCTGGTTTTGGAACTG 3840

KR817168.2014 ACATTTCGGCAAAGGATTTGAGAAACATTATGTATGATCACTTGCCTGGTTTTGGAACTG 3814

KY426696.2015 ACATTTCGGCAAAGGATTTGAGAAACATTATGTATGATCACTTGCCTGGTTTTGGAACTG 3840

KR105271.2014 ACATTTCGGCAAAGGATTTGAGAAACATTATGTATGATCACTTGCCTGGTTTTGGAACTG 3807

KY007522.2016 ACATTTCGGCAAAGGATTTGAGAAACATTATGTATGATCACTTGCCTGGTTTTGGAACTG 3820

KM034555.2014 ACATTTCGGCAAAGGATTTGAGAAACATTATGTATGATCACTTGCCTGGTTTTGGAACTG 3832

MH470381.2015 ACATTTCGGCAAAGGATTTGAGAAACATTATGTATGATCACTTGCCTGGTTTTGGAACTG 3804

MH470382.2015 ACATTTCGGCAAAGGATTTGAGAAACATTATGTATGATCACTTGCCTGGTTTTGGAACTG 3804

MF102255.2014 ACATTTCGGCAAAGGATTTGAGAAACATTATGTATGATCACTTGCCTGGTTTTGGAACTG 3817

KJ660348.2014 ACATTTCGGCAAAGGATTTGAGAAACATTATGTATGATCACTTGCCTGGTTTTGGAACTG 3840

KU143818.2014 ACATTTCGGCAAAGGATTTGAGAAACATTATGTATGATCACTTGCCTGGTTTTGGAACTG 3840

KT725333.2014 ACATTTCGGCAAAGGATTTGAGAAACATTATGTATGATCACTTGCCTGGTTTTGGAACTG 3805

KR819004.2014 ACATTTCGGCAAAGGATTTGAGAAACATTATGTATGATCACTTGCCTGGTTTTGGAACTG 3787

KP271020.2014 ACATTTCGGCAAAGGATTTGAGAAACATTATGTATGATCACTTGCCTGGTTTTGGAACTG 3787

KM519951.2014 ACATTTCGGCAAAGGATTTGAGAAACATTATGTATGATCACTTGCCTGGTTTTGGAACTG 3837

MH733488.2018 ACATTTCGGCAAAGGATTTGAGAAACATTATGTATGATCACTTGCCTGGTTTTGGAACTG 3831

MH733491.2018 ACATTTCGGCAAAGGATTTGAGAAACATTATGTATGATCACTTGCCTGGTTTTGGAACTG 3826

MH733478.2018 ACATTTCGGCAAAGGATTTGAGAAACATTATGTATGATCACTTGCCTGGTTTTGGAACTG 3829

MK007330.2018 ACATTTCGGCAAAGGATCTGAGAAACATTATGTATGATCACTTGCCTGGTTTTGGAACTG 3828

MK007344.2018 ACATTTCGGCAAAGGATCTGAGAAACATTATGTATGATCACTTGCCTGGTTTTGGAACTG 3822

******* ********* ******************************************

AY142960.1976 CTTTCCACCAATTAGTACAAGTGATTTGTAAATTGGGAAAAGATAGCAACTCATTGGACA 3900

KC242791.1977 CTTTCCACCAATTAGTACAAGTGATTTGTAAATTGGGAAAAGATAGCAACTCATTGGACA 3900

AF499101.1976 CTTTCCACCAATTAGTACAAGTGATTTGTAAATTGGGAAAAGATAGCAACTCATTGGACA 3900

KF113528.2003 CTTTCCACCAATTAGTACAAGTGATTTGTAAATTGGGAAAAGATAGCAACTCATTGGATA 3896

KC242800.2002 CTTTCCACCAATTAGTACAAGTGATTTGTAAATTGGGAAAAGATAGCAACTCATTGGATA 3900

KY471090.2001 CTTTCCACCAATTAGTACAAGTGATTTGTAAATTGGGAAAAGATAGCAACTCATTGGATA 3877

KY471092.2001 CTTTCCACCAATTAGTACAAGTGATTTGTAAATTGGGAAAAGATAGCAACTCATTGGATA 3877

MH481611.2017 CTTTCCACCAATTAGTACAAGTGATTTGTAAATTGGGAAAAGATAGCAACTCATTGGACA 3866

MH613311.2017 CTTTCCACCAATTAGTACAAGTGATTTGTAAATTGGGAAAAGATAGCAACTCATTGGACA 3845

KC242792.1994 CTTTCCACCAATTAGTACAAGTGATTTGTAAATTGGGAAAAGATAGCAACTCATTGGACA 3900

KC242793.1996 CTTTCCACCAATTAGTACAAGTGATTTGTAAATTGGGAAAAGATAGCAACTCATTGGACA 3900

KC242794.1996 CTTTCCACCAATTAGTACAAGTGATTTGTAAATTGGGAAAAGATAGCAACTCATTGGACA 3900

MH121164.1995 CTTTCCACCAATTAGTACAAGTGATTTGTAAATTGGGAAAAGATAGCAACTCATTGGACA 3900

AY354458.1995 CTTTCCACCAATTAGTACAAGTGATTTGTAAATTGGGAAAAGATAGCAACTCATTGGACA 3900

KT762962.1995 CTTTCCACCAATTAGTACAAGTGATTTGTAAATTGGGAAAAGATAGCAACTCATTGGACA 3900

HQ613402.2008 CTTTCCACCAGTTAGTACAAGTGATTTGTAAATTGGGAAAGGATAGCAACTCATTGGACA 3819

KC242789.2007 CTTTCCACCAGTTAGTACAAGTGATTTGTAAATTGGGAAAGGATAGCAACTCATTGGACA 3900

HQ613403.2007 CTTTCCACCAGTTAGTACAAGTGATTTGTAAATTGGGAAAGGATAGCAACTCATTGGACA 3852

KC242785.2007 CTTTCCACCAGTTAGTACAAGTGATTTGTAAATTGGGAAAGGATAGCAACTCATTGGACA 3900

KC242790.2007 CTTTCCACCAGTTAGTACAAGTGATTTGTAAATTGGGAAAGGATAGCAACTCATTGGACA 3900

KU143789.2014 CTTTCCACCAATTAGTACAAGTGATTTGTAAATTGGGAAAAGATAGCAATTCATTGGACA 3900

KR817168.2014 CTTTCCACCAATTAGTACAAGTGATTTGTAAATTGGGAAAAGATAGCAATTCATTGGACA 3874

KY426696.2015 CTTTCCACCAATTAGTACAAGTGATTTGTAAATTGGGAAAAGATAGCAATTCATTGGACA 3900

KR105271.2014 CTTTCCACCAATTAGTACAAGTGATTTGTAAATTGGGAAAAGATAGCAATTCATTGGACA 3867

KY007522.2016 CTTTCCACCAATTAGTACAAGTGATTTGTAAATTGGGAAAAGATAGCAATTCATTGGACA 3880

KM034555.2014 CTTTCCACCAATTAGTACAAGTGATTTGTAAATTGGGAAAAGATAGCAATTCATTGGACA 3892

MH470381.2015 CTTTCCACCAATTAGTACAAGTGATTTGTAAATTGGGAAAAGATAGCAATTCATTGGACA 3864

MH470382.2015 CTTTCCACCAATTAGTACAAGTGATTTGTAAATTGGGAAAAGATAGCAATTCATTGGACA 3864

MF102255.2014 CTTTCCACCAATTAGTACAAGTGATTTGTAAATTGGGAAAAGATAGCAATTCATTGGACA 3877

KJ660348.2014 CTTTCCACCAATTAGTACAAGTGATTTGTAAATTGGGAAAAGATAGCAATTCATTGGACA 3900

KU143818.2014 CTTTCCACCAATTAGTACAAGTGATTTGTAAATTGGGAAAAGATAGCAATTCATTGGACA 3900

KT725333.2014 CTTTCCACCAATTAGTACAAGTGATTTGTAAATTGGGAAAAGATAGCAATTCATTGGACA 3865

KR819004.2014 CTTTCCACCAATTAGTACAAGTGATTTGTAAATTGGGAAAAGATAGCAACTCATTGGACA 3847

KP271020.2014 CTTTCCACCAATTAGTACAAGTGATTTGTAAATTGGGAAAAGATAGCAACTCATTGGACA 3847

KM519951.2014 CTTTCCACCAATTAGTACAAGTGATTTGTAAATTGGGAAAAGATAGCAACTCATTGGACA 3897

MH733488.2018 CTTTCCACCAATTAGTACAAGTGATTTGTAAATTGGGAAAAGATAGCAACTCATTGGACA 3891

MH733491.2018 CTTTCCACCAATTAGTACAAGTGATTTGTAAATTGGGAAAAGATAGCAACTCATTGGACA 3886

MH733478.2018 CTTTCCACCAATTAGTACAAGTGATTTGTAAATTGGGAAAAGATAGCAACTCATTGGACA 3889

MK007330.2018 CTTTCCACCAATTAGTACAAGTGATTTGTAAATTGGGAAAAGATAGCAACTCATTGGACA 3888

MK007344.2018 CTTTCCACCAATTAGTACAAGTGATTTGTAAATTGGGAAAAGATAGCAACTCATTGGACA 3882

********** ***************************** ******** ******** *

AY142960.1976 TCATTCATGCTGAGTTCCAGGCCAGCCTGGCTGAAGGAGACTCTCCTCAATGTGCCCTAA 3960

KC242791.1977 TCATTCATGCTGAGTTCCAGGCCAGCCTGGCTGAAGGAGACTCTCCTCAATGTGCCCTAA 3960

AF499101.1976 TCATTCATGCTGAGTTCCAGGCCAGCCTGGCTGAAGGAGACTCTCCTCAATGTGCCCTAA 3960

KF113528.2003 TCATTCATGCTGAGTTCCAGGCCAGCCTGGCTGAAGGAGACTCTCCTCAATGTGCCCTAA 3956

KC242800.2002 TCATTCATGCTGAGTTCCAGGCCAGCCTGGCTGAAGGAGACTCTCCTCAATGTGCCCTAA 3960

KY471090.2001 TCATTCATGCTGAGTTCCAGGCCAGCCTGGCTGAAGGAGACTCTCCTCAATGTGCCCTAA 3937

KY471092.2001 TCATTCATGCTGAGTTCCAGGCCAGCCTGGCTGAAGGAGACTCTCCTCAATGTGCCCTAA 3937

MH481611.2017 TCATTCATGCTGAGTTCCAGGCCAGCCTGGCTGAAGGAGACTCTCCTCAATGTGCCCTAA 3926

MH613311.2017 TCATTCATGCTGAGTTCCAGGCCAGCCTGGCTGAAGGAGACTCTCCTCAATGTGCCCTAA 3905

KC242792.1994 TCATTCATGCTGAGTTCCAGGCCAGCCTGGCTGAAGGAGACTCTCCTCAATGTGCCCTAA 3960

KC242793.1996 TCATTCATGCTGAGTTCCAGGCCAGCCTGGCTGAAGGAGACTCTCCTCAATGTGCCCTAA 3960

KC242794.1996 TCATTCATGCTGAGTTCCAGGCCAGCCTGGCTGAAGGAGACTCTCCTCAATGTGCCCTAA 3960

MH121164.1995 TCATTCATGCTGAGTTCCAGGCCAGCCTGGCTGAAGGAGACTCTCCTCAATGTGCCCTAA 3960

AY354458.1995 TCATTCATGCTGAGTTCCAGGCCAGCCTGGCTGAAGGAGACTCTCCTCAATGTGCCCTAA 3960

KT762962.1995 TCATTCATGCTGAGTTCCAGGCCAGCCTGGCTGAAGGAGACTCTCCTCAATGTGCCCTAA 3960

HQ613402.2008 TCATTCATGCTGAGTTCCAGGCCAGCCTGGCTGAAGGAGACTCTCCTCAATGTGCCCTAA 3879

KC242789.2007 TCATTCATGCTGAGTTCCAGGCCAGCCTGGCTGAAGGAGACTCTCCTCAATGTGCCCTAA 3960

HQ613403.2007 TCATTCATGCTGAGTTCCAGGCCAGCCTGGCTGAAGGAGACTCTCCTCAATGTGCCCTAA 3912

KC242785.2007 TCATTCATGCTGAGTTCCAGGCCAGCCTGGCTGAAGGAGACTCTCCTCAATGTGCCCTAA 3960

KC242790.2007 TCATTCATGCTGAGTTCCAGGCCAGCCTGGCTGAAGGAGACTCTCCTCAATGTGCCCTAA 3960

KU143789.2014 TTATTCATGCTGAGTTCCAGGCCAGCCTGGCTGAAGGAGACTCCCCTCAATGTGCCCTAA 3960

KR817168.2014 TTATTCATGCTGAGTTCCAGGCCAGCCTGGCTGAAGGAGACTCCCCTCAATGTGCCCTAA 3934

KY426696.2015 TTATTCATGCTGAGTTCCAGGCCAGCCTGGCTGAAGGAGACTCCCCTCAATGTGCCCTAA 3960

KR105271.2014 TTATTCATGCTGAGTTCCAGGCCAGCCTGGCTGAAGGAGACTCCCCTCAATGTGCCCTAA 3927

KY007522.2016 TTATTCATGCTGAGTTCCAGGCCAGCCTGGCTGAAGGAGACTCCCCTCAATGTGCCCTAA 3940

KM034555.2014 TTATTCATGCTGAGTTCCAGGCCAGCCTGGCTGAAGGAGACTCCCCTCAATGTGCCCTAA 3952

MH470381.2015 TTATTCATGCTGAGTTCCAGGCCAGCCTGGCTGAAGGAGACTCCCCTCAATGTGCCCTAA 3924

MH470382.2015 TTATTCATGCTGAGTTCCAGGCCAGCCTGGCTGAAGGAGACTCCCCTCAATGTGCCCTAA 3924

MF102255.2014 TTATTCATGCTGAGTTCCAGGCCAGCCTGGCTGAAGGAGACTCCCCTCAATGTGCCCTAA 3937

KJ660348.2014 TTATTCATGCTGAGTTCCAGGCCAGCCTGGCTGAAGGAGACTCCCCTCAATGTGCCCTAA 3960

KU143818.2014 TTATTCATGCTGAGTTCCAGGCCAGCCTGGCTGAAGGAGACTCCCCTCAATGTGCCCTAA 3960

KT725333.2014 TTATTCATGCTGAGTTCCAGGCCAGCCTGGCTGAAGGAGACTCCCCTCAATGTGCCCTAA 3925

KR819004.2014 TCATTCATGCTGAGTTCCAGGCCAGCCTGGCTGAAGGAGACTCTCCTCAATGTGCCCTAA 3907

KP271020.2014 TCATTCATGCTGAGTTCCAGGCCAGCCTGGCTGAAGGAGACTCTCCTCAATGTGCCCTAA 3907

KM519951.2014 TCATTCATGCTGAGTTCCAGGCCAGCCTGGCTGAAGGAGACTCTCCTCAATGTGCCCTAA 3957

MH733488.2018 TCATTCATGCTGAGTTCCAGGCCAGCCTGGCTGAAGGAGACTCTCCTCAATGTGCCCTAA 3951

MH733491.2018 TCATTCATGCTGAGTTCCAGGCCAGCCTGGCTGAAGGAGACTCTCCTCAATGTGCCCTAA 3946

MH733478.2018 TCATTCATGCTGAGTTCCAGGCCAGCCTGGCTGAAGGAGACTCTCCTCAATGTGCCCTAA 3949

MK007330.2018 TCATTCATGCTGAGTTCCAGGCCAGCCTGGCTGAAGGAGACTCTCCGCAATGTGCCCTAA 3948

MK007344.2018 TCATTCATGCTGAGTTCCAGGCCAGCCTGGCTGAAGGAGACTCTCCGCAATGTGCCCTAA 3942

* ***************************************** ** *************

AY142960.1976 TTCAAATTACAAAAAGAGTTCCAATCTTCCAAGATGCTGCTCCACCTGTCATCCACATCC 4020

KC242791.1977 TTCAAATTACAAAAAGAGTTCCAATCTTCCAAGATGCTGCTCCACCTGTCATCCACATCC 4020

AF499101.1976 TTCAAATTACAAAAAGAGTTCCAATCTTCCAAGATGCTGCTCCACCTGTCATCCACATCC 4020

KF113528.2003 TTCAAATTACAAAAAGAGTTCCAATCTTCCAAGATGCTGCTCCACCTGTCATCCACATCC 4016

KC242800.2002 TTCAAATTACAAAAAGAGTTCCAATCTTCCAAGATGCTGCTCCACCTGTCATCCACATCC 4020

KY471090.2001 TTCAAATTACAAAAAGAGTTCCAATCTTCCAAGATGCTGCTCCACCTGTCATCCACATCC 3997

KY471092.2001 TTCAAATTACAAAAAGAGTTCCAATCTTCCAAGATGCTGCTCCACCTGTCATCCACATCC 3997

MH481611.2017 TTCAAATTACAAAAAGAGTTCCAATCTTCCAAGATGCTGCTCCACCTGTCATCCACATCC 3986

MH613311.2017 TTCAAATTACAAAAAGAGTTCCAATCTTCCAAGATGCTGCTCCACCTGTCATCCACATCC 3965

KC242792.1994 TTCAAATTACAAAAAGAGTTCCAATCTTCCAAGATGCTGCTCCACCTGTCATCCACATCC 4020

KC242793.1996 TTCAAATTACAAAAAGAGTTCCAATCTTCCAAGATGCTGCTCCACCTGTCATCCACATCC 4020

KC242794.1996 TTCAAATTACAAAAAGAGTTCCAATCTTCCAAGATGCTGCTCCACCTGTCATCCACATCC 4020

MH121164.1995 TTCAAATTACAAAAAGAGTTCCAATCTTCCAAGATGCTGCTCCACCTGTCATCCACATCC 4020

AY354458.1995 TTCAAATTACAAAAAGAGTTCCAATCTTCCAAGATGCTGCTCCACCTGTCATCCACATCC 4020

KT762962.1995 TTCAAATTACAAAAAGAGTTCCAATCTTCCAAGATGCTGCTCCACCTGTCATCCACATCC 4020

HQ613402.2008 TTCAAATCACAAAAAGAGTTCCAATCTTCCAAGATGCTGCTCCACCTGTCATCCACATCC 3939

KC242789.2007 TTCAAATCACAAAAAGAGTTCCAATCTTCCAAGATGCTGCTCCACCTGTCATCCACATCC 4020

HQ613403.2007 TTCAAATCACAAAAAGAGTTCCAATCTTCCAAGATGCTGCTCCACCTGTCATCCACATCC 3972

KC242785.2007 TTCAAATCACAAAAAGAGTTCCAATCTTCCAAGATGCTGCTCCACCTGTCATCCACATCC 4020

KC242790.2007 TTCAAATCACAAAAAGAGTTCCAATCTTCCAAGATGCTGCTCCACCTGTCATCCACATCC 4020

KU143789.2014 TTCAAATTACAAAAAGAGTTCCAATCTTCCAAGATGCTGCTCCACCTGTCATCCACATCC 4020

KR817168.2014 TTCAAATTACAAAAAGAGTTCCAATCTTCCAAGATGCTGCTCCACCTGTCATCCACATCC 3994

KY426696.2015 TTCAAATTACAAAAAGAGTTCCAATCTTCCAAGATGCTGCTCCACCTGTCATCCACATCC 4020

KR105271.2014 TTCAAATTACAAAAAGAGTTCCAATCTTCCAAGATGCTGCTCCACCTGTCATCCACATCC 3987

KY007522.2016 TTCAAATTACAAAAAGAGTTCCAATCTTCCAAGATGCTGCTCCACCTGTCATCCACATCC 4000

KM034555.2014 TTCAAATTACAAAAAGAGTTCCAATCTTCCAAGATGCTGCTCCACCTGTCATCCACATCC 4012

MH470381.2015 TTCAAATTACAAAAAGAGTTCCAATCTTCCAAGATGCTGCTCCACCTGTCATCCACATCC 3984

MH470382.2015 TTCAAATTACAAAAAGAGTTCCAATCTTCCAAGATGCTGCTCCACCTGTCATCCACATCC 3984

MF102255.2014 TTCAAATTACAAAAAGAGTTCCAATCTTCCAAGATGCTGCTCCACCTGTCATCCACATCC 3997

KJ660348.2014 TTCAAATTACAAAAAGAGTTCCAATCTTCCAAGATGCTGCTCCACCTGTCATCCACATCC 4020

KU143818.2014 TTCAAATTACAAAAAGAGTTCCAATCTTCCAAGATGCTGCTCCACCTGTCATCCACATCC 4020

KT725333.2014 TTCAAATTACAAAAAGAGTTCCAATCTTCCAAGATGCTGCTCCACCTGTCATCCACATCC 3985

KR819004.2014 TTCAAATTACAAAAAGAGTTCCAATCTTCCAAGATGCTGCTCCACCTGTCATCCACATCC 3967

KP271020.2014 TTCAAATTACAAAAAGAGTTCCAATCTTCCAAGATGCTGCTCCACCTGTCATCCACATCC 3967

KM519951.2014 TTCAAATTACAAAAAGAGTTCCAATCTTCCAAGATGCTGCTCCACCTGTCATCCACATCC 4017

MH733488.2018 TTCAAATTACAAAAAGAGTTCCAATCTTCCAAGATGCTGCTCCACCTGTCATACACATCC 4011

MH733491.2018 TTCAAATTACAAAAAGAGTTCCAATCTTCCAAGATGCTGCTCCACCTGTCATACACATCC 4006

MH733478.2018 TTCAAATTACAAAAAGAGTTCCAATCTTCCAAGATGCTGCTCCACCTGTCATACACATCC 4009

MK007330.2018 TTCAAATTACGAAAAGAGTTCCAATCTTCCAAGATGCTGCTCCACCTGTCATCCACATCC 4008

MK007344.2018 TTCAAATTACGAAAAGAGTTCCAATCTTCCAAGATGCTGCTCCACCTGTCATCCACATCC 4002

******* ** ***************************************** *******

AY142960.1976 GCTCTCGAGGTGACATTCCCCGAGCTTGCCAGAAAAGCTTGCGTCCAGTCCCACCATCGC 4080

KC242791.1977 GCTCTCGAGGTGACATTCCCCGAGCTTGCCAGAAAAGCTTGCGTCCAGTCCCACCATCGC 4080

AF499101.1976 GCTCTCGAGGTGACATTCCCCGAGCTTGCCAGAAAAGCTTGCGTCCAGTCCCACCATCGC 4080

KF113528.2003 GCTCTCGAGGTGACATTCCCCGAGCTTGCCAGAAAAGCTTGCGTCCAGTCCCGCCATCAC 4076

KC242800.2002 GCTCTCGAGGTGACATTCCCCGAGCTTGCCAGAAAAGCTTGCGTCCAGTCCCGCCATCAC 4080

KY471090.2001 GCTCTCGAGGTGACATTCCCCGAGCTTGCCAGAAAAGCTTGCGTCCAGTCCCGCCATCAC 4057

KY471092.2001 GCTCTCGAGGTGACATTCCCCGAGCTTGCCAGAAAAGCTTGCGTCCAGTCCCGCCATCAC 4057

MH481611.2017 GCTCTCGAGGTGACATTCCCCGAGCTTGCCAGAAAAGCTTGCGTCCAGTCCCACCATCGC 4046

MH613311.2017 GCTCTCGAGGTGACATTCCCCGAGCTTGCCAGAAAAGCTTGCGTCCAGTCCCACCATCGC 4025

KC242792.1994 GCTCTCGAGGTGACATTCCCCGAGCTTGCCAGAAAAGCTTGCGTCCAGTCCCACCATCGC 4080

KC242793.1996 GCTCTCGAGGTGACATTCCCCGAGCTTGCCAGAAAAGCTTGCGTCCAGTCCCACCATCGC 4080

KC242794.1996 GCTCTCGAGGTGACATTCCCCGAGCTTGCCAGAAAAGCTTGCGTCCAGTCCCACCATCGC 4080

MH121164.1995 GCTCTCGAGGTGACATTCCCCGAGCTTGCCAGAAAAGCTTGCGTCCAGTCCCACCATCGC 4080

AY354458.1995 GCTCTCGAGGTGACATTCCCCGAGCTTGCCAGAAAAGCTTGCGTCCAGTCCCACCATCGC 4080

KT762962.1995 GCTCTCGAGGTGACATTCCCCGAGCTTGCCAGAAAAGCTTGCGTCCAGTCCCACCATCGC 4080

HQ613402.2008 GCTCTCGAGGTGACATTCCCCGAGCTTGCCAGAAGAGCTTACGTCCAGTCCCGCCATCGC 3999

KC242789.2007 GCTCTCGAGGTGACATTCCCCGAGCTTGCCAGAAGAGCTTACGTCCAGTCCCGCCATCGC 4080

HQ613403.2007 GCTCTCGAGGTGACATTCCCCGAGCTTGCCAGAAGAGCTTACGTCCAGTCCCGCCATCGC 4032

KC242785.2007 GCTCTCGAGGTGACATTCCCCGAGCTTGCCAGAAGAGCTTACGTCCAGTCCCGCCATCGC 4080

KC242790.2007 GCTCTCGAGGTGACATTCCCCGAGCTTGCCAGAAGAGCTTACGTCCAGTCCCGCCATCGC 4080

KU143789.2014 GCTCTCGAGGTGACATTCCCCGAGCTTGCCAGAAGAGCTTGCGTCCAGTCCCACCATCAC 4080

KR817168.2014 GCTCTCGAGGTGACATTCCCCGAGCTTGCCAGAAGAGCTTGCGTCCAGTCCCACCATCAC 4054

KY426696.2015 GCTCTCGAGGTGACATTCCCCGAGCTTGCCAGAAGAGCTTGCGTCCAGTCCCACCATCAC 4080

KR105271.2014 GCTCTCGAGGTGACATTCCCCGAGCTTGCCAGAAGAGCTTGCGTCCAGTCCCACCATCAC 4047

KY007522.2016 GCTCTCGAGGTGACATTCCCCGAGCTTGCCAGAAGAGCTTGCGTCCAGTCCCACCATCAC 4060

KM034555.2014 GCTCTCGAGGTGACATTCCCCGAGCTTGCCAGAAGAGCTTGCGTCCAGTCCCACCATCAC 4072

MH470381.2015 GCTCTCGAGGTGACATTCCCCGAGCTTGCCAGAAGAGCTTGCGTCCAGTCCCACCATCAC 4044

MH470382.2015 GCTCTCGAGGTGACATTCCCCGAGCTTGCCAGAAGAGCTTGCGTCCAGTCCCACCATCAC 4044

MF102255.2014 GCTCTCGAGGTGACATTCCCCGAGCTTGCCAGAAGAGCTTGCGTCCAGTCCCACCATCAC 4057

KJ660348.2014 GCTCTCGAGGTGACATTCCCCGAGCTTGCCAGAAGAGCTTGCGTCCAGTCCCACCATCAC 4080

KU143818.2014 GCTCTCGAGGTGACATTCCCCGAGCTTGCCAGAAGAGCTTGCGTCCAGTCCCACCATCAC 4080

KT725333.2014 GCTCTCGAGGTGACATTCCCCGAGCTTGCCAGAAGAGCTTGCGTCCAGTCCCACCATCAC 4045

KR819004.2014 GCTCTCGAGGTGACATTCCTCGAGCTTGCCAGAAAAGCTTGCGTCCAGTCCCACCATCGC 4027

KP271020.2014 GCTCTCGAGGTGACATTCCTCGAGCTTGCCAGAAAAGCTTGCGTCCAGTCCCACCATCGC 4027

KM519951.2014 GCTCTCGAGGTGACATTCCTCGAGCTTGCCAGAAAAGCTTGCGTCCAGTCCCACCATCGC 4077

MH733488.2018 GCTCTCGAGGTGACATTCCTCGAGCTTGCCAGAAAAGCTTGCGTCCAGTCCCACCATCGC 4071

MH733491.2018 GCTCTCGAGGTGACATTCCTCGAGCTTGCCAGAAAAGCTTGCGTCCAGTCCCACCATCGC 4066

MH733478.2018 GCTCTCGAGGTGACATTCCTCGAGCTTGCCAGAAAAGCTTGCGTCCAGTCCCACCATCGC 4069

MK007330.2018 GCTCTCGAGGTGACATTCCCCGAGCTTGTCAGAAAAGCTTGCGTCCAGTCCCACCATCGC 4068

MK007344.2018 GCTCTCGAGGTGACATTCCCCGAGCTTGTCAGAAAAGCTTGCGTCCAGTCCCACCATCGC 4062

******************* ******** ***** ***** *********** ***** *

AY142960.1976 CCAAGATTGATCGAGGTTGGGTATGTGTTTTTCAGCTTCAAGATGGTAAAACACTTGGAC 4140

KC242791.1977 CCAAGATTGATCGAGGTTGGGTATGTGTTTTTCAGCTTCAAGATGGTAAAACACTTGGAC 4140

AF499101.1976 CCAAGATTGATCGAGGTTGGGTATGTGTTTTTCAGCTTCAAGATGGTAAAACACTTGGAC 4140

KF113528.2003 CCAAGATTGATCGAGGTTGGGTATGTGTTTTCCAGCTTCAAGATGGTAAAACACTTGGAC 4136

KC242800.2002 CCAAGATTGATCGAGGTTGGGTATGTGTTTTCCAGCTTCAAGATGGTAAAACACTTGGAC 4140

KY471090.2001 CCAAGATTGATCGAGGTTGGGTATGTGTTTTCCAGCTTCAAGATGGTAAAACACTTGGAC 4117

KY471092.2001 CCAAGATTGATCGAGGTTGGGTATGTGTTTTCCAGCTTCAAGATGGTAAAACACTTGGAC 4117

MH481611.2017 CCAAGATTGATCGAGGTTGGGTATGTGTTTTTCAGCTTCAAGATGGTAAAACACTTGGAC 4106

MH613311.2017 CCAAGATTGATCGAGGTTGGGTATGTGTTTTTCAGCTTCAAGATGGTAAAACACTTGGAC 4085

KC242792.1994 CCAAGATTGATCGAGGTTGGGTATGTGTTTTTCAGCTTCAAGATGGTAAAACACTTGGAC 4140

KC242793.1996 CCAAGATTGATCGAGGTTGGGTATGTGTTTTTCAGCTTCAAGATGGTAAAACACTTGGAC 4140

KC242794.1996 CCAAGATTGATCGAGGTTGGGTATGTGTTTTTCAGCTTCAAGATGGTAAAACACTTGGAC 4140

MH121164.1995 CCAAGATTGATCGAGGTTGGGTATGTGTTTTTCAGCTTCAAGATGGTAAAACACTTGGAC 4140

AY354458.1995 CCAAGATTGATCGAGGTTGGGTATGTGTTTTTCAGCTTCAAGATGGTAAAACACTTGGAC 4140

KT762962.1995 CCAAGATTGATCGAGGTTGGGTATGTGTTTTTCAGCTTCAAGATGGTAAAACACTTGGAC 4140

HQ613402.2008 CCAAGATTGATCGAGGTTGGGTATGTGTTTTTCAGCTTCAAGATGGTAAAACACTTGGAC 4059

KC242789.2007 CCAAGATTGATCGAGGTTGGGTATGTGTTTTTCAGCTTCAAGATGGTAAAACACTTGGAC 4140

HQ613403.2007 CCAAGATTGATCGAGGTTGGGTATGTGTTTTTCAGCTTCAAGATGGTAAAACACTTGGAC 4092

KC242785.2007 CCAAGATTGATCGAGGTTGGGTATGTGTTTTTCAGCTTCAAGATGGTAAAACACTTGGAC 4140

KC242790.2007 CCAAGATTGATCGAGGTTGGGTATGTGTTTTTCAGCTTCAAGATGGTAAAACACTTGGAC 4140

KU143789.2014 CCAAGATTGATCGAGGTTGGGTATGTGTTTTTCAGCTTCAAGATGGTAAAACACTTGGAC 4140

KR817168.2014 CCAAGATTGATCGAGGTTGGGTATGTGTTTTTCAGCTTCAAGATGGTAAAACACTTGGAC 4114

KY426696.2015 CCAAGATTGATCGAGGTTGGGTATGTGTTTTTCAGCTTCAAGATGGTAAAACACTTGGAC 4140

KR105271.2014 CCAAGATTGATCGAGGTTGGGTATGTGTTTTTCAGCTTCAAGATGGTAAAACACTTGGAC 4107

KY007522.2016 CCAAGATTGATCGAGGTTGGGTATGTGTTTTTCAGCTTCAAGATGGTAAAACACTTGGAC 4120

KM034555.2014 CCAAGATTGATCGAGGTTGGGTATGTGTTTTTCAGCTTCAAGATGGTAAAACACTTGGAC 4132

MH470381.2015 CCAAGATTGATCGAGGTTGGGTATGTGTTTTTCAGCTTCAAGATGGTAAAACACTTGGAC 4104

MH470382.2015 CCAAGATTGATCGAGGTTGGGTATGTGTTTTTCAGCTTCAAGATGGTAAAACACTTGGAC 4104

MF102255.2014 CCAAGATTGATCGAGGTTGGGTATGTGTTTTTCAGCTTCAAGATGGTAAAACACTTGGAC 4117

KJ660348.2014 CCAAGATTGATCGAGGTTGGGTATGTGTTTTTCAGCTTCAAGATGGTAAAACACTTGGAC 4140

KU143818.2014 CCAAGATTGATCGAGGTTGGGTATGTGTTTTTCAGCTTCAAGATGGTAAAACACTTGGAC 4140

KT725333.2014 CCAAGATTGATCGAGGTTGGGTATGTGTTTTTCAGCTTCAAGATGGTAAAACACTTGGAC 4105

KR819004.2014 CCAAGATTGATCGAGGTTGGGTATGTGTTTTTCAGCTTCAAGATGGTAAAACACTTGGAC 4087

KP271020.2014 CCAAGATTGATCGAGGTTGGGTATGTGTTTTTCAGCTTCAAGATGGTAAAACACTTGGAC 4087

KM519951.2014 CCAAGATTGATCGAGGTTGGGTATGTGTTTTTCAGCTTCAAGATGGTAAAACACTTGGAC 4137

MH733488.2018 CCAAGATTGATCGAGGTTGGGTATGTGTTTTTCAGCTTCAAGATGGTAAAACACTTGGAC 4131

MH733491.2018 CCAAGATTGATCGAGGTTGGGTATGTGTTTTTCAGCTTCAAGATGGTAAAACACTTGGAC 4126

MH733478.2018 CCAAGATTGATCGAGGTTGGGTATGTGTTTTTCAGCTTCAAGATGGTAAAACACTTGGAC 4129

MK007330.2018 CCAAGATTGATCGAGGTTGGGTATGTGTTTTTCAGCTTCAAGATGGTAAAACACTTGGAC 4128

MK007344.2018 CCAAGATTGATCGAGGTTGGGTATGTGTTTTTCAGCTTCAAGATGGTAAAACACTTGGAC 4122

******************************* ****************************

AY142960.1976 TCAAAATTTGAGCCAATCTCCCTTCCCTCCGAAAGAGGCGAATAATAGCAGAGGCTTCAA 4200 end VP35

KC242791.1977 TCAAAATTTGAGCCAATCTCCCTTCCCTCCGAAAGAGGCGAATAATAGCAGAGGCTTCAA 4200

AF499101.1976 TCAAAATTTGAGCCAATCTCCCTTCCCTCCGAAAGAGGCGAATAATAGCAGAGGCTTCAA 4200

KF113528.2003 TCAAAATTTGAGCCAATCTCCCTTCCCTCCGAAAGAGGCGACCAATAGCAGAGGCTTCAA 4196

KC242800.2002 TCAAAATTTGAGCCAATCTCCCTTCCCTCCGAAAGAGGCGACCAATAGCAGAGGCTTCAA 4200

KY471090.2001 TCAAAATTTGAGCCAATCTCCCTTCCCTCCGAAAGAGGCGACCAATAGCAGAGGCTTCAA 4177

KY471092.2001 TCAAAATTTGAGCCAATCTCCCTTCCCTCCGAAAGAGGCGACCAATAGCAGAGGCTTCAA 4177

MH481611.2017 TCAAAATTTGAGCCAATCTCCCTTCCCTCCGAAAGAGGCGACTAATAGCAGAGGCTTCAA 4166

MH613311.2017 TCAAAATTTGAGCCAATCTCCCTTCCCTCCGAAAGAGGCGACTAATAGCAGAGGCTTCAA 4145

KC242792.1994 TCAAAATTTGAGCCAATCTCCCTTCCCTCCGAAAGAGGCGACTAATAGCAGAGGCTTCAA 4200

KC242793.1996 TCAAAATTTGAGCCAATCTCCCTTCCCTCCGAAAGAGGCGACTAATAGCAGAGGCTTCAA 4200

KC242794.1996 TCAAAATTTGAGCCAATCTCCCTTCCCTCCGAAGGAGGCGACTAATAGCAGAGGCTTCAA 4200

MH121164.1995 TCAAAATTTGAGCCCATCTTCCTTCCCTCCGAAAGAGGCGACTAATAGCAGAGGCTTCAA 4200

AY354458.1995 TCAAAATTTGAGCCCATCTTCCTTCCCTCCGAAAGAGGCGACTAATAGCAGAGGCTTCAA 4200

KT762962.1995 TCAAAATTTGAGCCCATCTTCCTTCCCTCCGAAAGAGGCGACTAATAGCAGAGGCTTCAA 4200

HQ613402.2008 TCAAAATTTGAGCCAATCTCCCTTCCCTCCGAACGAGGCGACTAATAGCAGAGGCTTCAA 4119

KC242789.2007 TCAAAATTTGAGCCAATCTCCCTTCCCTCCGAACGAGGCGACTAATAGCAGAGGCTTCAA 4200

HQ613403.2007 TCAAAATTTGAGCCAATCTCCCTTCCCTCCGAACGAGGCGACTAATAGCAGAGGCTTCAA 4152

KC242785.2007 TCAAAATTTGAGCCAATCTCCCTTCCCTCCGAACGAGGCGACTAATAGCAGAGGCTTCAA 4200

KC242790.2007 TCAAAATTTGAGCCAATCTCCCTTCCCTCCGAACGAGGCGACTAATAGCAGAGGCTTCAA 4200

KU143789.2014 TCAAAATTTGAGCCAATCTCTTTTCCCTCCGAAAGAGGCAACTAATAGCAGAGGCTTCAA 4200

KR817168.2014 TCAAAATTTGAGCCAATCTCTTTTCCCTCCGAAAGAGGCAACTAATAGCAGAGGCTTCAA 4174

KY426696.2015 TCAAAATTTGAGCCAATCTCTTTTCCCTCCGAAAGAGGCAACTAACAGCAGAGGCTTCAA 4200

KR105271.2014 TCAAAATTTGAGCCAATCTCTTTTCCCTCCGAAAGAGGCAACTAATAGCAGAGGCTTCAA 4167

KY007522.2016 TCAAAATTTGAGCCAATCTCTTTTCCCTCCGAAAGAGGCAACTAATAGCAGAGGCTTCAA 4180

KM034555.2014 TCAAAATTTGAGCCAATCTCTTTTCCCTCCGAAAGAGGCAACTAATAGCAGAGGCTTCAA 4192

MH470381.2015 TCAAAATTTGAGCCAATCTCTTTTCCCTCCGAAAGAGGCAACTAATAGCAGAGGCTTCAA 4164

MH470382.2015 TCAAAATTTGAGCCAATCTCTTTTCCCTCCGAAAGAGGCAACTAATAGCAGAGGCTTCAA 4164

MF102255.2014 TCAAAATTTGAGCCAATCTCTTTTCCCTCCGAAAGAGGCAACTAATAGCAGAGGCTTCAA 4177

KJ660348.2014 TCAAAATTTGAGCCAATCTCTTTTCCCTCCGAAAGAGGCAACTAATAGCAGAGGCTTCAA 4200

KU143818.2014 TCAAAATTTGAGCCAATCTCTTTTCCCTCCGAAAGAGGCAACTAATAGCAGAGGCTTCAA 4200

KT725333.2014 TCAAAATTTGAGCCAATCTCTTTTCCCTCCGAAAGAGGCAACTAATAGCAGAGGCTTCAA 4165

KR819004.2014 TCAAAATTTGAGCCAATCTCCCTTCCCTCCGAAAGAGGCGACTAATAGCAGAGGCTTCAA 4147

KP271020.2014 TCAAAATTTGAGCCAATCTCCCTTCCCTCCGAAAGAGGCGACTAATAGCAGAGGCTTCAA 4147

KM519951.2014 TCAAAATTTGAGCCAATCTCCCTTCCCTCCGAAAGAGGCGACTAATAGCAGAGGCTTCAA 4197

MH733488.2018 TCAAAATTTGAGCCAATCTCCCTTCCCTCCGAAAGAGGCGACTAATAGCAGAGGCTTCAA 4191

MH733491.2018 TCAAAATTTGAGCCAATCTCCCTTCCCTCCGAAAGAGGCGACTAATAGCAGAGGCTTCAA 4186

MH733478.2018 TCAAAATTTGAGCCAATCTCCCTTCCCTCCGAAAGAGGCGACTAATAGCAGAGGCTTCAA 4189

MK007330.2018 TCAAAATTTGAGCCAATCTCCCTTCCCTCCGAAAGAGGCGACTAATAGCAGAGGCTTCAA 4188

MK007344.2018 TCAAAATTTGAGCCAATCTCCCTTCCCTCCGAAAGAGGCGACTAATAGCAGAGGCTTCAA 4182

************** **** *********** ***** * ** **************

AY142960.1976 CTGCTGAACTATAGGGTACGTTACATTAATGATACACTTGTGAGTATCAGCCCTGGATAA 4260

KC242791.1977 CTGCTGAACTATAGGGTACGTTACATTAATGATACACTTGTGAGTATCAGCCCTGGATAA 4260

AF499101.1976 CTGCTGAACTATAGGGTACGTTACATTAATGATACACTTGTGAGTATCAGCCCTGGATAA 4260

KF113528.2003 CTGCTGAACTACAGGGTACGTTACATTAATGATACACTTGTGAGTATCAGCCCTAGATAA 4256

KC242800.2002 CTGCTGAACTACAGGGTACGTTACATTAATGATACACTTGTGAGTATCAGCCCTAGATAA 4260

KY471090.2001 CTGCTGAACTACAGGGTACGTTACATTAATGATACACTTGTGAGTATCAGCCCTAGATAA 4237

KY471092.2001 CTGCTGAACTACAGGGTACGTTACATTAATGATACACTTGTGAGTATCAGCCCTAGATAA 4237

MH481611.2017 CTGCTGAACTATAGGATACGTTACATTAATGATACACTGGTGAGTATCAGCCCTGGATAA 4226

MH613311.2017 CTGCTGAACTATAGGATACGTTACATTAATGATACACTGGTGAGTATCAGCCCTGGATAA 4205

KC242792.1994 CTGCTGAACTATAGGGTACGTTACATTAATGATACACTTTTGAGTATCAGCCCTGGATAA 4260

KC242793.1996 CTGCTGAACTATAGGGTACGTTACATTAATGATACACTTGTGAGTATCAGCCCTGGATAA 4260

KC242794.1996 CTGCTGAACTATAGGGTACGTTACATTAATGATACACTTGTGAGTATTAGCCCTGGATAA 4260

MH121164.1995 CTGCTGAACTATAGGGTACGTTACATTAATGATACACTTGTGAGTATCAGCCCTGGATAA 4260

AY354458.1995 CTGCTGAACTATAGGGTACGTTACATTAATGATACACTTGTGAGTATCAGCCCTGGATAA 4260

KT762962.1995 CTGCTGAACTATAGGGTACGTTACATTAATGATACACTTGTGAGTATCAGCCCTGGATAA 4260

HQ613402.2008 CTGCTGAACTATAGGGTACGTTACATTAATGATACACTTGTGAGCATCAGCCCTAAATAA 4179

KC242789.2007 CTGCTGAACTATAGGGTACGTTACATTAATGATACACTTGTGAGCATCAGCCCTAAATAA 4260

HQ613403.2007 CTGCTGAACTATAGGGTACGTTACATTAATGATACACTTGTGAGCATCAGCCCTAAATAA 4212

KC242785.2007 CTGCTGAACTATAGGGTACGTTACATTAATGATACACTTGTGAGCATCAGCCCTAAATAA 4260

KC242790.2007 CTGCTGAACTATAGGGTACGTTACATTAATGATACACTTGTGAGCATCAGCCCTAAATAA 4260

KU143789.2014 CTGCTGAACTATAGGGTATGTTACATTAATGATACACTTGTGAGTATCAGCCCTAGATAA 4260

KR817168.2014 CTGCTGAACTATAGGGTATGTTACATTAATGATACACTTGTGAGTATCAGCCCTAGATAA 4234

KY426696.2015 CTGCTGAACTATAGGGTATGCTACATCAATGATACACTTGTGAGTATCAGCCCTAGATAA 4260

KR105271.2014 CTGCTGAACTATAGGGTATGTTACATTAATGATACACTTGTGAGTATCAGCCCTAGATAA 4227

KY007522.2016 CTGCTGAACTATAGGGTATGTTACATTAATGATACACTTGTGAGTATCAGCCCTAGATAA 4240

KM034555.2014 CTGCTGAACTATAGGGTATGTTACATTAATGATACACTTGTGAGTATCAGCCCTAGATAA 4252

MH470381.2015 CTGCTGAACTATAGGGTATGTTACATTAATGATACACTTGTGAGTATCAGCCCTAGATAA 4224

MH470382.2015 CTGCTGAACTATAGGGTATGTTACATTAATGATACACTTGTGAGTATCAGCCCTAGATAA 4224

MF102255.2014 CTGCTGAACTATAGGGTATGTTACATTAATGATACACTTGTGAGTATCAGCCCTAGATAA 4237

KJ660348.2014 CTGCTGAACTATAGGGTATGTTACATTAATGATACACTTGTGAGTATCAGCCCTAGATAA 4260

KU143818.2014 CTGCTGAACTATAGGGTATGTTACATTAATGATACACTTGTGAGTATCAGCCCTAGATAA 4260

KT725333.2014 CTGCTGAACTATAGGGTATGTTACATTAATGATACACTTGTGAGTATCAGCCCTAGATAA 4225

KR819004.2014 CTGCTGAACTATAGGGTACGTTACATTAATGATACACTTGTGAGTATCAGCCCTGGATAA 4207

KP271020.2014 CTGCTGAACTATAGGGTACGTTACATTAATGATACACTTGTGAGTATCAGCCCTGGATAA 4207

KM519951.2014 CTGCTGAACTATAGGGTACGTTACATTAATGATACACTTGTGAGTATCAGCCCTGGATAA 4257

MH733488.2018 CTGCTGAACTATAGGGTACGTTACATTAATGATACACTTGTGAGTATCAGCCCTGGATAA 4251

MH733491.2018 CTGCTGAACTATAGGGTACGTTACATTAATGATACACTTGTGAGTATCAGCCCTGGATAA 4246

MH733478.2018 CTGCTGAACTATAGGGTACGTTACATTAATGATACACTTGTGAGTATCAGCCCTGGATAA 4249

MK007330.2018 CTGCTGAACTATAGGATATGTTACATTAATGATACACTTGTGAGTATCAGCCCTGGATAA 4248

MK007344.2018 CTGCTGAACTATAGGATATGTTACATTAATGATACACTTGTGAGTATCAGCCCTGGATAA 4242

*********** *** ** * ***** *********** **** ** ****** ****

AY142960.1976 TATAAGTCAATTAAACGACCAAGATAAAATTGTTCATATCTCGCTAGCAGCTTAAAATAT 4320

KC242791.1977 TATAAGTCAATTAAACGACCAAGATAAAATTGTTCATATCTCGCTAGCAGCTTAAAATAT 4320

AF499101.1976 TATAAGTCAATTAAACGACCAAGATAAAATTGTTCATATCTCGCTAGCAGCTTAAAATAT 4320

KF113528.2003 TATAAGTCAATTAAACGACCAAGCTAAAATTGTTCATATCCCGCTAGCAGCTTAAAATAT 4316

KC242800.2002 TATAAGTCAATTAAACGACCAAGCCAAAATTGTTCATATCCCGCTAGCAGCTTAAAATAT 4320

KY471090.2001 TATAAGTCAATTAAACGACCAAGCCAAAATTGTTCATATCCCGCTAGCAGCTTAAAATAT 4297

KY471092.2001 TATAAGTCAATTAAACGACCAAGCCAAAATTGTTCATATCCCGCTAGCAGCTTAAAATAT 4297

MH481611.2017 TATAAGTCAATTAAACGACCAAGATAAAATTGCTCATATCTCGCTAGCAGCTTAAAGCAT 4286

MH613311.2017 TATAAGTCAATTAAACGACCAAGATAAAATTGCTCATATCTCGCTAGCAGCTTAAAGCAT 4265

KC242792.1994 TATAAGTCAATTAAACGACCAAGATAAAATTGTCCTTATCTCGCTAGCAGCTTAAAATAT 4320

KC242793.1996 TATAAGTCAATTAAACGACCAAGATAAAATTGTCCTTATCTCGCTAGCAGCTTAAAATAT 4320

KC242794.1996 TATAAGTCAATTAAACGACCAAGATAAAATTGTCCTTATCTCGCTAGCAGCTTAAAATAT 4320

MH121164.1995 TATAAGTCAATTAAACGACCAAGATAAAATTGTTCTTATCTCGCTAGCAGCTTAAAATAT 4320

AY354458.1995 TATAAGTCAATTAAACGACCAAGATAAAATTGTTCTTATCTCGCTAGCAGCTTAAAATAT 4320

KT762962.1995 TATAAGTCAATTAAACGACCAAGATAAAATTGTTCTTATCTCGCTAGCAGCTTAAAATAT 4320

HQ613402.2008 TATAAGTCAATTAAACGACCAAGATAAAATTGTTCATATCCCGCTAGCAGCTTAAAATAT 4239

KC242789.2007 TATAAGTCAATTAAACGACCAAGATAAAATTGTTCATATCCCGCTAGCAGCTTAAAATAT 4320

HQ613403.2007 TATAAGTCAATTAAACGACCAAGATAAAATTGTTCATATCCCGCTAGCAGCTTAAAATAT 4272

KC242785.2007 TATAAGTCAATTAAACGACCAAGATAAAATTGTTCATATCCCGCTAGCAGCTTAAAATAT 4320

KC242790.2007 TATAAGTCAATTAAACGACCAAGATAAAATTGTTCATATCCCGCTAGCAGCTTAAAATAT 4320

KU143789.2014 TATAAGTCAATTAAACAACCAAGATAAAATTATTCATATCCCGCTAGCAGCTTTAAAGAT 4320

KR817168.2014 TATAAGTCAATTAAACAACCAAGATAAAATTGTTCATATCCCGCTAGCAGCTTTAAAGAT 4294

KY426696.2015 TATAAGTCAATTAAACAACCAAGATAAAATTGTTCATATCCCGCTAGCAGCTTTAAAGAT 4320

KR105271.2014 TATAAGTCAATTAAACAACCAAGATAAAATTGTTCATATCCCGCTAGCAGCTTTAAAGAT 4287

KY007522.2016 TATAAGTCAATTAAACAACCAAGATAAAATTGTTCATATCCCGCTAGCAGCTTTAAAGAT 4300

KM034555.2014 TATAAGTCAATTAAACAACCAAGATAAAATTGTTCATATCCCGCTAGCAGCTTTAAAGAT 4312

MH470381.2015 TATAAGTCAATTAAACAACCAAGATAAAATTGTTCATATCCCGCTAGCAGCTTTAAAGAT 4284

MH470382.2015 TATAAGTCAATTAAACAACCAAGATAAAATTGTTCATATCCCGCTAGCAGCTTTAAAGAT 4284

MF102255.2014 TATAAGTCAATTAAACAACCAAGATAAAATTGTTCATATCCCGCTAGCAGCTTTAAAGAT 4297

KJ660348.2014 TATAAGTCAATTAAACAACCAAGATAAAATTGTTCATATCCCGCTAGCAGCTTTAAAGAT 4320

KU143818.2014 TATAAGTCAATTAAACAACCAAGATAAAATTGTTCATATCCCGCTAGCAGCTTTAAAGAT 4320

KT725333.2014 TATAAGTCAATTAAACAACCAAGATAAAATTGTTCATATCCCGCTAGCAGCTTTAAAGAT 4285

KR819004.2014 TATAAGTCAATCAAACGACCAAGATAAAATTGTTCTTATCTCGCTAGCAGCTTAAAATAT 4267

KP271020.2014 TATAAGTCAATCAAACGACCAAGATAAAATTGTTCTTATCTCGCTAGCAGCTTAAAATAT 4267

KM519951.2014 TATAAGTCAATCAAACGACCAAGATAAAATTGTTCTTATCTCGCTAGCAGCTTAAAATAT 4317

MH733488.2018 TATAAGTCAATCAAACGACCAAGATAAAATTGTTCTTATCTCGCTAGCAGCTTAAAATAT 4311

MH733491.2018 TATAAGTCAATCAAACGACCAAGATAAAATTGTTCTTATCTCGCTAGCAGCTTAAAATAT 4306

MH733478.2018 TATAAGTCAATCAAACGACCAAGATAAAATTGTTCTTATCTCGCTAGCAGCTTAAAATAT 4309

MK007330.2018 TATAAGTCAATTAAACGACTAAGATAAAATTGTTCATATCTCGCTAGCAGCTTAAAATAT 4308

MK007344.2018 TATAAGTCAATTAAACGACTAAGGTAAAATTGTTCATATCTCGCTAGCAGCTTAAAATAT 4302

*********** **** ** *** ****** * **** ************ ** **

AY142960.1976 AAATGTAATAGGAGCTATATCTCTGACAGTATTATAATCAATTGTTATTAAGTAACCCAA 4380

KC242791.1977 AAATGTAATAGGAGCTATATCTCTGACAGTATTATAATCAATTGTTATTAAGTAACCCAA 4380

AF499101.1976 AAATGTAATAGGAGCTATATCTCTGACAGTATTATAATCAATTGTTATTAAGTAACCCAA 4380

KF113528.2003 AAATGAAATAGGAGCTATATCTCTGACAGTATTATAATCAATTGTTATTAAGTAACCCAA 4376

KC242800.2002 AAATGAAATAGGAGCTATATCTCTGACAGTATTATAATCAATTGTTATTAAGTAACCCAA 4380

KY471090.2001 AAATGAAATAGGAGCTATATCTCTGACAGTATTATAATCAATTGTTATTAAGTAACCCAA 4357

KY471092.2001 AAATGAAATAGGAGCTATATCTCTGACAGTATTATAATCAATTGTTATTAAGTAACCCAA 4357

MH481611.2017 AAATGTAATAGAAGCTATATCTCTGACAGTATTATAATTGATTGTTATTAAGTAACCCAA 4346

MH613311.2017 AAATGTAATAGAAGCTATATCTCTGACAGTATTATAATTGATTGTTATTAAGTAACCCAA 4325

KC242792.1994 AAATGTAATAGGAGCTATATCTCTGACAGTATTATAATCAATTGTTATTAAGTAACCCAA 4380

KC242793.1996 AAATGTAATAGGAGCTATATCTCTGACAGTATTATAATCAATTGTTATTAAGTAACCCAA 4380

KC242794.1996 AAATGTAATAGGAGCTATATCTCTGACAGTATTATAATCAATTGTTATTAAGTAACCCAA 4380

MH121164.1995 GAATGTAATAGGAGCTATATCTCTGACAGTATTATAATCAATCGTTATTAAGTAACCCAA 4380

AY354458.1995 GAATGTAATAGGAGCTATATCTCTGACAGTATTATAATCAATCGTTATTAAGTAACCCAA 4380

KT762962.1995 GAATGTAATAGGAGCTATATCTCTGACAGTATTATAATCAATCGTTATTAAGTAACCCAA 4380

HQ613402.2008 AAATGTAATAGGAGCTATATCTGTGACAGTATTATAATCAATTGTTATTAAGTAACCCAA 4299

KC242789.2007 AAATGTAATAGGAGCTATATCTGTGACAGTATTATAATCAATTGTTATTAAGTAACCCAA 4380

HQ613403.2007 AAATGTAATAGGAGCTATATCTGTGACAGTATTATAATCAATTGTTATTAAGTAACCCAA 4332

KC242785.2007 AAATGTAATAGGAGCTATATCTGTGACAGTATTATAATCAATTGTTATTAAGTAACCCAA 4380

KC242790.2007 AAATGTAATAGGAGCTATATCTGTGACAGTATTATAATCAATCGTTATTAAGTAACCCAA 4380

KU143789.2014 AAATGTAATAGGAGCTATACCTCTGACAGTATTATAATTAATTGTTATTAAGTAACCCAA 4380

KR817168.2014 AAATGTAATAGGAGCTATACCTCTGACAGTATTATAATTAATTGTTATTAAGTAACCCAA 4354

KY426696.2015 AAATGTAATAGGAGCTATACCTCTGACAGTATTATAATTAATTGTTATTAAGTAACCCAA 4380

KR105271.2014 AAATGTAATAGGAGCTATACCTCTGACAGTATTATAATTAATTGTTATTAAGTAACCCAA 4347

KY007522.2016 AAATGTAATAGGAGCTATACCTCTGACAGTATTATAATTAATTGTTATTAAGTAACCCAA 4360

KM034555.2014 AAATGTAATAGGAGCTATACCTCTGACAGTATTATAATTAATTGTTATTAAGTAACCCAA 4372

MH470381.2015 AAATGTAATAGGAGCTATACCTCTGACAGTATTATAATTAATTGTTATTAAGTAACCCAA 4344

MH470382.2015 AAATGTAATAGGAGCTATACCTCTGACAGTATTATAATTAATTGTTATTAAGTAACCCAA 4344

MF102255.2014 AAATGTAATAGGAGCTATACCTCTGACAGTATTATAATTAATTGTTATTAAGTAACCCAA 4357

KJ660348.2014 AAATGTAATAGGAGCTATACCTCTGACAGTATTATAATTAATTGTTATTAAGTAACCCAA 4380

KU143818.2014 AAATGTAATAGGAGCTATACCTCTGACAGTATTATAATTAATTGTTATTAAGTAACCCAA 4380

KT725333.2014 AAATGTAATAGGAGCTATACCTCTGACAGTATTATAATTAATTGTTATTAAGTAACCCAA 4345

KR819004.2014 AAATGTAATAGGAGCTATATCTCTGACAGTATTATAATCAATTGTTATTAAGTAACCCAA 4327

KP271020.2014 AAATGTAATAGGAGCTATATCTCTGACAGTATTATAATCAATTGTTATTAAGTAACCCAA 4327

KM519951.2014 AAATGTAATAGGAGCTATATCTCTGACAGTATTATAATCAATTGTTATTAAGTAACCCAA 4377

MH733488.2018 AGATGTAATAGGAGCTATATCTCTGACAGTATTATAATCAATTGTTATTAAGTAACCCAA 4371

MH733491.2018 AGATGTAATAGGAGCTATATCTCTGACAGTATTATAATCAATTGTTATTAAGTAACCCAA 4366

MH733478.2018 AGATGTAATAGGAGCTATATCTCTGACAGTATTATAATCAATTGTTATTAAGTAACCCAA 4369

MK007330.2018 AAATGTAATAGGAGCTATATCTCTGACAGTATTACAAATGATTGTTATTAGGTAACCCAA 4368

MK007344.2018 AAATGTAATAGGAGCTATATCTCTGACAGTATTACAAATGATTGTTATTAGGTAACCCAA 4362

*** ***** ******* ** *********** ** ** ******* *********

AY142960.1976 ACCAAAAGTGATGAAGATTAAGAAAAACCTACCTCGGCTGAGAGAGTGTTTTTTCATTAA 4440 EBOV-miR-T2-3p

KC242791.1977 ACCAAAAGTGATGAAGATTAAGAAAAACCTACCTCGGCTGAGAGAGTGTTTTTTCATTAA 4440

AF499101.1976 ACCAAAAGTGATGAAGATTAAGAAAAACCTACCTCGGCTGAGAGAGTGTTTTTTCATTAA 4440

KF113528.2003 ACCAAAAATGATGAAGATTAAGAAAAACCTACCTCGACTGAGAGAGTGTTTTTTCATTAA 4436

KC242800.2002 ACCAAAAATGATGAAGATTAAGAAAAACCTACCTCGACTGAGAGAGTGTTTTTCCATTAA 4440

KY471090.2001 ACCAAAAATGATGAAGATTAAGAAAAACCTACCTCGACTGAGAGAGTGTTTTTCCATTAA 4417

KY471092.2001 ACCAAAAATGATGAAGATTAAGAAAAACCTACCTCGACTGAGAGAGTGTTTTTCCATTAA 4417

MH481611.2017 ACCAAAAGTGATGAAGATTAAGAAAAACCTACCTCGACTGAGAGAGTGTTTTTTCATTAA 4406

MH613311.2017 ACCAAAAGTGATGAAGATTAAGAAAAACCTACCTCGACTGAGAGAGTGTTTTTTCATTAA 4385

KC242792.1994 ACCAAAAGTGATGAAGATTAAGAAAAACCTACCTCGACTGAGAGAGTGTTTTTCCATTAA 4440

KC242793.1996 ACCAAAAGTGATGAAGATTAAGAAAAACCTACCTCGACTGAGAGAGTGTTTTTCCATTGA 4440

KC242794.1996 ACCAAAAGTGATGAAGATTAAGAAAAACCTACCTCGACTGAGAGAGTGTTTTTCCATTAA 4440

MH121164.1995 ACCAAAAGTGATGAAGATTAAGAAAAACCTACCTCGACTGAGAGAGTGTTTTTTCATTAA 4440

AY354458.1995 ACCAAAAGTGATGAAGATTAAGAAAAACCTACCTCGACTGAGAGAGTGTTTTTTCATTAA 4440

KT762962.1995 ACCAAAAGTGATGAAGATTAAGAAAAACCTACCTCGACTGAGAGAGTGTTTTTTCATTAA 4440

HQ613402.2008 ACCAAAAATGATGAAGATTAAGAAAAACCTACCTCGACTGAGAGAGTGTTTTTCCATTAA 4359

KC242789.2007 ACCAAAAATGATGAAGATTAAGAAAAACCTACCTCGACTGAGAGAGTGTTTTTCCATTAA 4440

HQ613403.2007 ACCAAAAATGATGAAGATTAAGAAAAACCTACCTCGACTGAGAGAGTGTTTTTCCATTAA 4392

KC242785.2007 ACCAAAAATGATGAAGATTAAGAAAAACCTACCTCGACTGAGAGAGTGTTTTTCCATTAA 4440

KC242790.2007 ACCAAAAATGATGAAGATTAAGAAAAACCTACCTCGACTGAGAGAGTGTTTTTCCATTAA 4440

KU143789.2014 ACCAAAAATGATGAAGATTAAGAAAAACCTACCTCGACTGAGAGAGTGTTTTTTCATTAA 4440

KR817168.2014 ACCAAAAATGATGAAGATTAAGAAAAACCTACCTCGACTGAGAGAGTGTTTTTTCATTAA 4414

KY426696.2015 ACCAAAAATGATGAAGATTAAGAAAAACCTACCTCGACTGAGAGAGTGTTTTTTCATTAA 4440

KR105271.2014 ACCAAAAATGATGAAGATTAAGAAAAACCTACCTCGACTGAGAGAGTGTTTTTTCATTAA 4407

KY007522.2016 ACCAAAAAAGATGAAGATTAAGAAAAACCTACCTCGACTGAGAGAGTGTTTTTTCATTAA 4420

KM034555.2014 ACCAAAAATGATGAAGATTAAGAAAAACCTACCTCGACTGAGAGAGTGTTTTTTCATTAA 4432

MH470381.2015 ACCAAAAATGATGAAGATTAAGAAAAACCTACCTCGACTGAGAGAGTGTTTTTTCATTAA 4404

MH470382.2015 ACCAAAAATGATGAAGATTAAGAAAAACCTACCTCGACTGAGAGAGTGTTTTTTCATTAA 4404

MF102255.2014 ACCAAAAATGATGAAGATTAAGAAAAACCTACCTCGACTGAGAGAGTGTTTTTTCATTAA 4417

KJ660348.2014 ACCAAAAATGATGAAGATTAAGAAAAACCTACCTCGACTGAGAGAGTGTTTTTTCATTAA 4440

KU143818.2014 ACCAAAAATGATGAAGATTAAGAAAAACCTACCTCGACTGAGAGAGTGTTTTTTCATTAA 4440

KT725333.2014 ACCAAAAATGATGAAGATTAAGAAAAACCTACCTCGACTGAGAGAGTGTTTTTTCATTAA 4405

KR819004.2014 ACCAAAAGTGATGAAGATTAAGAAAAACCTACCTCGACTGAGAGAGTGTTTTTTCATTAA 4387

KP271020.2014 ACCAAAAGTGATGAAGATTAAGAAAAACCTACCTCGACTGAGAGAGTGTTTTTTCATTAA 4387

KM519951.2014 ACCAAAAGTGATGAAGATTAAGAAAAACCTACCTCGACTGAGAGAGTGTTTTTTCATTAA 4437

MH733488.2018 ACCAAAAGTGATGAAGATTAAGAAAAACCTACCTCGACTGAGAGAGTGTTTTTTCATTAA 4431

MH733491.2018 ACCAAAAGTGATGAAGATTAAGAAAAACCTACCTCGACTGAGAGAGTGTTTTTTCATTAA 4426

MH733478.2018 ACCAAAAGTGATGAAGATTAAGAAAAACCTACCTCGACTGAGAGAGTGTTTTTTCATTAA 4429

MK007330.2018 ACCAAAAGTGATGAAGATTAAGAAAAACCTACCTCGACTGAGAGAGTGTTTTTTCATTAA 4428

MK007344.2018 ACCAAAAGTGATGAAGATTAAGAAAAACCTACCTCGACTGAGAGAGTGTTTTTTCATTAA 4422

******* *************************** **************** **** *

AY142960.1976 CCTTCATCTTGTAAACGTTGAGCAAAATTGTTAAAAATATGAGGCGGGTTATATTGCCTA 4500 start VP40

KC242791.1977 CCTTCATCTTGTAAACGTTGAGCAAAATTGTTAAAAATATGAGGCGGGTTATATTGCCTA 4500

AF499101.1976 CCTTCATCTTGTAAACGTTGAGCAAAATTGTTAAAAATATGAGGCGGGTTATATTGCCTA 4500

KF113528.2003 CCTTCATCCTGTAAACGTTGAGCAAAATTGTTAAGAATATGAGGCGGGTTATATTGCCTA 4496

KC242800.2002 CCTTCATCTTGTAAACGTTGAGCAAAATTGTTACGAATATGAGGCGGGTTATATTGCCTA 4500

KY471090.2001 CCTTCATCTTGTAAACGTTGAGCAAAATTGTTAAGAATATGAGGCGGGTTATATTGCCTA 4477

KY471092.2001 CCTTCATCTTGTAAACGTTGAGCAAAATTGTTAAGAATATGAGGCGGGTTATATTGCCTA 4477

MH481611.2017 TCTTCATCTTGTAAACGTTGAGCAAAATTGTTAAAAATATGAGGCGGGTTATATTGCCTA 4466

MH613311.2017 TCTTCATCTTGTAAACGTTGAGCAAAATTGTTAAAAATATGAGGCGGGTTATATTGCCTA 4445

KC242792.1994 CCTTCATCTTGTAAACGTTGAGCAAAATTGTTAAAAATATGAGGCGGGTTATATTGCCTA 4500

KC242793.1996 CCTTCATCTTGTAAACGTTGAGCAAAATTGTTAAAAATATGAGGCGGGTTATATTGCCTA 4500

KC242794.1996 CCTTCATCTTGTAAACGTTGAGCAAAATTGTTAAAAATATGAGGCGGGTTATATTGCCTA 4500

MH121164.1995 CCTTCATCTTGTAAACGTTGAGCAAAATTGTTAAAAATATGAGGCGGGTTATATTACCTA 4500

AY354458.1995 CCTTCATCTTGTAAACGTTGAGCAAAATTGTTAAAAATATGAGGCGGGTTATATTACCTA 4500

KT762962.1995 CCTTCATCTTGTAAACGTTGAGCAAAATTGTTAAAAATATGAGGCGGGTTATATTACCTA 4500

HQ613402.2008 CCTTCATCTTGTAAACGTTGAGCAAAATTGTTAAAAATATGAGGCGGGTTATATTGCCTA 4419

KC242789.2007 CCTTCATCTTGTAAACGTTGAGCAAAATTGTTAAAAATATGAGGCGGGTTATATTGCCTA 4500

HQ613403.2007 CCTTCATCTTGTAAACGTTGAGCAAAATTGTTAAAAATATGAGGCGGGTTATATTGCCTA 4452

KC242785.2007 CCTTCATCTTGTAAACGTTGAGCAAAATTGTTAAAAATATGAGGCGGGTTATATTGCCTA 4500

KC242790.2007 CCTTCATCTTGTAAACGTTGAGCAAAATTGTTAAAAATATGAGGCGGGTTATATTGCCTA 4500

KU143789.2014 CCTTCATCTTGTAAACGTTGAGCAAAATTGTTAAAAATATGAGGCGGGTTATATTGCCTA 4500

KR817168.2014 CCTTCATCTTGTAAACGTTGAGCAAAATTGTTAAAAATATGAGGCGGGTTATATTGCCTA 4474

KY426696.2015 CCTTCATCTTGTAAACGTTGAGCAAAATTGTTAAAAATATGAGGCGGGTTATATTGCCTA 4500

KR105271.2014 CCTTCATCTTGTAAACGTTGAGCAAAATTGTTAAAAATATGAGGCGGGTTATATTGCCTA 4467

KY007522.2016 CCTTCATCTTGTAAACGTTGAGCAAAATTGTTAAAAATATGAGGCGGGTTATATTGCCTA 4480

KM034555.2014 CCTTCATCTTGTAAACGTTGAGCAAAATTGTTAAAAATATGAGGCGGGTTATATTGCCTA 4492

MH470381.2015 CCTTCATCTTGTAAACGTTGAGCAAAATTGTTAAAAATATGAGGCGGGTTATATTGCCTA 4464

MH470382.2015 CCTTCATCTTGTAAACGTTGAGCAAAATTGTTAAAAATATGAGGCGGGTTATATTGCCTA 4464

MF102255.2014 CCTTCATCTTGTAAACGTTGAGCAAAATTGTTAAAAATATGAGGCGGGTTATATTGCCTA 4477

KJ660348.2014 CCTTCATCTTGTAAACGTTGAGCAAAATTGTTAAAAATATGAGGCGGGTTATATTGCCTA 4500

KU143818.2014 CCTTCATCTTGTAAACGTTGAGCAAAATTGTTAAAAATATGAGGCGGGTTATATTGCCTA 4500

KT725333.2014 CCTTCATCTTGTAAACGTTGAGCAAAATTGTTAAAAATATGAGGCGGGTTATATTGCCTA 4465

KR819004.2014 CCTTCATCTTGTAAACGTTGAGCAAAATTGTTAAAAATATGAGGCGGGTTATATTGCCTA 4447

KP271020.2014 CCTTCATCTTGTAAACGTTGAGCAAAATTGTTAAAAATATGAGGCGGGTTATATTGCCTA 4447

KM519951.2014 CCTTCATCTTGTAAACGTTGAGCAAAATTGTTAAAAATATGAGGCGGGTTATATTGCCTA 4497

MH733488.2018 CCTTCATCTTGTAAACGTTGAGCAAAATTGTTAAAAATATGAGGCGGGTTATATTGCCTA 4491

MH733491.2018 CCTTCATCTTGTAAACGTTGAGCAAAATTGTTAAAAATATGAGGCGGGTTATATTGCCTA 4486

MH733478.2018 CCTTCATCTTGTAAACGTTGAGCAAAATTGTTAAAAATATGAGGCGGGTTATATTGCCTA 4489

MK007330.2018 CCTTCATCTTGTAAACGTTGAGCAAAATTGTTAAAAATATGAGGCGGGTTATATTGCCTA 4488

MK007344.2018 CCTTCATCTTGTAAACGTTGAGCAAAATTGTTAAAAATATGAGGCGGGTTATATTGCCTA 4482

******* ************************ ******************** ****

AY142960.1976 CTGCTCCTCCTGAATATATGGAGGCCATATACCCTGTCAGGTCAAATTCAACAATTGCTA 4560

KC242791.1977 CTGCTCCTCCTGAATATATGGAGGCCATATACCCTGTCAGGTCAAATTCAACAATTGCTA 4560

AF499101.1976 CTGCTCCTCCTGAATATATGGAGGCCATATACCCTGTCAGGTCAAATTCAACAATTGCTA 4560

KF113528.2003 CTGCTCCTCCTGAATATATGGAGGCCATATACCCTGTCAGGTCAAATTCAACAATTGCTA 4556

KC242800.2002 CTGCTCCTCCTGAATATATGGAGGCCATATACCCTGTCAGGTCAAATTCAACAATTGCTA 4560

KY471090.2001 CTGCTCCTCCTGAATATATGGAGGCCATATACCCTGTCAGGTCAAATTCAACAATTGCTA 4537

KY471092.2001 CTGCTCCTCCTGAATATATGGAGGCCATATACCCTGTCAGGTCAAATTCAACAATTGCTA 4537

MH481611.2017 CTGCTCCTCCTGAATATATGGAGGCCATATACCCTGTCAGGTCAAATTCAACAATTGCTA 4526

MH613311.2017 CTGCTCCTCCTGAATATATGGAGGCCATATACCCTGTCAGGTCAAATTCAACAATTGCTA 4505

KC242792.1994 CTGCTCCTCCTGAATATATGGAGGCCATATACCCTGTCAGGTCAAATTCAACAATTGCTA 4560

KC242793.1996 CTGCTCCTCCTGAATATATGGAGGCCATATACCCTGTCAGGTCAAATTCAACAATTGCTA 4560

KC242794.1996 CTGCTCCTCCTGAATATATGGAGGCCATATACCCTGTCAGGTCAAATTCAACAATTGCTA 4560

MH121164.1995 CTGCTCCTCCTGAATATATGGAGGCCATATACCCTGTCAGGTCAAATTCAACAATTGCTA 4560

AY354458.1995 CTGCTCCTCCTGAATATATGGAGGCCATATACCCTGTCAGGTCAAATTCAACAATTGCTA 4560

KT762962.1995 CTGCTCCTCCTGAATATATGGAGGCCATATACCCTGTCAGGTCAAATTCAACAATTGCTA 4560

HQ613402.2008 CTGCTCCTCCTGAATATATGGAGGCCATATACCCTGTCAGGTCAAATTCAACAATTGCTA 4479

KC242789.2007 CTGCTCCTCCTGAATATATGGAGGCCATATACCCTGTCAGGTCAAATTCAACAATTGCTA 4560

HQ613403.2007 CTGCTCCTCCTGAATATATGGAGGCCATATACCCTGTCAGGTCAAATTCAACAATTGCTA 4512

KC242785.2007 CTGCTCCTCCTGAATATATGGAGGCCATATACCCTGTCAGGTCAAATTCAACAATTGCTA 4560

KC242790.2007 CTGCTCCTCCTGAATATATGGAGGCCATATACCCTGTCAGGTCAAATTCAACAATTGCTA 4560

KU143789.2014 CTGCTCCTCCTGAATATATGGAGGCCATATACCCTGCCAGGTCAAATTCAACAATTGCTA 4560

KR817168.2014 CTGCTCCTCCTGAATATATGGAGGCCATATACCCTGCCAGGTCAAATTCAACAATTGCTA 4534

KY426696.2015 CTGCTCCTCCTGAATATATGGAGGCCATATACCCTGCCAGGTCAAATTCAACAATTGCTA 4560

KR105271.2014 CTGCTCCTCCTGAATATATGGAGGCCATATACCCTGCCAGGTCAAATTCAACAATTGCTA 4527

KY007522.2016 CTGCTCCTCCTGAATATATGGAGGCCATATACCCTGCCAGGTCAAATTCAACAATTGCTA 4540

KM034555.2014 CTGCTCCTCCTGAATATATGGAGGCCATATACCCTGCCAGGTCAAATTCAACAATTGCTA 4552

MH470381.2015 CTGCTCCTCCTGAATATATGGAGGCCATATACCCTGCCAGGTCAAATTCAACAATTGCTA 4524

MH470382.2015 CTGCTCCTCCTGAATATATGGAGGCCATATACCCTGCCAGGTCAAATTCAACAATTGCTA 4524

MF102255.2014 CTGCTCCTCCTGAATATATGGAGGCCATATACCCTGCCAGGTCAAATTCAACAATTGCTA 4537

KJ660348.2014 CTGCTCCTCCTGAATATATGGAGGCCATATACCCTGCCAGGTCAAATTCAACAATTGCTA 4560

KU143818.2014 CTGCTCCTCCTGAATATATGGAGGCCATATACCCTGCCAGGTCAAATTCAACAATTGCTA 4560

KT725333.2014 CTGCTCCTCCTGAATATATGGAGGCCATATACCCTGCCAGGTCAAATTCAACAATTGCTA 4525

KR819004.2014 CTGCTCCTCCTGAATATATGGAGGCCATATACCCTGTCAGGTCAAATTCAACAATTGCTA 4507

KP271020.2014 CTGCTCCTCCTGAATATATGGAGGCCATATACCCTGTCAGGTCAAATTCAACAATTGCTA 4507

KM519951.2014 CTGCTCCTCCTGAATATATGGAGGCCATATACCCTGTCAGGTCAAATTCAACAATTGCTA 4557

MH733488.2018 CTGCTCCTCCTGAATATATGGAGGCCATATACCCTGTCAGGTCAAATTCAACAATTGCTA 4551

MH733491.2018 CTGCTCCTCCTGAATATATGGAGGCCATATACCCTGTCAGGTCAAATTCAACAATTGCTA 4546

MH733478.2018 CTGCTCCTCCTGAATATATGGAGGCCATATACCCTGTCAGGTCAAATTCAACAATTGCTA 4549

MK007330.2018 CTGCTCCTCCTGAATATATGGAGGCCATATACCCTGTCAGGTCAAATTCAACAATTGCTA 4548

MK007344.2018 CTGCTCCTCCTGAATATATGGAGGCCATATACCCTGTCAGGTCAAATTCAACAATTGCTA 4542

************************************ ***********************

AY142960.1976 GAGGTGGCAACAGCAATACAGGCTTCCTGACACCGGAGTCAGTCAATGGGGACACTCCAT 4620

KC242791.1977 GAGGTGGCAACAGCAATACAGGCTTCCTGACACCGGAGTCAGTCAATGGGGACACTCCAT 4620

AF499101.1976 GAGGTGGCAACAGCAATACAGGCTTCCTGACACCGGAGTCAGTCAATGGGGACACTCCAT 4620

KF113528.2003 GGGGTGGCAACAACAATACAGGCTTCCTGACACCGGAGTCAGTCAATGGAGACACTCCAT 4616

KC242800.2002 GGGGTGGCAACAACAATACAGGCTTCCTGACACCGGAGTCAGTCAATGGAGACACTCCAT 4620

KY471090.2001 GGGGTGGCAACAACAATACAGGCTTCCTGACACCGGAGTCAGTCAATGGAGACACTCCAT 4597

KY471092.2001 GGGGTGGCAACAACAATACAGGCTTCCTGACACCGGAGTCAGTCAATGGAGACACTCCAT 4597

MH481611.2017 GAGGTGGCAACAGCAATACAGGCTTCCTGACACCGGAGTCAGTCAATGGGGACACTCCAT 4586

MH613311.2017 GAGGTGGCAACAGCAATACAGGCTTCCTGACACCGGAGTCAGTCAATGGGGACACTCCAT 4565

KC242792.1994 GAGGTGGCAACAGCAATACAGGCTTCCTGACACCGGAGTCAGTCAATGGGGACACTCCAT 4620

KC242793.1996 GAGGTGGCAACAGCAATACAGGCTTCCTGACACCGGAGTCAGTCAATGGGGACACTCCAT 4620

KC242794.1996 GAGGTGGCAACAGCAATACAGGCTTCCTGACACCGGAGTCAGTCAATGGGGACACTCCAT 4620

MH121164.1995 GAGGTGGCAACAGCAATACAGGCTTCCTGACACCGGAGTCAGTCAATGGGGACACTCCAT 4620

AY354458.1995 GAGGTGGCAACAGCAATACAGGCTTCCTGACACCGGAGTCAGTCAATGGGGACACTCCAT 4620

KT762962.1995 GAGGTGGCAACAGCAATACAGGCTTCCTGACACCGGAGTCAGTCAATGGGGACACTCCAT 4620

HQ613402.2008 GGGGTGGCAACAGCAATACAGGCTTCCTGACACCGGAGTCAGTCAATGGAGACACTCCAT 4539

KC242789.2007 GGGGTGGCAACAGCAATACAGGCTTCCTGACACCGGAGTCAGTCAATGGAGACACTCCAT 4620

HQ613403.2007 GGGGTGGCAACAGCAATACAGGCTTCCTGACACCGGAGTCAGTCAATGGAGACACTCCAT 4572

KC242785.2007 GGGGTGGCAACAGCAATACAGGCTTCCTGACACCGGAGTCAGTCAATGGAGACACTCCAT 4620

KC242790.2007 GGGGTGGCAACAGCAATACAGGCTTCCTGACACCGGAGTCAGTCAATGGAGACACTCCAT 4620

KU143789.2014 GGGGTGGCAACAGCAATACAGGCTTCCTGACACCGGAGTCAGTCAATGGAGACACTCCAT 4620

KR817168.2014 GGGGTGGCAACAGCAATACAGGCTTCCTGACACCGGAGTCAGTCAATGGAGACACTCCAT 4594

KY426696.2015 GGGGTGGCAACAGCAATACAGGCTTCCTGACACCGGAGTCAGTCAATGGAGACACTCCAT 4620

KR105271.2014 GGGGTGGCAACAGCAATACAGGCTTCCTGACACCGGAGTCAGTCAATGGAGACACTCCAT 4587

KY007522.2016 GGGGTGGCAACAGCAATACAGGCTTCCTGACACCGGAGTCAGTCAATGGAGACACTCCAT 4600

KM034555.2014 GGGGTGGCAACAGCAATACAGGCTTCCTGACACCGGAGTCAGTCAATGGAGACACTCCAT 4612

MH470381.2015 GGGGTGGCAACAGCAATACAGGCTTCCTGACACCGGAGTCAGTCAATGGAGACACTCCAT 4584

MH470382.2015 GGGGTGGCAACAGCAATACAGGCTTCCTGACACCGGAGTCAGTCAATGGAGACACTCCAT 4584

MF102255.2014 GGGGTGGCAACAGCAATACAGGCTTCCTGACACCGGAGTCAGTCAATGGAGACACTCCAT 4597

KJ660348.2014 GGGGTGGCAACAGCAATACAGGCTTCCTGACACCGGAGTCAGTCAATGGAGACACTCCAT 4620

KU143818.2014 GGGGTGGCAACAGCAATACAGGCTTCCTGACACCGGAGTCAGTCAATGGAGACACTCCAT 4620

KT725333.2014 GGGGTGGCAACAGCAATACAGGCTTCCTGACACCGGAGTCAGTCAATGGAGACACTCCAT 4585

KR819004.2014 GAGGTGGCAACAGCAATACAGGCTTCCTGACACCGGAGTCAGTCAATGGGGACACTCCAT 4567

KP271020.2014 GAGGTGGCAACAGCAATACAGGCTTCCTGACACCGGAGTCAGTCAATGGGGACACTCCAT 4567

KM519951.2014 GAGGTGGCAACAGCAATACAGGCTTCCTGACACCGGAGTCAGTCAATGGGGACACTCCAT 4617

MH733488.2018 GAGGTGGCAACAGCAATACAGGCTTCCTGACACCGGAGTCAGTCAATGGGGACACTCCAT 4611

MH733491.2018 GAGGTGGCAACAGCAATACAGGCTTCCTGACACCGGAGTCAGTCAATGGGGACACTCCAT 4606

MH733478.2018 GAGGTGGCAACAGCAATACAGGCTTCCTGACACCGGAGTCAGTCAATGGGGACACTCCAT 4609

MK007330.2018 GAGGTGGCAACAGTAATACAGGCTTCCTGACACCAGAGTCAGTCAATGGGGACACTCCAT 4608

MK007344.2018 GAGGTGGCAACAGTAATACAGGCTTCCTGACACCAGAGTCAGTCAATGGGGACACTCCAT 4602

* ********** ******************** ************** **********

AY142960.1976 CGAATCCACTCAGGCCAATTGCCGATGACACCATCGACCATGCCAGCCACACACCAGGCA 4680

KC242791.1977 CGAATCCACTCAGGCCAATTGCCGATGACACCATCGACCATGCCAGCCACACACCAGGCA 4680

AF499101.1976 CGAATCCACTCAGGCCAATTGCCGATGACACCATCGACCATGCCAGCCACACACCAGGCA 4680

KF113528.2003 CGAATCCACTCAGGCCAATTGCTGATGACACCATCGACCATGCTAGCCACACACCAGGCA 4676

KC242800.2002 CGAATCCACTCAGGCCAATTGCTGATGACACCATCGACCATGCTAGCCACACACCAGGCA 4680

KY471090.2001 CGAATCCACTCAGGCCAATTGCTGATGACACCATCGACCATGCTAGCCACACACCAGGCA 4657

KY471092.2001 CGAATCCACTCAGGCCAATTGCTGATGACACCATCGACCATGCTAGCCACACACCAGGCA 4657

MH481611.2017 CGAATCCACTCAGGCCAATTGCCGATGACACCATCGACCATGCCAGCCACACACCAGGCA 4646

MH613311.2017 CGAATCCACTCAGGCCAATTGCCGATGACACCATCGACCATGCCAGCCACACACCAGGCA 4625

KC242792.1994 CGAATCCACTCAGGCCAATTGCCGATGACACCATCGACCATGCCAGCCACACACCAGGCA 4680

KC242793.1996 CGAATCCACTCAGGCCAATTGCCGATGACACCATCGACCATGCCAGCCACACACCAGGCA 4680

KC242794.1996 CGAATCCACTCAGGCCAATTGCCGATGACACCATCGACCATGCCAGCCACATACCAGGCA 4680

MH121164.1995 CGAATCCACTCAGGCCAATTGCCGATGACACCATCGACCATGCCAGCCACACACCAGGCA 4680

AY354458.1995 CGAATCCACTCAGGCCAATTGCCGATGACACCATCGACCATGCCAGCCACACACCAGGCA 4680

KT762962.1995 CGAATCCACTCAGGCCAATTGCCGATGACACCATCGACCATGCCAGCCACACACCAGGCA 4680

HQ613402.2008 CAAATCCACTCAGACCAATTGCTGATGACACCATCGACCATGCTAGCCACACACCAGGCA 4599

KC242789.2007 CAAATCCACTCAGACCAATTGCTGATGACACCATCGACCATGCTAGCCACACACCAGGCA 4680

HQ613403.2007 CAAATCCACTCAGACCAATTGCTGATGACACCATCGACCATGCTAGCCACACACCAGGCA 4632

KC242785.2007 CAAATCCACTCAGACCAATTGCTGATGACACCATCGACCATGCTAGCCACACACCAGGCA 4680

KC242790.2007 CAAATCCACTCAGACCAATTGCTGATGACACCATCGACCATGCTAGCCACACACCAGGCA 4680

KU143789.2014 CGAATCCACTCAGGCCAATTGCTGATGACACCATCGACCATGCCAGCCACACACCAGGCA 4680

KR817168.2014 CGAATCCACTCAGGCCAATTGCTGATGACACCATCGACCATGCCAGCCACACACCAGGCA 4654

KY426696.2015 CGAATCCACTCAGGCCAATTGCTGATGACACCATCGACCATGCCAGCCACACACCAGGCA 4680

KR105271.2014 CGAATCCACTCAGGCCAATTGCTGATGACACCATCGACCATGCCAGCCACACACCAGGCA 4647

KY007522.2016 CGAATCCACTCAGGCCAATTGCTGATGACACCATCGACCACGCCAGCCACACACCAGGCA 4660

KM034555.2014 CGAATCCACTCAGGCCAATTGCTGATGACACCATCGACCATGCCAGCCACACACCAGGCA 4672

MH470381.2015 CGAATCCACTCAGGCCAATTGCTGATGACACCATCGACCATGCCAGCCACACACCAGGCA 4644

MH470382.2015 CGAATCCACTCAGGCCAATTGCTGATGACACCATCGACCATGCCAGCCACACACCAGGCA 4644

MF102255.2014 CGAATCCACTCAGGCCAATTGCTGATGACACCATCGACCATGCCAGCCACACACCAGGCA 4657

KJ660348.2014 CGAATCCACTCAGGCCAATTGCTGATGACACCATCGACCATGCCAGCCACACACCAGGCA 4680

KU143818.2014 CGAATCCACTCAGGCCAATTGCTGATGACACCATCGACCATGCCAGCCACACACCAGGCA 4680

KT725333.2014 CGAATCCACTCAGGCCAATTGCTGATGACACCATCGACCATGCCAGCCACACACCAGGCA 4645

KR819004.2014 CGAATCCACTCAGGCCAATTGCCGATGACACCATCGACCATGCCAGCCACACACCAGGCA 4627

KP271020.2014 CGAATCCACTCAGGCCAATTGCCGATGACACCATCGACCATGCCAGCCACACACCAGGCA 4627

KM519951.2014 CGAATCCACTCAGGCCAATTGCCGATGACACCATCGACCATGCCAGCCACACACCAGGCA 4677

MH733488.2018 CGAATCCACTCAGGCCAATTGCCGATGACACCATCGACCATGCCAGCCACACACCAGGCA 4671

MH733491.2018 CGAATCCACTCAGGCCAATTGCCGATGACACCATCGACCATGCCAGCCACACACCAGGCA 4666

MH733478.2018 CGAATCCACTCAGGCCAATTGCCGATGACACCATCGACCATGCCAGCCACACACCAGGCA 4669

MK007330.2018 CGAATCCACTCAGGCCAATTGCCGATGACACCATCGACCATGCCAGCCACACACCAGGCA 4668

MK007344.2018 CGAATCCACTCAGGCCAATTGCCGATGACACCATCGACCATGCCAGCCACACACCAGGCA 4662

* *********** ******** ***************** ** ******* ********

AY142960.1976 GTGTGTCATCAGCATTCATCCTTGAAGCTATGGTGAATGTCATATCGGGCCCCAAAGTGC 4740 EBOV-miR-VP-3p (minus)

KC242791.1977 GTGTGTCATCAGCATTCATCCTTGAAGCTATGGTGAATGTCATATCGGGCCCCAAAGTGC 4740

AF499101.1976 GTGTGTCATCAGCATTCATCCTTGAAGCTATGGTGAATGTCATATCGGGCCCCAAAGTGC 4740

KF113528.2003 GTGTGTCATCAGCATTCATCCTTGAAGCTATGGTGAATGTCATATCGGGCCCCAAAGTGC 4736

KC242800.2002 GTGTGTCATCAGCATTCATCCTTGAAGCTATGGTGAATGTCATATCGGGCCCCAAAGTGC 4740

KY471090.2001 GTGTGTCATCAGCATTCATCCTTGAAGCTATGGTGAATGTCATATCGGGCCCCAAAGTGC 4717

KY471092.2001 GTGTGTCATCAGCATTCATCCTTGAAGCTATGGTGAATGTCATATCGGGCCCCAAAGTGC 4717

MH481611.2017 GTGTGTCATCAGCATTCATCCTTGAAGCTATGGTGAATGTCATATCGGGCCCCAAAGTGC 4706

MH613311.2017 GTGTGTCATCAGCATTCATCCTTGAAGCTATGGTGAATGTCATATCGGGCCCCAAAGTGC 4685

KC242792.1994 GTGTGTCATCAGCATTCATCCTTGAAGCTATGGTGAATGTCATATCGGGCCCCAAAGTGC 4740

KC242793.1996 GTGTGTCATCAGCATTCATCCTTGAAGCTATGGTGAATGTCATATCGGGCCCCAAAGTGC 4740

KC242794.1996 GTGTTTCATCAGCATTCATCCTTGAAGCTATGGTGAATGTCATATCGGGCCCCAAAGTGC 4740

MH121164.1995 GTGTGTCATCAGCATTCATCCTTGAAGCTATGGTGAATGTCATATCGGGCCCCAAAGTGC 4740

AY354458.1995 GTGTGTCATCAGCATTCATCCTTGAAGCTATGGTGAATGTCATATCGGGCCCCAAAGTGC 4740

KT762962.1995 GTGTGTCATCAGCATTCATCCTTGAAGCTATGGTGAATGTCATATCGGGCCCCAAAGTGC 4740

HQ613402.2008 GTGTGTCATCAGCATTCATCCTTGAAGCTATGGTGAATGTCATATCGGGCCCCAAAGTGC 4659

KC242789.2007 GTGTGTCATCAGCATTCATCCTTGAAGCTATGGTGAATGTCATATCGGGCCCCAAAGTGC 4740

HQ613403.2007 GTGTGTCATCAGCATTCATCCTTGAAGCTATGGTGAATGTCATATCGGGCCCCAAAGTGC 4692

KC242785.2007 GTGTGTCATCAGCATTCATCCTTGAAGCTATGGTGAATGTCATATCGGGCCCCAAAGTGC 4740

KC242790.2007 GTGTGTCATCAGCATTCATCCTTGAAGCTATGGTGAATGTCATATCGGGCCCCAAAGTGC 4740

KU143789.2014 GTGTGTCATCAGCATTCATCCTCGAAGCTATGGTGAATGTCATATCGGGCCCCAAAGTGC 4740

KR817168.2014 GTGTGTCATCAGCATTCATCCTCGAAGCTATGGTGAATGTCATATCGGGCCCCAAAGTGC 4714

KY426696.2015 GTGTGTCATCAGCATTCATCCTCGAAGCTATGGTGAATGTCATATCGGGCCCCAAAGTGC 4740

KR105271.2014 GTGTGTCATCAGCATTCATCCTCGAAGCTATGGTGAATGTCATATCGGGCCCCAAAGTGC 4707

KY007522.2016 GTGTGTCATCAGCATTCATCCTCGAAGCTATGGTGAATGTCATATCGGGCCCCAAAGTGC 4720

KM034555.2014 GTGTGTCATCAGCATTCATCCTCGAAGCTATGGTGAATGTCATATCGGGCCCCAAAGTGC 4732

MH470381.2015 GTGTGTCATCAGCATTCATCCTCGAAGCTATGGTGAATGTCATATCGGGCCCCAAAGTGC 4704

MH470382.2015 GTGTGTCATCAGCATTCATCCTCGAAGCTATGGTGAATGTCATATCGGGCCCCAAAGTGC 4704

MF102255.2014 GTGTGTCATCAGCATTCATCCTCGAAGCTATGGTGAATGTCATATCGGGCCCCAAAGTGC 4717

KJ660348.2014 GTGTGTCATCAGCATTCATCCTCGAAGCTATGGTGAATGTCATATCGGGCCCCAAAGTGC 4740

KU143818.2014 GTGTGTCATCAGCATTCATCCTCGAAGCTATGGTGAATGTCATATCGGGCCCCAAAGTGC 4740

KT725333.2014 GTGTGTCATCAGCATTCATCCTCGAAGCTATGGTGAATGTCATATCGGGCCCCAAAGTGC 4705

KR819004.2014 GTGTGTCATCAGCATTCATCCTTGAAGCTATGGTGAATGTCATATCGGGCCCCAAAGTGC 4687

KP271020.2014 GTGTGTCATCAGCATTCATCCTTGAAGCTATGGTGAATGTCATATCGGGCCCCAAAGTGC 4687

KM519951.2014 GTGTGTCATCAGCATTCATCCTTGAAGCTATGGTGAATGTCATATCGGGCCCCAAAGTGC 4737

MH733488.2018 GTGTGTCATCAGCATTCATCCTTGAAGCTATGGTGAATGTCATATCGGGCCCCAAAGTGC 4731

MH733491.2018 GTGTGTCATCAGCATTCATCCTTGAAGCTATGGTGAATGTCATATCGGGCCCCAAAGTGC 4726

MH733478.2018 GTGTGTCATCAGCATTCATCCTTGAAGCTATGGTGAATGTCATATCGGGCCCCAAAGTGC 4729

MK007330.2018 GTGTGTCATCAGCATTCATCCTTGAAGCTATGGTGAATGTCATATCGGGCCCCAAAGTGC 4728

MK007344.2018 GTGTGTCATCAGCATTCATCCTTGAAGCTATGGTGAATGTCATATCGGGCCCCAAAGTGC 4722

**** ***************** *************************************

AY142960.1976 TAATGAAGCAAATTCCAATTTGGCTTCCTCTAGGTGTCGCTGATCAAAAGACCTACAGCT 4800

KC242791.1977 TAATGAAGCAAATTCCAATTTGGCTTCCTCTAGGTGTCGCTGATCAAAAGACCTACAGCT 4800

AF499101.1976 TAATGAAGCAAATTCCAATTTGGCTTCCTCTAGGTGTCGCTGATCAAAAGACCTACAGCT 4800

KF113528.2003 TAATGAAGCAAATTCCAATTTGGCTTCCTCTAGGTGTCGCTGATCAAAAGACCTACAGCT 4796

KC242800.2002 TAATGAAGCAAATTCCAATTTGGCTTCCTCTAGGTGTCGCTGATCAAAAGACCTACAGCT 4800

KY471090.2001 TAATGAAGCAAATTCCAATTTGGCTTCCTCTAGGTGTCGCTGATCAAAAGACCTACAGCT 4777

KY471092.2001 TAATGAAGCAAATTCCAATTTGGCTTCCTCTAGGTGTCGCTGATCAAAAGACCTACAGCT 4777

MH481611.2017 TAATGAAGCAAATTCCAATTTGGCTTCCTCTAGGTGTCGCTGATCAAAAGACCTACAGCT 4766

MH613311.2017 TAATGAAGCAAATTCCAATTTGGCTTCCTCTAGGTGTCGCTGATCAAAAGACCTACAGCT 4745

KC242792.1994 TAATGAAGCAAATTCCAATTTGGCTTCCTCTAGGTGTCGCTGATCAAAAGACCTACAGCT 4800

KC242793.1996 TAATGAAGCAAATTCCAATTTGGCTTCCTCTAGGTGTCGCTGATCAAAAGACCTACAGCT 4800

KC242794.1996 TAATGAAGCAAATTCCAATTTGGCTTCCTCTAGGTGTCGCTGATCAAAAGACCTACAGCT 4800

MH121164.1995 TAATGAAGCAAATTCCAATTTGGCTTCCTCTAGGTGTCGCTGATCAAAAGACCTACAGCT 4800

AY354458.1995 TAATGAAGCAAATTCCAATTTGGCTTCCTCTAGGTGTCGCTGATCAAAAGACCTACAGCT 4800

KT762962.1995 TAATGAAGCAAATTCCAATTTGGCTTCCTCTAGGTGTCGCTGATCAAAAGACCTACAGCT 4800

HQ613402.2008 TAATGAAGCAAATTCCAATTTGGCTTCCTCTAGGTGTCGCTGATCAAAAGACCTACAGCT 4719

KC242789.2007 TAATGAAGCAAATTCCAATTTGGCTTCCTCTAGGTGTCGCTGATCAAAAGACCTACAGCT 4800

HQ613403.2007 TAATGAAGCAAATTCCAATTTGGCTTCCTCTAGGTGTCGCTGATCAAAAGACCTACAGCT 4752

KC242785.2007 TAATGAAGCAAATTCCAATTTGGCTTCCTCTAGGTGTCGCTGATCAAAAGACCTACAGCT 4800

KC242790.2007 TAATGAAGCAAATTCCAATTTGGCTTCCTCTAGGTGTCGCTGATCAAAAGACCTACAGCT 4800

KU143789.2014 TAATGAAGCAAATTCCAATTTGGCTTCCTCTAGGTGTCGCTGATCAAAAGACCTACAGCT 4800

KR817168.2014 TAATGAAGCAAATTCCAATTTGGCTTCCTCTAGGTGTCGCTGATCAAAAGACCTACAGCT 4774

KY426696.2015 TAATGAAGCAAATTCCAATTTGGCTTCCTCTAGGTGTCGCTGATCAAAAGACCTACAGCT 4800

KR105271.2014 TAATGAAGCAAATTCCAATTTGGCTTCCTCTAGGTGTCGCTGATCAAAAGACCTACAGCT 4767

KY007522.2016 TAATGAAGCAAATTCCAATTTGGCTTCCTCTAGGTGTCGCTGATCAAAAGACCTACAGCT 4780

KM034555.2014 TAATGAAGCAAATTCCAATTTGGCTTCCTCTAGGTGTCGCTGATCAAAAGACCTACAGCT 4792

MH470381.2015 TAATGAAGCAAATTCCAATTTGGCTTCCTCTAGGTGTCGCTGATCAAAAGACCTACAGCT 4764

MH470382.2015 TAATGAAGCAAATTCCAATTTGGCTTCCTCTAGGTGTCGCTGATCAAAAGACCTACAGCT 4764

MF102255.2014 TAATGAAGCAAATTCCAATTTGGCTTCCTCTAGGTGTCGCTGATCAAAAGACCTACAGCT 4777

KJ660348.2014 TAATGAAGCAAATTCCAATTTGGCTTCCTCTAGGTGTCGCTGATCAAAAGACCTACAGCT 4800

KU143818.2014 TAATGAAGCAAATTCCAATTTGGCTTCCTCTAGGTGTCGCTGATCAAAAGACCTACAGCT 4800

KT725333.2014 TAATGAAGCAAATTCCAATTTGGCTTCCTCTAGGTGTCGCTGATCAAAAGACCTACAGCT 4765

KR819004.2014 TAATGAAGCAAATTCCAATTTGGCTTCCTCTAGGTGTCGCTGATCAAAAGACCTACAGCT 4747

KP271020.2014 TAATGAAGCAAATTCCAATTTGGCTTCCTCTAGGTGTCGCTGATCAAAAGACCTACAGCT 4747

KM519951.2014 TAATGAAGCAAATTCCAATTTGGCTTCCTCTAGGTGTCGCTGATCAAAAGACCTACAGCT 4797

MH733488.2018 TAATGAAGCAAATTCCAATTTGGCTTCCTCTAGGTGTCGCTGATCAAAAGACCTACAGCT 4791

MH733491.2018 TAATGAAGCAAATTCCAATTTGGCTTCCTCTAGGTGTCGCTGATCAAAAGACCTACAGCT 4786

MH733478.2018 TAATGAAGCAAATTCCAATTTGGCTTCCTCTAGGTGTCGCTGATCAAAAGACCTACAGCT 4789

MK007330.2018 TAATGAAGCAAATTCCAATTTGGCTTCCTCTAGGTGTCGCTGATCAAAAGACCTACAGCT 4788

MK007344.2018 TAATGAAGCAAATTCCAATTTGGCTTCCTCTAGGTGTCGCTGATCAAAAGACCTACAGCT 4782

************************************************************ >100 pos conserved

AY142960.1976 TTGACTCAACTACGGCCGCCATCATGCTTGCTTCATACACTATCACCCATTTCGGCAAGG 4860

KC242791.1977 TTGACTCAACTACGGCCGCCATCATGCTTGCTTCATACACTATCACCCATTTCGGCAAGG 4860

AF499101.1976 TTGACTCAACTACGGCCGCCATCATGCTTGCTTCATACACTATCACCCATTTCGGCAAGG 4860

KF113528.2003 TTGACTCAACTACGGCCGCCATCATGCTTGCTTCATATACTATCACCCATTTCGGCAAGG 4856

KC242800.2002 TTGACTCAACTACGGCCGCCATCATGCTTGCTTCATATACTATCACCCATTTCGGCAAGG 4860

KY471090.2001 TTGACTCAACTACGGCCGCCATCATGCTTGCTTCATATACTATCACCCATTTCGGCAAGG 4837

KY471092.2001 TTGACTCAACTACGGCCGCCATCATGCTTGCTTCATATACTATCACCCATTTCGGCAAGG 4837

MH481611.2017 TTGACTCAACTACGGCCGCCATCATGCTTGCTTCATATACTATTACCCATTTCGGCAAGG 4826

MH613311.2017 TTGACTCAACTACGGCCGCCATCATGCTTGCTTCATATACTATTACCCATTTCGGCAAGG 4805

KC242792.1994 TTGACTCAACTACGGCCGCCATCATGCTTGCTTCATATACTATCACCCATTTCGGCAAGG 4860

KC242793.1996 TTGACTCAACTACGGCCGCCATCATGCTTGCTTCATATACTATCACCCATTTCGGCAAGG 4860

KC242794.1996 TTGACTCAACTACGGCCGCCATCATGCTTGCTTCATATACTATCACCCATTTCGGCAAGG 4860

MH121164.1995 TTGACTCAACAACGGCCGCCATCATGCTTGCTTCATATACTATCACCCATTTCGGCAAGG 4860

AY354458.1995 TTGACTCAACAACGGCCGCCATCATGCTTGCTTCATATACTATCACCCATTTCGGCAAGG 4860

KT762962.1995 TTGACTCAACAACGGCCGCCATCATGCTTGCTTCATATACTATCACCCATTTCGGCAAGG 4860

HQ613402.2008 TTGACTCAACTACGGCCGCCATCATGCTTGCTTCATATACTATCACCCATTTCGGCAAGG 4779

KC242789.2007 TTGACTCAACTACGGCCGCCATCATGCTTGCTTCATATACTATCACCCATTTCGGCAAGG 4860

HQ613403.2007 TTGACTCAACTACGGCCGCCATCATGCTTGCTTCATATACTATCACCCATTTCGGCAAGG 4812

KC242785.2007 TTGACTCAACTACGGCCGCCATCATGCTTGCTTCATATACTATCACCCATTTCGGCAAGG 4860

KC242790.2007 TTGACTCAACTACGGCCGCCATCATGCTTGCTTCATATACTATCACCCATTTCGGCAAGG 4860

KU143789.2014 TTGACTCAACTACGGCCGCCATCATGCTTGCTTCATATACTATCACCCATTTCGGCAAGG 4860

KR817168.2014 TTGACTCAACTACGGCCGCCATCATGCTTGCTTCATATACTATCACCCATTTCGGCAAGG 4834

KY426696.2015 TTGACTCAACTACGGCCGCCATCATGCTTGCTTCATATACTATCACCCATTTCGGCAAGG 4860

KR105271.2014 TTGACTCAACTACGGCCGCCATCATGCTTGCTTCATATACTATCACCCATTTCGGCAAGG 4827

KY007522.2016 TTGACTCAACTACGGCCGCCATCATGCTTGCTTCATATACTATCACCCATTTCGGCAAGG 4840

KM034555.2014 TTGACTCAACTACGGCCGCCATCATGCTTGCTTCATATACTATCACCCATTTCGGCAAGG 4852

MH470381.2015 TTGACTCAACTACGGCCGCCATCATGCTTGCTTCATATACTATCACCCATTTCGGCAAGG 4824

MH470382.2015 TTGACTCAACTACGGCCGCCATCATGCTTGCTTCATATACTATCACCCATTTCGGCAAGG 4824

MF102255.2014 TTGACTCAACTACGGCCGCCATCATGCTTGCTTCATATACTATCACCCATTTCGGCAAGG 4837

KJ660348.2014 TTGACTCAACTACGGCCGCCATCATGCTTGCTTCATATACTATCACCCATTTCGGCAAGG 4860

KU143818.2014 TTGACTCAACTACGGCCGCCATCATGCTTGCTTCATATACTATCACCCATTTCGGCAAGG 4860

KT725333.2014 TTGACTCAACTACGGCCGCCATCATGCTTGCTTCATATACTATCACCCATTTCGGCAAGG 4825

KR819004.2014 TTGACTCAACTACGGCCGCCATCATGCTTGCTTCATATACTATCACCCATTTCGGCAAGG 4807

KP271020.2014 TTGACTCAACTACGGCCGCCATCATGCTTGCTTCATATACTATCACCCATTTCGGCAAGG 4807

KM519951.2014 TTGACTCAACTACGGCCGCCATCATGCTTGCTTCATATACTATCACCCATTTCGGCAAGG 4857

MH733488.2018 TTGACTCAACTACGGCCGCCATCATGCTTGCTTCATATACTATCACCCATTTCGGCAAGG 4851

MH733491.2018 TTGACTCAACTACGGCCGCCATCATGCTTGCTTCATATACTATCACCCATTTCGGCAAGG 4846

MH733478.2018 TTGACTCAACTACGGCCGCCATCATGCTTGCTTCATATACTATCACCCATTTCGGCAAGG 4849

MK007330.2018 TTGACTCAACTACGGCCGCCATCATGCTTGCTTCATATACTATCACCCATTTCGGCAAGG 4848

MK007344.2018 TTGACTCAACTACGGCCGCCATCATGCTTGCTTCATATACTATCACCCATTTCGGCAAGG 4842

********** ************************** ***** ****************

AY142960.1976 CAACCAATCCACTTGTCAGAGTCAATCGGCTGGGTCCTGGAATCCCGGATCATCCCCTCA 4920

KC242791.1977 CAACCAATCCACTTGTCAGAGTCAATCGGCTGGGTCCTGGAATCCCGGATCATCCCCTCA 4920

AF499101.1976 CAACCAATCCACTTGTCAGAGTCAATCGGCTGGGTCCTGGAATCCCGGATCATCCCCTCA 4920

KF113528.2003 CAACCAATCCACTTGTCAGAGTCAATCGGCTGGGTCCTGGAATCCCGGATCACCCCCTCA 4916

KC242800.2002 CAACCAATCCACTTGTCAGAGTCAATCGGCTGGGTCCTGGAATCCCGGATCACCCCCTCA 4920

KY471090.2001 CAACCAATCCACTTGTCAGAGTCAATCGGCTGGGTCCTGGAATCCCGGATCACCCCCTCA 4897

KY471092.2001 CAACCAATCCACTTGTCAGAGTCAATCGGCTGGGTCCTGGAATCCCGGATCACCCCCTCA 4897

MH481611.2017 CAACCAATCCACTTGTCAGAGTCAATCGGCTGGGTCCTGGAATCCCGGATCACCCCCTCA 4886

MH613311.2017 CAACCAATCCACTTGTCAGAGTCAATCGGCTGGGTCCTGGAATCCCGGATCACCCCCTCA 4865

KC242792.1994 CAACTAATCCACTTGTCAGAGTCAATCGGCTGGGTCCTGGAATCCCGGATCACCCCCTCA 4920

KC242793.1996 CAACTAATCCACTTGTCAGAGTCAATCGGCTGGGTCCTGGAATCCCGGATCACCCCCTCA 4920

KC242794.1996 CAACTAATCCACTTGTCAGAGTCAATCGGCTGGGTCCTGGAATCCCGGATCACCCCCTCA 4920

MH121164.1995 CAACCAATCCACTTGTCAGAGTCAATCGGCTGGGTCCTGGAATCCCGGATCACCCCCTCA 4920

AY354458.1995 CAACCAATCCACTTGTCAGAGTCAATCGGCTGGGTCCTGGAATCCCGGATCACCCCCTCA 4920

KT762962.1995 CAACCAATCCACTTGTCAGAGTCAATCGGCTGGGTCCTGGAATCCCGGATCACCCCCTCA 4920

HQ613402.2008 CAACCAATCCACTTGTCAGAGTCAATCGGCTGGGTCCTGGAATCCCGGATCACCCCCTCA 4839

KC242789.2007 CAACCAATCCACTTGTCAGAGTCAATCGGCTGGGTCCTGGAATCCCGGATCACCCCCTCA 4920

HQ613403.2007 CAACCAATCCACTTGTCAGAGTCAATCGGCTGGGTCCTGGAATCCCGGATCACCCCCTCA 4872

KC242785.2007 CAACCAATCCACTTGTCAGAGTCAATCGGCTGGGTCCTGGAATCCCGGATCACCCCCTCA 4920

KC242790.2007 CAACCAATCCACTTGTCAGAGTCAATCGGCTGGGTCCTGGAATCCCGGATCACCCCCTCA 4920

KU143789.2014 CAACCAATCCGCTTGTCAGAGTCAATCGGCTGGGTCCTGGAATCCCGGATCACCCCCTCA 4920

KR817168.2014 CAACCAATCCGCTTGTCAGAGTCAATCGGCTGGGTCCTGGAATCCCGGATCACCCCCTCA 4894

KY426696.2015 CAACCAATCCGCTTGTCAGAGTCAATCGGCTGGGTCCTGGAATCCCGGATCACCCCCTCA 4920

KR105271.2014 CAACCAATCCGCTTGTCAGAGTCAATCGGCTGGGTCCTGGAATCCCGGATCACCCCCTCA 4887

KY007522.2016 CAACCAATCCGCTTGTCAGAGTCAATCGGCTGGGTCCTGGAATCCCGGATCACCCCCTCA 4900

KM034555.2014 CAACCAATCCGCTTGTCAGAGTCAATCGGCTGGGTCCTGGAATCCCGGATCACCCCCTCA 4912

MH470381.2015 CAACCAATCCGCTTGTCAGAGTCAATCGGCTGGGTCCTGGAATCCCGGATCACCCCCTCA 4884

MH470382.2015 CAACCAATCCGCTTGTCAGAGTCAATCGGCTGGGTCCTGGAATCCCGGATCACCCCCTCA 4884

MF102255.2014 CAACCAATCCGCTTGTCAGAGTCAATCGGCTGGGTCCTGGAATCCCGGATCACCCCCTCA 4897

KJ660348.2014 CAACCAATCCGCTTGTCAGAGTCAATCGGCTGGGTCCTGGAATCCCGGATCACCCCCTCA 4920

KU143818.2014 CAACCAATCCGCTTGTCAGAGTCAATCGGCTGGGTCCTGGAATCCCGGATCACCCCCTCA 4920

KT725333.2014 CAACCAATCCGCTTGTCAGAGTCAATCGGCTGGGTCCTGGAATCCCGGATCACCCCCTCA 4885

KR819004.2014 CAACCAATCCACTTGTCAGAGTCAATCGGCTGGGTCCTGGAATCCCGGATCACCCCCTCA 4867

KP271020.2014 CAACCAATCCACTTGTCAGAGTCAATCGGCTGGGTCCTGGAATCCCGGATCACCCCCTCA 4867

KM519951.2014 CAACCAATCCACTTGTCAGAGTCAATCGGCTGGGTCCTGGAATCCCGGATCACCCCCTCA 4917

MH733488.2018 CAACCAATCCACTTGTCAGAGTCAATCGGCTGGGTCCTGGAATCCCGGATCACCCCCTCA 4911

MH733491.2018 CAACCAATCCACTTGTCAGAGTCAATCGGCTGGGTCCTGGAATCCCGGATCACCCCCTCA 4906

MH733478.2018 CAACCAATCCACTTGTCAGAGTCAATCGGCTGGGTCCTGGAATCCCGGATCACCCCCTCA 4909

MK007330.2018 CAACCAATCCACTTGTCAGAGTCAATCGGCTGGGTCCCGGAATCCCGGATCACCCCCTCA 4908

MK007344.2018 CAACCAATCCACTTGTCAGAGTCAATCGGCTGGGTCCCGGAATCCCGGATCACCCCCTCA 4902

**** ***** ************************** ************** *******

AY142960.1976 GGCTCCTGCGAATTGGAAACCAGGCTTTCCTCCAGGAGTTCGTTCTTCCGCCAGTCCAAC 4980

KC242791.1977 GGCTCCTGCGAATTGGAAACCAGGCTTTCCTCCAGGAGTTCGTTCTTCCGCCAGTCCAAC 4980

AF499101.1976 GGCTCCTGCGAATTGGAAACCAGGCTTTCCTCCAGGAGTTCGTTCTTCCGCCAGTCCAAC 4980

KF113528.2003 GGCTCCTGCGAATTGGAAACCAGGCCTTCCTCCAGGAGTTCGTTCTTCCGCCAGTCCAAC 4976

KC242800.2002 GGCTCCTGCGAATTGGAAACCAGGCCTTCCTCCAGGAGTTCGTTCTTCCGCCAGTCCAAC 4980

KY471090.2001 GGCTCCTGCGAATTGGAAACCAGGCCTTCCTCCAGGAGTTCGTTCTTCCGCCAGTCCAAC 4957

KY471092.2001 GGCTCCTGCGAATTGGAAACCAGGCCTTCCTCCAGGAGTTCGTTCTTCCGCCAGTCCAAC 4957

MH481611.2017 GACTCCTGCGAATTGGAAACCAGGCTTTCCTCCAGGAGTTCGTTCTTCCGCCAGTCCAAC 4946

MH613311.2017 GACTCCTGCGAATTGGAAACCAGGCTTTCCTCCAGGAGTTCGTTCTTCCGCCAGTCCAAC 4925

KC242792.1994 GACTCCTGCGAATTGGAAACCAGGCCTTCCTCCAGGAGTTCGTTCTTCCGCCAGTCCAAC 4980

KC242793.1996 GACTCCTGCGAATTGGAAACCAGGCCTTCCTCCAGGAGTTCGTTCTTCCGCCAGTCCAAC 4980

KC242794.1996 GACTCCTGCGAATTGGAAACCAGGCCTTCCTCCAGGAGTTCGTTCTTCCGCCAGTCCAAC 4980

MH121164.1995 GGCTCCTGCGAATTGGAAACCAGGCCTTCCTCCAGGAGTTCGTTCTTCCGCCAGTCCAAC 4980

AY354458.1995 GGCTCCTGCGAATTGGAAACCAGGCCTTCCTCCAGGAGTTCGTTCTTCCGCCAGTCCAAC 4980

KT762962.1995 GGCTCCTGCGAATTGGAAACCAGGCCTTCCTCCAGGAGTTCGTTCTTCCGCCAGTCCAAC 4980

HQ613402.2008 GGCTCCTGCGAATTGGGAACCAGGCTTTCCTCCAGGAGTTCGTTCTTCCGCCAGTCCAAC 4899

KC242789.2007 GGCTCCTGCGAATTGGAAACCAGGCTTTCCTCCAGGAGTTCGTTCTTCCGCCAGTCCAAC 4980

HQ613403.2007 GGCTCCTGCGAATTGGAAACCAGGCTTTCCTCCAGGAGTTCGTTCTTCCGCCAGTCCAAC 4932

KC242785.2007 GGCTCCTGCGAATTGGAAACCAGGCTTTCCTCCAGGAGTTCGTTCTTCCGCCAGTCCAAC 4980

KC242790.2007 GGCTCCTGCGAATTGGAAACCAGGCTTTCCTCCAGGAGTTCGTTCTTCCGCCAGTCCAAC 4980

KU143789.2014 GGCTCCTGCGAATTGGAAACCAGGCTTTCCTCCAGGAGTTCGTTCTTCCACCAGTCCAAC 4980

KR817168.2014 GGCTCCTGCGAATTGGAAACCAGGCTTTCCTCCAGGAGTTCGTTCTTCCACCAGTCCAAC 4954

KY426696.2015 GGCTCCTGCGAATTGGAAACCAGGCTTTCCTCCAGGAGTTCGTTCTTCCACCAGTCCAAC 4980

KR105271.2014 GGCTCCTGCGAATTGGAAACCAGGCTTTCCTCCAGGAGTTCGTTCTTCCACCAGTCCAAC 4947

KY007522.2016 GGCTCCTGCGAATTGGAAACCAGGCTTTCCTCCAGGAGTTCGTTCTTCCACCAGTCCAAC 4960

KM034555.2014 GGCTCCTGCGAATTGGAAACCAGGCTTTCCTCCAGGAGTTCGTTCTTCCACCAGTCCAAC 4972

MH470381.2015 GGCTCCTGCGAATTGGAAACCAGGCTTTCCTCCAGGAGTTCGTTCTTCCACCAGTCCAAC 4944

MH470382.2015 GGCTCCTGCGAATTGGAAACCAGGCTTTCCTCCAGGAGTTCGTTCTTCCACCAGTCCAAC 4944

MF102255.2014 GGCTCCTGCGAATTGGAAACCAGGCTTTCCTCCAGGAGTTCGTTCTTCCACCAGTCCAAC 4957

KJ660348.2014 GGCTCCTGCGAATTGGAAACCAGGCTTTCCTCCAGGAGTTCGTTCTTCCACCAGTCCAAC 4980

KU143818.2014 GGCTCCTGCGAATTGGAAACCAGGCTTTCCTCCAGGAGTTCGTTCTTCCACCAGTCCAAC 4980

KT725333.2014 GGCTCCTGCGAATTGGAAACCAGGCTTTCCTCCAGGAGTTCGTTCTTCCACCAGTCCAAC 4945

KR819004.2014 GGCTCCTGCGAATTGGAAACCAGGCCTTCCTCCAGGAGTTCGTTCTTCCGCCAGTCCAAC 4927

KP271020.2014 GGCTCCTGCGAATTGGAAACCAGGCCTTCCTCCAGGAGTTCGTTCTTCCGCCAGTCCAAC 4927

KM519951.2014 GGCTCCTGCGAATTGGAAACCAGGCCTTCCTCCAGGAGTTCGTTCTTCCGCCAGTCCAAC 4977

MH733488.2018 GGCTCCTGCGAATTGGAAACCAGGCCTTCCTCCAGGAGTTCGTTCTTCCGCCAGTCCAAC 4971

MH733491.2018 GGCTCCTGCGAATTGGAAACCAGGCCTTCCTCCAGGAGTTCGTTCTTCCGCCAGTCCAAC 4966

MH733478.2018 GGCTCCTGCGAATTGGAAACCAGGCCTTCCTCCAGGAGTTCGTTCTTCCGCCAGTCCAAC 4969

MK007330.2018 GGCTCCTGCGAATTGGAAACCAGGCTTTCCTCCAGGAGTTCGTTCTTCCGCCAGTCCAAC 4968

MK007344.2018 GGCTCCTGCGAATTGGAAACCAGGCTTTCCTCCAGGAGTTCGTTCTTCCGCCAGTCCAAC 4962

* ************** ******** *********************** **********

AY142960.1976 TACCCCAGTATTTCACCTTTGATTTGACAGCACTCAAACTGATCACCCAACCACTGCCTG 5040

KC242791.1977 TACCCCAGTATTTCACCTTTGATTTGACAGCACTCAAACTGATCACCCAACCACTGCCTG 5040

AF499101.1976 TACCCCAGTATTTCACCTTTGATTTGACAGCACTCAAACTGATCACCCAACCACTGCCTG 5040

KF113528.2003 TACCCCAGTATTTCACCTTTGATTTGACAGCACTCAAACTGATCACCCAACCACTGCCTG 5036

KC242800.2002 TACCCCAGTATTTCACCTTTGATTTGACAGCACTCAAACTGATCACCCAACCACTGCCTG 5040

KY471090.2001 TACCCCAGTATTTCACCTTTGATTTGACAGCACTCAAACTGATCACCCAACCACTGCCTG 5017

KY471092.2001 TACCCCAGTATTTCACCTTTGATTTGACAGCACTCAAACTGATCACCCAACCACTGCCTG 5017

MH481611.2017 TACCCCAGTATTTCACCTTTGATTTGACAGCACTCAAACTGATCACCCAACCACTGCCTG 5006

MH613311.2017 TACCCCAGTATTTCACCTTTGATTTGACAGCACTCAAACTGATCACCCAACCACTGCCTG 4985

KC242792.1994 TACCCCAGTATTTCACCTTTGATTTGACAGCACTCAAACTGATCACCCAACCACTGCCTG 5040

KC242793.1996 TACCCCAGTATTTCACCTTTGATTTGACAGCACTCAAACTGATCACCCAACCACTGCCTG 5040

KC242794.1996 TACCCCAGTATTTCACCTTTGATTTGACAGCACTCAAACTGATCACCCAACCACTGCCTG 5040

MH121164.1995 TACCCCAGTATTTCACCTTTGATTTGACAGCACTCAAACTGATCACCCAACCACTGCCTG 5040

AY354458.1995 TACCCCAGTATTTCACCTTTGATTTGACAGCACTCAAACTGATCACCCAACCACTGCCTG 5040

KT762962.1995 TACCCCAGTATTTCACCTTTGATTTGACAGCACTCAAACTGATCACCCAACCACTGCCTG 5040

HQ613402.2008 TACCCCAGTATTTCACCTTTGATTTGACAGCACTCAAACTGATCACCCAACCACTGCCTG 4959

KC242789.2007 TACCCCAGTATTTCACCTTTGATTTGACAGCACTCAAACTGATCACCCAACCACTGCCTG 5040

HQ613403.2007 TACCCCAGTATTTCACCTTTGATTTGACAGCACTCAAACTGATCACCCAACCACTGCCTG 4992

KC242785.2007 TACCCCAGTATTTCACCTTTGATTTGACAGCACTCAAACTGATCACCCAACCACTGCCTG 5040

KC242790.2007 TACCCCAGTATTTCACCTTTGATTTGACAGCACTCAAACTGATCACCCAACCACTGCCTG 5040

KU143789.2014 TACCCCAGTATTTCACCTTTGATTTGACAGCACTCAAACTGATCACTCAACCACTGCCTG 5040

KR817168.2014 TACCCCAGTATTTCACCTTTGATTTGACAGCACTCAAACTGATCACTCAACCACTGCCTG 5014

KY426696.2015 TACCCCAGTATTTCACCTTTGATTTGACAGCACTCAAACTGATCACTCAACCACTGCCTG 5040

KR105271.2014 TACCCCAGTATTTCACCTTTGATTTGACAGCACTCAAACTGATCACTCAACCACTGCCTG 5007

KY007522.2016 TACCCCAGTATTTCACCTTTGATTTGACAGCACTCAAACTGATCACTCAACCACTGCCTG 5020

KM034555.2014 TACCCCAGTATTTCACCTTTGATTTGACAGCACTCAAACTGATCACTCAACCACTGCCTG 5032

MH470381.2015 TACCCCAGTATTTCACCTTTGATTTGACAGCACTCAAACTGATCACTCAACCACTGCCTG 5004

MH470382.2015 TACCCCAGTATTTCACCTTTGATTTGACAGCACTCAAACTGATCACTCAACCACTGCCTG 5004

MF102255.2014 TACCCCAGTATTTCACCTTTGATTTGACAGCACTCAAACTGATCACTCAACCACTGCCTG 5017

KJ660348.2014 TACCCCAGTATTTCACCTTTGATTTGACAGCACTCAAACTGATCACTCAACCACTGCCTG 5040

KU143818.2014 TACCCCAGTATTTCACCTTTGATTTGACAGCACTCAAACTGATCACTCAACCACTGCCTG 5040

KT725333.2014 TACCCCAGTATTTCACCTTTGATTTGACAGCACTCAAACTGATCACTCAACCACTGCCTG 5005

KR819004.2014 TACCCCAGTATTTCACCTTTGATTTGACAGCACTCAAACTAATCACCCAACCACTGCCTG 4987

KP271020.2014 TACCCNNGTATTTCACCTTTGATTTGACAGCACTCAAACTAATCACCCAACCACTGCCTG 4987

KM519951.2014 TACCCCAGTATTTCACCTTTGATTTGACAGCACTCAAACTAATCACCCAACCACTGCCTG 5037

MH733488.2018 TACCCCAGTATTTCACCTTTGATTTGACAGCACTCAAACTGATCACCCAACCACTGCCTG 5031

MH733491.2018 TACCCCAGTATTTCACCTTTGATTTGACAGCACTCAAACTGATCACCCAACCACTGCCTG 5026

MH733478.2018 TACCCCAGTATTTCACCTTTGATTTGACAGCACTCAAACTGATCACCCAACCACTGCCTG 5029

MK007330.2018 TACCCCAGTATTTCACCTTTGATTTGACAGCACTCAAACTGATCACCCAACCACTGCCTG 5028

MK007344.2018 TACCCCAGTATTTCACCTTTGATTTGACAGCACTCAAACTGATCACCCAACCACTGCCTG 5022

***** ********************************* ***** *************

AY142960.1976 CTGCAACATGGACCGATGACACTCCAACAGGATCAAATGGAGCGTTGCGTCCAGGAATTT 5100

KC242791.1977 CTGCAACATGGACCGATGACACTCCAACAGGATCAAATGGAGCGTTGCGTCCAGGAATTT 5100

AF499101.1976 CTGCAACATGGACCGATGACACTCCAACAGGATCAAATGGAGCGTTGCGTCCAGGAATTT 5100

KF113528.2003 CTGCAACATGGACCGATGACACTCCAACAGGATCAAATGGAGCGCTGCGTCCAGGGATTT 5096

KC242800.2002 CTGCAACATGGACCGATGACACTCCAACAGGATCAAATGGAGCGCTGCGTCCAGGAATTT 5100

KY471090.2001 CTGCAACATGGACCGATGACACTCCAACAGGATCAAATGGAGCGCTGCGTCCAGGAATTT 5077

KY471092.2001 CTGCAACATGGACCGATGACACTCCAACAGGATCAAATGGAGCGCTGCGTCCAGGAATTT 5077

MH481611.2017 CTGCAACATGGACCGATGACGCTCCAACAGGATCAAATGGAGCGTTGCGTCCAGGAATTT 5066

MH613311.2017 CTGCAACATGGACCGATGACGCTCCAACAGGATCAAATGGAGCGTTGCGTCCAGGAATTT 5045

KC242792.1994 CTGCAACATGGACCGATGACACTCCAACAGGATCAAATGGAGCGTTGCGCCCAGGAATTT 5100

KC242793.1996 CTGCAACATGGACCGATGACACTCCAACAGGATCAAATGGAGCGTTGCGCCCAGGAATTT 5100

KC242794.1996 CTGCAACATGGACCGATGACACTCCAACAGGATCAAATGGAGCGTTGCGCCCAGGAATTT 5100

MH121164.1995 CTGCAACATGGACCGATGACACTCCAACAGGATCAAATGGAGCGTTGCGCCCAGGGATTT 5100

AY354458.1995 CTGCAACATGGACCGATGACACTCCAACAGGATCAAATGGAGCGTTGCGCCCAGGGATTT 5100

KT762962.1995 CTGCAACATGGACCGATGACACTCCAACAGGATCAAATGGAGCGTTGCGCCCAGGGATTT 5100

HQ613402.2008 CTGCAACATGGACCGATGACACTCCAACAGGATCAAACGGAGCGTTGCGTCCAGGAATTT 5019

KC242789.2007 CTGCAACATGGACCGATGACACTCCAACAGGATCAAACGGAGCGTTGCGTCCAGGAATTT 5100

HQ613403.2007 CTGCAACATGGACCGATGACACTCCAACAGGATCAAACGGAGCGTTGCGTCCAGGAATTT 5052

KC242785.2007 CTGCAACATGGACCGATGACACTCCAACAGGATCAAACGGAGCGTTGCGTCCAGGAATTT 5100

KC242790.2007 CTGCAACATGGACCGATGACACTCCAACAGGATCAAACGGAGCGTTGCGTCCAGGAATTT 5100

KU143789.2014 CTGCAACATGGACCGATGACACTCCAACTGGATCAAATGGAGCGTTGCGTCCAGGAATTT 5100

KR817168.2014 CTGCAACATGGACCGATGACACTCCAACTGGATCAAATGGAGCGTTGCGTCCAGGAATTT 5074

KY426696.2015 CTGCAACATGGACCGATGACACTCCAACTGGATCAAATGGAGCGTTGCGTCCAGGAATTT 5100

KR105271.2014 CTGCAACATGGACCGATGACACTCCAACTGGATCAAATGGAGCGTTGCGTCCAGGAATTT 5067

KY007522.2016 CTGCAACATGGACCGATGACACTCCAACTGGATCAAATGGAGCGTTGCGTCCAGGAATTT 5080

KM034555.2014 CTGCAACATGGACCGATGACACTCCAACTGGATCAAATGGAGCGTTGCGTCCAGGAATTT 5092

MH470381.2015 CTGCAACATGGACCGATGACACTCCAACTGGATCAAATGGAGCGTTGCGTCCAGGAATTT 5064

MH470382.2015 CTGCAACATGGACCGATGACACTCCAACTGGATCAAATGGAGCGTTGCGTCCAGGAATTT 5064

MF102255.2014 CTGCAACATGGACCGATGACACTCCAACTGGATCAAATGGAGCGTTGCGTCCAGGAATTT 5077

KJ660348.2014 CTGCAACATGGACCGATGACACTCCAACTGGATCAAATGGAGCGTTGCGTCCAGGAATTT 5100

KU143818.2014 CTGCAACATGGACCGATGACACTCCAACTGGATCAAATGGAGCGTTGCGTCCAGGAATTT 5100

KT725333.2014 CTGCAACATGGACCGATGACACTCCAACTGGATCAAATGGAGCGTTGCGTCCAGGAATTT 5065

KR819004.2014 CTGCAACATGGACCGATGACACTCCAACGGGATCAAATGGAGCGTTGCGCCCAGGAATTT 5047

KP271020.2014 CTGCAACATGGACCGATGACACTCCAACGGGATCAAATGGAGCGTTGCGCCCAGGAATTT 5047

KM519951.2014 CTGCAACATGGACCGATGACACTCCAACGGGATCAAATGGAGCGTTGCGCCCAGGAATTT 5097

MH733488.2018 CTGCAACATGGACCGATGACACTCCAACGGGATCAAATGGAGCGTTGCGCCCAGGAATTT 5091

MH733491.2018 CTGCAACATGGACCGATGACACTCCAACGGGATCAAATGGAGCGTTGCGCCCAGGAATTT 5086

MH733478.2018 CTGCAACATGGACCGATGACACTCCAACGGGATCAAATGGAGCGTTGCGCCCAGGAATTT 5089

MK007330.2018 CTGCAACATGGACCGATGACACTCCAACAGGATCAAATGGAGCATTGCGTCCAGGAATTT 5088

MK007344.2018 CTGCAACATGGACCGATGACACTCCAACAGGATCAAATGGAGCATTGCGTCCAGGAATTT 5082

******************** ******* ******** ***** **** ***** ****

AY142960.1976 CATTTCATCCAAAACTTCGCCCCATTCTTTTACCCAACAAAAGTGGGAAGAAGGGGAACA 5160

KC242791.1977 CATTTCATCCAAAACTTCGCCCCATTCTTTTACCCAACAAAAGTGGGAAGAAGGGGAACA 5160

AF499101.1976 CATTTCATCCAAAACTTCGCCCCATTCTTTTACCCAACAAAAGTGGGAAGAAGGGGAACA 5160

KF113528.2003 CGTTTCATCCAAAACTTCGCCCCATTCTTTTACCTAACAAAAGTGGGAAGAAGGGGAACA 5156

KC242800.2002 CGTTTCATCCAAAACTTCGCCCCATTCTTTTACCTAACAAAAGTGGGAAGAAGGGGAACA 5160

KY471090.2001 CGTTTCATCCAAAACTTCGCCCCATTCTTTTACCTAACAAAAGTGGGAAGAAGGGGAACA 5137

KY471092.2001 CGTTTCATCCAAAACTTCGCCCCATTCTTTTACCTAACAAAAGTGGGAAGAAGGGGAACA 5137

MH481611.2017 CATTTCATCCAAAACTTCGCCCCATTCTTTTACCCAACAAAAGTGGGAAGAAGGGGAACA 5126

MH613311.2017 CATTTCATCCAAAACTTCGCCCCATTCTTTTACCCAACAAAAGTGGGAAGAAGGGGAACA 5105

KC242792.1994 CATTTCATCCAAAACTGCGCCCCATTCTTTTACCCAACAAGAGTGGGAAGAAGGGGAATA 5160

KC242793.1996 CATTTCATCCAAAACTGCGCCCCATTCTTTTACCCAACAAGAGTGGGAAGAAGGGGAATA 5160

KC242794.1996 CATTTCATCCAAAACTGCGCCCCATTCTTTTACCCAACAAGAGTGGGAAGAAGGGGAATA 5160

MH121164.1995 CATTTCATCCAAAACTTCGCCCCATTCTTTTACCCAACAAGAGTGGGAAGAAGGGGAATA 5160

AY354458.1995 CATTTCATCCAAAACTTCGCCCCATTCTTTTACCCAACAAGAGTGGGAAGAAGGGGAATA 5160

KT762962.1995 CATTTCATCCAAAACTTCGCCCCATTCTTTTACCCAACAAGAGTGGGAAGAAGGGGAATA 5160

HQ613402.2008 CATTTCATCCAAAACTTCGCCCCATTCTTTTACCCAACAAAAGTGGGAAGAAGGGGAACA 5079

KC242789.2007 CATTTCATCCAAAACTTCGCCCCATTCTTTTACCCAACAAAAGTGGGAAGAAGGGGAACA 5160

HQ613403.2007 CATTTCATCCAAAACTTCGCCCCATTCTTTTACCCAACAAAAGTGGGAAGAAGGGGAACA 5112

KC242785.2007 CATTTCATCCAAAACTTCGCCCCATTCTTTTACCCAACAAAAGTGGGAAGAAGGGGAACA 5160

KC242790.2007 CATTTCATCCAAAACTTCGCCCCATTCTTTTACCCAACAAAAGTGGGAAGAAGGGGAACA 5160

KU143789.2014 CATTTCATCCAAAACTTCGCCCCATTCTTTTACCCAACAAAAGTGGGAAGAAGGGGAACA 5160

KR817168.2014 CATTTCATCCAAAACTTCGCCCCATTCTTTTACCCAACAAAAGTGGGAAGAAGGGGAACA 5134

KY426696.2015 CATTTCATCCAAAACTTCGCCCCATTCTTTTACCCAACAAAAGTGGGAAGAAGGGGAACA 5160

KR105271.2014 CATTTCATCCAAAACTTCGCCCCATTCTTTTACCCAACAAAAGTGGGAAGAAGGGGAACA 5127

KY007522.2016 CATTTCATCCAAAACTTCGCCCCATTCTTTTACCCAACAAAAGTGGGAAGAAGGGGAACA 5140

KM034555.2014 CATTTCATCCAAAACTTCGCCCCATTCTTTTACCCAACAAAAGTGGGAAGAAGGGGAACA 5152

MH470381.2015 CATTTCATCCAAAACTTCGCCCCATTCTTTTACCCAACAAAAGTGGGAAGAAGGGGAACA 5124

MH470382.2015 CATTTCATCCAAAACTTCGCCCCATTCTTTTACCCAACAAAAGTGGGAAGAAGGGGAACA 5124

MF102255.2014 CATTTCATCCAAAACTTCGCCCCATTCTTTTACCCAACAAAAGTGGGAAGAAGGGGAACA 5137

KJ660348.2014 CATTTCATCCAAAACTTCGCCCCATTCTTTTACCCAACAAAAGTGGGAAGAAGGGGAACA 5160

KU143818.2014 CATTTCATCCAAAACTTCGCCCCATTCTTTTACCCAACAAAAGTGGGAAGAAGGGGAACA 5160

KT725333.2014 CATTTCATCCAAAACTTCGCCCCATTCTTTTACCCAACAAAAGTGGGAAGAAGGGGAACA 5125

KR819004.2014 CATTTCATCCAAAACTTCGCCCCATTCTTTTACCCAACAAGAGTGGGAAGAAGGGGAATA 5107

KP271020.2014 CATTTCATCCAAAACTTCGCCCCATTCTTTTACCCAACAAGAGTGGGAAGAAGGGGAATA 5107

KM519951.2014 CATTTCATCCAAAACTTCGCCCCATTCTTTTACCCAACAAGAGTGGGAAGAAGGGGAATA 5157

MH733488.2018 CATTTCATCCAAAACTTCGCCCCATTCTTTTACCCAACAAGAGTGGGAAGAAGGGGAATA 5151

MH733491.2018 CATTTCATCCAAAACTTCGCCCCATTCTTTTACCCAACAAGAGTGGGAAGAAGGGGAATA 5146

MH733478.2018 CATTTCATCCAAAACTTCGCCCCATTCTTTTACCCAACAAGAGTGGGAAGAAGGGGAATA 5149

MK007330.2018 CATTTCATCCAAAACTTCGCCCCATTCTTTTGCCCAACAAAAGTGGGAAGAAGGGGAACA 5148

MK007344.2018 CATTTCATCCAAAACTTCGCCCCATTCTTTTGCCCAACAAAAGTGGGAAGAAGGGGAACA 5142

* ************** ************** ** ***** ***************** *

AY142960.1976 GTGCCGATCTAACATCTCCGGAGAAAATCCAAGCAATAATGACTTCACTCCAGGACTTTA 5220

KC242791.1977 GTGCCGATCTAACATCTCCGGAGAAAATCCAAGCAATAATGACTTCACTCCAGGACTTCA 5220

AF499101.1976 GTGCCGATCTAACATCTCCGGAGAAAATCCAAGCAATAATGACTTCACTCCAGGACTTCA 5220

KF113528.2003 GTGCCGATCTAACATCTCCAGAGAAAATCCAAGCAATAATGACTTCACTCCAGGACTTTA 5216

KC242800.2002 GTGCCGATCTAACATCTCCAGAGAAAATCCAAGCAATAATGACTTCACTCCAGGACTTTA 5220

KY471090.2001 GTGCCGATCTAACATCTCCAGAGAAAATCCAAGCAATAATGACTTCACTCCAGGACTTTA 5197

KY471092.2001 GTGCCGATCTAACATCTCCAGAGAAAATCCAAGCAATAATGACTTCACTCCAGGACTTTA 5197

MH481611.2017 GTGCCGATCTAACATCTCCGGAGAAAATCCAAGCAATAATGACTTCACTCCAGGACTTTA 5186

MH613311.2017 GTGCCGATCTAACATCTCCGGAGAAAATCCAAGCAATAATGACTTCACTCCAGGACTTTA 5165

KC242792.1994 GTGCCGATCTAACATCTCCGGAGAAAATCCAAGCAATAATGACTTCACTCCAGGACTTAA 5220

KC242793.1996 GTGCCGATCTAACATCTCCGGAGAAAATCCAAGCAATAATGACTTCACTCCAGGACTTTA 5220

KC242794.1996 GTGCCGATCTAACATCTCCGGAGAAAATCCAAGCAATAATGACTTCACTCCAGGACTTTA 5220

MH121164.1995 GTGCCGATCTAACATCTCCGGAGAAAATCCAAGCAATAATGACTTCACTCCAGGACTTTA 5220

AY354458.1995 GTGCCGATCTAACATCTCCGGAGAAAATCCAAGCAATAATGACTTCACTCCAGGACTTTA 5220

KT762962.1995 GTGCCGATCTAACATCTCCGGAGAAAATCCAAGCAATAATGACTTCACTCCAGGACTTTA 5220

HQ613402.2008 ATGCCGATCTAACATCTCCGGAGAAAATCCAAGCAATAATGACTTCACTCCAGGACTTTA 5139

KC242789.2007 ATGCCGATCTAACATCTCCGGAGAAAATCCAAGCAATAATGACTTCACTCCAGGACTTTA 5220

HQ613403.2007 ATGCCGATCTAACATCTCCGGAGAAAATCCAAGCAATAATGACTTCACTCCAGGACTTTA 5172

KC242785.2007 ATGCCGATCTAACATCTCCGGAGAAAATCCAAGCAATAATGACTTCACTCCAGGACTTTA 5220

KC242790.2007 ATGCCGATCTAACATCTCCGGAGAAAATCCAAGCAATAATGACTTCACTCCAGGACTTTA 5220

KU143789.2014 GTGCCGATCTAACATCTCCGGAGAAAATCCAAGCAATAATGACTTCACTCCAGGACTTTA 5220

KR817168.2014 GTGCCGATCTAACATCTCCGGAGAAAATCCAAGCAATAATGACTTCACTCCAGGACTTTA 5194

KY426696.2015 GTGCCGATCTAACATCTCCGGAGAAAATCCAAGCAATAATGACTTCACTCCAGGACTTTA 5220

KR105271.2014 GTGCCGATCTAACATCTCCGGAGAAAATCCAAGCAATAATGACTTCACTCCAGGACTTTA 5187

KY007522.2016 GTGCCGATCTAACATCTCCGGAGAAAATCCAAGCAATAATGACTTCACTCCAGGACTTTA 5200

KM034555.2014 GTGCCGATCTAACATCTCCGGAGAAAATCCAAGCAATAATGACTTCACTCCAGGACTTTA 5212

MH470381.2015 GTGCCGATCTAACATCTCCGGAGAAAATCCAAGCAATAATGACTTCACTCCAGGACTTTA 5184

MH470382.2015 GTGCCGATCTAACATCTCCGGAGAAAATCCAAGCAATAATGACTTCACTCCAGGACTTTA 5184

MF102255.2014 GTGCCGATCTAACATCTCCGGAGAAAATCCAAGCAATAATGACTTCACTCCAGGACTTTA 5197

KJ660348.2014 GTGCCGATCTAACATCTCCGGAGAAAATCCAAGCAATAATGACTTCACTCCAGGACTTTA 5220

KU143818.2014 GTGCCGATCTAACATCTCCGGAGAAAATCCAAGCAATAATGACTTCACTCCAGGACTTTA 5220

KT725333.2014 GTGCCGATCTAACATCTCCGGAGAAAATCCAAGCAATAATGACTTCACTCCAGGACTTTA 5185

KR819004.2014 GTGCCGATCTAACATCTCCGGAGAAAATCCAAGCAATAATGACTTCACTCCAGGACTTTA 5167

KP271020.2014 GTGCCGATCTAACATCTCCGGAGAAAATCCAAGCAATAATGACTTCACTCCAGGACTTTA 5167

KM519951.2014 GTGCCGATCTAACATCTCCGGAGAAAATCCAAGCAATAATGACTTCACTCCAGGACTTTA 5217

MH733488.2018 GTGCCGATCTAACATCTCCGGAGAAAATCCAAGCAATAATGACTTCACTCCAGGACTTTA 5211

MH733491.2018 GTGCCGATCTAACATCTCCGGAGAAAATCCAAGCAATAATGACTTCACTCCAGGACTTTA 5206

MH733478.2018 GTGCCGATCTAACATCTCCGGAGAAAATCCAAGCAATAATGACTTCACTCCAGGACTTTA 5209

MK007330.2018 GTGCCGATCTAACATCTCCAGAGAAAATCCAAGCAATAATGACTTCACTCCAGGACTTTA 5208

MK007344.2018 GTGCCGATCTAACATCTCCAGAGAAAATCCAAGCAATAATGACTTCACTCCAGGACTTTA 5202

****************** ************************************** *

AY142960.1976 AGATCGTTCCAATTGATCCAACCAAAAATATCATGGGAATCGAAGTGCCAGAAACTCTGG 5280

KC242791.1977 AGATCGTTCCAATTGATCCAACCAAAAATATCATGGGAATCGAAGTGCCAGAAACTCTGG 5280

AF499101.1976 AGATCGTTCCAATTGATCCAACCAAAAATATCATGGGAATCGAAGTGCCAGAAACTCTGG 5280

KF113528.2003 AGATCGTTCCAATTGATCCAACCAAAAATATCATGGGTATCGAAGTGCCAGAAACTCTGG 5276

KC242800.2002 AGATCGTTCCAATTGATCCAACCAAAAATATCATGGGTATCGAAGTGCCAGAAACTCTGG 5280

KY471090.2001 AGATCGTTCCAATTGATCCAACCAAAAATATCATGGGTATCGAAGTGCCAGAAACTCTGG 5257

KY471092.2001 AGATCGTTCCAATTGATCCAACCAAAAATATCATGGGTATCGAAGTGCCAGAAACTCTGG 5257

MH481611.2017 AGATCGTTCCAATTGATCCAGCCAAAAATATCATGGGAATCGAAGTGCCAGAAACTCTGG 5246

MH613311.2017 AGATCGTTCCAATTGATCCAGCCAAAAATATCATGGGAATCGAAGTGCCAGAAACTCTGG 5225

KC242792.1994 AGATCGTTCCAATTGATCCAACCAAAAATATCATGGGAATCGAAGTGCCAGAAACTCTGG 5280

KC242793.1996 AGATCGTTCCAATTGATCCAACCAAAAATATCATGGGAATCGAAGTGCCAGAAACTCTGG 5280

KC242794.1996 AGATCGTTCCAATTGATCCAACCAAAAATATCATGGGAATCGAAGTGCCAGAAACTCTGG 5280

MH121164.1995 AGATCGTTCCAATTGATCCAACCAAAAATATCATGGGAATCGAAGTGCCAGAAACTCTGG 5280

AY354458.1995 AGATCGTTCCAATTGATCCAACCAAAAATATCATGGGAATCGAAGTGCCAGAAACTCTGG 5280

KT762962.1995 AGATCGTTCCAATTGATCCAACCAAAAATATCATGGGAATCGAAGTGCCAGAAACTCTGG 5280

HQ613402.2008 AGATCGTTCCAATTGATCCAACCAAAAATATTATGGGTATCGAAGTGCCAGAAACTCTGG 5199

KC242789.2007 AGATCGTTCCAATTGATCCAACCAAAAATATTATGGGTATCGAAGTGCCAGAAACTCTGG 5280

HQ613403.2007 AGATCGTTCCAATTGATCCAACCAAAAATATTATGGGTATCGAAGTGCCAGAAACTCTGG 5232

KC242785.2007 AGATCGTTCCAATTGATCCAACCAAAAATATTATGGGTATCGAAGTGCCAGAAACTCTGG 5280

KC242790.2007 AGATCGTTCCAATTGATCCAACCAAAAATATTATGGGTATCGAAGTGCCAGAAACTCTGG 5280

KU143789.2014 AGATCGTTCCAATTGATCCAACCAAAAATATCATGGGTATCGAAGTGCCAGAAACTCTGG 5280

KR817168.2014 AGATCATTCCAATTGATCCAACCAAAAATATCATGGGTATCGAAGTGCCAGAAACTCTGG 5254

KY426696.2015 AGATCGTTCCAATTGATCCAACCAAAAATATCATGGGTATCGAAGTGCCAGAAACTCTGG 5280

KR105271.2014 AGATCGTTCCAATTGATCCAACCAAAAATATCATGGGTATCGAAGTGCCAGAAACTCTGG 5247

KY007522.2016 AGATCGTTCCAATTGATCCAACCAAAAATATCATGGGTATCGAAGTGCCAGAAACTCTGG 5260

KM034555.2014 AGATCGTTCCAATTGATCCAACCAAAAATATCATGGGTATCGAAGTGCCAGAAACTCTGG 5272

MH470381.2015 AGATCGTTCCAATTGATCCAACCAAAAATATCATGGGTATCGAAGTGCCAGAAACTCTGG 5244

MH470382.2015 AGATCGTTCCAATTGATCCAACCAAAAATATCATGGGTATCGAAGTGCCAGAAACTCTGG 5244

MF102255.2014 AGATCGTTCCAATTGATCCAACCAAAAATATCATGGGTATCGAAGTGCCAGAAACTCTGG 5257

KJ660348.2014 AGATCGTTCCAATTGATCCAACCAAAAATATCATGGGTATCGAAGTGCCAGAAACTCTGG 5280

KU143818.2014 AGATCGTTCCAATTGATCCAACCAAAAATATCATGGGTATCGAAGTGCCAGAAACTCTGG 5280

KT725333.2014 AGATCGTTCCAATTGATCCAACCAAAAATATCATGGGTATCGAAGTGCCAGAAACTCTGG 5245

KR819004.2014 AGATTGTTCCAATTGATCCAACCAAAAATATCATGGGAATCGAAGTGCCAGAAACTCTGG 5227

KP271020.2014 AGATTGTTCCAATTGATCCAACCAAAAATATCATGGGAATCGAAGTGCCAGAAACTCTGG 5227

KM519951.2014 AGATTGTTCCAATTGATCCAACCAAAAATATCATGGGAATCGAAGTGCCAGAAACTCTGG 5277

MH733488.2018 AGATCGTTCCAATTGATCCAACCAAAAATATCATGGGAATCGAAGTGCCAGAAACTCTGG 5271

MH733491.2018 AGATCGTTCCAATTGATCCAACCAAAAATATCATGGGAATCGAAGTGCCAGAAACTCTGG 5266

MH733478.2018 AGATCGTTCCAATTGATCCAACCAAAAATATCATGGGAATCGAAGTGCCAGAAACTCTGG 5269

MK007330.2018 AAATCGTTCCAATTGATCCAGCCAAAAATATCATGGGAATCGAAGTGCCAGAAACTCTGG 5268

MK007344.2018 AAATCGTTCCAATTGATCCAGCCAAAAATATCATGGGAATCGAAGTGCCAGAAACTCTGG 5262

* ** ************** ********** ***** **********************

AY142960.1976 TCCACAAGCTGACCGGTAAGAAGGTGACTTCTAAAAATGGACAACCAATCATCCCTGTTC 5340

KC242791.1977 TCCACAAGCTGACCGGTAAGAAGGTGACTTCTAAAAATGGACAACCAATCATCCCTGTTC 5340

AF499101.1976 TCCACAAGCTGACCGGTAAGAAGGTGACTTCTAAAAATGGACAACCAATCATCCCTGTTC 5340

KF113528.2003 TCCACAAGCTGACCGGTAAGAAGGTGACTTCTAAAAATGGACAACCAATCATCCCTGTTC 5336

KC242800.2002 TCCACAAGCTGACCGGTAAGAAGGTGACTTCTAAAAATGGACAACCAATCATCCCTGTTC 5340

KY471090.2001 TCCACAAGCTGACCGGTAAGAAGGTGACTTCTAAAAATGGACAACCAATCATCCCTGTTC 5317

KY471092.2001 TCCACAAGCTGACCGGTAAGAAGGTGACTTCTAAAAATGGACAACCAATCATCCCTGTTC 5317

MH481611.2017 TCCACAAGCTGACCGGTAAGAAGGTGAATTCTAAAGATGGACAACCAATCATCCCTGTTC 5306

MH613311.2017 TCCACAAGCTGACCGGTAAGAAGGTGAATTCTAAAGATGGACAACCAATCATCCCTGTTC 5285

KC242792.1994 TCCACAAGCTGACCGGTAAGAAGGTGACTTCTAAAAATGGACAACCAATCATCCCTGTTC 5340

KC242793.1996 TCCACAAGCTGACCGGTAAGAAGGTGACTTCTAAAAATGGACAACCAATCATCCCTGTTC 5340

KC242794.1996 TCCACAAGCTGACCGGTAAGAAGGTGACTTCTAAAAATGGACAACCAATCATCCCTGTTC 5340

MH121164.1995 TCCACAAGCTGACCGGTAAGAAGGTGACTTCTAAAAATGGACAACCAATCATCCCTGTTC 5340

AY354458.1995 TCCACAAGCTGACCGGTAAGAAGGTGACTTCTAAAAATGGACAACCAATCATCCCTGTTC 5340

KT762962.1995 TCCACAAGCTGACCGGTAAGAAGGTGACTTCTAAAAATGGACAACCAATCATCCCTGTTC 5340

HQ613402.2008 TCCACAAGCTGACCGGTAAGAAGGTGACTTCTAAAAATGGACAACCAATCATCCCTGTTC 5259

KC242789.2007 TCCACAAGCTGACCGGTAAGAAGGTGACTTCTAAAAATGGACAACCAATCATCCCTGTTC 5340

HQ613403.2007 TCCACAAGCTGACCGGTAAGAAGGTGACTTCTAAAAATGGACAACCAATCATCCCTGTTC 5292

KC242785.2007 TCCACAAGCTGACCGGTAAGAAGGTGACTTCTAAAAATGGACAACCAATCATCCCTGTTC 5340

KC242790.2007 TCCACAAGCTGACCGGTAAGAAGGTGACTTCTAAAAATGGACAACCAATCATCCCTGTTC 5340

KU143789.2014 TCCACAAGCTGACCGGTAAGAAGGTGACTTCCAAAAATGGACAACCAATCATCCCTGTTC 5340

KR817168.2014 TCCACAAGCTGACCGGTAAGAAGGTGACTTCCAAAAATGGACAACCAATCATCCCTGTTC 5314

KY426696.2015 TCCACAAGCTGACCGGTAAGAAGGTGACTTCCAAAAATGGACAACCAATCATCCCTGTTC 5340

KR105271.2014 TCCACAAGCTGACCGGTAAGAAGGTGACTTCCAAAAATGGACAACCAATCATCCCTGTTC 5307

KY007522.2016 TCCACAAGCTGACCGGTAAGAAGGTGACTTCCAAAAATGGACAACCAATCATCCCTGTTC 5320

KM034555.2014 TCCACAAGCTGACCGGTAAGAAGGTGACTTCCAAAAATGGACAACCAATCATCCCTGTTC 5332

MH470381.2015 TCCACAAGCTGACCGGTAAGAAGGTGACTTCCAAAAATGGACAACCAATCATCCCTGTTC 5304

MH470382.2015 TCCACAAGCTGACCGGTAAGAAGGTGACTTCCAAAAATGGACAACCAATCATCCCTGTTC 5304

MF102255.2014 TCCACAAGCTGACCGGTAAGAAGGTGACTTCCAAAAATGGACAACCAATCATCCCTGTTC 5317

KJ660348.2014 TCCACAAGCTGACCGGTAAGAAGGTGACTTCCAAAAATGGACAACCAATCATCCCTGTTC 5340

KU143818.2014 TCCACAAGCTGACCGGTAAGAAGGTGACTTCCAAAAATGGACAACCAATCATCCCTGTTC 5340

KT725333.2014 TCCACAAGCTGACCGGTAAGAAGGTGACTTCCAAAAATGGACAACCAATCATCCCTGTTC 5305

KR819004.2014 TCCACAAGCTGACCGGTAAGAAGGTGACTTCTAAAAATGGACAACCAATCATCCCTGTTC 5287

KP271020.2014 TCCACAAGCTGACCGGTAAGAAGGTGACTTCTAAAAATGGACAACCAATCATCCCTGTTC 5287

KM519951.2014 TCCACAAGCTGACCGGTAAGAAGGTGACTTCTAAAAATGGACAACCAATCATCCCTGTTC 5337

MH733488.2018 TCCACAAGCTGACCGGTAAGAAGGTGACTTCTAAAAATGGACAACCAATCATCCCTGTTC 5331

MH733491.2018 TCCACAAGCTGACCGGTAAGAAGGTGACTTCTAAAAATGGACAACCAATCATCCCTGTTC 5326

MH733478.2018 TCCACAAGCTGACCGGTAAGAAGGTGACTTCTAAAAATGGACAACCAATCATCCCTGTTC 5329

MK007330.2018 TCCACAAGCTGACTGGTAAGAAGGTGAATTCTAAAAATGGACAACCAATCATCCCTGTTC 5328

MK007344.2018 TCCACAAGCTGACTGGTAAGAAGGTGAATTCTAAAAATGGACAACCAATCATCCCTGTTC 5322

************* ************* *** *** ************************

AY142960.1976 TTTTGCCAAAGTACATTGGGTTGGACCCGGTGGCTCCAGGAGACCTCACCATGGTAATCA 5400

KC242791.1977 TTTTGCCAAAGTACATTGGGTTGGACCCGGTGGCTCCAGGAGACCTCACCATGGTAATCA 5400

AF499101.1976 TTTTGCCAAAGTACATTGGGTTGGACCCGGTGGCTCCAGGAGACCTCACCATGGTAATCA 5400

KF113528.2003 TTTTGCCAAAGTACATTGGGTTGGACCCGGTGGCTCCAGGAGACCTCACCATGGTAATCA 5396

KC242800.2002 TTTTGCCAAAGTACATTGGGTTGGACCCGGTGGCTCCAGGAGACCTCACCATGGTAATCA 5400

KY471090.2001 TTTTGCCAAAGTACATTGGGTTGGACCCGGTGGCTCCAGGAGACCTCACCATGGTAATCA 5377

KY471092.2001 TTTTGCCAAAGTACATTGGGTTGGACCCGGTGGCTCCAGGAGACCTCACCATGGTAATCA 5377

MH481611.2017 TTTTGCCAAAGTACATTGGGTTGGACCCGGTGTCTCCAGGAGACCTCACCATGGTAATCA 5366

MH613311.2017 TTTTGCCAAAGTACATTGGGTTGGACCCGGTGTCTCCAGGAGACCTCACCATGGTAATCA 5345

KC242792.1994 TTTTGCCAAAGTACATTGGTTTGGACCCGGTGGCTCCAGGAGACCTCACCATGGTAATCA 5400

KC242793.1996 TTTTGCCAAAGTACATTGGTTTGGACCCGGTGGCTCCAGGAGACCTCACCATGGTAATCA 5400

KC242794.1996 TTTTGCCAAAGTACATTGGTTTGGACCCGGTGGCTCCAGGAGACCTCACCATGGTAATCA 5400

MH121164.1995 TTTTGCCAAAGTACATTGGTTTGGACCCGGTGGCTCCAGGAGACCTCACCATGGTAATCA 5400

AY354458.1995 TTTTGCCAAAGTACATTGGTTTGGACCCGGTGGCTCCAGGAGACCTCACCATGGTAATCA 5400

KT762962.1995 TTTTGCCAAAGTACATTGGTTTGGACCCGGTGGCTCCAGGAGACCTCACCATGGTAATCA 5400

HQ613402.2008 TTTTGCCAAAGTACATTGGGTTGGACCCGGTGGCTCCAGGAGACCTCACCATGGTAATCA 5319

KC242789.2007 TTTTGCCAAAGTACATTGGGTTGGACCCGGTGGCTCCAGGAGACCTCACCATGGTAATCA 5400

HQ613403.2007 TTTTGCCAAAGTACATTGGGTTGGACCCGGTGGCTCCAGGAGACCTCACCATGGTAATCA 5352

KC242785.2007 TTTTGCCAAAGTACATTGGGTTGGACCCGGTGGCTCCAGGAGACCTCACCATGGTAATCA 5400

KC242790.2007 TTTTGCCAAAGTACATTGGGTTGGACCCGGTGGCTCCAGGAGACCTCACCATGGTAATCA 5400

KU143789.2014 TTTTGCCAAAGTACATTGGGTTGGACCCGGTGGCTCCAGGAGACCTCACCATGGTAATCA 5400

KR817168.2014 TTTTGCCAAAGTACATTGGGTTGGACCCGGTGGCTCCAGGAGACCTCACCATGGTAATCA 5374

KY426696.2015 TTTTGCCAAAGTACATTGGGTTGGACCCGGTGGCTCCAGGAGACCTCACCATGGTAATCA 5400

KR105271.2014 TTTTGCCAAAGTACATTGGGTTGGACCCGGTGGCTCCAGGAGACCTCACCATGGTAATCA 5367

KY007522.2016 TTTTGCCAAAGTACATTGGGTTGGACCCGGTGGCTCCAGGAGACCTCACCATGGTAATCA 5380

KM034555.2014 TTTTGCCAAAGTACATTGGGTTGGACCCGGTGGCTCCAGGAGACCTCACCATGGTAATCA 5392

MH470381.2015 TTTTGCCAAAGTACATTGGGTTGGACCCGGTGGCTCCAGGAGACCTCACCATGGTAATCA 5364

MH470382.2015 TTTTGCCAAAGTACATTGGGTTGGACCCGGTGGCTCCAGGAGACCTCACCATGGTAATCA 5364

MF102255.2014 TTTTGCCAAAGTACATTGGGTTGGACCCGGTGGCTCCAGGAGACCTCACCATGGTAATCA 5377

KJ660348.2014 TTTTGCCAAAGTACATTGGGTTGGACCCGGTGGCTCCAGGAGACCTCACCATGGTAATCA 5400

KU143818.2014 TTTTGCCAAAGTACATTGGGTTGGACCCGGTGGCTCCAGGAGACCTCACCATGGTAATCA 5400

KT725333.2014 TTTTGCCAAAGTACATTGGGTTGGACCCGGTGGCTCCAGGAGACCTCACCATGGTAATCA 5365

KR819004.2014 TTTTGCCAAAGTACATTGGTTTGGACCCGGTGGCTCCAGGAGACCTCACCATGGTAATCA 5347

KP271020.2014 TTTTGCCAAAGTACATTGGTTTGGACCCGGTGGCTCCAGGAGACCTCACCATGGTAATCA 5347

KM519951.2014 TTTTGCCAAAGTACATTGGTTTGGACCCGGTGGCTCCAGGAGACCTCACCATGGTAATCA 5397

MH733488.2018 TTTTGCCAAAGTACATTGGTTTGGACCCGGTGGCTCCAGGAGACCTCACCATGGTAATCA 5391

MH733491.2018 TTTTGCCAAAGTACATTGGTTTGGACCCGGTGGCTCCAGGAGACCTCACCATGGTAATCA 5386

MH733478.2018 TTTTGCCAAAGTACATTGGTTTGGACCCGGTGGCTCCAGGAGACCTCACCATGGTAATCA 5389

MK007330.2018 TTTTGCCAAAGTACATTGGGTTGGACCCGGTGGCTCCAGGAGACCTCACCATGGTAATCA 5388

MK007344.2018 TTTTGCCAAAGTACATTGGGTTGGACCCGGTGGCTCCAGGAGACCTCACCATGGTAATCA 5382

******************* ************ ***************************

AY142960.1976 CACAGGATTGTGACACGTGTCATTCTCCTGCAAGTCTTCCAGCTGTGATTGAGAAGTAAT 5460 end VP40

KC242791.1977 CACAGGATTGTGACACGTGTCATTCTCCTGCAAGTCTTCCAGCTGTGATTGAGAAGTAAT 5460

AF499101.1976 CACAGGATTGTGACACGTGTCATTCTCCTGCAAGTCTTCCAGCTGTGATTGAGAAGTAAT 5460

KF113528.2003 CACAGGATTGTGACACGTGTCATTCTCCTGCAAGTCTTCCAGCTGTGATTGAGAAGTAAT 5456

KC242800.2002 CACAGGATTGTGACACGTGTCATTCTCCTGCAAGTCTTCCAGCTGTGATTGAGAAGTAAT 5460

KY471090.2001 CACAGGATTGTGACACGTGTCATTCTCCTGCAAGTCTTCCAGCTGTGATTGAGAAGTAAT 5437

KY471092.2001 CACAGGATTGTGACACGTGTCATTCTCCTGCAAGTCTTCCAGCTGTGATTGAGAAGTAAT 5437

MH481611.2017 CACAGGATTGTGACACGTGTCACTCTCCTGCAAGTCTTCCAGCTGTGATTGAGAAGTAAT 5426

MH613311.2017 CACAGGATTGTGACACGTGTCACTCTCCTGCAAGTCTTCCAGCTGTGATTGAGAAGTAAT 5405

KC242792.1994 CACAGGATTGTGACACGTGTCATTCTCCTGCGAGTCTTCCAGCTGTGATTGAGAAGTAAT 5460

KC242793.1996 CACAGGATTGTGACACGTGTCATTCTCCTGCGAGTCTTCCAGCTGTGATTGAGAAGTAAT 5460

KC242794.1996 CACAGGATTGTGACACGTGTCATTCTCCTGCGAGTCTTCCAGCTGTGATTGAGAAGTAAT 5460

MH121164.1995 CACAGGATTGTGACACGTGTCATTCTCCTGCGAGTCTTCCAGCTGTGATTGAGAAGTAAT 5460

AY354458.1995 CACAGGATTGTGACACGTGTCATTCTCCTGCGAGTCTTCCAGCTGTGATTGAGAAGTAAT 5460

KT762962.1995 CACAGGATTGTGACACGTGTCATTCTCCTGCGAGTCTTCCAGCTGTGATTGAGAAGTAAT 5460

HQ613402.2008 CACAGGATTGTGACACGTGTCATTCTCCTGCAAGTCTTCCAGCTGTGCTCGAGAAGTAAT 5379

KC242789.2007 CACAGGATTGTGACACGTGTCATTCTCCTGCAAGTCTTCCAGCTGTGCTCGAGAAGTAAT 5460

HQ613403.2007 CACAGGATTGTGACACGTGTCATTCTCCTGCAAGTCTTCCAGCTGTGCTCGAGAAGTAAT 5412

KC242785.2007 CACAGGATTGTGACACGTGTCATTCTCCTGCAAGTCTTCCAGCTGTGCTCGAGAAGTAAT 5460

KC242790.2007 CACAGGATTGTGACACGTGTCATTCTCCTGCAAGTCTTCCAGCTGTGCTCGAGAAGTAAT 5460

KU143789.2014 CACAGGATTGTGACACGTGTCATTCTCCTGCAAGTCTTCCAGCTGTGGTTGAGAAGTAAT 5460

KR817168.2014 CACAGGATTGTGACACGTGTCATTCTCCTGCAAGTCTTCCAGCTGTGGTTGAGAAGTAAT 5434

KY426696.2015 CACAGGATTGTGACACGTGTCATTCTCCTGCAAGTCTTCCAGCTGTGGTTGAGAAGTAAT 5460

KR105271.2014 CACAGGATTGTGACACGTGTCATTCTCCTGCAAGTCTTCCAGCTGTGGTTGAGAAGTAAT 5427

KY007522.2016 CACAGGATTGTGACACGTGTCATTCTCCTGCAAGTCTTCCAGCTGTGGTTGAGAAGTAAT 5440

KM034555.2014 CACAGGATTGTGACACGTGTCATTCTCCTGCAAGTCTTCCAGCTGTGGTTGAGAAGTAAT 5452

MH470381.2015 CACAGGATTGTGACACGTGTCATTCTCCTGCAAGTCTTCCAGCTGTGGTTGAGAAGTAAT 5424

MH470382.2015 CACAGGATTGTGACACGTGTCATTCTCCTGCAAGTCTTCCAGCTGTGGTTGAGAAGTAAT 5424

MF102255.2014 CACAGGATTGTGACACGTGTCATTCTCCTGCAAGTCTTCCAGCTGTGGTTGAGAAGTAAT 5437

KJ660348.2014 CACAGGATTGTGACACGTGTCATTCTCCTGCAAGTCTTCCAGCTGTGGTTGAGAAGTAAT 5460

KU143818.2014 CACAGGATTGTGACACGTGTCATTCTCCTGCAAGTCTTCCAGCTGTGGTTGAGAAGTAAT 5460

KT725333.2014 CACAGGATTGTGACACGTGTCATTCTCCTGCAAGTCTTCCAGCTGTGGTTGAGAAGTAAT 5425

KR819004.2014 CACAGGATTGTGGCACGTGTCATTCTCCTGCGAGTCTTCCAGCTGTGATTGAGAAGTAAT 5407

KP271020.2014 CACAGGATTGTGGCACGTGTCATTCTCCTGCGAGTCTTCCAGCTGTGATTGAGAAGTAAT 5407

KM519951.2014 CACAGGATTGTGGCACGTGTCATTCTCCTGCGAGTCTTCCAGCTGTGATTGAGAAGTAAT 5457

MH733488.2018 CACAGGATTGTGACACGTGTCATTCTCCTGCGAGTCTTCCAGCTGTGATTGAGAAGTAAT 5451

MH733491.2018 CACAGGATTGTGACACGTGTCATTCTCCTGCGAGTCTTCCAGCTGTGATTGAGAAGTAAT 5446

MH733478.2018 CACAGGATTGTGACACGTGTCATTCTCCTGCGAGTCTTCCAGCTGTGATTGAGAAGTAAT 5449

MK007330.2018 CACAGGATTGTGACACGTGTCATTCTCCTGCAAGTCTTCCAGCTGTGATTGAGAAGTAAT 5448

MK007344.2018 CACAGGATTGTGACACGTGTCATTCTCCTGCAAGTCTTCCAGCTGTGATTGAGAAGTAAT 5442

************ ********* ******** *************** * **********

AY142960.1976 TGCAATAATTGACTCAGATCCAGTTTTATAGAATCTTCTCAGGGATAGTGATAACATCTA 5520 -> F primer urGP

KC242791.1977 TGCAATAATTGACTCAGATCCAGTTTTATAGAATCTTCTCAGGGATAGTGATAACATCTA 5520

AF499101.1976 TGCAATAATTGACTCAGATCCAGTTTTATAGAATCTTCTCAGGGATAGTGATAACATCTA 5520

KF113528.2003 TGCAATAATTGACTCAGATCCAGTTTTACAGAATCTTCTCAGGGATAGTGATAACATCTA 5516

KC242800.2002 TGCAATAATTGACTCAGATCCAGTTTTACAGAATCTTCTCAGGGATAGTGATAACATCTA 5520

KY471090.2001 TGCAATAATTGACTCAGATCCAGTTTTACAGAATCTTCTCAGGGATAGTGATAACATCTA 5497

KY471092.2001 TGCAATAATTGACTCAGATCCAGTTTTACAGAATCTTCTCAGGGATAGTGATAACATCTA 5497

MH481611.2017 TGCAATAATTGACTCAGATCCAGTTTTACAGAATCTTCTCAGGGATAGTGATAACATCTA 5486

MH613311.2017 TGCAATAATTGACTCAGATCCAGTTTTACAGAATCTTCTCAGGGATAGTGATAACATCTA 5465

KC242792.1994 TGCAATAATTGACTCAGATCCAGTTTTACAGAATCTTCTCAGGGATAGTGATAACATCTA 5520

KC242793.1996 TGCAATAATTGACTCAGATCCAGTTTTACAGAATCTTCTCAGGGATAGTGATAACATCTA 5520

KC242794.1996 TGCAATAATTGACTCAGATCCAGTTTTACAGAATCTTCTCAGGGATAGTGATAACATCTA 5520

MH121164.1995 TGCAATAATTGACTCAGATCCAGTTTTACAGAATCTTCTCAGGGATAGTGATAACATCTA 5520

AY354458.1995 TGCAATAATTGACTCAGATCCAGTTTTACAGAATCTTCTCAGGGATAGTGATAACATCTA 5520

KT762962.1995 TGCAATAATTGACTCAGATCCAGTTTTACAGAATCTTCTCAGGGATAGTGATAACATCTA 5520

HQ613402.2008 TGCAATAATTGACTCAGATCCAGTTTTACAGAATCTTCTCAGGGATAGTGGTAACATCTA 5439

KC242789.2007 TGCAATAATTGACTCAGATCCAGTTTTACAGAATCTTCTCAGGGATAGTGGTAACATCTA 5520

HQ613403.2007 TGCAATAATTGACTCAGATCCAGTTTTACAGAATCTTCTCAGGGATAGTGGTAACATCTA 5472

KC242785.2007 TGCAATAATTGACTCAGATCCAGTTTTACAGAATCTTCTCAGGGATAGTGGTAACATCTA 5520

KC242790.2007 TGCAATAATTGACTCAGATCCAGTTTTACAGAATCTTCTCAGGGATAGTGGTAACATCTA 5520

KU143789.2014 TGCAATAATTGACTCAGATCCAGTTTTACAGAATCTTCTCAGGGATAGTGATAACATCTT 5520

KR817168.2014 TGCAATAATTGACTCAGATCCAGTTTTACAGAATCTTCTCAGGGATAGTGATAACATCTT 5494

KY426696.2015 TGCAATAATTGACTCAGATCCAGTTTTACAGAATCTTCTCAGGGATAGTGATAACATCTT 5520

KR105271.2014 TGCAATAATTGACTCAGATCCAGTTTTACAGAATCTTCTCAGGGATAGTGATAACATCTT 5487

KY007522.2016 TGCAATAATTGACTCAGATCCAGTTTTACAGAATCTTCTCAGGGATAGTGATAACATCTT 5500

KM034555.2014 TGCAATAATTGACTCAGATCCAGTTTTACAGAATCTTCTCAGGGATAGTGATAACATCTT 5512

MH470381.2015 TGCAATAATTGACTCAGATCCAGTTTTACAGAATCTTCTCAGGGATAGTGATAACATCTT 5484

MH470382.2015 TGCAATAATTGACTCAGATCCAGTTTTACAGAATCTTCTCAGGGATAGTGATAACATCTT 5484

MF102255.2014 TGCAATAATTGACTCAGATCCAGTTTTACAGAATCTTCTCAGGGATAGTGATAACATCTT 5497

KJ660348.2014 TGCAATAATTGACTCAGATCCAGTTTTACAGAATCTTCTCAGGGATAGTGATAACATCTT 5520

KU143818.2014 TGCAATAATTGACTCAGATCCAGTTTTACAGAATCTTCTCAGGGATAGTGATAACATCTT 5520

KT725333.2014 TGCAATAATTGACTCAGATCCAGTTTTACAGAATCTTCTCAGGGATAGTGATAACATCTT 5485

KR819004.2014 TGCAATAATTGACTCAGATCCAGTTTTACAGAATCTTCTCAGGGATAGTGATAACATCTA 5467

KP271020.2014 TGCAATAATTGACTCAGATCCAGTTTTACAGAATCTTCTCAGGGATAGTGATAACATCTA 5467

KM519951.2014 TGCAATAATTGACTCAGATCCAGTTTTACAGAATCTTCTCAGGGATAGTGATAACATCTA 5517

MH733488.2018 TGCAATAATTGACTCAGATCCAGTTTTACAGAATCTTCTCAGGGATAGTGATAATATCTA 5511

MH733491.2018 TGCAATAATTGACTCAGATCCAGTTTTACAGAATCTTCTCAGGGATAGTGATAATATCTA 5506

MH733478.2018 TGCAATAATTGACTCAGATCCAGTTTTACAGAATCTTCTCAGGGATAGTGATAATATCTA 5509

MK007330.2018 TGCAATAATTGACTCAGATCCAGTTTTACAGAATCTTCTCAGGGATAGTGACAACATCTA 5508

MK007344.2018 TGCAATAATTGACTCAGATCCAGTTTTACAGAATCTTCTCAGGGATAGTGACAACATCTA 5502

**************************** ********************* ** ****

AY142960.1976 TTTAGTAATCCGTCCATTAGAGGAGACACTTTTAATTGATCAATATACTAAAGGTGCTTT 5580

KC242791.1977 TTTAGTAATCCGTCCATTAGAGGAGACACTTTTAATTGATCAATATACTAAAGGTGCTTT 5580

AF499101.1976 TTTAGTAATCCGTCCATTAGAGGAGACACTTTTAATTGATCAATATACTAAAGGTGCTTT 5580

KF113528.2003 TTTAGTAATCCGTCTATTAGAGGAGATACTTTTAATCGATCAATATACTAAAGGTGCTTT 5576

KC242800.2002 TTTAGTAATCCGTCTATTAGAGGAGATACTTTTAATTGATCAATATACTAAAGGTGCTTT 5580

KY471090.2001 TTTAGTAATCCGTCTATTAGAGGAGATACTTTTAATTGATCAATATACTAAAGGTGCTTT 5557

KY471092.2001 TTTAGTAATCCGTCTATTAGAGGAGATACTTTTAATTGATCAATATACTAAAGGTGCTTT 5557

MH481611.2017 TTCAGTAATCCGTCCATTAGAGGAGATACTTTTAATTGATCAATATACTAAAGGTGCTTT 5546

MH613311.2017 TTCAGTAATCCGTCCATTAGAGGAGATACTTTTAATTGATCAATATACTAAAGGTGCTTT 5525

KC242792.1994 TTTAGTAATCCGTCCATTAGAGGAGATACTTTAAATTGATCGATATACTAAAGGTGCTTT 5580

KC242793.1996 TTTAGTAATCCGTCCATTAGAGGAGATACTTTAAATTGATCGATATACTAAAGGTGCTTT 5580

KC242794.1996 TTTAGTAATCCGTCCATTAGAGGAGATACTTTAAATTGATCAATATACTAAAGGTGCTTT 5580

MH121164.1995 TTTAGTAATCCGTCCATTAGAGGAGATACTTTTAATTGATCAATATACTAAAGGTGCTTT 5580

AY354458.1995 TTTAGTAATCCGTCCATTAGAGGAGATACTTTTAATTGATCAATATACTAAAGGTGCTTT 5580

KT762962.1995 TTTAGTAATCCGTCCATTAGAGGAGATACTTTTAATTGATCAATATACTAAAGGTGCTTT 5580

HQ613402.2008 TTTAGTAATCCGTCTATTAGAGGAGATACTTTTAATTGATCAATATACTAAAGGTGCTTT 5499

KC242789.2007 TTTAGTAATCCGTCTATTAGAGGAGATACTTTTAATTGATCAATATACTAAAGGTGCTTT 5580

HQ613403.2007 TTTAGTAATCCGTCTATTAGAGGAGATACTTTTAATTGATCAATATACTAAAGGTGCTTT 5532

KC242785.2007 TTTAGTAATCCGTCTATTAGAGGAGATACTTTTAATTGATCAATATACTAAAGGTGCTTT 5580

KC242790.2007 TTTAGTAATCCGTCTATTAGAGGAGATACTTTTAATTGATCAATATACTAAAGGTGCTTT 5580

KU143789.2014 TTTAATAATCCGTCTACTAGAAGAGATACTTCTAATTGATCAATATACTAAAGGTGCTTT 5580

KR817168.2014 TTTAATAATCCGTCTACTAGAAGAGATACTTCTAATTGATCAATATACTAAAGGTGCTTT 5554

KY426696.2015 TTTAATAATCCGTCTACTAGAAGAGATACTTCTAATTGATCAATATACTAAAGGTGCTTT 5580

KR105271.2014 TTTAATAATCCGTCTACTAGAAGAGATACTTCTAATTGATCAATATACTAAAGGTGCTTT 5547

KY007522.2016 TTTAATAATCCGTCTACTAGAAGAGATACTTCTAATTGATCAATATACTAAAGGTGCTTT 5560

KM034555.2014 TTTAATAATCCGTCTACTAGAAGAGATACTTCTAATTGATCAATATACTAAAGGTGCTTT 5572

MH470381.2015 TTTAATAATCCGTCTACTAGAAGAGATACTTCTAATTGATCAATATACTAAAGGTGCTTT 5544

MH470382.2015 TTTAATAATCCGTCTACTAGAAGAGATACTTCTAATTGATCAATATACTAAAGGTGCTTT 5544

MF102255.2014 TTTAATAATCCGTCTACTAGAAGAGATACTTCTAATTGATCAATATACTAAAGGTGCTTT 5557

KJ660348.2014 TTTAATAATCCGTCTACTAGAAGAGATACTTCTAATTGATCAATATACTAAAGGTGCTTT 5580

KU143818.2014 TTTAATAATCCGTCTACTAGAAGAGATACTTCTAATTGATCAATATACTAAAGGTGCTTT 5580

KT725333.2014 TTTAATAATCCGTCTACTAGAAGAGATACTTCTAATTGATCAATATACTAAAGGTGCTTT 5545

KR819004.2014 TTTAGTAATCCGTCCATTAGAGGAGATACTTTTGATTGATCAATATACTAAAGGTGCTTT 5527

KP271020.2014 TTTAGTAATCCGTCCATTAGAGGAGATACTTTTGATTGATCAATATACTAAAGGTGCTTT 5527

KM519951.2014 TTTAGTAATCCGTCCATTAGAGGAGATACTTTTGATTGATCAATATACTAAAGGTGCTTT 5577

MH733488.2018 TTTAGTAATTCGTCCATTAGAGGAGATACTTTTGATTGATCAATATACTAAAGGTGCTTT 5571

MH733491.2018 TTTAGTAATTCGTCCATTAGAGGAGATACTTTTGATTGATCAATATACTAAAGGTGCTTT 5566

MH733478.2018 TTTAGTAATTCGTCCATTAGAGGAGATACTTTTGATTGATCAATATACTAAAGGTGCTTT 5569

MK007330.2018 TTTAGTAATCCGTCCATTAGCGGAGATACTTTTAATTGATCAATATACTAAAGGTGCTTT 5568

MK007344.2018 TTTAGTAATCCGTCCATTAGCGGAGATACTTTTAATTGATCAATATACTAAAGGTGCTTT 5562

** * **** **** * *** **** **** ** **** ******************

AY142960.1976 ACACCATTGTCTTTTTTCTCTCCTAAATGTAGAACTTAACAAAAGACTCATAATATACTT 5640

KC242791.1977 ACACCATTGTCTTTTTTCTCTCCTAAATGTAGAACTTAACAAAAGACTCATAATATACTT 5640

AF499101.1976 ACACCATTGTCTTTTTTCTCTCCTAAATGTAGAACTTAACAAAAGACTCATAATATACTT 5640

KF113528.2003 ACACCATTGTCTTTTTTCTCTCCTAAATGTAGAACTTAACAAAAGACTCACAATATACTT 5636

KC242800.2002 ACACCATTGTCTTTTTTCTCTCCTAAATGTAGAACTTAACAAAAGACTCACAATATACTT 5640

KY471090.2001 ACACCATTGTCTTTTTTCTCTCCTAAATGTAGAACTTAACAAAAGACTCACAATATACTT 5617

KY471092.2001 ACACCATTGTCTTTTTTCTCTCCTAAATGTAGAACTTAACAAAAGACTCACAATATACTT 5617

MH481611.2017 ACACCATTGTCTTTTTTCTCTACTAAATGTAGAACTTAACAAAAGACTCATAATATACTT 5606

MH613311.2017 ACACCATTGTCTTTTTTCTCTACTAAATGTAGAACTTAACAAAAGACTCATAATATACTT 5585

KC242792.1994 ACACCATTGTCTTTTTTCTCTCCTAAATGTAGAACTTAACAAAAGACTCATAATATACTT 5640

KC242793.1996 ACACCATTGTCTTTTTTCTCTCCTAAATGTAGAACTTAACAAAAGACTCATAATATACTT 5640

KC242794.1996 ACACCATTGTCTTTTTTCTCTCCTAAATGTAGAACTTAACAAAAGACTCATAATATACTT 5640

MH121164.1995 ACACCATTGTCTTTTTCCTCTCCTAAATGTAGAACTTAACAAAAGACTCATAATATACTT 5640

AY354458.1995 ACACCATTGTCTTTTTCCTCTCCTAAATGTAGAACTTAACAAAAGACTCATAATATACTT 5640

KT762962.1995 ACACCATTGTCTTTTTCCTCTCCTAAATGTAGAACTTAACAAAAGACTCATAATATACTT 5640

HQ613402.2008 ACACCGTTGTCTCTTTTTTCTCCTAAATGTAGAACTTAACAAAAAACTCATAATATACTT 5559

KC242789.2007 ACACCGTTGTCTCTTTTTTCTCCTAAATGTAGAACTTAACAAAAAACTCATAATATACTT 5640

HQ613403.2007 ACACCGTTGTCTCTTTTTTCTCCTAAATGTAGAACTTAACAAAAAACTCATAATATACTT 5592

KC242785.2007 ACACCGTTGTCTCTTTTTTCTCCTAAATGTAGAACTTAACAAAAAACTCATAATATACTT 5640

KC242790.2007 ACACCGTTGTCTCTTTTTTCTCCTAAATGTAGAACTTAACAAAAAACTCATAATATACTT 5640

KU143789.2014 ACACCATTGTCTCTTTTCTCTCCTAAATGTAGAGCTTAACAAAAGACTCATAATATACCT 5640

KR817168.2014 ACACCATTGTCTCTTTTCTCTCCTAAATGTAGAGCTTAACAAAAGACTCATAATATACCT 5614

KY426696.2015 ACACCATTGTCTCTTTTCTCTCCTAAATGTAGAGCTTAACAAAAGACTCATAATATACCT 5640

KR105271.2014 ACACCATTGTCTCTTTTCTCTCCTAAATGTAGAGCTTAACAAAAGACTCATAATATACCT 5607

KY007522.2016 ACACCATTGTCTCTTTTCTCTCCTAAATGTAGAGCTTAACAAAAGACTCATAATATACCT 5620

KM034555.2014 ACACCATTGTCTCTTTTCTCTCCTAAATGTAGAGCTTAACAAAAGACTCATAATATACCT 5632

MH470381.2015 ACACCATTGTCTCTTTTCTCTCCTAAATGTAGAGCTTAACAAAAGACTCATAATATACCT 5604

MH470382.2015 ACACCATTGTCTCTTTTCTCTCCTAAATGTAGAGCTTAACAAAAGACTCATAATATACCT 5604

MF102255.2014 ACACCATTGTCTCTTTTCTCTCCTAAATGTAGAGCTTAACAAAAGACTCATAATATACCT 5617

KJ660348.2014 ACACCATTGTCTCTTTTCTCTCCTAAATGTAGAGCTTAACAAAAGACTCATAATATACCT 5640

KU143818.2014 ACACCATTGTCTCTTTTCTCTCCTAAATGTAGAGCTTAACAAAAGACTCATAATATACCT 5640

KT725333.2014 ACACCATTGTCTCTTTTCTCTCCTAAATGTAGAGCTTAACAAAAGACTCATAATATACCT 5605

KR819004.2014 ACACCATTGTCTTTTTCCTCTCCTAAATGTAGAACTTAACAAAAGACTCATAATATACTC 5587

KP271020.2014 ACACCATTGTCTTTTTCCTCTCCTAAATGTAGAACTTAACAAAAGACTCATAATATACTC 5587

KM519951.2014 ACACCATTGTCTTTTTCCTCTCCTAAATGTAGAACTTAACAAAAGACTCATAATATACTC 5637

MH733488.2018 ACACCATTGTCTTTTTTCTCTCCTAAATGTAGAACTTAACAAAAGACTCATAATATACTT 5631

MH733491.2018 ACACCATTGTCTTTTTTCTCTCCTAAATGTAGAACTTAACAAAAGACTCATAATATACTT 5626

MH733478.2018 ACACCATTGTCTTTTTTCTCTCCTAAATGTAGAACTTAACAAAAGACTCATAATATACTT 5629

MK007330.2018 ACACCATTGTCTTTTTCCTCTCCTAAATGTAGAACTTAACAAAAGACTCATAATATACTT 5628

MK007344.2018 ACACCATTGTCTTTTTCCTCTCCTAAATGTAGAACTTAACAAAAGACTCATAATATACTT 5622

***** ****** *** *** *********** ********** ***** *******

AY142960.1976 GTTTTTAAAGGATTGATTGATGAAAGATCATAACTAATAACATTACAAATAATCCTACTA 5700

KC242791.1977 GTTTTTAAAGGATTGATTGATGAAAGATCATAACTAATAACATTACAAATAATCCTACTA 5700

AF499101.1976 GTTTTTAAAGGATTGATTGATGAAAGATCATAACTAATAACATTACAAATAATCCTACTA 5700

KF113528.2003 GTCTTAAAGAGATTGATTGATGAAAGATCAAGACTAATAACATTACAAATAATCCTACTA 5696

KC242800.2002 GTCTTAAAGAGATTGATTGATGAAAGATCATGACTAATAACATTACAAATAATCCTACTA 5700

KY471090.2001 GTCTTAAAGAGATTGATTGATGAAAGATCATGACTAATAACATTACAAATAATCCTACTA 5677

KY471092.2001 GTCTTAAAGAGATTGATTGATGAAAGATCATGACTAATAACATTACAAATAATCCTACTA 5677

MH481611.2017 GTTTTTAAAAGATTGATTGATGGAAGATCATAACTAATAACATTACAAATAATCCTACTA 5666

MH613311.2017 GTTTTTAAAAGATTGATTGATGGAAGATCATAACTAATAACATTACAAATAATCCTACTA 5645

KC242792.1994 GGTTTTAAAAGATTGATTGATAGAAGATCATAACTAATAACATTACAAATAATCCTACTA 5700

KC242793.1996 GGTTTTAAAAGATTGATTGATAGAAGATCATAACTAATAACATTACAAATAATCCTACTA 5700

KC242794.1996 GGTTTTAAAAGATTGATTGATAGAAGATCATAACTAATAACATTACAAATAATCCTACTA 5700

MH121164.1995 GTTTTTAAAAGATTGATTGATGAAAGATCATAACTAATAACATTACAAATAATCCTACTA 5700

AY354458.1995 GTTTTTAAAAGATTGATTGATGAAAGATCATAACTAATAACATTACAAATAATCCTACTA 5700

KT762962.1995 GTTTTTAAAAGATTGATTGATGAAAGATCATAACTAATAACATTACAAATAATCCTACTA 5700

HQ613402.2008 GTCTTTAAAAGATTGATTGATGAAAGATCATGACTAATAACATTACAAATAATCCTACTA 5619

KC242789.2007 GTCTTTAAAAGATTGATTGATGAAAGATCATGACTAATAACATTACAAATAATCCTACTA 5700

HQ613403.2007 GTCTTTAAAAGATTGATTGATGAAAGATCATGACTAATAACATTACAAATAATCCTACTA 5652

KC242785.2007 GTCTTTAAAAGATTGATTGATGAAAGATCATGACTAATAACATTACAAATAATCCTACTA 5700

KC242790.2007 GTCTTTAAAAGATTGATTGATGAAAGATCATGACTAATAACATTACAAATAATCCTACTA 5700

KU143789.2014 GTTTTTAAAAGATTGATTGATGAAAGATCATGACTAATAACATTACAAACAATCCTACTA 5700

KR817168.2014 GTTTTTAAAAGATTGATTGATGAAAGATCATGACTAATAACATTACAAACAATCCTACTA 5674

KY426696.2015 GTTTTTAAAAGATTGATTGATGAAAGATCATGACTAATAACATTACAAACAATCCTACTA 5700

KR105271.2014 GTTTTTAAAAGATTGATTGATGAAAGATCATGACTAATAACATTACAAACAATCCTACTA 5667

KY007522.2016 GTTTTTAAAAGATTGATTGATGAAAGATCATGACTAATAACATTACAAACAATCCTACTA 5680

KM034555.2014 GTTTTTAAAAGATTGATTGATGAAAGATCATGACTAATAACATTACAAACAATCCTACTA 5692

MH470381.2015 GTTTTTAAAAGATTGATTGATGAAAGATCATGACTAATAACATTACAAACAATCCTACTA 5664

MH470382.2015 GTTTTTAAAAGATTGATTGATGAAAGATCATGACTAATAACATTACAAACAATCCTACTA 5664

MF102255.2014 GTTTTTAAAAGATTGATTGATGAAAGATCATGACTAATAACATTACAAACAATCCTACTA 5677

KJ660348.2014 GTTTTTAAAAGATTGATTGATGAAAGATCATGACTAATAACATTACAAACAATCCTACTA 5700

KU143818.2014 GTTTTTAAAAGATTGATTGATGAAAGATCATGACTAATAACATTACAAACAATCCTACTA 5700

KT725333.2014 GTTTTTAAAAGATTGATTGATGAAAGATCATGACTAATAACATTACAAACAATCCTACTA 5665

KR819004.2014 GTTTTTAAAAGATTGATTGATGAAAGATCATAACTAATAACATTACAAATAATCCTACTA 5647

KP271020.2014 GTTTTTAAAAGATTGATTGATGAAAGATCATAACTAATAACATTACAAATAATCCTACTA 5647

KM519951.2014 GTTTTTAAAAGATTGATTGATGAAAGATCATAACTAATAACATTACAAATAATCCTACTA 5697

MH733488.2018 GTTTTTAAAAGATTGATTGATGAAAGATCATAACTAATAACATCACAAATAATCCTACTA 5691

MH733491.2018 GTTTTTAAAAGATTGATTGATGAAAGATCATAACTAATAACATCACAAATAATCCTACTA 5686

MH733478.2018 GTTTTTAAAAGATTGATTGATGAAAGATCATAACTAATAACATCACAAATAATCCTACTA 5689

MK007330.2018 GTTTTAAACAGATTGATTGATGAAAGATCATAACTAATAACATTACAAATAATCCTACTA 5688

MK007344.2018 GTTTTAAACAGATTGATTGATGAAAGATCATAACTAATAACATTACAAATAATCCTACTA 5682

* ** ** *********** ******* *********** ***** **********

AY142960.1976 TAATCAATACGGTGATTCAAATGTTAATCTTTCTCATTGCACATACTTTTTGCCCTTATC 5760

KC242791.1977 TAATCAATACGGTGATTCAAATGTTAATCTTTCTCATTGCACATACTTTTTGCCCTTATC 5760

AF499101.1976 TAATCAATACGGTGATTCAAATGTTAATCTTTCTCATTGCACATACTTTTTGCCCTTATC 5760

KF113528.2003 TAATCAATACGGTGATTCAAATATTAATCTTTCTAATTGCACATACTCTCTGCCCCCATC 5756

KC242800.2002 TAATCAATACGGTGATTCAAATATTAATCTTTCTAATTGCACATACTCTCTGCCCCTATC 5760

KY471090.2001 TAATCAATACGGTGATTCAAATATTAATCTTTCTAATTGCACATACTCTCTGCCCCTATC 5737

KY471092.2001 TAATCAATACGGTGATTCAAATATTAATCTTTCTAATTGCACATACTCTCTGCCCCTATC 5737

MH481611.2017 TAATCAATGCGGTGATTCAAATGTTAATCTTTCTCATTGCACATACTCTTTGCCCTTATC 5726

MH613311.2017 TAATCAATGCGGTGATTCAAATGTTAATCTTTCTCATTGCACATACTCTTTGCCCTTATC 5705

KC242792.1994 TAATCAATACGGTGATTCAAATGTTAATCTTTCTCATTGCACATACTCTTTGCCCTTATC 5760

KC242793.1996 TAATCAATACGGTGATTCAAATGTTAATCTTTCTCATTGCGCATACTCTTTGCCCTTATC 5760

KC242794.1996 TAATCAATACGGTGATTCAAATGTTAATCTTTCTCATTGCACATACTCTTTGCCCTTATC 5760

MH121164.1995 TAATCAATACGGTGATCCAAATGTTAATCTTTCTCATTGCAAATACTCTTTGCCCTTATC 5760

AY354458.1995 TAATCAATACGGTGATCCAAATGTTAATCTTTCTCATTGCAAATACTCTTTGCCCTTATC 5760

KT762962.1995 TAATCAATACGGTGATCCAAATGTTAATCTTTCTCATTGCAAATACTCTTTGCCCTTATC 5760

HQ613402.2008 TAATCAATACGGTGATTCAAATGTTAATCTTTCTCATTGCACATACTCTTTGCCCTTATC 5679

KC242789.2007 TAATCAATACGGTGATTCAAATGTTAATCTTTCTCATTGCACATACTCTTTGCCCTTATC 5760

HQ613403.2007 TAATCAATACGGTGATTCAAATGTTAATCTTTCTCATTGCACATACTCTTTGCCCTTATC 5712

KC242785.2007 TAATCAATACGGTGATTCAAATGTTAATCTTTCTCATTGCACATACTCTTTGCCCTTATC 5760

KC242790.2007 TAATCAATACGGTGATTCAAATGTTAATCTTTCTCATTGCACATACTCTTTGCCCTTATC 5760

KU143789.2014 TAATCAATACGGTGATTCAAATGTCAATCTTTCTCATTGCACATACTCTTTGTCCTTATC 5760

KR817168.2014 TAATCAATACGGTGATTCAAATGTCAATCTTTCTCATTGCACATACTCTTTGTCCTTATC 5734

KY426696.2015 TAATCAATACGGTGATTCAAATGTCAATCTTTCTCATTGCACATACTCTTTGTCCTTATC 5760

KR105271.2014 TAATCAATACGGTGATTCAAATGTCAATCTTTCTCATTGCACATACTCTTTGTCCTTATC 5727

KY007522.2016 TAATCAATACGGTGATTCAAATGTCAATCTTTCTCATTGCACATACTCTTTGTCCTTATC 5740

KM034555.2014 TAATCAATACGGTGATTCAAATGTCAATCTTTCTCATTGCACATACTCTTTGTCCTTATC 5752

MH470381.2015 TAATCAATACGGTGATTCAAATGTCAATCTTTCTCATTGCACATACTCTTTGTCCTTATC 5724

MH470382.2015 TAATCAATACGGTGATTCAAATGTCAATCTTTCTCATTGCACATACTCTTTGTCCTTATC 5724

MF102255.2014 TAATCAATACGGTGATTCAAATGTCAATCTTTCTCATTGCACATACTCTTTGTCCTTATC 5737

KJ660348.2014 TAATCAATACGGTGATTCAAATGTCAATCTTTCTCATTGCACATACTCTTTGTCCTTATC 5760

KU143818.2014 TAATCAATACGGTGATTCAAATGTCAATCTTTCTCATTGCACATACTCTTTGTCCTTATC 5760

KT725333.2014 TAATCAATACGGTGATTCAAATGTCAATCTTTCTCATTGCACATACTCTTTGTCCTTATC 5725

KR819004.2014 TAATCAATACGGTGATTCAAATGTTAATCTTTCTCATTGCACATACTCTTTGCCCTTATC 5707

KP271020.2014 TAATCAATACGGTGATTCAAATGTTAATCTTTCTCATTGCACATACTCTTTGCCCTTATC 5707

KM519951.2014 TAATCAATACGGTGATTCAAATGTTAATCTTTCTCATTGCACATACTCTTTGCCCTTATC 5757

MH733488.2018 TAATCAATACGGTGATTCAAATGTTAATCTTTCTCATTGCACATACTCTTTGCCCTTATC 5751

MH733491.2018 TAATCAATACGGTGATTCAAATGTTAATCTTTCTCATTGCACATACTCTTTGCCCTTATC 5746

MH733478.2018 TAATCAATACGGTGATTCAAATGTTAATCTTTCTCATTGCACATACTCTTTGCCCTTATC 5749

MK007330.2018 TAATCAATACGGTGATTCAAATGTTAATCTTTCTCATTGCACATACTCTTTGCCCTTATC 5748

MK007344.2018 TAATCAATACGGTGATTCAAATGTTAATCTTTCTCATTGCACATACTCTTTGCCCTTATC 5742

******** ******* ***** * ********* ***** ***** * ** ** ***

AY142960.1976 CTCAAATTGCCTGCATGCTTACATCTGAGGATAGCCAGTGTGACTTGGATTGGAAATGTG 5820

KC242791.1977 CTCAAATTGCCTGCATGCTTACATCTGAGGATAGCCAGTGTGACTTGGATTGGAAATGTG 5820

AF499101.1976 CTCAAATTGCCTGCATGCTTACATCTGAGGATAGCCAGTGTGACTTGGATTGGAAATGTG 5820

KF113528.2003 CTCAAATTGCCTACATGCCTACATCTGAGGATAGCCAGTGTGACTTGGATTGGAGATGTA 5816

KC242800.2002 CTCAAATTGCCTACATGCCTACATCTGAGGATAGCCAGTGTGACTTGGATTGGAGATGTA 5820

KY471090.2001 CTCAAATTGCCTACATGCCTACATCTGAGGATAGCCAGTGTGACTTGGATTGGAGATGTA 5797

KY471092.2001 CTCAAATTGCCTACATGCCTACATCTGAGGATAGCCAGTGTGACTTGGATTGGAGATGTA 5797

MH481611.2017 CTCAAATTGACTGCATGCTTACATCTAAGGATAGCCAGTGTGACTTGGATTGGAGATGTG 5786

MH613311.2017 CTCAAATTGACTGCATGCTTACATCTAAGGATAGCCAGTGTGACTTGGATTGGAGATGTG 5765

KC242792.1994 CTCAAATTGCCTACATGCTTACATCTGAGGATAGCCAGTGTGACTTGGATTGGAGATGTG 5820

KC242793.1996 CTCAAATTGCCTACATGCTTACATCTGAGGATAGCCAGTGTGACTTGGATTGGAGATGTG 5820

KC242794.1996 CTCAAATTGCCTACATGCTTACATCTGAGGATAGCCAGTGTGACTTGGATTGGAGATGTG 5820

MH121164.1995 CTCAAATTGCCTACATGCTTACATCTGAGGATAGCCAGTGTGACTTGGATTGGAGATGTG 5820

AY354458.1995 CTCAAATTGCCTACATGCTTACATCTGAGGATAGCCAGTGTGACTTGGATTGGAGATGTG 5820

KT762962.1995 CTCAAATTGCCTACATGCTTACATCTGAGGATAGCCAGTGTGACTTGGATTGGAGATGTG 5820

HQ613402.2008 CTCAAATTGCCTACATGCTTACATTTGGGGATAACCAGTGTGACTTGGATTGGAGATGTG 5739

KC242789.2007 CTCAAATTGCCTACATGCTTACATTTGGGGATAACCAGTGTGACTTGGATTGGAGATGTG 5820

HQ613403.2007 CTCAAATTGCCTACATGCTTACATTTGGGGATAACCAGTGTGACTTGGATTGGAGATGTG 5772

KC242785.2007 CTCAAATTGCCTACATGCTTACATTTGGGGATAACCAGTGTGACTTGGATTGGAGATGTG 5820

KC242790.2007 CTCAAATTGCCTACATGCTTACATTTGGGGATAACCAGTGTGACTTGGATTGGAGATGTG 5820

KU143789.2014 CTCAAATTGCCTACATGCTTACATCTGAGGACAGCCAGTGTGACTTGGATTGGAGATGTG 5820

KR817168.2014 CTCAAATTGCCTACATGCTTACATCTGAGGACAGCCAGTGTGACTTGGATTGGAGATGTG 5794

KY426696.2015 CTCAAATTGCCTACATGCTTACATCTGAGGACAGCCAGTGTGACTTGGATTGGAGATGTG 5820

KR105271.2014 CTCAAATTGCCTACATGCTTACATCTGAGGACAGCCAGTGTGACTTGGATTGGAGATGTG 5787

KY007522.2016 CTCAAATTGCCTACATGCTTACATCTGAGGACAGCCAGTGTGACTTGGATTGGAGATGTG 5800

KM034555.2014 CTCAAATTGCCTACATGCTTACATCTGAGGACAGCCAGTGTGACTTGGATTGGAGATGTG 5812

MH470381.2015 CTCAAATTGCCTACATGCTTACATCTGAGGACAGCCAGTGTGACTTGGATTGGAGATGTG 5784

MH470382.2015 CTCAAATTGCCTACATGCTTACATCTGAGGACAGCCAGTGTGACTTGGATTGGAGATGTG 5784

MF102255.2014 CTCAAATTGCCTACATGCTTACATCTGAGGACAGCCAGTGTGACTTGGATTGGAGATGTG 5797

KJ660348.2014 CTCAAATTGCCTACATGCTTACATCTGAGGACAGCCAGTGTGACTTGGATTGGAGATGTG 5820

KU143818.2014 CTCAAATTGCCTACATGCTTACATCTGAGGACAGCCAGTGTGACTTGGATTGGAGATGTG 5820

KT725333.2014 CTCAAATTGCCTACATGCTTACATCTGAGGACAGCCAGTGTGACTTGGATTGGAGATGTG 5785

KR819004.2014 CTCAAATTGCCTACATGCTTACATCTGAGGATAGCCAGTGTGACTTGGATTGGAGATGTG 5767

KP271020.2014 CTCAAATTGCCTACATGCTTACATCTGAGGATAGCCAGTGTGACTTGGATTGGAGATGTG 5767

KM519951.2014 CTCAAATTGCCTACATGCTTACATCTGAGGATAGCCAGTGTGACTTGGATTGGAGATGTG 5817

MH733488.2018 CTCAAATTGCCTACATGCTTACATCTGAGGATAGCCAGTGTGACTTGGATTGGAGATGTG 5811

MH733491.2018 CTCAAATTGCCTACATGCTTACATCTGAGGATAGCCAGTGTGACTTGGATTGGAGATGTG 5806

MH733478.2018 CTCAAATTGCCTACATGCTTACATCTGAGGATAGCCAGTGTGACTTGGATTGGAGATGTG 5809

MK007330.2018 CTCAAACTGCCTGCATGCTTACATCTGAGGATAGCCAGTGTGACTTGGATTGGAGATGTG 5808

MK007344.2018 CTCAAACTGCCTGCATGCTTACATCTGAGGATAGCCAGTGTGACTTGGATTGGAGATGTG 5802

****** ** ** ***** ***** * *** * ******************** ****

AY142960.1976 GAGAAAAAATCGGGACCCATTTCTAGGTTGTTCACAATCCAAGTACAGACATTGCCCTTC 5880

KC242791.1977 GAGAAAAAATCGGGACCCATTTCTAGGTTGTTCACAATCCAAGTACAGACATTGCCCTTC 5880

AF499101.1976 GAGAAAAAATCGGGACCCATTTCTAGGTTGTTCACAATCCAAGTACAGACATTGCCCTTC 5880

KF113528.2003 GGGAAGAAATCGGAACCCATCTCCAGGTTGTTCACAATCCAAGCACAGACATCGCCCTTC 5876

KC242800.2002 GGGAAGAAATCGGAACCCATCTCCAGGTTGTTCACAATCCAAGCACAGACATCGCCCTTC 5880

KY471090.2001 GGGAAGAAATCGGAACCCATCTCCAGGTTGTTCACAATCCAAGCACAGACATCGCCCTTC 5857

KY471092.2001 GGGAAGAAATCGGAACCCATCTCCAGGTTGTTCACAATCCAAGCACAGACATCGCCCTTC 5857

MH481611.2017 GAGAAAAAATCGAGACCCATTTCTAGGTTGTTCACAATCCAAGTACAGACATTGCCCTTC 5846

MH613311.2017 GAGAAAAAATCGAGACCCATTTCTAGGTTGTTCACAATCCAAGTACAGACATTGCCCTTC 5825

KC242792.1994 GAGAAAAAATCGGGACCCATTTCTAGTTTGTTCACCATCCAAGTACAGACATTGCCCTTC 5880

KC242793.1996 GAGAAAAAATCGGGACCCATTTCTAGTTTGTTCACCATCCAAGTACAGACATTGCCCTTC 5880

KC242794.1996 GAGAAAAAATCGGGACCCATTTCTAGTTTGTTCACCATCCAAGTACAGACATTGCCCTTC 5880

MH121164.1995 GAGAAAAAATCGGGACCCATTTCTAGGTTGTTCACCATCCAAGTACAGACATTGCCCTTC 5880

AY354458.1995 GAGAAAAAATCGGGACCCATTTCTAGGTTGTTCACCATCCAAGTACAGACATTGCCCTTC 5880

KT762962.1995 GAGAAAAAATCGGGACCCATTTCTAGGTTGTTCACCATCCAAGTACAGACATTGCCCTTC 5880

HQ613402.2008 AAGAAAAAATCGGGACCCATTTCTAGGTTGTTCACAATCCAGGTACAGACATTGCCCTTC 5799

KC242789.2007 AAGAAAAAATCGGGACCCATTTCTAGGTTGTTCACAATCCAGGTACAGACATTGCCCTTC 5880

HQ613403.2007 AAGAAAAAATCGGGACCCATTTCTAGGTTGTTCACAATCCAGGTACAGACATTGCCCTTC 5832

KC242785.2007 AAGAAAAAATCGGGACCCATTTCTAGGTTGTTCACAATCCAGGTACAGACATTGCCCTTC 5880

KC242790.2007 AAGAAAAAATCGGGACCCATTTCTAGGTTGTTCACAATCCAGGTACAGACATTGCCCTTC 5880

KU143789.2014 GAGGAAAAATCGGGGCCCATTTCTAGGTTGTTCACAATCTAAGTACAGACATTGCTCTTC 5880

KR817168.2014 GAGGAAAAATCGGGGCCCATTTCTAAGTTGTTCACAATCTAAGTACAGACATTGCTCTTC 5854

KY426696.2015 GAGGAAAAATCGGGGCCCATTTCTAAGTTGTTCACAATCTAAGTACAGACATTGCTCTTC 5880

KR105271.2014 GAGGAAAAATCGGGGCCCATTTCTAAGTTGTTCACAATCTAAGTACAGACATTGCTCTTC 5847

KY007522.2016 GAGGAAAAATCGGGGCCCATTTCTAAGTTGTTCACAATCTAAGTACAGACATTGCTCTTC 5860

KM034555.2014 GAGGAAAAATCGGGGCCCATTTCTAAGTTGTTCACAATCTAAGTACAGACATTGCTCTTC 5872

MH470381.2015 GAGGAAAAATCGGGGCCCATTTCTAAGTTGTTCACAATCTAAGTACAGACATTGCTCTTC 5844

MH470382.2015 GAGGAAAAATCGGGGCCCATTTCTAAGTTGTTCACAATCTAAGTACAGACATTGCTCTTC 5844

MF102255.2014 GAGGAAAAATCGGGGCCCATTTCTAAGTTGTTCACAATCTAAGTACAGACATTGCTCTTC 5857

KJ660348.2014 GAGGAAAAATCGGGGCCCATTTCTAAGTTGTTCACAATCTAAGTACAGACATTGCTCTTC 5880

KU143818.2014 GAGGAAAAATCGGGGCCCATTTCTAAGTTGTTCACAATCTAAGTACAGACATTGCTCTTC 5880

KT725333.2014 GAGGAAAAATCGGGGCCCATTTCTAAGTTGTTCACAATCTAAGTACAGACATTGCTCTTC 5845

KR819004.2014 GAGAAAAAATCGGGACCCATTTCTAGGTTGTTCACCATCCAAGTACAGACATTGCCCTTC 5827

KP271020.2014 GAGAAAAAATCGGGACCCATTTCTAGGTTGTTCACCATCCAAGTACAGACATTGCCCTTC 5827

KM519951.2014 GAGAAAAAATCGGGACCCATTTCTAGGTTGTTCACCATCCAAGTACAGACATTGCCCTTC 5877

MH733488.2018 GAGAAAAAATCGGGACCCATTTCTAGGTTGTTCACCATCCAAGTACAGACATTGCCCTTC 5871

MH733491.2018 GAGAAAAAATCGGGACCCATTTCTAGGTTGTTCACCATCCAAGTACAGACATTGCCCTTC 5866

MH733478.2018 GAGAAAAAATCGGGACCCATTTCTAGGTTGTTCACCATCCAAGTACAGACATTGCCCTTC 5869

MK007330.2018 GAGAAAAAATCGGGACCCATTTCTAGGTTGTTCACAATCCAAGTACAGACATTGCCCTTC 5868

MK007344.2018 GAGAAAAAATCGGGACCCATTTCTAGGTTGTTCACAATCCAAGTACAGACATTGCCCTTC 5862

* * ****** ***** ** * ******** *** * * ******** ** ****

AY142960.1976 TAATTAAGAAAAAATCGGCGATGAAGATTAAGCCGACAGTGAGCGTAATCTTCATCTCTC 5940

KC242791.1977 TAATTAAGAAAAAATCGGCGATGAAGATTAAGCCGACAGTGAGCGTAATCTTCATCTCTC 5940

AF499101.1976 TAATTAAGAAAAAATCGGCGATGAAGATTAAGCCGACAGTGAGCGTAATCTTCATCTCTC 5940

KF113528.2003 TAATTAAGAAAAAATCGGCGATGAAGATTAAGCCGACAGTGAGCGCAATCTTCATCTCTC 5936

KC242800.2002 TAATTAAGAAAAAATCGGCGATGAAGATTAAGCCGACAGTGAGCGCAATCTTCATCTCTC 5940

KY471090.2001 TAATTAAGAAAAAATCGGCGATGAAGATTAAGCCGACAGTGAGCGCAATCTTCATCTCTC 5917

KY471092.2001 TAATTAAGAAAAAATCGGCGATGAAGATTAAGCCGACAGTGAGCGCAATCTTCATCTCTC 5917

MH481611.2017 TAATTAAGAAAAAATCGGCGATGAAGATTAAGCCGACAGTGAGCGTAATCTTCATCTCTC 5906

MH613311.2017 TAATTAAGAAAAAATCGGCGATGAAGATTAAGCCGACAGTGAGCGTAATCTTCATCTCTC 5885

KC242792.1994 TAATTAAGAAAAAATCGGCGATGAAGATTAAGCCGACAGTGAGCGTAATCTTCATCTCTC 5940

KC242793.1996 TAATTAAGAAAAAATCGGCGATGAAGATTAAGCCGACAGTGAGCGTAATCTTCATCTCTC 5940

KC242794.1996 TAATTAAGAAAAAATCGGCGATGAAGATTAAGCCGACAGTGAGCGTAATCTTCATCTCTC 5940

MH121164.1995 TAATTAAGAAAAAATCGGCGATGAAGATTAAGCCGACAGTGAGCGTAATCTTCATCTCTC 5940

AY354458.1995 TAATTAAGAAAAAATCGGCGATGAAGATTAAGCCGACAGTGAGCGTAATCTTCATCTCTC 5940

KT762962.1995 TAATTAAGAAAAAATCGGCGATGAAGATTAAGCCGACAGTGAGCGTAATCTTCATCTCTC 5940

HQ613402.2008 TAATTAAGAAAAAATCGGCGATGAAGATTAAGCCGACAGTGAGCGTAATCTTCATCTCTC 5859

KC242789.2007 TAATTAAGAAAAAATCGGCGATGAAGATTAAGCCGACAGTGAGCGTAATCTTCATCTCTC 5940

HQ613403.2007 TAATTAAGAAAAAATCGGCGATGAAGATTAAGCCGACAGTGAGCGTAATCTTCATCTCTC 5892

KC242785.2007 TAATTAAGAAAAAATCGGCGATGAAGATTAAGCCGACAGTGAGCGTAATCTTCATCTCTC 5940

KC242790.2007 TAATTAAGAAAAAATCGGCGATGAAGATTAAGCCGACAGTGAGCGTAATCTTCATCTCTC 5940

KU143789.2014 TAATTAAGAAAAAATCGGCGATGAAGATTAAGCCGACAGTGAGCGTAATCTTCATCTCTC 5940

KR817168.2014 TAATTAAGAAAAAATCGGCGATGAAGATTAAGCCGACAGTGAGCGTAATCTTCATCTCTC 5914

KY426696.2015 TAATTAAGAAAAAATCGGTGATGAAGATTAAGCCGACAGTGAGCGTAATCTTCATCTCTC 5940

KR105271.2014 TAATTAAGAAAAAATCGGCGATGAAGATTAAGCCGACAGTGAGCGTAATCTTCATCTCTC 5907

KY007522.2016 TAATTAAGAAAAAATCGGCGATGAAGATTAAGCCGACAGTGAGCGTAATCTTCATCTCTC 5920

KM034555.2014 TAATTAAGAAAAAATCGGCGATGAAGATTAAGCCGACAGTGAGCGTAATCTTCATCTCTC 5932

MH470381.2015 TAATTAAGAAAAAATCGGCGATGAAGATTAAGCCGACAGTGAGCGTAATCTTCATCTCTC 5904

MH470382.2015 TAATTAAGAAAAAATCGGCGATGAAGATTAAGCCGACAGTGAGCGTAATCTTCATCTCTC 5904

MF102255.2014 TAATTAAGAAAAAATCGGCGATGAAGATTAAGCCGACAGTGAGCGTAATCTTCATCTCTC 5917

KJ660348.2014 TAATTAAGAAAAAATCGGCGATGAAGATTAAGCCGACAGTGAGCGTAATCTTCATCTCTC 5940

KU143818.2014 TAATTAAGAAAAAATCGGCGATGAAGATTAAGCCGACAGTGAGCGTAATCTTCATCTCTC 5940

KT725333.2014 TAATTAAGAAAAAATCGGCGATGAAGATTAAGCCGACAGTGAGCGTAATCTTCATCTCTC 5905

KR819004.2014 TAATTAAGAAAAAATCGGCGATGAAGATTAAGCCGACAGTGAGCGTAATCTTCATCTCTC 5887

KP271020.2014 TAATTAAGAAAAAATCGGCGATGAAGATTAAGCCGACAGTGAGCGTAATCTTCATCTCTC 5887

KM519951.2014 TAATTAAGAAAAAATCGGCGATGAAGATTAAGCCGACAGTGAGCGTAATCTTCATCTCTC 5937

MH733488.2018 TAATTAAGAAAAAATCGGCGATGAAGATTAAGCCGACAGTGAGCGTAATCTTCATCTCTC 5931

MH733491.2018 TAATTAAGAAAAAATCGGCGATGAAGATTAAGCCGACAGTGAGCGTAATCTTCATCTCTC 5926

MH733478.2018 TAATTAAGAAAAAATCGGCGATGAAGATTAAGCCGACAGTGAGCGTAATCTTCATCTCTC 5929

MK007330.2018 TAATTAAGAAAAAATCGGCGATGAAGATTAAGCCGACAGTGAGCGTAATCTTCATCTCTC 5928

MK007344.2018 TAATTAAGAAAAAATCGGCGATGAAGATTAAGCCGACAGTGAGCGTAATCTTCATCTCTC 5922

****************** ************************** **************

AY142960.1976 TTAGATTATTTGTTTTCCAGAGTAGGGGTCGTCAGGTCCTTTTCAATCGTGTAACCAAAA 6000

KC242791.1977 TTAGATTATTTGTTTTCCAGAGTAGGGGTCGTCAGGTCCTTTTCAATCGTGTAACCAAAA 6000

AF499101.1976 TTAGATTATTTGTTTTCCAGAGTAGGGGTCGTCAGGTCCTTTTCAATCGTGTAACCAAAA 6000

KF113528.2003 TTAGATTATTTGTTTTCCAGAGTAGGGGTCATCAGGTCCTTTCCAATCATATAACCAAAA 5996

KC242800.2002 TTAGATTATTTGTTTTCCAGAGTAGGGGTCATCAGGTCCTTTCCAATCATATAACCAAAA 6000

KY471090.2001 TTAGATTATTTGTTTTCCAGAGTAGGGGTCATCAGGTCCTTTCCAATCATATAACCAAAA 5977

KY471092.2001 TTAGATTATTTGTTTTCCAGAGTAGGGGTCATCAGGTCCTTTCCAATCATATAACCAAAA 5977

MH481611.2017 TTAGATTATTTGTTTTCTAGAGTAGGGGTCGTCAGGTCCTTTTCAATCGTATAACCAGAA 5966

MH613311.2017 TTAGATTATTTGTTTTCTAGAGTAGGGGTCGTCAGGTCCTTTTCAATCGTATAACCAGAA 5945

KC242792.1994 TTAGATTATTTGTCCTCCAGAGCAGGGATCGTCAGGTTCTTTTCAATCGTATAACCAAAA 6000

KC242793.1996 TTAGATTATTTGTCCTCCAGAGCAGGGATCGTCAGGTTCTTTTCAATCGTATAACCAAAA 6000

KC242794.1996 TTAGATTATTTGTCCTCCAGAGCAGGGATCGTCAGGTTCTTTTCAATCGTATAACCAAAA 6000

MH121164.1995 TTAGATTATTTGTCCTCCAGAGTAGGGATCGTCAGGTCCTTTTCAATCGTATAACCAAAA 6000

AY354458.1995 TTAGATTATTTGTCCTCCAGAGTAGGGATCGTCAGGTCCTTTTCAATCGTATAACCAAAA 6000

KT762962.1995 TTAGATTATTTGTCCTCCAGAGTAGGGATCGTCAGGTCCTTTTCAATCGTATAACCAAAA 6000

HQ613402.2008 TTAGATTATTTGTTTTCCAGAGTAGGGGTCATCAGGTCCTTTTCAATCGTATAACCAAAG 5919

KC242789.2007 TTAGATTATTTGTTTTCCAGAGTAGGGGTCATCAGGTCCTTTTCAATCGTATAACCAAAG 6000

HQ613403.2007 TTAGATTATTTGTTTTCCAGAGTAGGGGTCATCAGGTCCTTTTCAATCGTATAACCAAAG 5952

KC242785.2007 TTAGATTATTTGTTTTCCAGAGTAGGGGTCATCAGGTCCTTTTCAATCGTATAACCAAAG 6000

KC242790.2007 TTAGATTATTTGTTTTCCAGAGTAGGGGTCATCAGGTCCTTTTCAATCGTATAACCAAAG 6000

KU143789.2014 TTAGATTATTTGTCTTCCAGAGTAGGGGTCATCAGGTCCTTTTCAATTGGATAACCAAAA 6000

KR817168.2014 TTAGATTATTTGTCTTCCAGAGTAGGGGTCATCAGGTCCTTTTCAATTGGATAACCAAAA 5974

KY426696.2015 TTAGATTATTTGTCTTCCAGAGTAGGGGTCATCAGGTCCTTTTCAATTGGATAACCAAAA 6000

KR105271.2014 TTAGATTATTTGTCTTCCAGAGTAGGGGTCATCAGGTCCTTTTCAATTGGATAACCAAAA 5967

KY007522.2016 TTAGATTATTTGTCTTCCAGAGTAGGGGTCATCAGGTCCTTTTCAATTGGATAACCAAAA 5980

KM034555.2014 TTAGATTATTTGTCTTCCAGAGTAGGGGTCATCAGGTCCTTTTCAATTGGATAACCAAAA 5992

MH470381.2015 TTAGATTATTTGTCTTCCAGAGTAGGGGTCATCAGGTCCTTTTCAATTGGATAACCAAAA 5964

MH470382.2015 TTAGATTATTTGTCTTCCAGAGTAGGGGTCATCAGGTCCTTTTCAATTGGATAACCAAAA 5964

MF102255.2014 TTAGATTATTTGTCTTCCAGAGTAGGGGTCATCAGGTCCTTTTCAATTGGATAACCAAAA 5977

KJ660348.2014 TTAGATTATTTGTCTTCCAGAGTAGGGGTCATCAGGTCCTTTTCAATTGGATAACCAAAA 6000

KU143818.2014 TTAGATTATTTGTCTTCCAGAGTAGGGGTCATCAGGTCCTTTTCAATTGGATAACCAAAA 6000

KT725333.2014 TTAGATTATTTGTCTTCCAGAGTAGGGGTCATCAGGTCCTTTTCAATTGGATAACCAAAA 5965

KR819004.2014 TTAGATTATTTATCCTCCAGAGTAGGGATTGTCAGGTCCTTTTCAATCGTATAACCAAAA 5947

KP271020.2014 TTAGATTATTTATCCTCCAGAGTAGGGATTGTCAGGTCCTTTTCAATCGTATAACCAAAA 5947

KM519951.2014 TTAGATTATTTATCCTCCAGAGTAGGGATTGTCAGGTCCTTTTCAATCGTATAACCAAAA 5997

MH733488.2018 TTAGATTATTTGTCTTCCAGAGTAGGGATCGTCAGGTCCTTTTCAATCGTATAACCAAAA 5991

MH733491.2018 TTAGATTATTTGTCTTCCAGAGTAGGGATCGTCAGGTCCTTTTCAATCGTATAACCAAAA 5986

MH733478.2018 TTAGATTATTTGTCTTCCAGAGTAGGGATCGTCAGGTCCTTTTCAATCGTATAACCAAAA 5989

MK007330.2018 TTAGATTATTTGTTTTCCAGAGTAGGGGTCGTCAGGTCCTTTTCAATCGTATAACCAAGA 5988

MK007344.2018 TTAGATTATTTGTTTTCCAGAGTAGGGGTCGTCAGGTCCTTTTCAATCGTAGAACCAAGA 5982

*********** * ** **** **** * ****** **** **** *****

AY142960.1976 TAAACTCCACTAGAAGGATATTGTGGGGCAACAACACAATGGGCGTTACAGGAATATTGC 6060 start GP

KC242791.1977 TAAACTCCACTAGAAGGATATTGTGGGGCAACAACACAATGGGCGTTACAGGAATATTGC 6060

AF499101.1976 TAAACTCCACTAGAAGGATATTGTGGGGCAACAACACAATGGGCGTTACAGGAATATTGC 6060

KF113528.2003 TAAACTTCACTAGAAGGATATTGTGAGGCAACAACACAATGGGTATTACAGGAATATTGC 6056

KC242800.2002 TAAACTTCACTAGAAGGATATTGTGAGGCAACAACACAATGGGTATTACAGGAATATTGC 6060

KY471090.2001 TAAACTTCACTAGAAGGATATTGTGAGGCAACAACACAATGGGTATTACAGGAATATTGC 6037

KY471092.2001 TAAACTTCACTAGAAGGATATTGTGAGGCAACAACACAATGGGTATTACAGGAATATTGC 6037

MH481611.2017 TAAACTTCACTAGAAGGATATTGTGGGGCAACAACACAATGGGTGTTACAGAAATATTGC 6026

MH613311.2017 TAAACTTCACTAGAAGGATATTGTGGGGCAACAACACAATGGGTGTTACAGAAATATTGC 6005

KC242792.1994 TAAACTTCACTAGAAGGATATTGTGGGGCAACAACACAATGGGTGTTACAGGAATATTGC 6060

KC242793.1996 TAAACTTCACTAGAAGGATATTGTGGGGCAACAACACAATGGGTGTTACAGGAATATTGC 6060

KC242794.1996 TAAACTTCACTAGAAGGATATTGTGGGGCAACAACACAATGGGTGTTACAGGAATATTGC 6060

MH121164.1995 TAAACTTCACTAGAAGGATATTGTGGGGCAACAACACAATGGGTGTTACAGGAATATTGC 6060

AY354458.1995 TAAACTTCACTAGAAGGATATTGTGGGGCAACAACACAATGGGTGTTACAGGAATATTGC 6060

KT762962.1995 TAAACTTCACTAGAAGGATATTGTGGGGCAACAACACAATGGGTGTTACAGGAATATTGC 6060

HQ613402.2008 TAAACTTCACTAGAAGGATATTGTGGGGCAACAACACAATGGGTGTCACAGGAATATTGC 5979

KC242789.2007 TAAACTTCACTAGAAGGATATTGTGGGGCAACAACACAATGGGTGTCACAGGAATATTGC 6060

HQ613403.2007 TAAACTTCACTAGAAGGATATTGTGGGGCAACAACACAATGGGTGTCACAGGAATATTGC 6012

KC242785.2007 TAAACTTCACTAGAAGGATATTGTGGGGCAACAACACAATGGGTGTCACAGGAATATTGC 6060

KC242790.2007 TAAACTTCACTAGAAGGATATTGTGGGGCAACAACACAATGGGTGTCACAGGAATATTGC 6060

KU143789.2014 TAAGCTTCACTAGAAGGATATTGTGAGGCGACAACACAATGGGTGTTACAGGAATATTGC 6060

KR817168.2014 TAAGCTTCACTAGAAGGATATTGTGAGGCGACAACACAATGGGTGTTACAGGAATCTTGC 6034

KY426696.2015 TAAGCTTCACTAGAAGGATATTGTGAGGCGACAACACAATGGGTGTTACAGGAATATTGC 6060

KR105271.2014 TAAGCTTCACTAGAAGGATATTGTGAGGCGACAACACAATGGGTGTTACAGGAATATTGC 6027

KY007522.2016 TAAGCTTCACTAGAAGGATATTGTGAGGCGACAACACAATGGGTGTTACAGGAATATTGC 6040

KM034555.2014 TAAGCTTCACTAGAAGGATATTGTGAGGCGACAACACAATGGGTGTTACAGGAATATTGC 6052

MH470381.2015 TAAGCTTCACTAGAAGGATATTGTGAGGCGACAACACAATGGGTGTTACAGGAATATTGC 6024

MH470382.2015 TAAGCTTCACTAGAAGGATATTGTGAGGCGACAACACAATGGGTGTTACAGGAATATTGC 6024

MF102255.2014 TAAGCTTCACTAGAAGGATATTGTGAGGCGACAACACAATGGGTGTTACAGGAATATTGC 6037

KJ660348.2014 TAAGCTTCACTAGAAGGATATTGTGAGGCGACAACACAATGGGTGTTACAGGAATATTGC 6060

KU143818.2014 TAAGCTTCACTAGAAGGATATTGTGAGGCGACAACACAATGGGTGTTACAGGAATATTGC 6060

KT725333.2014 TAAGCTTCACTAGAAGGATATTGTGAGGCGACAACACAATGGGTGTTACAGGAATATTGC 6025

KR819004.2014 TAAACTTCACTAGAAGGATATTGTGGGGCAACAACACAATGGGTGTCACAGGAATATTGC 6007

KP271020.2014 TAAACTTCACTAGAAGGATATTGTGGGGCAACAACACAATGGGTGTCACAGGAATATTGC 6007

KM519951.2014 TAAACTTCACTAGAAGGATATTGTGGGGCAACAACACAATGGGTGTCACAGGAATATTGC 6057

MH733488.2018 TAAACTTCACTAGAAGGATATTGTGGGGCAACAACACAATGGGTGTCACAGGAATATTGC 6051

MH733491.2018 TAAACTTCACTAGAAGGATATTGTGGGGCAACAACACAATGGGTGTCACAGGAATATTGC 6046

MH733478.2018 TAAACTTCACTAGAAGGATATTGTGGGGCAACAACACAATGGGTGTCACAGGAATATTGC 6049

MK007330.2018 TAAACTTCACTAGAAGGATATTGTGGGGCAACAACACAATGGGTGCTACAGGAATATTAC 6048

MK007344.2018 TAAACTTCACTAGAAGGATATTGTGGGGCAACAACACAATGGGTGCTACAGGAATATTAC 6042

*** ** ****************** *** ************* **** *** ** *

AY142960.1976 AGTTACCTCGTGATCGATTCAAGAGGACATCATTCTTTCTTTGGGTAATTATCCTTTTCC 6120 <- R primer urGP

KC242791.1977 AGTTACCTCGTGATCGATTCAAGAGGACATCATTCTTTCTTTGGGTAATTATCCTTTTCC 6120

AF499101.1976 AGTTACCTCGTGATCGATTCAAGAGGACATCATTCTTTCTTTGGGTAATTATCCTTTTCC 6120

KF113528.2003 AGTTACCTCGTGATCGATTCAAGAGGACATCATTCTTTCTTTGGGTAATTATCCTTTTCC 6116

KC242800.2002 AGTTACCTCGTGATCGATTCAAGAGGACATCATTCTTTCTTTGGGTAATTATCCTTTTCC 6120

KY471090.2001 AGTTACCTCGTGATCGATTCAAGAGGACATCATTCTTTCTTTGGGTAATTATCCTTTTCC 6097

KY471092.2001 AGTTACCTCGTGATCGATTCAAGAGGACATCATTCTTTCTTTGGGTAATTATCCTTTTCC 6097

MH481611.2017 AGTTACCTCGTGATCGATTCAAGAGGACATCATTCTTTCTTTGGGTAATTATCCTTTTCC 6086

MH613311.2017 AGTTACCTCGTGATCGATTCAAGAGGACATCATTCTTTCTTTGGGTAATTATCCTTTTCC 6065

KC242792.1994 AGTTACCTCGTGATCGATTCAAGAGGACATCATTCTTTCTTTGGGTAATTATCCTTTTCC 6120

KC242793.1996 AGTTACCTCGTGATCGATTCAAGAGGACATCATTCTTTCTTTGGGTAATTATCCTTTTCC 6120

KC242794.1996 AGTTACCTCGTGATCGATTCAAGAGGACATCATTCTTTCTTTGGGTAATTATCCTTTTCC 6120

MH121164.1995 AGTTACCTCGTGATCGATTCAAGAGGACATCATTCTTTCTTTGGGTAATTATCCTTTTCC 6120

AY354458.1995 AGTTACCTCGTGATCGATTCAAGAGGACATCATTCTTTCTTTGGGTAATTATCCTTTTCC 6120

KT762962.1995 AGTTACCTCGTGATCGATTCAAGAGGACATCATTCTTTCTTTGGGTAATTATCCTTTTCC 6120

HQ613402.2008 AGTTACCTCGTGATCGATTCAAGAGGACATCATTCTTTCTTTGGGTAATTATCCTTTTCC 6039

KC242789.2007 AGTTACCTCGTGATCGATTCAAGAGGACATCATTCTTTCTTTGGGTAATTATCCTTTTCC 6120

HQ613403.2007 AGTTACCTCGTGATCGATTCAAGAGGACATCATTCTTTCTTTGGGTAATTATCCTTTTCC 6072

KC242785.2007 AGTTACCTCGTGATCGATTCAAGAGGACATCATTCTTTCTTTGGGTAATTATCCTTTTCC 6120

KC242790.2007 AGTTACCTCGTGATCGATTCAAGAGGACATCATTCTTTCTTTGGGTAATTATCCTTTTCC 6120

KU143789.2014 AGTTACCTCGTGATCGATTCAAGAGGACATCATTCTTTCTTTGGGTAATTATCCTTTTCC 6120

KR817168.2014 AGTTACCTCGTGATCGATTCAAGAGGACATCATTCTTTCTTTGGGTAATTATCCTTTTCC 6094

KY426696.2015 AGTTACCTCGTGATCGATTCAAGAGGACATCATTCTTTCTTTGGGTAATTATCCTTTTCC 6120

KR105271.2014 AGTTACCTCGTGATCGATTCAAGAGGACATCATTCTTTCTTTGGGTAATTATCCTTTTCC 6087

KY007522.2016 AGTTACCTCGTGATCGATTCAAGAGGACATCATTCTTTCTTTGGGTAATTATCCTCTTCC 6100

KM034555.2014 AGTTACCTCGTGATCGATTCAAGAGGACATCATTCTTTCTTTGGGTAATTATCCTTTTCC 6112

MH470381.2015 AGTTACCTCGTGATCGATTCAAGAGGACATCATTCTTTCTTTGGGTAATTATCCTTTTCC 6084

MH470382.2015 AGTTACCTCGTGATCGATTCAAGAGGACATCATTCTTTCTTTGGGTAATTATCCTTTTCC 6084

MF102255.2014 AGTTACCTCGTGATCGATTCAAGAGGACATCATTCTTTCTTTGGGTAATTATCCTTTTCC 6097

KJ660348.2014 AGTTACCTCGTGATCGATTCAAGAGGACATCATTCTTTCTTTGGGTAATTATCCTTTTCC 6120

KU143818.2014 AGTTACCTCGTGATCGATTCAAGAGGACATCATTCTTTCTTTGGGTAATTATCCTTTTCC 6120

KT725333.2014 AGTTACCTCGTGATCGATTCAAGAGGACATCATTCTTTCTTTGGGTAATTATCCTTTTCC 6085

KR819004.2014 AGTTACCTCGTGATCGATTCAAGAAGACATCATTCTTTCTTTGGGTAATTATCCTTTTCC 6067

KP271020.2014 AGTTACCTCGTGATCGATTCAAGAAGACATCATTCTTTCTTTGGGTAATTATCCTTTTCC 6067

KM519951.2014 AGTTACCTCGTGATCGATTCAAGAAGACATCATTCTTTCTTTGGGTAATTATCCTTTTCC 6117

MH733488.2018 AGTTACCTCGTGATCGATTCAAGAGGACATCATTCTTTCTTTGGGTAATTATCCTTTTCC 6111

MH733491.2018 AGTTACCTCGTGATCGATTCAAGAGGACATCATTCTTTCTTTGGGTAATTATCCTTTTCC 6106

MH733478.2018 AGTTACCTCGTGATCGATTCAAGAGGACATCATTCTTTCTTTGGGTAATTATCCTTTTCC 6109

MK007330.2018 AGTTACCTCGTGATCGATTCAAGAGGACATCATTCTTTCTTTGGGTAATTATCCTTTTCC 6108

MK007344.2018 AGTTACCTCGTGATCGATTCAAGAGGACATCATTCTTTCTTTGGGTAATTATCCTTTTCC 6102

************************ ****************************** ****

AY142960.1976 AAAGAACATTTTCCATCCCACTTGGAGTCATCCACAATAGCACATTACAGGTTAGTGATG 6180

KC242791.1977 AAAGAACATTTTCCATCCCACTTGGAGTCATCCACAATAGCACATTACAGGTTAGTGATG 6180

AF499101.1976 AAAGAACATTTTCCATCCCACTTGGAGTCATCCACAATAGCACATTACAGGTTAGTGATG 6180

KF113528.2003 AAAGAACATTTTCCATCCCACTTGGAGTCATCCACAATAGCACATTACAAGTTAGTGATG 6176

KC242800.2002 AAAGAACATTTTCCATCCCACTTGGAGTCATCCACAATAGCACATTACAAGTTAGTGATG 6180

KY471090.2001 AAAGAACATTTTCCATCCCACTTGGAGTCATCCACAATAGCACATTACAAGTTAGTGATG 6157

KY471092.2001 AAAGAACATTTTCCATCCCACTTGGAGTCATCCACAATAGCACATTACAAGTTAGTGATG 6157

MH481611.2017 AAAGAACATTTTCCATTCCACTTGGAGTCATCCACAATAGCACATTACAGGTTAGTGATG 6146

MH613311.2017 AAAGAACATTTTCCATTCCACTTGGAGTCATCCACAATAGCACATTACAGGTTAGTGATG 6125

KC242792.1994 AAAGAACATTTTCTATCCCACTTGGAGTCATCCACAATAGCACATTACAGGTTAGTGATG 6180

KC242793.1996 AAAGAACATTTTCCATCCCACTTGGAGTCATCCACAATAGCACATTACAGGTTAGTGATG 6180

KC242794.1996 AAAGAACATTTTCCATCCCACTTGGAGTCATCCACAATAGCACATTACAGGTTAGTGATG 6180

MH121164.1995 AAAGAACATTTTCCATCCCACTTGGAGTCATCCACAATAGCACATTACAGGTTAGTGATG 6180

AY354458.1995 AAAGAACATTTTCCATCCCACTTGGAGTCATCCACAATAGCACATTACAGGTTAGTGAGG 6180

KT762962.1995 AAAGAACATTTTCCATCCCACTTGGAGTCATCCACAATAGCACATTACAGGTTAGTGATG 6180

HQ613402.2008 AAAGAACATTTTCCATCCCACTTGGAGTCATCCACAATAGCACATTACAGGTTAGTGATG 6099

KC242789.2007 AAAGAACATTTTCCATCCCACTTGGAGTCATCCACAATAGCACATTACAGGTTAGTGATG 6180

HQ613403.2007 AAAGAACATTTTCCATCCCACTTGGAGTCATCCACAATAGCACATTACAGGTTAGTGATG 6132

KC242785.2007 AAAGAACATTTTCCATCCCACTTGGAGTCATCCACAATAGCACATTACAGGTTAGTGATG 6180

KC242790.2007 AAAGAACATTTTCCATCCCACTTGGAGTCATCCACAATAGCACATTACAGGTTAGTGATG 6180

KU143789.2014 AAAGAACATTTTCCATCCCGCTTGGAGTTATCCACAATAGTACATTACAGGTTAGTGATG 6180

KR817168.2014 AAAGAACATTTTCCATCCCGCTTGGAGTTATCCACAATAGTACATTACAGGTTAGTGATG 6154

KY426696.2015 AAAGAACATTTTCCATCCCGCTTGGAGTTATCCACAATAGTACATTACAGGTTAGTGATG 6180

KR105271.2014 AAAGAACATTTTCCATCCCGCTTGGAGTTATCCACAATAGTACATTACAGGTTAGTGATG 6147

KY007522.2016 AAAGAACATTTTCCATCCCGCTTGGAGTTATCCACAATAGTACATTACAGGTTAGTGATG 6160

KM034555.2014 AAAGAACATTTTCCATCCCGCTTGGAGTTATCCACAATAGTACATTACAGGTTAGTGATG 6172

MH470381.2015 AAAGAACATTTTCCATCCCGCTTGGAGTTATCCACAATAGTACATTACAGGTTAGTGATG 6144

MH470382.2015 AAAGAACATTTTCCATCCCGCTTGGAGTTATCCACAATAGTACATTACAGGTTAGTGATG 6144

MF102255.2014 AAAGAACATTTTCCATCCCGCTTGGAGTTATCCACAATAGTACATTACAGGTTAGTGATG 6157

KJ660348.2014 AAAGAACATTTTCCATCCCGCTTGGAGTTATCCACAATAGTACATTACAGGTTAGTGATG 6180

KU143818.2014 AAAGAACATTTTCCATCCCGCTTGGAGTTATCCACAATAGTACATTACAGGTTAGTGATG 6180

KT725333.2014 AAAGAACATTTTCCATCCCGCTTGGAGTTATCCACAATAGTACATTACAGGTTAGTGATG 6145

KR819004.2014 AAAGAACATTTTCCATCCCACTTGGAGTCATCCACAATAGCACATTACAGGTTAGTGATG 6127

KP271020.2014 AAAGAACATTTTCCATCCCACTTGGAGTCATCCACAATAGCACATTACAGGTTAGTGATG 6127

KM519951.2014 AAAGAACATTTTCCATCCCACTTGGAGTCATCCACAATAGCACATTACAGGTTAGTGATG 6177

MH733488.2018 AAAGAACATTTTCCATCCCACTTGGAGTCATCCACAATAGCACATTACAGGTTAGTGATG 6171

MH733491.2018 AAAGAACATTTTCCATCCCACTTGGAGTCATCCACAATAGCACATTACAGGTTAGTGATG 6166

MH733478.2018 AAAGAACATTTTCCATCCCACTTGGAGTCATCCACAATAGCACATTACAGGTTAGTGATG 6169

MK007330.2018 AAAGAACATTTTCCATCCCACTTGGAGTCATCCACAATAGCACATTACAGGTTAGTGATG 6168

MK007344.2018 AAAGAACATTTTCCATCCCACTTGGAGTCATCCACAATAGCACATTACAGGTTAGTGATG 6162

************* ** ** ******** *********** ******** ******** *

AY142960.1976 TCGACAAACTAGTTTGTCGTGACAAACTGTCATCCACAAATCAATTGAGATCAGTTGGAC 6240

KC242791.1977 TCGACAAACTAGTTTGTCGTGACAAACTGTCATCCACAAATCAATTGAGATCAGTTGGAC 6240

AF499101.1976 TCGACAAACTAGTTTGTCGTGACAAACTGTCATCCACAAATCAATTGAGACCAGTTGGAC 6240

KF113528.2003 TCGACAAACTAGTTTGTCGTGACAAACTGTCATCCACAAATCAATTGAGATCAGTTGGAC 6236

KC242800.2002 TCGACAAACTAGTTTGTCGTGACAAACTGTCATCCACAAATCAATTGAGATCAGTTGGAC 6240

KY471090.2001 TCGACAAACTAGTTTGTCGTGACAAACTGTCATCCACAAATCAATTGAGATCAGTTGGAC 6217

KY471092.2001 TCGACAAACTAGTTTGTCGTGACAAACTGTCATCCACAAATCAATTGAGATCAGTTGGAC 6217

MH481611.2017 TCGACAAACTAGTTTGTCGTGACAAACTGTCATCCACAAATCAATTGAGATCAGTTGGAC 6206

MH613311.2017 TCGACAAACTAGTTTGTCGTGACAAACTGTCATCCACAAATCAATTGAGATCAGTTGGAC 6185

KC242792.1994 TCGACAAACTGGTTTGCCGTGACAAACTGTCATCCACGAATCAATTGAGATCAGTTGGAC 6240

KC242793.1996 TCGACAAACTGGTTTGCCGTGACAAACTGTCATCCACGAATCAATTGAGATCAGTTGGAC 6240

KC242794.1996 TCGACAAACTGGTTTGCCGTGACAAACTGTCATCCACGAATCAATTGAGATCAGTTGGAC 6240

MH121164.1995 TCGACAAACTGGTTTGCCGTGACAAACTGTCATCCACAAATCAATTGAGATCAGTTGGAC 6240

AY354458.1995 TCGACAAACTGGTTTGCCGTGACAAACTGTCATCCACAAATCAATTGAGATCAGTTGGAC 6240

KT762962.1995 TCGACAAACTGGTTTGCCGTGACAAACTGTCATCCACAAATCAATTGAGATCAGTTGGAC 6240

HQ613402.2008 TCGACAAACTAGTTTGTCGTGACAAACTGTCATCCACAAATCAATTGAGATCAGTTGGAC 6159

KC242789.2007 TCGACAAACTAGTTTGTCGTGACAAACTGTCATCCACAAATCAATTGAGATCAGTTGGAC 6240

HQ613403.2007 TCGACAAACTAGTTTGTCGTGACAAACTGTCATCCACAAATCAATTGAGATCAGTTGGAC 6192

KC242785.2007 TCGACAAACTAGTTTGTCGTGACAAACTGTCATCCACAAATCAATTGAGATCAGTTGGAC 6240

KC242790.2007 TCGACAAACTAGTTTGTCGTGACAAACTGTCATCCACAAATCAATTGAGATCAGTTGGAC 6240

KU143789.2014 TCGACAAACTAGTTTGTCGTGACAAACTGTCATCCACAAATCAATTGAGATCAGTTGGAC 6240

KR817168.2014 TCGACAAACTAGTTTGTCGTGACAAACTGTCATCCACAAATCAATTGAGATCAGTTGGAC 6214

KY426696.2015 TCGACAAACTAGTTTGTCGTGACAAACTGTCATCCACAAATCAATTGAGATCAGTTGGAC 6240

KR105271.2014 TCGACAAACTAGTTTGTCGTGACAAACTGTCATCCACAAATCAATTGAGATCAGTTGGAC 6207

KY007522.2016 TCGACAAACTAGTTTGTCGTGACAAACTGTCATCCACAAATCAATTGAGATCAGTTGGAC 6220

KM034555.2014 TCGACAAACTAGTTTGTCGTGACAAACTGTCATCCACAAATCAATTGAGATCAGTTGGAC 6232

MH470381.2015 TCGACAAACTAGTTTGTCGTGACAAACTGTCATCCACAAATCAATTGAGATCAGTTGGAC 6204

MH470382.2015 TCGACAAACTAGTTTGTCGTGACAAACTGTCATCCACAAATCAATTGAGATCAGTTGGAC 6204

MF102255.2014 TCGACAAACTAGTTTGTCGTGACAAACTGTCATCCACAAATCAATTGAGATCAGTTGGAC 6217

KJ660348.2014 TCGACAAACTAGTTTGTCGTGACAAACTGTCATCCACAAATCAATTGAGATCAGTTGGAC 6240

KU143818.2014 TCGACAAACTAGTTTGTCGTGACAAACTGTCATCCACAAATCAATTGAGATCAGTTGGAC 6240

KT725333.2014 TCGACAAACTAGTTTGTCGTGACAAACTGTCATCCACAAATCAATTGAGATCAGTTGGAC 6205

KR819004.2014 TCGACAAACTGGTTTGCCGTGACAAACTGTCATCCACAAATCAATTGAGATCAGTTGGAC 6187

KP271020.2014 TCGACAAACTGGTTTGCCGTGACAAACTGTCATCCACAAATCAATTGAGATCAGTTGGAC 6187

KM519951.2014 TCGACAAACTGGTTTGCCGTGACAAACTGTCATCCACAAATCAATTGAGATCAGTTGGAC 6237

MH733488.2018 TCGACAAACTGGTTTGCCGTGACAAACTGTCATCCACAAATCAATTGAGATCAGTTGGAC 6231

MH733491.2018 TCGACAAACTGGTTTGCCGTGACAAACTGTCATCCACAAATCAATTGAGATCAGTTGGAC 6226

MH733478.2018 TCGACAAACTGGTTTGCCGTGACAAACTGTCATCCACAAATCAATTGAGATCAGTTGGAC 6229

MK007330.2018 TCGACAAACTAGTTTGTCGTGACAAACTGTCATCCACAAATCAATTGAGATCAGTTGGAC 6228

MK007344.2018 TCGACAAACTAGTTTGTCGTGACAAACTGTCATCCACAAATCAATTGAGATCAGTTGGAC 6222

********** ***** ******************** ************ *********

AY142960.1976 TGAATCTCGAAGGGAATGGAGTGGCAACTGACGTGCCATCTGCAACTAAAAGATGGGGCT 6300

KC242791.1977 TGAATCTCGAAGGGAATGGAGTGGCAACTGACGTGCCATCTGCAACTAAAAGATGGGGCT 6300

AF499101.1976 TGAATCTCGAAGGGAATGGAGTGGCAACTGACGTGCCATCTGCAACTAAAAGATGGGGCT 6300

KF113528.2003 TGAATCTCGAAGGGAATGGAGTGGCAACTGACGTGCCATCTGCAACTAAAAGATGGGGCT 6296

KC242800.2002 TGAATCTCGAAGGGAATGGAGTGGCAACTGACGTGCCATCTGCAACTAAAAGATGGGGCT 6300

KY471090.2001 TGAATCTCGAAGGGAATGGAGTGGCAACTGACGTGCCATCTGCAACTAAAAGATGGGGCT 6277

KY471092.2001 TGAATCTCGAAGGGAATGGAGTGGCAACTGACGTGCCATCTGCAACTAAAAGATGGGGCT 6277

MH481611.2017 TGAATCTCGAAGGGAATGGAGTGGCAACTGACGTGCCATCTGCAACTAAAAGATGGGGCT 6266

MH613311.2017 TGAATCTCGAAGGGAATGGAGTGGCAACTGACGTGCCATCTGCAACTAAAAGATGGGGCT 6245

KC242792.1994 TGAATCTCGAAGGGAATGGAGTGGCAACTGACGTGCCATCTGCAACTAAAAGATGGGGCT 6300

KC242793.1996 TGAATCTCGAAGGGAATGGAGTGGCAACTGACGTGCCATCTGCAACTAAAAGATGGGGCT 6300

KC242794.1996 TGAATCTCGAAGGGAATGGAGTGGCAACTGACGTGCCATCTGCAACTAAAAGATGGGGCT 6300

MH121164.1995 TGAATCTCGAAGGGAATGGAGTGGCAACTGACGTGCCATCTGCAACTAAAAGATGGGGCT 6300

AY354458.1995 TGAATCTCGAAGGGAATGGAGTGGCAACTGACGTGCCATCTGCAACTAAAAGATGGGGCT 6300

KT762962.1995 TGAATCTCGAAGGGAATGGAGTGGCAACTGACGTGCCATCTGCAACTAAAAGATGGGGCT 6300

HQ613402.2008 TGAATCTCGAAGGGAATGGAGTGGCAACTGATGTGCCATCTGCAACTAAAAGATGGGGCT 6219

KC242789.2007 TGAATCTCGAAGGGAATGGAGTGGCAACTGATGTGCCATCTGCAACTAAAAGATGGGGCT 6300

HQ613403.2007 TGAATCTCGAAGGGAATGGAGTGGCAACTGATGTGCCATCTGCAACTAAAAGATGGGGCT 6252

KC242785.2007 TGAATCTCGAAGGGAATGGAGTGGCAACTGATGTGCCATCTGCAACTAAAAGATGGGGCT 6300

KC242790.2007 TGAATCTCGAAGGGAATGGAGTGGCAACTGATGTGCCATCTGCAACTAAAAGATGGGGCT 6300

KU143789.2014 TGAATCTCGAGGGGAATGGAGTGGCAACTGACGTGCCATCTGTGACTAAAAGATGGGGCT 6300

KR817168.2014 TGAATCTCGAGGGGAATGGAGTGGCAACTGACGTGCCATCTGTGACTAAAAGATGGGGCT 6274

KY426696.2015 TGAATCTCGAGGGGAATGGAGTGGCAACTGACGTGCCATCTGTGACTAAAAGATGGGGCT 6300

KR105271.2014 TGAATCTCGAGGGGAATGGAGTGGCAACTGACGTGCCATCTGTGACTAAAAGATGGGGCT 6267

KY007522.2016 TGAATCTCGAGGGGAATGGAGTGGCAACTGACGTGCCATCTGTGACTAAAAGATGGGGCT 6280

KM034555.2014 TGAATCTCGAGGGGAATGGAGTGGCAACTGACGTGCCATCTGTGACTAAAAGATGGGGCT 6292

MH470381.2015 TGAATCTCGAGGGGAATGGAGTGGCAACTGACGTGCCATCTGTGACTAAAAGATGGGGCT 6264

MH470382.2015 TGAATCTCGAGGGGAATGGAGTGGCAACTGACGTGCCATCTGTGACTAAAAGATGGGGCT 6264

MF102255.2014 TGAATCTCGAGGGGAATGGAGTGGCAACTGACGTGCCATCTGCGACTAAAAGATGGGGCT 6277

KJ660348.2014 TGAATCTCGAGGGGAATGGAGTGGCAACTGACGTGCCATCTGCGACTAAAAGATGGGGCT 6300

KU143818.2014 TGAATCTCGAGGGGAATGGAGTGGCAACTGACGTGCCATCTGTGACTAAAAGATGGGGCT 6300

KT725333.2014 TGAATCTCGAGGGGAATGGAGTGGCAACTGACGTGCCATCTGTGACTAAAAGATGGGGCT 6265

KR819004.2014 TGAATCTCGAAGGGAATGGAGTGGCAACCGACGTGCCATCTGCAACTAAAAGATGGGGCT 6247

KP271020.2014 TGAATCTCGAAGGGAATGGAGTGGCAACCGACGTGCCATCTGCAACTAAAAGATGGGGCT 6247

KM519951.2014 TGAATCTCGAAGGGAATGGAGTGGCAACCGACGTGCCATCTGCAACTAAAAGATGGGGCT 6297

MH733488.2018 TGAATCTCGAAGGGAATGGAGTGGCAACCGACGTGCCATCTGCAACTAAAAGATGGGGCT 6291

MH733491.2018 TGAATCTCGAAGGGAATGGAGTGGCAACCGACGTGCCATCTGCAACTAAAAGATGGGGCT 6286

MH733478.2018 TGAATCTCGAAGGGAATGGAGTGGCAACCGACGTGCCATCTGCAACTAAAAGATGGGGCT 6289

MK007330.2018 TGAATCTCGAAGGGAATGGAGTGGCAACTGACGTGCCATCTGCAACTAAAAGATGGGGCT 6288

MK007344.2018 TGAATCTCGAAGGGAATGGAGTGGCAACTGACGTGCCATCTGCAACTAAAAGATGGGGCT 6282

********** ***************** ** ********** ****************

AY142960.1976 TCAGGTCCGGTGTCCCACCAAAGGTGGTCAATTATGAAGCTGGTGAA*TGGGCTGAAAACT* 6360 *EBOV-GP2-Fwd Lau (22)*

KC242791.1977 TCAGGTCCGGTGTCCCACCAAAGGTGGTCAATTATGAAGCTGGTGAA*TGGGCTGAAAACT* 6360

AF499101.1976 TCAGGTCCGGTGTCCCACCAAAGGTGGTCAATTATGAAGCTGGTGAA*TGGGCTGAAAACT* 6360

KF113528.2003 TCAGGTCCGGTGTCCCTCCAAAGGTGGTCAATTATGAAGCTGGTGAA*TGGGCTGAAAACT* 6356

KC242800.2002 TCAGGTCCGGTGTCCCTCCAAAGGTGGTCAATTATGAAGCTGGTGAA*TGGGCTGAAAACT* 6360

KY471090.2001 TCAGGTCCGGTGTCCCTCCAAAGGTGGTCAATTATGAAGCTGGTGAA*TGGGCTGAAAACT* 6337

KY471092.2001 TCAGGTCCGGTGTCCCTCCAAAGGTGGTCAATTATGAAGCTGGTGAA*TGGGCTGAAAACT* 6337

MH481611.2017 TCAGGTCCGGTGTCCCACCAAAGGTGGTCAATTATGAAGCTGGTGAA*TGGGCTGAAAACT* 6326

MH613311.2017 TCAGGTCCGGTGTCCCACCAAAGGTGGTCAATTATGAAGCTGGTGAA*TGGGCTGAAAACT* 6305

KC242792.1994 TCAGGTCCGGTGTCCCACCAAAAGTGGTCAATTATGAAGCTGGTGAA*TGGGCTGAAAACT* 6360

KC242793.1996 TCAGGTCCGGTGTCCCACCAAAAGTGGTCAATTATGAAGCTGGTGAA*TGGGCTGAAAACT* 6360

KC242794.1996 TCAGGTCCGGTGTCCCACCAAAAGTGGTCAATTATGAAGCTGGTGAA*TGGGCTGAAAACT* 6360

MH121164.1995 TCAGGTCCGGTGTCCCACCAAAGGTGGTCAATTATGAAGCTGGTGAA*TGGGCTGAAAACT* 6360

AY354458.1995 TCAGGTCCGGTGTCCCACCAAAGGTGGTCAATTATGAAGCTGGTGAA*TGGGCTGAAAACT* 6360

KT762962.1995 TCAGGTCCGGTGTCCCACCAAAGGTGGTCAATTATGAAGCTGGTGAA*TGGGCTGAAAACT* 6360

HQ613402.2008 TCAGGTCCGGTGTCCCACCAAAGGTGGTCAATTATGAAGCTGGTGAA*TGGGCTGAAAACT* 6279

KC242789.2007 TCAGGTCCGGTGTCCCACCAAAGGTGGTCAATTATGAAGCTGGTGAA*TGGGCTGAAAACT* 6360

HQ613403.2007 TCAGGTCCGGTGTCCCACCAAAGGTGGTCAATTATGAAGCTGGTGAA*TGGGCTGAAAACT* 6312

KC242785.2007 TCAGGTCCGGTGTCCCACCAAAGGTGGTCAATTATGAAGCTGGTGAA*TGGGCTGAAAACT* 6360

KC242790.2007 TCAGGTCCGGTGTCCCACCAAAGGTGGTCAATTATGAAGCTGGTGAA*TGGGCTGAAAACT* 6360

KU143789.2014 TCAGGTCCGGTGTCCCACCAAAGGTGGTCAATTATGAAGCTGGTGAA*TGGGCTGAAAACT* 6360

KR817168.2014 TCAGGTCCGGTGTCCCACCAAAGGTGGTCAATTATGAAGCTGGTGAA*TGGGCTGAAAACT* 6334

KY426696.2015 TCAGGTCCGGTGTCCCACCAAAGGTGGTCAATTATGAAGCTGGTGAA*TGGGCTGAAAACT* 6360

KR105271.2014 TCAGGTCCGGTGTCCCACCAAAGGTGGTCAATTATGAAGCTGGTGAA*TGGGCTGAAAACT* 6327

KY007522.2016 TCAGGTCCGGTGTCCCACCAAAGGTGGTCAATTATGAAGCTGGTGAA*TGGGCTGAAAACT* 6340

KM034555.2014 TCAGGTCCGGTGTCCCACCAAAGGTGGTCAATTATGAAGCTGGTGAA*TGGGCTGAAAACT* 6352

MH470381.2015 TCAGGTCCGGTGTCCCACCAAAGGTGGTCAATTATGAAGCTGGTGAA*TGGGCTGAAAACT* 6324

MH470382.2015 TCAGGTCCGGTGTCCCACCAAAGGTGGTCAATTATGAAGCTGGTGAA*TGGGCTGAAAACT* 6324

MF102255.2014 TCAGGTCCGGTGTCCCACCAAAGGTGGTCAATTATGAAGCTGGTGAA*TGGGCTGAAAACT* 6337

KJ660348.2014 TCAGGTCCGGTGTCCCACCAAAGGTGGTCAATTATGAAGCTGGTGAA*TGGGCTGAAAACT* 6360

KU143818.2014 TCAGGTCCGGTGTCCCACCAAAGGTGGTCAATTATGAAGCTGGTGAA*TGGGCTGAAAACT* 6360

KT725333.2014 TCAGGTCCGGTGTCCCACCAAAGGTGGTCAATTATGAAGCTGGTGAA*TGGGCTGAAAACT* 6325

KR819004.2014 TCAGGTCCGGTGTCCCACCAAAGGTGGTCAATTATGAAGCTGGTGAA*TGGGCTGAAAACT* 6307

KP271020.2014 TCAGGTCCGGTGTCCCACCAAAGGTGGTCAATTATGAAGCTGGTGAA*TGGGCTGAAAACT* 6307

KM519951.2014 TCAGGTCCGGTGTCCCACCAAAGGTGGTCAATTATGAAGCTGGTGAA*TGGGCTGAAAACT* 6357

MH733488.2018 TCAGGTCCGGTGTCCCACCAAAAGTGGTCAATTATGAAGCTGGTGAA*TGGGCTGAAAACT* 6351

MH733491.2018 TCAGGTCCGGTGTCCCACCAAAAGTGGTCAATTATGAAGCTGGTGAA*TGGGCTGAAAACT* 6346

MH733478.2018 TCAGGTCCGGTGTCCCACCAAAAGTGGTCAATTATGAAGCTGGTGAA*TGGGCTGAAAACT* 6349

MK007330.2018 TCAGGTCCGGTGTCCCACCAAAGGTGGTCAATTATGAAGCTGGTGAA*TGGGCTGAAAACT* 6348

MK007344.2018 TCAGGTCCGGTGTCCCACCAAAGGTGGTCAATTATGAAGCTGGTGAA*TGGGCTGAAAACT* 6342

**************** ***** *************************************

AY142960.1976 *GCTACAAT*CTTGAAATCAAAAAACCTGACGGGAGTGAGTGTCTACCAGCAGCGCCAGACG 6420

KC242791.1977 *GCTACAAT*CTTGAAATCAAAAAACCTGACGGGAGTGAGTGTCTACCAGCAGCGCCAGACG 6420

AF499101.1976 *GCTACAAT*CTTGAAATCAAAAAACCTGACGGGAGTGAGTGTCTACCAGCAGCGCCAGACG 6420

KF113528.2003 *GCTACAAT*CTTGAAATCAAAAAACCTGACGGGAGTGAGTGTCTACCAGCAGCGCCAGACG 6416

KC242800.2002 *GCTACAAT*CTTGAAATCAAAAAACCTGACGGGAGTGAGTGTCTACCAGCAGCGCCAGACG 6420

KY471090.2001 *GCTACAAT*CTTGAAATCAAAAAACCTGACGGGAGTGAGTGTCTACCAGCAGCGCCAGACG 6397

KY471092.2001 *GCTACAAT*CTTGAAATCAAAAAACCTGACGGGAGTGAGTGTCTACCAGCAGCGCCAGACG 6397

MH481611.2017 *GCTACAAT*CTTGAGATCAAAAAACCTGACGGGAGTGAGTGTCTACCAGCAGCGCCAGACG 6386

MH613311.2017 *GCTACAAT*CTTGAGATCAAAAAACCTGACGGGAGTGAGTGTCTACCAGCAGCGCCAGACG 6365

KC242792.1994 *GCTACAAT*CTTGAAATCAAAAAACCTGACGGGAGTGAGTGTCTACCAGCAGCGCCAGACG 6420

KC242793.1996 *GCTACAAT*CTTGAAATCAAAAAACCTGACGGGAGTGAGTGTCTACCAGCAGCGCCAGACG 6420

KC242794.1996 *GCTACAAT*CTTGAAATCAAAAAACCTGACGGGAGTGAGTGTCTACCAGCAGCGCCAGACG 6420

MH121164.1995 *GCTACAAT*CTTGAAATCAAAAAACCTGACGGGAGTGAGTGTCTACCAGCAGCGCCAGACG 6420

AY354458.1995 *GCTACAAT*CTTGAAATCAAAAAACCTGACGGGAGTGAGTGTCTACCAGCAGCGCCAGACG 6420

KT762962.1995 *GCTACAAT*CTTGAAATCAAAAAACCTGACGGGAGTGAGTGTCTACCAGCAGCGCCAGACG 6420

HQ613402.2008 *GCTACAAT*CTTGAAATCAAAAAACCTGACGGGAGTGAGTGTCTACCAGCAGCGCCAGACG 6339

KC242789.2007 *GCTACAAT*CTTGAAATCAAAAAACCTGACGGGAGTGAGTGTCTACCAGCAGCGCCAGACG 6420

HQ613403.2007 *GCTACAAT*CTTGAAATCAAAAAACCTGACGGGAGTGAGTGTCTACCAGCAGCGCCAGACG 6372

KC242785.2007 *GCTACAAT*CTTGAAATCAAAAAACCTGACGGGAGTGAGTGTCTACCAGCAGCGCCAGACG 6420

KC242790.2007 *GCTACAAT*CTTGAAATCAAAAAACCTGACGGGAGTGAGTGTCTACCAGCAGCGCCAGACG 6420

KU143789.2014 *GCTACAAT*CTTGAAATCAAAAAACCTGACGGGAGTGAGTGTCTACCAGCAGCGCCAGACG 6420

KR817168.2014 *GCTACAAT*CTTGAAATCAAAAAACCTGACGGGAGTGAGTGTCTACCAGCAGCGCCAGACG 6394

KY426696.2015 *GCTACAAT*CTTGAAATCAAAAAACCTGACGGGAGTGAGTGTCTACCAGCAGCGCCAGACG 6420

KR105271.2014 *GCTACAAT*CTTGAAATCAAAAAACCTGACGGGAGTGAGTGTCTACCAGCAGCGCCAGACG 6387

KY007522.2016 *GCTACAAT*CTTGAAATCAAAAAACCTGACGGGAGTGAGTGTCTACCAGCAGCGCCAGACG 6400

KM034555.2014 *GCTACAAT*CTTGAAATCAAAAAACCTGACGGGAGTGAGTGTCTACCAGCAGCGCCAGACG 6412

MH470381.2015 *GCTACAAT*CTTGAAATCAAAAAACCTGACGGGAGTGAGTGTCTACCAGCAGCGCCAGACG 6384

MH470382.2015 *GCTACAAT*CTTGAAATCAAAAAACCTGACGGGAGTGAGTGTCTACCAGCAGCGCCAGACG 6384

MF102255.2014 *GCTACAAT*CTTGAAATCAAAAAACCTGACGGGAGTGAGTGTCTACCAGCAGCGCCAGACG 6397

KJ660348.2014 *GCTACAAT*CTTGAAATCAAAAAACCTGACGGGAGTGAGTGTCTACCAGCAGCGCCAGACG 6420

KU143818.2014 *GCTACAAT*CTTGAAATCAAAAAACCTGACGGGAGTGAGTGTCTACCAGCAGCGCCAGACG 6420

KT725333.2014 *GCTACAAT*CTTGAAATCAAAAAACCTGACGGGAGTGAGTGTCTACCAGCAGCGCCAGACG 6385

KR819004.2014 *GCTACAAT*CTTGAAATCAAAAAACCTGACGGGAGTGAGTGTCTACCAGCAGCGCCAGACG 6367

KP271020.2014 *GCTACAAT*CTTGAAATCAAAAAACCTGACGGGAGTGAGTGTCTACCAGCAGCGCCAGACG 6367

KM519951.2014 *GCTACAAT*CTTGAAATCAAAAAACCTGACGGGAGTGAGTGTCTACCAGCAGCGCCAGACG 6417

MH733488.2018 *GCTACAAT*CTTGAAATCAAAAAACCTGACGGGAGTGAGTGTCTACCAGCAGCGCCAGACG 6411

MH733491.2018 *GCTACAAT*CTTGAAATCAAAAAACCTGACGGGAGTGAGTGTCTACCAGCAGCGCCAGACG 6406

MH733478.2018 *GCTACAAT*CTTGAAATCAAAAAACCTGACGGGAGTGAGTGTCTACCAGCAGCGCCAGACG 6409

MK007330.2018 *GCTACAAT*CTTGAAATCAAAAAACCTGACGGGAGTGAGTGTCTACCAGCAGCGCCAGACG 6408

MK007344.2018 *GCTACAAT*CTTGAAATCAAAAAACCTGACGGGAGTGAGTGTCTACCAGCAGCGCCAGACG 6402

************* **********************************************

AY142960.1976 GGATTCGGGGCTTCCCCCG*GTGCCGGTATGTGCACAAAG*TATCAGGAACGGGACCGTGTG 6480 *EBOV-Rev-Fwd Lau (22)*

KC242791.1977 GGATTCGGGGCTTCCCCCG*GTGCCGGTATGTGCACAAAG*TATCAGGAACGGGACCGTGTG 6480

AF499101.1976 GGATTCGGGGCTTCCCCCG*GTGCCGGTATGTGCACAAAG*TATCAGGAACGGGACCGTGTG 6480

KF113528.2003 GGATTCGGGGCTTCCCCCG*GTGCCGGTATGTGCACAAAG*TATCAGGAACGGGACCGTGTG 6476

KC242800.2002 GGATTCGGGGCTTCCCCCG*GTGCCGGTATGTGCACAAAG*TATCAGGAACGGGACCGTGTG 6480

KY471090.2001 GGATTCGGGGCTTCCCCCG*GTGCCGGTATGTGCACAAAG*TATCAGGAACGGGACCGTGTG 6457

KY471092.2001 GGATTCAGGGCTTCCCCCG*GTGCCGGTATGTGCACAAAG*TATCAGGAACGGGACCGTGTG 6457

MH481611.2017 GGATTCGGGGCTTCCCCCG*GTGCCGGTATGTGCACAAAG*TATCAGGAACGGGACCGTGTG 6446

MH613311.2017 GGATTCGGGGCTTCCCCCG*GTGCCGGTATGTGCACAAAG*TATCAGGAACGGGACCGTGTG 6425

KC242792.1994 GGATTCGGGGCTTCCCCCG*GTGCCGGTATGTGCACAAAG*TATCAGGAACGGGACCGTGTG 6480

KC242793.1996 GGATTCGGGGCTTCCCCCG*GTGCCGGTATGTGCACAAAG*TATCAGGAACGGGACCGTGTG 6480

KC242794.1996 GGATTCGGGGCTTCCCCCG*GTGCCGGTATGTGCACAAAG*TATCAGGAACGGGACCGTGTG 6480

MH121164.1995 GGATTCGGGGCTTCCCCCG*GTGCCGGTATGTGCACAAAG*TATCAGGAACGGGACCGTGTG 6480

AY354458.1995 GGATTCGGGGCTTCCCCCG*GTGCCGGTATGTGCACAAAG*TATCAGGAACGGGACCGTGTG 6480

KT762962.1995 GGATTCGGGGCTTCCCCCG*GTGCCGGTATGTGCACAAAG*TATCAGGAACGGGACCGTGTG 6480

HQ613402.2008 GGATTCGGGGCTTCCCCCG*GTGCCGGTATGTGCACAAAG*TATCAGGAACAGGACCGTGTG 6399

KC242789.2007 GGATTCGGGGCTTCCCCCG*GTGCCGGTATGTGCACAAAG*TATCAGGAACAGGACCGTGTG 6480

HQ613403.2007 GGATTCGGGGCTTCCCCCG*GTGCCGGTATGTGCACAAAG*TATCAGGAACAGGACCGTGTG 6432

KC242785.2007 GGATTCGGGGCTTCCCCCG*GTGCCGGTATGTGCACAAAG*TATCAGGAACAGGACCGTGTG 6480

KC242790.2007 GGATTCGGGGCTTCCCCCG*GTGCCGGTATGTGCACAAAG*TATCAGGAACAGGACCGTGTG 6480

KU143789.2014 GGATTCGGGGCTTCCCCCG*GTGCCGGTATGTGCACAAAG*TATCAGGAACGGGACCATGTG 6480

KR817168.2014 GGATTCGGGGCTTCCCCCG*GTGCCGGTATGTGCACAAAG*TATCAGGAACGGGACCATGTG 6454

KY426696.2015 GGATTCGGGGCTTCCCCCG*GTGCCGGTATGTGCACAAAG*TATCAGGAACGGGACCATGTG 6480

KR105271.2014 GGATTCGGGGCTTCCCCCG*GTGCCGGTATGTGCACAAAG*TATCAGGAACGGGACCATGTG 6447

KY007522.2016 GGATTCGGGGCTTCCCCCG*GTGCCGGTATGTGCACAAAG*TATCAGGAACGGGACCATGTG 6460

KM034555.2014 GGATTCGGGGCTTCCCCCG*GTGCCGGTATGTGCACAAAG*TATCAGGAACGGGACCATGTG 6472

MH470381.2015 GGATTCGGGGCTTCCCCCG*GTGCCGGTATGTGCACAAAG*TATCAGGAACGGGACCATGTG 6444

MH470382.2015 GGATTCGGGGCTTCCCCCG*GTGCCGGTATGTGCACAAAG*TATCAGGAACGGGACCATGTG 6444

MF102255.2014 GGATTCGGGGCTTCCCCCG*GTGCCGGTATGTGCACAAAG*TATCAGGAACGGGACCATGTG 6457

KJ660348.2014 GGATTCGGGGCTTCCCCCG*GTGCCGGTATGTGCACAAAG*TATCAGGAACGGGACCATGTG 6480

KU143818.2014 GGATTCGGGGCTTCCCCCG*GTGCCGGTATGTGCACAAAG*TATCAGGAACGGGACCATGTG 6480

KT725333.2014 GGATTCGGGGCTTCCCCCG*GTGCCGGTATGTGCACAAAG*TATCAGGAACGGGACCATGTG 6445

KR819004.2014 GGATTCGGGGCTTCCCCCG*GTGCCGGTATGTGCACAAAG*TATCAGGAACGGGACCGTGTG 6427

KP271020.2014 GGATTCGGGGCTTCCCCCG*GTGCCGGTATGTGCACAAAG*TATCAGGAACGGGACCGTGTG 6427

KM519951.2014 GGATTCGGGGCTTCCCCCG*GTGCCGGTATGTGCACAAAG*TATCAGGAACGGGACCGTGTG 6477

MH733488.2018 GGATTCGGGGCTTCCCCCG*GTGCCGGTATGTGCACAAAG*TATCAGGAACGGGACCGTGTG 6471

MH733491.2018 GGATTCGGGGCTTCCCCCG*GTGCCGGTATGTGCACAAAG*TATCAGGAACGGGACCGTGTG 6466

MH733478.2018 GGATTCGGGGCTTCCCCCG*GTGCCGGTATGTGCACAAAG*TATCAGGAACGGGACCGTGTG 6469

MK007330.2018 GGATTCGGGGCTTCCCCCG*GTGCCGGTATGTGCACAAAG*TATCAGGAACGGGACCGTGTG 6468

MK007344.2018 GGATTCGGGGCTTCCCCCG*GTGCCGGTATGTGCACAAAG*TATCAGGAACGGGACCGTGTG 6462

****** ****************************************** ***** ****

AY142960.1976 CCGGAGACTTTGCCTTCCATAAAGAGGGTGCTTTCTTCCTGTATGATCGACTTGCTTCCA 6540

KC242791.1977 CCGGAGACTTTGCCTTCCATAAAGAGGGTGCTTTCTTCCTGTATGATCGACTTGCTTCCA 6540

AF499101.1976 CCGGAGACTTTGCCTTCCATAAAGAGGGTGCTTTCTTCCTGTATGATCGACTTGCTTCCA 6540

KF113528.2003 CCGGAGACTTTGCCTTCCACAAAGAGGGTGCTTTCTTCCTGTATGATCGACTTGCTTCCA 6536

KC242800.2002 CCGGAGACTTTGCCTTCCACAAAGAGGGTGCTTTCTTCCTGTATGATCGACTTGCTTCCA 6540

KY471090.2001 CCGGAGACTTTGCCTTCCACAAAGAGGGTGCTTTCTTCCTGTATGATCGACTTGCTTCCA 6517

KY471092.2001 CCGGAGACTTTGCCTTCCACAAAGAGGGTGCTTTCTTCCTGTATGATCGACTTGCTTCCA 6517

MH481611.2017 CCGGAGACTTTGCCTTCCACAAAGAGGGTGCTTTCTTCCTGTATGATCGACTTGCTTCCA 6506

MH613311.2017 CCGGAGACTTTGCCTTCCACAAAGAGGGTGCTTTCTTCCTGTATGATCGACTTGCTTCCA 6485

KC242792.1994 CCGGAGACTTTGCCTTCCACAAAGAGGGTGCTTTCTTCCTGTATGATCGACTTGCTTCCA 6540

KC242793.1996 CCGGAGACTTTGCCTTCCACAAAGAGGGTGCTTTCTTCCTGTATGATCGACTTGCTTCCA 6540

KC242794.1996 CCGGAGACTTTGCCTTCCACAAAGAGGGTGCTTTCTTCCTGTATGATCGACTTGCTTCCA 6540

MH121164.1995 CCGGAGACTTTGCCTTCCACAAAGAGGGTGCTTTCTTCCTGTATGACCGACTTGCTTCCA 6540

AY354458.1995 CCGGAGACTTTGCCTTCCACAAAGAGGGTGCTTTCTTCCTGTATGACCGACTTGCTTCCA 6540

KT762962.1995 CCGGAGACTTTGCCTTCCACAAAGAGGGTGCTTTCTTCCTGTATGACCGACTTGCTTCCA 6540

HQ613402.2008 CCGGAGACTTTGCCTTCCACAAAGAGGGTGCTTTCTTCCTGTATGATCGACTTGCTTCCA 6459

KC242789.2007 CCGGAGACTTTGCCTTCCACAAAGAGGGTGCTTTCTTCCTGTATGATCGACTTGCTTCCA 6540

HQ613403.2007 CCGGAGACTTTGCCTTCCACAAAGAGGGTGCTTTCTTCCTGTATGATCGACTTGCTTCCA 6492

KC242785.2007 CCGGAGACTTTGCCTTCCACAAAGAGGGTGCTTTCTTCCTGTATGATCGACTTGCTTCCA 6540

KC242790.2007 CCGGAGACTTTGCCTTCCACAAAGAGGGTGCTTTCTTCCTGTATGATCGACTTGCTTCCA 6540

KU143789.2014 CCGGAGACTTTGCCTTCCACAAAGAGGGTGCTGTCTTCCTGTATGATCGACTTGCTTCCA 6540

KR817168.2014 CCGGAGACTTTGCCTTCCACAAAGAGGGTGCTTTCTTCCTGTATGATCGACTTGCTTCCA 6514

KY426696.2015 CCGGAGACTTTGCCTTCCACAAAGAGGGTGCTTTCTTCCTGTATGATCGACTTGCTTCCA 6540

KR105271.2014 CCGGAGACTTTGCCTTCCACAAAGAGGGTGCTTTCTTCCTGTATGATCGACTTGCTTCCA 6507

KY007522.2016 CCGGAGACTTTGCCTTCCACAAAGAGGGTGCTTTCTTCCTGTATGATCGACTTGCTTCCA 6520

KM034555.2014 CCGGAGACTTTGCCTTCCACAAAGAGGGTGCTTTCTTCCTGTATGATCGACTTGCTTCCA 6532

MH470381.2015 CCGGAGACTTTGCCTTCCACAAAGAGGGTGCTTTCTTCCTGTATGATCGACTTGCTTCCA 6504

MH470382.2015 CCGGAGACTTTGCCTTCCACAAAGAGGGTGCTTTCTTCCTGTATGATCGACTTGCTTCCA 6504

MF102255.2014 CCGGAGACTTTGCCTTCCACAAAGAGGGTGCTTTCTTCCTGTATGATCGACTTGCTTCCA 6517

KJ660348.2014 CCGGAGACTTTGCCTTCCACAAAGAGGGTGCTTTCTTCCTGTATGATCGACTTGCTTCCA 6540

KU143818.2014 CCGGAGACTTTGCCTTCCACAAAGAGGGTGCTTTCTTCCTGTATGATCGACTTGCTTCCA 6540

KT725333.2014 CCGGAGACTTTGCCTTCCACAAAGAGGGTGCTTTCTTCCTGTATGATCGACTTGCTTCCA 6505

KR819004.2014 CCGGAGACTTTGCCTTCCACAAAGAGGGTGCTTTCTTCCTGTATGATCGACTTGCTTCCA 6487

KP271020.2014 CCGGAGACTTTGCCTTCCACAAAGAGGGTGCTTTCTTCCTGTATGATCGACTTGCTTCCA 6487

KM519951.2014 CCGGAGACTTTGCCTTCCACAAAGAGGGTGCTTTCTTCCTGTATGATCGACTTGCTTCCA 6537

MH733488.2018 CCGGAGACTTTGCCTTCCACAAAGAGGGTGCTTTCTTCCTGTATGATCGACTTGCTTCCA 6531

MH733491.2018 CCGGAGACTTTGCCTTCCACAAAGAGGGTGCTTTCTTCCTGTATGATCGACTTGCTTCCA 6526

MH733478.2018 CCGGAGACTTTGCCTTCCACAAAGAGGGTGCTTTCTTCCTGTATGATCGACTTGCTTCCA 6529

MK007330.2018 CCGGAGACTTTGCCTTCCACAAAGAGGGTGCTTTCTTCCTGTATGATCGACTTGCTTCCA 6528

MK007344.2018 CCGGAGACTTTGCCTTCCACAAAGAGGGTGCTTTCTTCCTGTATGATCGACTTGCTTCCA 6522

******************* ************ ************* *************

AY142960.1976 CAGTTATCTACCGAGGAACGACTTTCGCTGAAGGTGTCGTTGCATTTCTGATACTGCCCC 6600

KC242791.1977 CAGTTATCTACCGAGGAACGACTTTCGCTGAAGGTGTCGTTGCATTTCTGATACTGCCCC 6600

AF499101.1976 CAGTTATCTACCGAGGAACGACTTTCGCTGAAGGTGTCGTTGCATTTCTGATACTGCCCC 6600

KF113528.2003 CAGTTATCTACCGAGGAACGACTTTCGCTGAAGGTGTCGTTGCATTTCTGATACTGCCCC 6596

KC242800.2002 CAGTTATCTACCGAGGAACGACTTTCGCTGAAGGTGTCGTTGCATTTCTGATACTGCCCC 6600

KY471090.2001 CAGTTATCTACCGAGGAACGACTTTCGCTGAAGGTGTCGTTGCATTTCTGATACTGCCCC 6577

KY471092.2001 CAGTTATCTACCGAGGAACGACTTTCGCTGAAGGTGTCGTTGCATTTCTGATACTGCCCC 6577

MH481611.2017 CAGTTATCTACCGAGGAACGACTTTCGCTGAAGGTGTTGTTGCATTTCTGATACTGCCCC 6566

MH613311.2017 CAGTTATCTACCGAGGAACGACTTTCGCTGAAGGTGTTGTTGCATTTCTGATACTGCCCC 6545

KC242792.1994 CAGTTATCTACCGAGGAACGACTTTCGCTGAAGGTGTCGTGGCATTTCTGATACTGCCCC 6600

KC242793.1996 CAGTTATCTACCGAGGAACGACTTTCGCTGAAGGTGTCGTGGCATTTCTGATACTGCCCC 6600

KC242794.1996 CAGTTATCTACCGAGGAACGACTTTCGCTGAAGGTGTCGTGGCATTTCTGATACTGCCCC 6600

MH121164.1995 CAGTTATCTACCGAGGAACGACTTTCGCTGAAGGTGTCGTTGCATTTCTGATACTGCCCC 6600

AY354458.1995 CAGTTATCTACCGAGGAACGACTTTCGCTGAAGGTGTCGTTGCATTTCTGATACTGCCCC 6600

KT762962.1995 CAGTTATCTACCGAGGAACGACTTTCGCTGAAGGTGTCGTTGCATTTCTGATACTGCCCC 6600

HQ613402.2008 CAGTTATTTACCGAGGGACGACTTTCGCTGAAGGTGTCGTTGCATTTCTGATACTGCCCC 6519

KC242789.2007 CAGTTATTTACCGAGGGACGACTTTCGCTGAAGGTGTCGTTGCATTTCTGATACTGCCCC 6600

HQ613403.2007 CAGTTATTTACCGAGGGACGACTTTCGCTGAAGGTGTCGTTGCATTTCTGATACTGCCCC 6552

KC242785.2007 CAGTTATTTACCGAGGGACGACTTTCGCTGAAGGTGTCGTTGCATTTCTGATACTGCCCC 6600

KC242790.2007 CAGTTATTTACCGAGGGACGACTTTCGCTGAAGGTGTCGTTGCATTTCTGATACTGCCCC 6600

KU143789.2014 CAGTTATCTACCGAGGAACGACTTTCGCTGAAGGTGTCGTTGCATTTCTGATACTGCCCC 6600

KR817168.2014 CAGTTATCTACCGAGGAACGACTTTCGCTGAAGGTGTCGTTGCATTTCTGATACTGCCCC 6574

KY426696.2015 CAGTTATCTACCGAGGAACGACTTTCGCTGAAGGTGTCGTTGCATTTCTGATACTGCCCC 6600

KR105271.2014 CAGTTATCTACCGAGGAACGACTTTCGCTGAAGGTGTCGTTGCATTTCTGATACTGCCCC 6567

KY007522.2016 CAGTTATCTACCGAGGAACGACTTTCGCTGAAGGTGTCGTTGCATTTCTGATACTGCCCC 6580

KM034555.2014 CAGTTATCTACCGAGGAACGACTTTCGCTGAAGGTGTCGTTGCATTTCTGATACTGCCCC 6592

MH470381.2015 CAGTTATCTACCGAGGAACGACTTTCGCTGAAGGTGTCGTTGCATTTCTGATACTGCCCC 6564

MH470382.2015 CAGTTATCTACCGAGGAACGACTTTCGCTGAAGGTGTCGTTGCATTTCTGATACTGCCCC 6564

MF102255.2014 CAGTTATCTACCGAGGAACGACTTTCGCTGAAGGTGTCGTTGCATTTCTGATACTGCCCC 6577

KJ660348.2014 CAGTTATCTACCGAGGAACGACTTTCGCTGAAGGTGTCGTTGCATTTCTGATACTGCCCC 6600

KU143818.2014 CAGTTATCTACCGAGGAACGACTTTCGCTGAAGGTGTCGTTGCATTTCTGATACTGCCCC 6600

KT725333.2014 CAGTTATCTACCGAGGAACGACTTTCGCTGAAGGTGTCGTTGCATTTCTGATACTGCCCC 6565

KR819004.2014 CAGTTATCTACCGAGGAACGACTTTCGCTGAAGGTGTCGTCGCATTTCTGATACTGCCCC 6547

KP271020.2014 CAGTTATCTACCGAGGAACGACTTTCGCTGAAGGTGTCGTTGCATTTCTGATACTGCCCC 6547

KM519951.2014 CAGTTATCTACCGAGGAACGACTTTCGCTGAAGGTGTCGTTGCATTTCTGATACTGCCCC 6597

MH733488.2018 CGGTTATCTACCGAGGGACGACTTTCGCTGAAGGTGTCGTTGCATTTCTGATACTGCCCC 6591

MH733491.2018 CGGTTATCTACCGAGGGACGACTTTCGCTGAAGGTGTCGTTGCATTTCTGATACTGCCCC 6586

MH733478.2018 CGGTTATCTACCGAGGGACGACTTTCGCTGAAGGTGTCGTTGCATTTCTGATACTGCCCC 6589

MK007330.2018 CAGTTATCTACCGAGGAACGACTTTCGCTGAAGGTGTCGTTGCATTTCTGATACTGCCCC 6588

MK007344.2018 CAGTTATCTACCGAGGAACGACTTTCGCTGAAGGTGTCGTTGCATTTCTGATACTGCCCC 6582

* ***** ******** ******************** ** *******************

AY142960.1976 AAGCTAAGAAGGACTTCTTCAGCTCACACCCCTTGAGAGAGCCGGTCAATGCAACGGAGG 6660

KC242791.1977 AAGCTAAGAAGGACTTCTTCAGCTCACACCCCTTGAGAGAGCCGGTCAATGCAACGGAGG 6660

AF499101.1976 AAGCTAAGAAGGACTTCTTCAGCTCACACCCCTTGAGAGAGCCGGTCAATGCAACGGAGG 6660

KF113528.2003 AAGCTAAGAAGGACTTCTTCAGCTCACACCCCTTAAGAGAGCCGGTCAATGCAACGGAGG 6656

KC242800.2002 AAGCTAAGAAGGACTTCTTCAGCTCACACCCCTTGAGAGAGCCGGTCAATGCAACGGAGG 6660

KY471090.2001 AAGCTAAGAAGGACTTCTTCAGCTCACACCCCTTGAGAGAGCCGGTCAATGCAACGGAGG 6637

KY471092.2001 AAGCTAAGAAGGACTTCTTCAGCTCACACCCCTTGAGAGAGCCGGTCAATGCAACGGAGG 6637

MH481611.2017 AAGCTAAGAAGGACTTCTTCAGCTCACACCCCTTGAGAGAGCCGGTCAATGCGACGGAGG 6626

MH613311.2017 AAGCTAAGAAGGACTTCTTCAGCTCACACCCCTTGAGAGAGCCGGTCAATGCGACGGAGG 6605

KC242792.1994 AAGCTAAGAAGGACTTCTTCAGCTCACACCCCTTGAGAGAGCCGGTCAATGCAACGGAGG 6660

KC242793.1996 AAGCTAAGAAGGACTTCTTCAGCTCACACCCCTTGAGAGAGCCGGTCAATGCAACGGAGG 6660

KC242794.1996 AAGCTAAGAAGGACTTCTTCAGCTCACACCCTTTGAGAGAGCCGGTCAATGCAACGGAGG 6660

MH121164.1995 AAGCTAAGAAGGACTTCTTCAGCTCACACCCCTTGAGAGAGCCGGTCAATGCAACGGAGG 6660

AY354458.1995 AAGCTAAGAAGGACTTCTTCAGCTCACACCCCTTGAGAGAGCCGGTCAATGCAACGGAGG 6660

KT762962.1995 AAGCTAAGAAGGACTTCTTCAGCTCACACCCCTTGAGAGAGCCGGTCAATGCAACGGAGG 6660

HQ613402.2008 AAGCTAAGAAGGACTTCTTCAGCTCACACCCCTTGAGAGAGCCGGTCAATGCAACGGAGG 6579

KC242789.2007 AAGCTAAGAAGGATTTCTTCAGCTCACACCCCTTGAGAGAGCCGGTCAATGCAACGGAGG 6660

HQ613403.2007 AAGCTAAGAAGGACTTCTTCAGCTCACACCCCTTGAGAGAGCCGGTCAATGCAACGGAGG 6612

KC242785.2007 AAGCTAAGAAGGACTTCTTCAGCTCACACCCCTTGAGAGAGCCGGTCAATGCAACGGAGG 6660

KC242790.2007 AAGCTAAGAAGGACTTCTTCAGCTCACACCCCTTGAGAGAGCCGGTCAATGCAACGGAGG 6660

KU143789.2014 AAGCTAAGAAGGACTTCTTCAGCTCACACCCCTTGAGAGAGCCGGTCAATGCAACGGAGG 6660

KR817168.2014 AAGCTAAGAAGGACTTCTTCAGCTCACACCCCTTGAGAGAGCCGGTCAATGCAACGGAGG 6634

KY426696.2015 AAGCTAAGAAGGACTTCTTCAGCTCACACCCCTTGAGAGAGCCGGTCAATGCAACGGAGG 6660

KR105271.2014 AAGCTAAGAAGGACTTCTTCAGCTCACACCCCTTGAGAGAGCCGGTCAATGCAACGGAGG 6627

KY007522.2016 AAGCTAAGAAGGACTTCTTCAGCTCACACCCCTTGAGAGAGCCGGTCAATGCAACGGAGG 6640

KM034555.2014 AAGCTAAGAAGGACTTCTTCAGCTCACACCCCTTGAGAGAGCCGGTCAATGCAACGGAGG 6652

MH470381.2015 AAGCTAAGAAGGACTTCTTCAGCTCACACCCCTTGAGAGAGCCGGTCAATGCAACGGAGG 6624

MH470382.2015 AAGCTAAGAAGGACTTCTTCAGCTCACACCCCTTGAGAGAGCCGGTCAATGCAACGGAGG 6624

MF102255.2014 AAGCTAAGAAGGACTTCTTCAGCTCACACCCCTTGAGAGAGCCGGTCAATGCAACGGAGG 6637

KJ660348.2014 AAGCTAAGAAGGACTTCTTCAGCTCACACCCCTTGAGAGAGCCGGTCAATGCAACGGAGG 6660

KU143818.2014 AAGCTAAGAAGGACTTCTTCAGCTCACACCCCTTGAGAGAGCCGGTCAATGCAACGGAGG 6660

KT725333.2014 AAGCTAAGAAGGACTTCTTCAGCTCACACCCCTTGAGAGAGCCGGTCAATGCAACGGAGG 6625

KR819004.2014 AAGCTAAGAAGGACTTCTTCAGCTCACACCCCTTGAGAGAGCCGGTCAATGCAACGGAGG 6607

KP271020.2014 AAGCTAAGAAGGACTTCTTCAGCTCACACCCCTTGAGAGAGCCGGTCAATGCAACGGAGG 6607

KM519951.2014 AAGCTAAGAAGGACTTCTTCAGCTCACACCCCTTGAGAGAGCCGGTCAATGCAACGGAGG 6657

MH733488.2018 AAGCTAAGAAGGACTTCTTCAGCTCACACCCCTTGAGAGAGCCGGTCAATGCAACGGAGG 6651

MH733491.2018 AAGCTAAGAAGGACTTCTTCAGCTCACACCCCTTGAGAGAGCCGGTCAATGCAACGGAGG 6646

MH733478.2018 AAGCTAAGAAGGACTTCTTCAGCTCACACCCCTTGAGAGAGCCGGTCAATGCAACGGAGG 6649

MK007330.2018 AAGCTAAGAAGGACTTCTTCAGCTCACACCCCTTGAGAGAGCCGGTCAATGCAACGGAGG 6648

MK007344.2018 AAGCTAAGAAGGACTTCTTCAGCTCACACCCCTTGAGAGAGCCGGTCAATGCAACGGAGG 6642

************* ***************** ** ***************** *******

AY142960.1976 ACCCGTCTAGTGGCTACTATTCTACCACAATTAGATATCAGGCTACCGGTTTTGGAACCA 6720

KC242791.1977 ACCCGTCTAGTGGCTACTATTCTACCACAATTAGATATCAGGCTACCGGTTTTGGAACCA 6720

AF499101.1976 ACCCGTCTAGTGGCTACTATTCTACCACAATTAGATATCAGGCTACCGGTTTTGGAACCA 6720

KF113528.2003 ACCCGTCCAGTGGCTACTATTCTACCACAATTAGATATCAGGCTACCGGTTTTGGAACCA 6716

KC242800.2002 ACCCGTCCAGTGGCTACTATTCTACCACAATTAGATATCAGGCTACCGGTTTTGGAACCA 6720

KY471090.2001 ACCCGTCCAGTGGCTACTATTCTACCACAATTAGATATCAGGCTACCGGTTTTGGAACCA 6697

KY471092.2001 ACCCGTCCAGTGGCTACTATTCTACCACAATTAGATATCAGGCTACCGGTTTTGGAACCA 6697

MH481611.2017 ACCCGTCTAGTGGCTACTATTCTACCACAATTAGATATCAGGCTACCGGTTTTGGAACCA 6686

MH613311.2017 ACCCGTCTAGTGGCTACTATTCTACCACAATTAGATATCAGGCTACCGGTTTTGGAACCA 6665

KC242792.1994 ACCCGTCTAGTGGCTACTATTCTACCACAATTAGATATCAGGCTACCGGCTTTGGAACCA 6720

KC242793.1996 ACCCGTCTAGTGGCTACTATTCTACCACAATTAGATATCAGGCTACCGGCTTTGGAACCA 6720

KC242794.1996 ACCCGTCTAGTGGCTACTATTCTACCACAATTAAATATCAGGCTACCGGCTTTGGAACCA 6720

MH121164.1995 ACCCGTCTAGTGGCTACTATTCTACCACAATTAGATATCAAGCTACCGGTTTTGGAACCA 6720

AY354458.1995 ACCCGTCTAGTGGCTACTATTCTACCACAATTAGATATCAAGCTACCGGTTTTGGAACCA 6720

KT762962.1995 ACCCGTCTAGTGGCTACTATTCTACCACAATTAGATATCAAGCTACCGGTTTTGGAACCA 6720

HQ613402.2008 ACCCGTCTAGTGGCTACTATTCTACCACAATTAGATATCAGGCTACCGGTTTTGGAACCA 6639

KC242789.2007 ACCCGTCTAGTGGCTACTATTCTACCACAATTAGATATCAGGCTACCGGTTTTGGAACCA 6720

HQ613403.2007 ACCCGTCTAGTGGCTACTATTCTACCACAATTAGATATCAGGCTACCGGTTTTGGAACCA 6672

KC242785.2007 ACCCGTCTAGTGGCTACTATTCTACCACAATTAGATATCAGGCTACCGGTTTTGGAACCA 6720

KC242790.2007 ACCCGTCTAGTGGCTACTATTCTACCACAATTAGATATCAGGCTACCGGTTTTGGAACCA 6720

KU143789.2014 ACCCGTCGAGTGGCTATTATTCTACCACAAGTAGATATCAGGCTACCGGTTTTAGAACTA 6720

KR817168.2014 ACCCGTCGAGTGGCTATTATTCTACCACAATTAGATATCAGGCTACCGGTTTTGGAACTA 6694

KY426696.2015 ACCCGTCGAGTGGCTATTATTCTACCACAATTAGATATCAGGCTACCGGTTTTGGAACTA 6720

KR105271.2014 ACCCGTCGAGTGGCTATTATTCTACCACAATTAGATATCAGGCTACCGGTTTTGGAACTA 6687

KY007522.2016 ACCCGTCGAGTGGCTATTATTCTACCACAATTAGATATCAGGCTACCGGTTTTGGAACTA 6700

KM034555.2014 ACCCGTCGAGTGGCTATTATTCTACCACAATTAGATATCAGGCTACCGGTTTTGGAACTA 6712

MH470381.2015 ACCCGTCGAGTGGCTATTATTCTACCACAATTAGATATCAGGCTACCGGTTTTGGAACTA 6684

MH470382.2015 ACCCGTCGAGTGGCTATCATTCTACCACAATTAGATATCAGGCTACCGGTTTTGGAACTA 6684

MF102255.2014 ACCCGTCGAGTGGCTATTATTCTACCACAATTAGATATCAGGCTACCGGTTTTGGAACTA 6697

KJ660348.2014 ACCCGTCGAGTGGCTATTATTCTACCACAATTAGATATCAGGCTACCGGTTTTGGAACTA 6720

KU143818.2014 ACCCGTCGAGTGGCTATTATTCTACCACAATTAGATATCAGGCTACCGGTTTTGGAACTA 6720

KT725333.2014 ACCCGTCGAGTGGCTATTATTCTACCACAATTAGATATCAGGCTACCGGTTTTGGAACTA 6685

KR819004.2014 ACCCGTCTAGTGGTTACTATTCTACCACAATTAGATATCAGGCTACCGGTTTTGGAACCA 6667

KP271020.2014 ACCCGTCTAGTGGTTACTATTCTACCACAATTAGATATCAGGCTACCGGTTTTGGAACCA 6667

KM519951.2014 ACCCGTCTAGTGGTTACTATTCTACCACAATTAGATATCAGGCTACCGGTTTTGGAACCA 6717

MH733488.2018 ACCCGTCTAGTGGTTACTATTCTACCACAATTAGATATCAGGCTACCGGTTTTGGAACCA 6711

MH733491.2018 ACCCGTCTAGTGGTTACTATTCTACCACAATTAGATATCAGGCTACCGGTTTTGGAACCA 6706

MH733478.2018 ACCCGTCTAGTGGTTACTATTCTACCACAATTAGATATCAGGCTACCGGTTTTGGAACCA 6709

MK007330.2018 ACCCGTCTAGTGGCTACTACTCTACCACAATTAGATATCAGGCTACCGGTTTTGGAACCA 6708

MK007344.2018 ACCCGTCTAGTGGCTACTACTCTACCACAATTAGATATCAGGCTACCGGTTTTGGAACCA 6702

******* ***** ** * ********** ** ****** ******** *** **** *

AY142960.1976 ATGAGACAGAGTACTTGTTCGAGGTTGACAATTTGACCTACGTCCAACTTGAATCAAGAT 6780

KC242791.1977 ATGAGACAGAGTACTTGTTCGAGGTTGACAATTTGACCTACGTCCAACTTGAATCAAGAT 6780

AF499101.1976 ATGAGACAGAGTACTTGTTCGAGGTTGACAATTTGACCTACGTCCAACTTGAACCAAGAT 6780

KF113528.2003 ATGAGACGGAGTACTTGTTCGAGGTTGACAATTTGACCTACGTCCAACTTGAATCAAGAT 6776

KC242800.2002 ATGAGACGGAGTACTTGTTCGAGGTTGACAATTTGACCTACGTCCAACTTGAATCAAGAT 6780

KY471090.2001 ATGAGACGGAGTACTTGTTCGAGGTTGACAATTTGACCTACGTCCAACTTGAATCAAGAT 6757

KY471092.2001 ATGAGACGGAGTACTTGTTCGAGGTTGACAATTTGACCTACGTCCAACTTGAATCAAGAT 6757

MH481611.2017 ATGAGACAGAGTACTTGTTCGAGGTTGACAATTTGACCTACGTCCAACTTGAATCAAGAT 6746

MH613311.2017 ATGAGACAGAGTACTTGTTCGAGGTTGACAATTTGACCTACGTCCAACTTGAATCAAGAT 6725

KC242792.1994 ATGAGACAGAGTATTTGTTCGAGGTTGACAATTTGACCTACGTCCAACTTGAATCAAGAT 6780

KC242793.1996 ATGAGACAGAGTATTTGTTCGAGGTTGACAATTTGACCTACGTCCAACTTGAATCAAGAT 6780

KC242794.1996 ATGAGACAGAGTATTTGTTCGAGGTTGACAATTTGACCTACGTCCAACTTGAATCAAGAT 6780

MH121164.1995 ATGAGACAGAGTATTTGTTCGAGGTTGACAATTTGACCTACGTCCAACTTGAATCAAGAT 6780

AY354458.1995 ATGAGACAGAGTATTTGTTCGAGGTTGACAATTTGACCTACGTCCAACTTGAATCAAGAT 6780

KT762962.1995 ATGAGACAGAGTATTTGTTCGAGGTTGACAATTTGACCTACGTCCAACTTGAATCAAGAT 6780

HQ613402.2008 ATGAGACAGAGTACTTGTTCGAGGTTGACAATTTGACCTACGTCCAACTTGAATCAAGAT 6699

KC242789.2007 ATGAGACAGAGTACTTGTTCGAGGTTGACAATTTGACCTACGTCCAACTTGAATCAAGAT 6780

HQ613403.2007 ATGAGACAGAGTACTTGTTCGAGGTTGACAATTTGACCTACGTCCAACTTGAATCAAGAT 6732

KC242785.2007 ATGAGACAGAGTACTTGTTCGAGGTTGACAATTTGACCTACGTCCAACTTGAATCAAGAT 6780

KC242790.2007 ATGAGACAGAGTACTTGTTCGAGGTTGACAATTTGACCTACGTCCAACTTGAATCAAGAT 6780

KU143789.2014 ATGAGACAGAGTACTTGTTCGAGGTTGACAATTTGACCTACGTCCAACTTGAATCAAGAT 6780

KR817168.2014 ATGAGACAGAGTACTTGTTCGAGGTTGACAATTTGACCTACGTCCAACTTGAATCAAGAT 6754

KY426696.2015 ATGAGACAGAGTACTTGTTCGAGGTTGACAATTTGACCTACGTCCAACTTGAATCAAGAT 6780

KR105271.2014 ATGAGACAGAGTACTTGTTCGAGGTTGACAATTTGACCTACGTCCAACTTGAATCAAGAT 6747

KY007522.2016 ATGAGACAGAGTACTTGTTCGAGGTTGACAATTTGACCTACGTCCAACTTGAATCAAGAT 6760

KM034555.2014 ATGAGACAGAGTACTTGTTCGAGGTTGACAATTTGACCTACGTCCAACTTGAATCAAGAT 6772

MH470381.2015 ATGAGACAGAGTACTTGTTCGAGGTTGACAATTTGACCTACGTCCAACTTGAATCAAGAT 6744

MH470382.2015 ATGAGACAGAGTACTTGTTCGAGGTTGACAATTTGACCTACGTCCAACTTGAATCAAGAT 6744

MF102255.2014 ATGAGACAGAGTACTTGTTCGAGGTTGACAATTTGACCTACGTCCAACTTGAATCAAGAT 6757

KJ660348.2014 ATGAGACAGAGTACTTGTTCGAGGTTGACAATTTGACCTACGTCCAACTTGAATCAAGAT 6780

KU143818.2014 ATGAGGCAGAGTACTTGTTCGAGGTTGACAATTTGACCTACGTCCAACTTGAATCAAGAT 6780

KT725333.2014 ATGAGACAGAGTACTTGTTCGAGGTTGACAATTTGACCTACGTCCAACTTGAATCAAGAT 6745

KR819004.2014 ATGAGACAGAGTATTTGTTCGAGGTTGACAATTTGACCTACGTCCAACTTGAATCAAGAT 6727

KP271020.2014 ATGAGACAGAGTATTTGTTCGAGGTTGACAATTTGACCTACGTCCAACTTGAATCAAGAT 6727

KM519951.2014 ATGAGACAGAGTATTTGTTCGAGGTTGACAATTTGACCTACGTCCAACTTGAATCAAGAT 6777

MH733488.2018 ATGAGACAGAGTATTTGTTCGAGGTTGACAATTTGACCTACGTCCAACTTGAATCAAGAT 6771

MH733491.2018 ATGAGACAGAGTATTTGTTCGAGGTTGACAATTTGACCTACGTCCAACTTGAATCAAGAT 6766

MH733478.2018 ATGAGACAGAGTATTTGTTCGAGGTTGACAATTTGACCTACGTCCAACTTGAATCAAGAT 6769

MK007330.2018 ATGAGACAGAGTACTTGTTCGAGGTTGACAATTTGACCTATGTCCAACTTGAATCAAGAT 6768

MK007344.2018 ATGAGACAGAGTACTTGTTCGAGGTTGACAATTTGACCTATGTCCAACTTGAATCAAGAT 6762

***** * ***** ************************** ************ ******

AY142960.1976 TCACACCACAGTTTCTGCTCCAGCTGAATGAGACAATATATACAAGTGGGAAAAGGAGCA 6840

KC242791.1977 TCACACCACAGTTTCTGCTCCAGCTGAATGAGACAATATATACAAGTGGGAAAAGGAGCA 6840

AF499101.1976 TCACACCACAGTTTCTGCTCCAGCTGAATGAGACAATATATACAAGTGGGAAAAGGAGCA 6840

KF113528.2003 TCACGCCACAGTTTTTGCTCCAGCTGAATGAGACAATATATGCAAGTGGGAAAAGGAGCA 6836

KC242800.2002 TCACGCCACAGTTTTTGCTCCAGCTGAATGAGACAATATATGCAAGTGGGAAAAGGAGCA 6840

KY471090.2001 TCACGCCACAGTTTTTGCTCCAGCTGAATGAGACAATATATGCAAGTGGGAAAAGGAGCA 6817

KY471092.2001 TCACGCCACAGTTTTTGCTCCAGCTGAATGAGACAATATATGCAAGTGGGAAAAGGAGCA 6817

MH481611.2017 TCACACCACAGTTTCTGCTCCAGCTGAACGAGACAATATATACAAGTGGGAAGAGGAGCA 6806

MH613311.2017 TCACACCACAGTTTCTGCTCCAGCTGAACGAGACAATATATACAAGTGGGAAGAGGAGCA 6785

KC242792.1994 TCACACCACAGTTTCTGCTCCAGCTGAATGAGACAATATATACAAGTGGGAAAAGGAGCA 6840

KC242793.1996 TCACACCACAGTTTCTGCTCCAGCTGAATGAGACAATATATACAAGTGGGAAAAGGAGCA 6840

KC242794.1996 TCACACCACAGTTTCTGCTCCAGCTGAATGAGACAATATATACAAGTGGGAAAAGGAGCA 6840

MH121164.1995 TCACACCACAGTTTCTGCTCCAGCTGAATGAGACAATATATACAAGTGGGAAAAGGAGCA 6840

AY354458.1995 TCACACCACAGTTTCTGCTCCAGCTGAATGAGACAATATATACAAGTGGGAAAAGGAGCA 6840

KT762962.1995 TCACACCACAGTTTCTGCTCCAGCTGAATGAGACAATATATACAAGTGGGAAAAGGAGCA 6840

HQ613402.2008 TCACACCACAATTTCTGCTCCAGCTGAATGAGACAATATATGCAAGTGGGAAAAGGAGCA 6759

KC242789.2007 TCACACCACAGTTTCTGCTCCAGCTGAATGAGACAATATATGCAAGTGGGAAAAGGAGCA 6840

HQ613403.2007 TCACACCACAGTTTCTGCTCCAGCTGAATGAGACAATATATGCAAGTGGGAAAAGGAGCA 6792

KC242785.2007 TCACACCACAGTTTCTGCTCCAGCTGAATGAGACAATATATGCAAGTGGGAAAAGGAGCA 6840

KC242790.2007 TCACACCACAGTTTCTGCTCCAGCTGAATGAGACAATATATGCAAGTGGGAAAAGGAGCA 6840

KU143789.2014 TCACACCACAGTTTCTGCTCCAGCTGAATGAGACAATATATGCAAGTGGGAAGAGGAGCA 6840

KR817168.2014 TCACACCACAGTTTCTGCTCCAGCTGAATGAGACAATATATGCAAGTGGGAAGAGGAGCA 6814

KY426696.2015 TCACACCACAGTTTCTGCTCCAGCTGAATGAGACAATATATGCAAGTGGGAAGAGGAGCA 6840

KR105271.2014 TCACACCACAGTTTCTGCTCCAGCTGAATGAGACAATATATGCAAGTGGGAAGAGGAGCA 6807

KY007522.2016 TCACACCACAGTTTCTGCTCCAGCTGAATGAGACAATATATGCAAGTGGGAAGAGGAGCA 6820

KM034555.2014 TCACACCACAGTTTCTGCTCCAGCTGAATGAGACAATATATGCAAGTGGGAAGAGGAGCA 6832

MH470381.2015 TCACACCACAGTTTCTGCTCCAGCTGAATGAGACAATATATGCAAGTGGGAAGAGGAGCA 6804

MH470382.2015 TCACACCACAGTTTCTGCTCCAGCTGAATGAGACAATATATGCAAGTGGGAAGAGGAGCA 6804

MF102255.2014 TCACACCACAGTTTCTGCTCCAGCTGAATGAGACAATATATGCAAGTGGGAAGAGGAGCA 6817

KJ660348.2014 TCACACCACAGTTTCTGCTCCAGCTGAATGAGACAATATATGCAAGTGGGAAGAGGAGCA 6840

KU143818.2014 TCACACCACAGTTTCTGCTCCAGCTGAATGAGACAATATATGCAAGTGGGAAGAGGAGCA 6840

KT725333.2014 TCACACCACAGTTTCTGCTCCAGCTGAATGAGACAATATATGCAAGTGGGAAGAGGAGCA 6805

KR819004.2014 TCACACCACAGTTTCTGCTCCAGCTGAATGAGACAATATATACAAGTGGGAAAAGGAGCA 6787

KP271020.2014 TCACACCACAGTTTCTGCTCCAGCTGAATGAGACAATATATACAAGTGGGAAAAGGAGCA 6787

KM519951.2014 TCACACCACAGTTTCTGCTCCAGCTGAATGAGACAATATATACAAGTGGGAAAAGGAGCA 6837

MH733488.2018 TCACACCACAGTTTCTGCTCCAGCTGAATGAGACAATATATACAAGTGGGAAAAGGAGCA 6831

MH733491.2018 TCACACCACAGTTTCTGCTCCAGCTGAATGAGACAATATATACAAGTGGGAAAAGGAGCA 6826

MH733478.2018 TCACACCACAGTTTCTGCTCCAGCTGAATGAGACAATATATACAAGTGGGAAAAGGAGCA 6829

MK007330.2018 TCACACCACAGTTTCTGCTCCAGCTGAATGAGACAATATATACAAGTGGGAAGAGGAGCA 6828

MK007344.2018 TCACACCACAGTTTCTGCTCCAGCTGAATGAGACAATATATACAAGTGGGAAGAGGAGCA 6822

**** ***** *** ************* ************ ********** *******

AY142960.1976 ATACCACGGGAAAACTAATTTGGAAGGTCAACCCCGAAATTGATACAACAATCGGGGAGT 6900

KC242791.1977 ATACCACGGGAAAACTAATTTGGAAGGTCAACCCCGAAATTGATACAACAATCGGGGAGT 6900

AF499101.1976 ATACCACGGGAAAACTAATTTGGAAGGTCAACCCCGAAATTGATACAACAATCGGGGAGT 6900

KF113528.2003 ACACCACGGGAAAACTAATTTGGAAGGTCAACCCCGAAATTGATACAACAATCGGGGAGT 6896

KC242800.2002 ACACCACGGGAAAACTAATTTGGAAGGTCAACCCCGAAATTGATACAACAATCGGGGAGT 6900

KY471090.2001 ACACCACGGGAAAACTAATTTGGAAGGTCAACCCCGAAATTGATACAACAATCGGGGAGT 6877

KY471092.2001 ACACCACGGGAAAACTAATTTGGAAGGTCAACCCCGAAATTGATACAACAATCGGGGAGT 6877

MH481611.2017 ATACCACGGGAAAACTAATTTGGAAGGTCAACCCCGAAATTGATACAACAATCGGGGAGT 6866

MH613311.2017 ATACCACGGGAAAACTAATTTGGAAGGTCAACCCCGAAATTGATACAACAATCGGGGAGT 6845

KC242792.1994 ATACCACGGGAAAACTAATTTGGAAGGTCAACCCCGAAATTGATACAACAATCGGGGAGT 6900

KC242793.1996 ATACCACGGGAAAACTAATTTGGAAGGTCAACCCCGAAATTGATACAACAATCGGGGAGT 6900

KC242794.1996 ATACCACGGGAAAACTAATTTGGAAGGTCAACCCCGAAATTGATACAACAATCGGGGAGT 6900

MH121164.1995 ATACCACGGGAAAACTAATTTGGAAGGTCAACCCCGAAATTGATACAACAATCGGGGAGT 6900

AY354458.1995 ATACCACGGGAAAACTAATTTGGAAGGTCAACCCCGAAATTGATACAACAATCGGGGAGT 6900

KT762962.1995 ATACCACGGGAAAACTAATTTGGAAGGTCAACCCCGAAATTGATACAACAATCGGGGAGT 6900

HQ613402.2008 ACACCACGGGAAAACTAATTTGGAAAGTCAACCCCGAAATTGATACAACAATCGGGGAGT 6819

KC242789.2007 ACACCACGGGAAAACTAATTTGGAAAGTCAACCCCGAAATTGATACAACAATCGGGGAGT 6900

HQ613403.2007 ACACCACGGGAAAACTAATTTGGAAAGTCAACCCCGAAATTGATACAACAATCGGGGAGT 6852

KC242785.2007 ACACCACGGGAAAACTAATTTGGAAAGTCAACCCCGAAATTGATACAACAATCGGGGAGT 6900

KC242790.2007 ACACCACGGGAAAACTAATTTGGAAAGTCAACCCCGAAATTGATACAACAATCGGGGAGT 6900

KU143789.2014 ACACCACGGGAAAACTAATTTGGAAGGTCAACCCCGAAATTGATACAACAATCGGGGAGT 6900

KR817168.2014 ACACCACGGGAAAACTAATTTGGAAGGTCAACCCCGAAATTGATACAACAATCGGGGAGT 6874

KY426696.2015 ACACCACGGGAAAACTAATTTGGAAGGTCAACCCCGAAATTGATACAACAATCGGGGAGT 6900

KR105271.2014 ACACCACGGGAAAACTAATTTGGAAGGTCAACCCCGAAATTGATACAACAATCGGGGAGT 6867

KY007522.2016 ACACCACGGGAAAACTAATTTGGAAGGTCAACCCCGAAATTGATACAACAATCGGGGAGT 6880

KM034555.2014 ACACCACGGGAAAACTAATTTGGAAGGTCAACCCCGAAATTGATACAACAATCGGGGAGT 6892

MH470381.2015 ACACCACGGGAAAACTAATTTGGAAGGTCAACCCCGAAATTGATACAACAATCGGGGAGT 6864

MH470382.2015 ACACCACGGGAAAACTAATTTGGAAGGTCAACCCCGAAATTGATACAACAATCGGGGAGT 6864

MF102255.2014 ACACCACGGGAAAACTAATTTGGAAGGTCAACCCCGAAATTGATACAACAATCGGGGAGT 6877

KJ660348.2014 ACACCACGGGAAAACTAATTTGGAAGGTCAACCCCGAAATTGATACAACAATCGGGGAGT 6900

KU143818.2014 ACACCACGGGAAAACTAATTTGGAAGGTCAACCCCGAAATTGATACAACAATCGGGGAGT 6900

KT725333.2014 ACACCACGGGAAAACTAATTTGGAAGGTCAACCCCGAAATTGATACAACAATCGGGGAGT 6865

KR819004.2014 ATACCACGGGAAAACTAATTTGGAAGGTCAACCCCGAAATTGATACAACAATCGGGGAGT 6847

KP271020.2014 ATACCACGGGAAAACTAATTTGGAAGGTCAACCCCGAAATTGATACAACAATCGGGGAGT 6847

KM519951.2014 ATACCACGGGAAAACTAATTTGGAAGGTCAACCCCGAAATTGATACAACAATCGGGGAGT 6897

MH733488.2018 ATACCACGGGAAAACTAATTTGGAAGGTCAACCCCGAAATTGATACAACAATCGGGGAGT 6891

MH733491.2018 ATACCACGGGAAAACTAATTTGGAAGGTCAACCCCGAAATTGATACAACAATCGGGGAGT 6886

MH733478.2018 ATACCACGGGAAAACTAATTTGGAAGGTCAACCCCGAAATTGATACAACAATCGGGGAGT 6889

MK007330.2018 ATACCACGGGAAAACTAATTTGGAAGGTCAACCCCGAAATTGATACAACAATCGGGGAGT 6888

MK007344.2018 ATACCACGGGAAAACTAATTTGGAAGGTCAACCCCGAAATTGATACAACAATCGGGGAGT 6882

* *********************** **********************************

AY142960.1976 GGGCCTTCTGGGAAACTAAAAAAA-CCTCACTAGAAAAATTCGCAGTGAAGAGTTGTCTT 6959 end ssGP

KC242791.1977 GGGCCTTCTGGGAAACTAAAAAAA-CCTCACTAGAAAAATTCGCAGTGAAGAGTTGTCTT 6959

AF499101.1976 GGGCCTTCTGGGAAACTAAAAAAA-CCTCACTAGAAAAATTCGCAGTGAAGAGTTGTCTT 6959

KF113528.2003 GGGCCTTCTGGGAAACTAAAAAAA-CCTCACTAGAAAAATTCGCAGTGAAGAGTTGTCTT 6955

KC242800.2002 GGGCCTTCTGGGAAACTAAAAAAA-CCTCACTAGAAAAATTCGCAGTGAAGAGTTGTCTT 6959

KY471090.2001 GGGCCTTCTGGGAAACTAAAAAAA-CCTCACTAGAAAAATTCGCAGTGAAGAGTTGTCTT 6936

KY471092.2001 GGGCCTTCTGGGAAACTAAAAAAA-CCTCACTAGAAAAATTCGCAGTGAAGAGTTGTCTT 6936

MH481611.2017 GGGCCTTCTGGGAAACTAAAAAAA-CCTCACTAGAAAAATTCGCAGTGAAGAGTTGTCTT 6925

MH613311.2017 GGGCCTTCTGGGAAACTAAAAAAA-CCTCACTAGAAAAATTCGCAGTGAAGAGTTGTCTT 6904

KC242792.1994 GGGCCTTCTGGGAAACTAAAAAAA-CCTCACTAGAAAAATTCGCAGTGAAGAGTTGTCTT 6959

KC242793.1996 GGGCCTTCTGGGAAACTAAAAAAA-CCTCACTAGAAAAATTCGCAGTGAAGAGTTGTCTT 6959

KC242794.1996 GGGCCTTCTGGGAAACTAAAAAAA-CCTCACTAGAAAAATTCGCAGTGAAGAGTTGTCTT 6959

MH121164.1995 GGGCCTTCTGGGAAACTAAAAAAA-CCTCACTAGAAAAATTCGCAGTGAAGAGTTGTCTT 6959

AY354458.1995 GGGCCTTCTGGGAAACTAAAAAAAACCTCACTAGAAAAATTCGCAGTGAAGAGTTGTCTT 6960 slippage

KT762962.1995 GGGCCTTCTGGGAAACTAAAAAAA-CCTCACTAGAAAAATTCGCAGTGAAGAGTTGTCTT 6959

HQ613402.2008 GGGCCTTCTGGGAAACTAAAAAAA-CCTCACTAGAAAAATTCGCAGTGAAGAGTTGTCTT 6878

KC242789.2007 GGGCCTTCTGGGAAACTAAAAAAA-CCTCACTAGAAAAATTCGCAGTGAAGAGTTGTCTT 6959

HQ613403.2007 GGGCCTTCTGGGAAACTAAAAAAA-CCTCACTAGAAAAATTCGCAGTGAAGAGTTGTCTT 6911

KC242785.2007 GGGCCTTCTGGGAAACTAAAAAAA-CCTCACTAGAAAAATTCGCAGTGAAGAGTTGTCTT 6959

KC242790.2007 GGGCCTTCTGGGAAACTAAAAAAA-CCTCACTAGAAAAATTCGCAGTGAAGAGTTGTCTT 6959

KU143789.2014 GGGCCTTCTGGGAAACTAAAAAAA-CCTCACTAGAAAAATTCGCAGTGAAGAGTTGTCTT 6959

KR817168.2014 GGGCCTTCTGGGAAACTAAAAAAA-CCTCACTAGAAAAATTCGCAGTGAAGAGTTGTCTT 6933

KY426696.2015 GGGCCTTCTGGGAAACTAAAAAAA-CCTCACTAGAAAAATTCGCAGTGAAGAGTTGTCTT 6959

KR105271.2014 GGGCCTTCTGGGAAACTAAAAAAA-CCTCACTAGAAAAATTCGCAGTGAAGAGTTGTCTT 6926

KY007522.2016 GGGCCTTCTGGGAAACTAAAAAAA-CCTCACTAGAAAAATTCGCAGTGAAGAGTTGTCTT 6939

KM034555.2014 GGGCCTTCTGGGAAACTAAAAAAA-CCTCACTAGAAAAATTCGCAGTGAAGAGTTGTCTT 6951

MH470381.2015 GGGCCTTCTGGGAAACTAAAAAAA-CCTCACTAGAAAAATTCGCAGTGAAGAGTTGTCTT 6923

MH470382.2015 GGGCCTTCTGGGAAACTAAAAAAA-CCTCACTAGAAAAATTCGCAGTGAAGAGTTGTCTT 6923

MF102255.2014 GGGCCTTCTGGGAAACTAAAAAAA-CCTCACTAGAAAAATTCGCAGTGAAGAGTTGTCTT 6936

KJ660348.2014 GGGCCTTCAGGGAAACTAAAAAAA-CCTCACTAGAAAAATTCGCAGTGAAGAGTTGTCTT 6959

KU143818.2014 GGGCCTTCTGGGAAACTAAAAAAA-CCTCACTAGAAAAATTCGCAGTGAAGAGTTGTCTT 6959

KT725333.2014 GGGCCTTCTGGGAAACTAAAAAAA-CCTCACTAGAAAAATTCGCAGTGAAGAGTTGTCTT 6924

KR819004.2014 GGGCCTTCTGGGAAACTAAAAAAA-CCTCACTAGAAAAATTCGCAGTGAAGAGTTGTCTT 6906

KP271020.2014 GGGCCTTCTGGGAAACTAAAAAAA-CCTCACTAGAAAAATTCGCAGTGAAGAGTTGTCTT 6906

KM519951.2014 GGGCCTTCTGGGAAACTAAAAAAA-CCTCACTAGAAAAATTCGCAGTGAAGAGTTGTCTT 6956

MH733488.2018 GGGCCTTCTGGGAAACTAAAAAAA-CCTCACTAGAAAAATTCGCAGTGAAGAGTTGTCTT 6950

MH733491.2018 GGGCCTTCTGGGAAACTAAAAAAA-CCTCACTAGAAAAATTCGCAGTGAAGAGTTGTCTT 6945

MH733478.2018 GGGCCTTCTGGGAAACTAAAAAAA-CCTCACTAGAAAAATTCGCAGTGAAGAGTTGTCTT 6948

MK007330.2018 GGGCCTTCTGGGAAACTAAAAAAA-CCTCACTAGAAAAATTCGCAGTGAAGAGTTGTCTT 6947

MK007344.2018 GGGCCTTCTGGGAAACTAAAAAAA-CCTCACTAGAAAAATTCGCAGTGAAGAGTTGTCTT 6941

******** *************** ***********************************

AY142960.1976 TCACAGTTGTATCAAACGGAGCCAAAAACATCAGTGGTCAGAGTCCGGCGCGAACTTCTT 7019

KC242791.1977 TCACAGTTGTATCAAACGGAGCCAAAAACATCAGTGGTCAGAGTCCGGCGCGAACTTCTT 7019

AF499101.1976 TCACAGTTGTATCAAACGGAGCCAAAAACATCAGTGGTCAGAGTCCGGCGCGAACTTCTT 7019

KF113528.2003 TCACAGCTGTATCAAACGGAGCCAAAGACATCAGTGGTCAGAGTCCGGCGCGAACTTCTT 7015

KC242800.2002 TCACAGCTGTATCAAACGGAGCCAAAGACATCAGTGGTCAGAGTCCGGCGCGAACTTCTT 7019

KY471090.2001 TCACAGCTGTATCAAACGGAGCCAAAGACATCAGTGGTCAGAGTCCGGCGCGAACTTCTT 6996

KY471092.2001 TCACAGCTGTATCAAACGGAGCCAAAGACATCAGTGGTCAGAGTCCGGCGCGAACTTCTT 6996

MH481611.2017 TCACAGCTGTATCAAACGGAGCCAAAAACATCAGTGGTCAGAGTCCGGCGCGAACTTCTT 6985

MH613311.2017 TCACAGCTGTATCAAACGGAGCCAAAAACATCAGTGGTCAGAGTCCGGCGCGAACTTCTT 6964

KC242792.1994 TCACAGCTGTATCAAACAGAGCCAAAAACATCAGTGGTCAGAGTCCGGCGCGAACTTCTT 7019

KC242793.1996 TCACAGCTGTATCAAACAGAGCCAAAAACATCAGTGGTCAGAGTCCGGCGCGAACTTCTT 7019

KC242794.1996 TCACAGCTGTATCAAACAGAGCCAAAAACATCAGTGGTCAGAGTCCGGCGCGAACTTCTT 7019

MH121164.1995 TCACAGCTGTATCAAACAGAGCCAAAAACATCAGTGGTCAGAGTCCGGCGCGAACTTCTT 7019

AY354458.1995 TCACAGCTGTATCAAACAGAGCCAAAAACATCAGTGGTCAGAGTCCGGCGCGAACTTCTT 7020

KT762962.1995 TCACAGCTGTATCAAACAGAGCCAAAAACATCAGTGGTCAGAGTCCGGCGCGAACTTCTT 7019

HQ613402.2008 TCACAGCTGTATCAAACGGAGCCAAAAACCTCAGTGGTCAGAGTCCGGCGCGAACTTCTT 6938

KC242789.2007 TCACAGCTGTATCAAACGGAGCCAAAAACCTCAGTGGTCAGAGTCCGGCGCGAACTTCTT 7019

HQ613403.2007 TCACAGCTGTATCAAACGGAGCCAAAAACCTCAGTGGTCAGAGTCCGGCGCGAACTTCTT 6971

KC242785.2007 TCACAGCTGTATCAAACGGAGCCAAAAACCTCAGTGGTCAGAGTCCGGCGCGAACTTCTT 7019

KC242790.2007 TCACAGCTGTATCAAACGGAGCCAAAAACCTCAGTGGTCAGAGTCCGGCGCGAACTTCTT 7019

KU143789.2014 TCACAGCTGTATCAAACGGACCCAAAAACATCAGTGGTCAGAGTCCGGCGCGAACTTCTT 7019

KR817168.2014 TCACAGCTGTATCAAACGGACCCAAAAACATCAGTGGTCAGAGTCCGGCGCGAACTTCTT 6993

KY426696.2015 TCACAGCTGTATCAAACGGACCCAAAAACATCAGTGGTCAGAGTCCGGCGCGAACTTCTT 7019

KR105271.2014 TCACAGCTGTATCAAACGGACCCAAAAACATCAGTGGTCAGAGTCCGGCGCGAACTTCTT 6986

KY007522.2016 TCACAGCTGTATCAAACGGACCCAAAAACATCAGTGGTCAGAGTCCGGCGCGAACTTCTT 6999

KM034555.2014 TCACAGCTGTATCAAACGGACCCAAAAACATCAGTGGTCAGAGTCCGGCGCGAACTTCTT 7011

MH470381.2015 TCACAGCTGTATCAAACGGACCCAAAAACATCAGTGGTCAGAGTCCGGCGCGAACTTCTT 6983

MH470382.2015 TCACAGCTGTATCAAACGGACCCAAAAACATCAGTGGTCAGAGTCCGGCGCGAACTTCTT 6983

MF102255.2014 TCACAGCTGTATCAAATGGACCCAAAAACATCAGTGGTCAGAGTCCGGCGCGAACTTCTT 6996

KJ660348.2014 TCACAGCTGTATCAAACGGACCCAAAAACATCAGTGGTCAGAGTCCGGCGCGAACTTCTT 7019

KU143818.2014 TCACAGCTGTATCAAACGGACCCAAAAACATCAGTGGTCAGAGTCCGGCGCGAACTTCTT 7019

KT725333.2014 TCACAGCTGTATCAAACGGACCCAAAAACATCAGTGGTCAGAGTCCGGCGCGAACTTCTT 6984

KR819004.2014 TCACAGCTGTATCAAACAGAGCCAAAAACATCAGTGGTCAGAGTCCGGCGCGAACTTCTT 6966

KP271020.2014 TCACAGCTGTATCAAACAGAGCCAAAAACATCAGTGGTCAGAGTCCGGCGCGAACTTCTT 6966

KM519951.2014 TCACAGCTGTATCAAACAGAGCCAAAAACATCAGTGGTCAGAGTCCGGCGCGAACTTCTT 7016

MH733488.2018 TCACAGCTGTATCAAACAGAGCCAAAAACATCAGTGGTCAGAGTCCGGCGCGAACTTCTT 7010

MH733491.2018 TCACAGCTGTATCAAACAGAGCCAAAAACATCAGTGGTCAGAGTCCGGCGCGAACTTCTT 7005

MH733478.2018 TCACAGCTGTATCAAACAGAGCCAAAAACATCAGTGGTCAGAGTCCGGCGCGAACTTCTT 7008

MK007330.2018 TCACAGCTGTATCAAACGGAGCCAAAAACATCAGTGGTCAGAGTCCGGCGCGAACTTCTT 7007

MK007344.2018 TCACAGCTGTATCAAACGGAGCCAAAAACATCAGTGGTCAGAGTCCGGCGCGAACTTCTT 7001

****** ********* ** ***** ** ******************************

AY142960.1976 CCGACCCAGGGACCAACACAACAACTGAAGACCACAAAATCATGGCTTCAGAAAATTCCT 7079

KC242791.1977 CCGACCCAGGGACCAACACAACAACTGAAGACCACAAAATCATGGCTTCAGAAAATTCCT 7079

AF499101.1976 CCGACCCAGGGACCAACACAACAACTGAAGACCACAAAATCATGGCTTCAGAAAATTCCT 7079

KF113528.2003 CCGACCCAGAGACCTACACAACAACTGGAGACCACAAAATCATGGCTTCAGAAGATTCCT 7075

KC242800.2002 CCGACCCAGAGACCTACACAACAACTGAAGACCACAAAATCATGGCTTCAGAAAATTCCT 7079

KY471090.2001 CCGACCCAGAGACCTACACAACAACTGAAGACCACAAAATCATGGCTTCAGAAAATTCCT 7056

KY471092.2001 CCGACCCAGAGACCTACACAACAACTGAAGACCACAAAATCATGGCTTCAGAAAATTCCT 7056

MH481611.2017 CCGACCCAGGGACCAACACAACAACTGAAGACCACAAAATCATGGCTTCAGAAAATTCCT 7045

MH613311.2017 CCGACCCAGGGACCAACACAACAACTGAAGACCACAAAATCATGGCTTCAGAAAATTCCT 7024

KC242792.1994 CCGACCCAGGGACCAACACAACAACTGAAGACCACAAAATCATGGCTTCAGAAAATTCCT 7079

KC242793.1996 CCGACCCAGGGACCAACACAACAACTGAAGACCACAAAATCATGGCTTCAGAAAATTCCT 7079

KC242794.1996 CCGACCCAGGGACCAACACAACAACTGAAGACCACAAAATCATGGCTTCAGAAAATTCCT 7079

MH121164.1995 CCGACCCAGGGACCAACACAACAACTGAAGACCACAAAATCATGGCTTCAGAAAATTCCT 7079

AY354458.1995 CCGACCCAGGGACCAACACAACAACTGAAGACCACAAAATCATGGCTTCAGAAAATTCCT 7080

KT762962.1995 CCGACCCAGGGACCAACACAACAACTGAAGACCACAAAATCATGGCTTCAGAAAATTCCT 7079

HQ613402.2008 CCGACCCAAAGACCAACACAACAACTGAAGACCACAAAATCGTGGCTTCAGAAAATTCCT 6998

KC242789.2007 CCGACCCAAAGACCAACACAACAACTGAAGACCACAAAATCGTGGCTTCAGAAAATTCCT 7079

HQ613403.2007 CCGACCCAAAGACCAACACAACAACTGAAGACCACAAAATCGTGGCTTCAGAAAATTCCT 7031

KC242785.2007 CCGACCCAAAGACCAACACAACAACTGAAGACCACAAAATCGTGGCTTCAGAAAATTCCT 7079

KC242790.2007 CCGACCCAAAGACCAACACAACAACTGAAGACCACAAAATCGTGGCTTCAGAAAATTCCT 7079

KU143789.2014 CCGACCCAGAGACCAACACAACAAATGAAGACCACAAAATCATGGCTTCAGAAAATTCCT 7079

KR817168.2014 CCGACCCAGAGACCAACACAACAAATGAAGACCACAAAATCATGGCTTCAGAAAATTCCT 7053

KY426696.2015 CCGACCCAGAGACCAACACAACAAATGAAGACCACAAAATCATGGCTTCAGAAAATTCCT 7079

KR105271.2014 CCGACCCAGAGACCAACACAACAAATGAAGACCACAAAATCATGGCTTCAGAAAATTCCT 7046

KY007522.2016 CCGACCCAGAGACCAACACAACAAATGAAGACCACAAAATCATGGCTTCAGAAAATTCCT 7059

KM034555.2014 CCGACCCAGAGACCAACACAACAAATGAAGACCACAAAATCATGGCTTCAGAAAATTCCT 7071

MH470381.2015 CCGACCCAGAGACCAACACAACAAATGAAGACCACAAAATCATGGCTTCAGAAAATTCCT 7043

MH470382.2015 CCGACCCAGAGACCAACACAACAAATGAAGACCACAAAATCATGGCTTCAGAAAATTCCT 7043

MF102255.2014 CCGACCCAGAGACCAACACAACAAATGAAGACCACAAAATCATGGCTTCAGAAAATTCCT 7056

KJ660348.2014 CCGACCCAGAGACCAACACAACAAATGAAGACCACAAAATCATGGCTTCAGAAAATTCCT 7079

KU143818.2014 CCGACCCAGAGACCAACACAACAAATGAAGACCACAAAATCATGGCTTCAGAAAATTCCT 7079

KT725333.2014 CCGACCCAGAGACCAACACAACAAATGAAGACCACAAAATCATGGCTTCAGAAAATTCCT 7044

KR819004.2014 CCGACCCAGGGACCAACACAACAACTGAAGACCACAAAATCATGGCTTCAGAAAATTCCT 7026

KP271020.2014 CCGACCCAGGGACCAACACAACAACTGAAGACCACAAAATCATGGCTTCAGAAAATTCCT 7026

KM519951.2014 CCGACCCAGGGACCAACACAACAACTGAAGACCACAAAATCATGGCTTCAGAAAATTCCT 7076

MH733488.2018 CCGACCCAGGGACCAACACAACAACTGAAGGCCACAAAATCATGGCTTCAGAAAATTCCT 7070

MH733491.2018 CCGACCCAGGGACCAACACAACAACTGAAGGCCACAAAATCATGGCTTCAGAAAATTCCT 7065

MH733478.2018 CCGACCCAGGGACCAACACAACAACTGAAGGCCACAAAATCATGGCTTCAGAAAATTCCT 7068

MK007330.2018 CCGACCCAGGTACCAACACAACAACTGAAGACCACAAAATCATGGCTTCAGAAAATTCCT 7067

MK007344.2018 CCGACCCAGGTACCAACACAACAACTGAAGACCACAAAATCATGGCTTCAGAAAATTCCT 7061

******** *** ********* ** ** ********** *********** ******

AY142960.1976 CTGCAATGGTTCAAGTGCACAGTCAAGGAAGGGAAGCTGCAGTGTCGCATCTAACAACCC 7139 end sGP

KC242791.1977 CTGCAATGGTTCAAGTGCACAGTCAAGGAAGGGAAGCTGCAGTGTCGCATCTAACAACCC 7139 -> F primer GP

AF499101.1976 CTGCAATGGTTCAAGTGCACAGTCAAGGAAGGGAAGCTGCAGTGTCGCATCTAACAACCC 7139

KF113528.2003 CTGCAATGGTTCAAGTGCACAATCAAGGAAGGGAAGCTGCAGTGTCGCATCTGATAACCT 7135

KC242800.2002 CTGCAATGGTTCAAGTGCACAATCAAGGAAGGGAAGCTGCAGTGTCGCATCTGATAACCC 7139

KY471090.2001 CTGCAATGGTTCAAGTGCACAATCAAGGAAGGGAAGCTGCAGTGTCGCATCTGATAACCC 7116

KY471092.2001 CTGCAATGGTTCAAGTGCACAATCAAGGAAGGGAAGCTGCAGTGTCGCATCTGATAACCC 7116

MH481611.2017 CTGCAATGGTTCAAGTGCACAGTCAAGGAAGGGAAGCTGCAGTGTCGCATCTGACAACCC 7105

MH613311.2017 CTGCAATGGTTCAAGTGCACAGTCAAGGAAGGGAAGCTGCAGTGTCGCATCTGACAACCC 7084

KC242792.1994 CTGCAATGGTTCAAGTGCACAGTCAAGGAAGGGAAGCTGCAGTGTCGCATCTGACAACCC 7139

KC242793.1996 CTGCAATGGTTCAAGTGCACAGTCAAGGAAGGGAAGCTGCAGTGTCGCATCTGACAACCC 7139

KC242794.1996 CTGCAATGGTTCAAGTGCACAGTCAAGGAAGGGAAGCTGCAGTGTCGCATCTGACAACCC 7139

MH121164.1995 CTGCAATGGTTCAAGTGCACAGTCAAGGAAGGGAAGCTGCAGTGTCGCATCTGACAACCC 7139

AY354458.1995 CTGCAATGGTTCAAGTGCACAGTCAAGGAAGGGAAGCTGCAGTGTCGCATCTGACAACCC 7140

KT762962.1995 CTGCAATGGTTCAAGTGCACAGTCAAGGAAGGGAAGCTGCAGTGTCGCATCTGACAACCC 7139

HQ613402.2008 CTGCAATGGTTCAAGTGCACAGTCAAGGAAGGGAAGCTGCAGTGTCGCATCTGACAACCC 7058

KC242789.2007 CTGCAATGGTTCAAGTGCACAGTCAAGGAAGGGAAGCTGCAGTGTCGCATCTGACAACCC 7139

HQ613403.2007 CTGCAATGGTTCAAGTGCACAGTCAAGGAAGGGAAGCTGCAGTGTCGCATCTGACAACCC 7091

KC242785.2007 CTGCAATGGTTCAAGTGCACAGTCAAGGAAGGGAAGCTGCAGTGTCGCATCTGACAACCC 7139

KC242790.2007 CTGCAATGGTTCAAGTGCACAGTCAAGGAAGGGAAGCTGCAGTGTCGCATCTGACAACCC 7139

KU143789.2014 CTGCAATGGTTCAAGTGCACAGTCAAGGAAGGAAAGCTGCAGTGTCGCATCTGACAACCC 7139

KR817168.2014 CTGCAATGGTTCAAGTGCACAGTCAAGGAAGGAAAGCTGCAGTGTCGCATCTGACAACCC 7113

KY426696.2015 CTGCAATGGTTCAAGTGCACAGTCAAGGAAGGAAAGCTGCAGTGTCGCATCTGACAACCC 7139

KR105271.2014 CTGCAATGGTTCAAGTGCACAGTCAAGGAAGGAAAGCTGCAGTGTCGCATCTGACAACCC 7106

KY007522.2016 CTGCAATGGTTCAAGTGCACAGTCAAGGAAGGAAAGCTGCAGTGTCGCATCTGACAACCC 7119

KM034555.2014 CTGCAATGGTTCAAGTGCACAGTCAAGGAAGGAAAGCTGCAGTGTCGCATCTGACAACCC 7131

MH470381.2015 CTGCAATGGTTCAAGTGCACAGTCAAGGAAGGAAAGCTGCAGTGTCGCATCTGACAACCC 7103

MH470382.2015 CTGCAATGGTTCAAGTGCACAGTCAAGGAAGGAAAGCTGCAGTGTCGCATCTGACGACCC 7103

MF102255.2014 CTGCAATGGTTCAAGTGCACAGTCAAGGAAGGAAAGCTGCAGTGTCGCATCTGACAACCC 7116

KJ660348.2014 CTGCAATGGTTCAAGTGCACAGTCAAGGAAGGAAAGCTGCAGTGTCGCATCTGACAACCC 7139

KU143818.2014 CTGCAATGGTTCAAGTGCACAGTCAAGGAAGGAAAGCTGCAGTGTCGCATCTGACAACCC 7139

KT725333.2014 CTGCAATGGTTCAAGTGCACAGTCAAGGAAGGAAAGCTGCAGTGTCGCATCTGACAACCC 7104

KR819004.2014 CTGCAATGGTTCAAGTGCACAGTCAAGGAAGGGAAGCTGCAGTGTCGCATCTGACAACCC 7086

KP271020.2014 CTGCAATGGTTCAAGTGCACAGTCAAGGAAGGGAAGCTGCAGTGTCGCATCTGACAACCC 7086

KM519951.2014 CTGCAATGGTTCAAGTGCACAGTCAAGGAAGGGAAGCTGCAGTGTCGCATCTGACAACCC 7136

MH733488.2018 CTGCAATGGTTCAAGTGCACAGTCAAGGAAGGGAAGCTGCAGTGTCGCATCTGACAACCC 7130

MH733491.2018 CTGCAATGGTTCAAGTGCACAGTCAAGGAAGGGAAGCTGCAGTGTCGCATCTGACAACCC 7125

MH733478.2018 CTGCAATGGTTCAAGTGCACAGTCAAGGAAGGGAAGCTGCAGTGTCGCATCTGACAACCC 7128

MK007330.2018 CTGCAATGGTTCAAGTGCACAGTCAAGGAAGGGAAGCTGCAGTGTCGCATCTGACAACCC 7127

MK007344.2018 CTGCAATGGTTCAAGTGCACAGTCAAGGAAGGGAAGCTGCAGTGTCGCATCTGACAACCC 7121

********************* ********** ******************* * ***

AY142960.1976 TTGCCACAATCTCCACGAGTCCCCAATCCCTCACAACCAAACCAGGTCCGGACAACAGCA 7199

KC242791.1977 TTGCCACAATCTCCACGAGTCCCCAATCCCTCACAACCAAACCAGGTCCGGACAACAGCA 7199

AF499101.1976 TTGCCACAATCTCCACGAGTCCCCAATCCCTCACAACCAAACCAGGTCCGGACAACAGCA 7199

KF113528.2003 TTGCCACAATCTCCACGAGTCCTCAATCCCCCACAACCAAACCAGGTCAGGACAACAGCA 7195

KC242800.2002 TTGCCACAATCTCCACGAGTCCTCAATCCCCTACAACCAAACCAGGTCAGGACAACAGCA 7199

KY471090.2001 TTGCCACAATCTCCACGAGTCCTCAATCCCCTACAACCAAACCAGGTCAGGACAACAGCA 7176

KY471092.2001 TTGCCACAATCTCCACGAGTCCTCAATCCCCTACAACCAAACCAGGTCAGGACAACAGCA 7176

MH481611.2017 TTGCCACAATCTCCACGAGTCCTCAACCCCTCACAACCAAGCCAGGTCCGGACAACAGCA 7165

MH613311.2017 TTGCCACAATCTCCACGAGTCCTCAACCCCTCACAACCAAGCCAGGTCCGGACAACAGCA 7144

KC242792.1994 TTGCCACAATCTCCACGAGTCTTCGACCCCCCATAACCAAACCAGGTCCGGACAACAGCA 7199

KC242793.1996 TTGCCACAATCTCCACGAGTCTTCAACCCCCCACAACCAAACCAGGTCCGGACAACAGCA 7199

KC242794.1996 CTGCCACAATCTCCACGAGTCTTCAACCCCCCACAACCAAACCAGGTCCGGACAACAGCA 7199

MH121164.1995 TTGCCACAATCTCCACGAGTCCTCAACCCCCCACAACCAAACCAGGTCCGGACAACAGCA 7199

AY354458.1995 TTGCCACAATCTCCACGAGTCCTCAACCCCCCACAACCAAACCAGGTCCGGACAACAGCA 7200

KT762962.1995 TTGCCACAATCTCCACGAGTCCTCAACCCCCCACAACCAAACCAGGTCCGGACAACAGCA 7199

HQ613402.2008 TTGCCACAATCTCCACGAGTCCTCAACCCCCCACAACCAAACCAGGTCCGGACAACAGCA 7118

KC242789.2007 TTGCCACAATCTCCACGAGTCCTCAACCCCCCACAACCAAACCAGGTCCGGACAACAGCA 7199

HQ613403.2007 TTGCCACAATCTCCACGAGTCCTCAACCCCCCACAACCAAACCAGGTCCGGACAACAGCA 7151

KC242785.2007 TTGCCACAATCTCCACGAGTCCTCAACCCCCCACAACCAAACCAGGTCCGGACAACAGCA 7199

KC242790.2007 TTGCCACAATCTCCACGAGTCCTCAACCCCCCACAACCAAACCAGGTCCGGACAACAGCA 7199

KU143789.2014 TTGCCACAATCTCCACGAGTCCTCAACCTCCCACAACCAAAACAGGTCCGGACAACAGCA 7199

KR817168.2014 TTGCCACAATCTCCACGAGTCCTCAACCTCCCACAACCAAAACAGGTCCGGACAACAGCA 7173

KY426696.2015 TTGCCACAGTCTCCACGAGTCCTCAACCTCCCACAACCAAAACAGGTCCGGACAACAGCA 7199

KR105271.2014 TTGCCACAATCTCCACGAGTCCTCAACCTCCCACAACCAAAACAGGTCCGGACAACAGCA 7166

KY007522.2016 TTGCCACAATCTCCACGAGTCCTCAACCTCCCACAACCAAAACAGGTCCGGACAACAGCA 7179

KM034555.2014 TTGCCACAATCTCCACGAGTCCTCAACCTCCCACAACCAAAACAGGTCCGGACAACAGCA 7191

MH470381.2015 TTGCCACAATCTCCACGAGTCCTCAACCTCCCACAACCAAAACAGGTCCGGACAACAGCA 7163

MH470382.2015 TTGCCACAATCTCCACGAGTCCTCAACCTCCCACAACCAAAACAGGTCCGGACAACAGCA 7163

MF102255.2014 TTGCCACAATCTCCACGAGTCCTCAACCTCCCACAACCAAAACAGGTCCGGACAACAGCA 7176

KJ660348.2014 TTGCCACAATCTCCACGAGTCCTCAACCTCCCACAACCAAAACAGGTCCGGACAACAGCA 7199

KU143818.2014 TTGCCACAATCTCCACGAGTCCTCAACCTCCCACAACCAAAACAGGTCCGGACAACAGCA 7199

KT725333.2014 TTGCCACAATCTCCACGAGTCCTCAACCTCCCACAACCAAAACAGGTCCGGACAACAGCA 7164

KR819004.2014 TTGCCACAATCTCCACGAGTCCTCAACCCCCCACAACCAAACCAGGTCCGGACAACAGCA 7146

KP271020.2014 TTGCCACAATCTCCACGAGTCCTCAACCCCCCACAACCAAACCAGGTCCGGACAACAGCA 7146

KM519951.2014 TTGCCACAATCTCCACGAGTCCTCAACCCCCCACAACCAAACCAGGTCCGGACAACAGCA 7196

MH733488.2018 TTGCCACAATCTCCACGAGTCCTCAACCCCCCACAACCAAACCAGGTCCGGACAACAGCA 7190

MH733491.2018 TTGCCACAATCTCCACGAGTCCTCAACCCCCCACAACCAAACCAGGTCCGGACAACAGCA 7185

MH733478.2018 TTGCCACAATCTCCACGAGTCCTCAACCCCCCACAACCAAACCAGGTCCGGACAACAGCA 7188

MK007330.2018 CTGCCACAATCTCCACGAGTCCTCAACCCCTCACAACCAAGCCAGGTCCGGACAACAGCA 7187

MK007344.2018 CTGCCACAATCTCCACGAGTCCTCAACCCCTCACAACCCAGCCAGGTCCGGACAACAGCA 7181

******* ************ * * * * * **** * ****** ***********

AY142960.1976 CCCATAATACACCCGTGTATAAACTTGACATCTCTGAGGCAACTCAAGTTGAACAACATC 7259

KC242791.1977 CCCATAATACACCCGTGTATAAACTTGACATCTCTGAGGCAACTCAAGTTGAACAACATC 7259

AF499101.1976 CCCATAATACACCCGTGTATAAACTTGACATCTCTGAGGCAACTCAAGTTGAACAACATC 7259

KF113528.2003 CCCATAATACACCCGTGTATAAACTTGACATCTCTGAGGCAACTCAAGTTGAACAACATC 7255

KC242800.2002 CCCATAATACACCCGTGTATAAACTTGACATCTCTGAGGCAACTCAAGTTGAACAACATC 7259

KY471090.2001 CCCATAATACACCCGTGTATAAACTTGACATCTCTGAGGCAACTCAAGTTGAACAACATC 7236

KY471092.2001 CCCATAATACACCCGTGTATAAACTTGACATCTCTGAGGCAACTCAAGTTGAACAACATC 7236

MH481611.2017 CCCATAATACACCCGTGTATAAACCTGACATCTCTGAGGCAACTCAAGCCGAACAATACC 7225

MH613311.2017 CCCATAATACACCCGTGTATAAACCTGACATCTCTGAGGCAACTCAAGCCGAACAATACC 7204

KC242792.1994 CCCACAATACACCCGTGTATAAACTTGACATCTCTGAGGCAACTCAAGTTGAACAACATC 7259

KC242793.1996 CCCACAATACACCCGTGTATAAACTTGACATCTCTGAGGCAACTCAAGTTGAACAACATC 7259

KC242794.1996 CCCACAATACACCCGTGTATAAACTTGACATCTCTGAGGCAACTCAAGTTGAACAACATC 7259

MH121164.1995 CCCACAATACACCCGTGTATAAACTTGACATCTCTGAGGCAACTCAAGTTGAACAACATC 7259

AY354458.1995 CCCACAATACACCCGTGTATAAACTTGACATCTCTGAGGCAACTCAAGTTGAACAACATC 7260

KT762962.1995 CCCACAATACACCCGTGTATAAACTTGACATCTCTGAGGCAACTCAAGTTGAACAACATC 7259

HQ613402.2008 CTTATAATACACCCGTATATAAACTTGACACCTCTGAGGCAACTCAAGTTGAACAACATC 7178

KC242789.2007 CTTATAATACACCCGTATATAAACTTGACACCTCTGAGGCAACTCAAGTTGAACAACATC 7259

HQ613403.2007 CTTATAATACACCCGTATATAAACTTGACACCTCTGAGGCAACTCAAGTTGAACAACATC 7211

KC242785.2007 CTTATAATACACCCGTATATAAACTTGACACCTCTGAGGCAACTCAAGTTGAACAACATC 7259

KC242790.2007 CTTATAATACACCCGTATATAAACTTGACACCTCTGAGGCAACTCAAGTTGAACAACATC 7259

KU143789.2014 CCCATAATACACCCGTGTATAAACTTGACATCTCTGAGGCAACTCAAGTTGGACAACATC 7259

KR817168.2014 CCCATAATACACCCGTGTATAAACTTGACATCTCTGAGGCAACTCAAGTTGGACAACATC 7233

KY426696.2015 CCCATAATACACCCGTGTATAAACTTGACATCTCTGAGGCAACTCAAGTTGGACAACATC 7259

KR105271.2014 CCCATAATACACCCGTGTATAAACTTGACATCTCTGAGGCAACTCAAGTTGGACAACATC 7226

KY007522.2016 CCCATAATACACCCGTGTATAAACTTGACATCTCTGAGGCAACTCAAGTTGGACAACATC 7239

KM034555.2014 CCCATAATACACCCGTGTATAAACTTGACATCTCTGAGGCAACTCAAGTTGGACAACATC 7251

MH470381.2015 CCCATAATACACCCGTGTATAAACTTGACATCTCTGAGGCAACTCAAGTTGGACAACATC 7223

MH470382.2015 CCCATAATACACCCGTGTATAAACTTGACATCTCTGAGGCAACTCAAGTTGGACAACATC 7223

MF102255.2014 CCCATAATACACCCGTGTATAAACTTGACATCTCTGAGGCAACTCAAGTTGGACAACATC 7236

KJ660348.2014 CCCATAATACACCCGTGTATAAACTTGACATCTCTGAGGCAACTCAAGTTGGACAACATC 7259
[truncated: 652,470 more chars]
